# Supplementary material for: Propellanes as Rigid Scaffolds for the Stereodefined Attachment of σ-Pharmacophoric Structural Elements to Achieve σ Affinity
Source: Int J Mol Sci. 2021 May 26;22(11):5685. doi: 10.3390/ijms22115685 (PMC8199339; doi:10.3390/ijms22115685)

Supplementary Materials

Propellanes as rigid scaffolds for the stereodefined attachment of σ-pharmacophoric structural elements to achieve σ affinity

Héctor Torres-Gómez,[a] Constantin Daniliuc,[b] Dirk Schepmann,[a] Erik Laurini,[c] Sabrina Pricl,[c,d] Bernhard Wünsch[a,e]*

[a] Institut für Pharmazeutische und Medizinische Chemie der Westfälischen Wilhelms-Universität Münster, Corrensstraße 48, D-48149 Münster, Germany
Tel.: +49-251-8333311; Fax: +49-251-8332144; E-mail: wuensch@uni-muenster.de

[b] Organisch-Chemisches Institut der Westfälischen Wilhelms-Universität Münster, Corrensstraße 40, D-48149 Münster, Germany

[c] Molecular Biology and Nanotechnology Laboratory (MolBNL@UniTS), DEA, University of Trieste, 34127 Trieste, Italy

[d] Department of General Biophysics, Faculty of Biology and Environmental Protection, University of Lodz, Lodz, Poland

[e] GRK 2515, Chemical biology of ion channels (Chembion), Westfälische Wilhelms-Universität Münster, Germany.

Content page

1. Purity data of prepared compounds S2
2. Receptor binding studies S4
3. Crystallography section S8
4. Molecular Dynamics study to determine the binding mode of **18** S77
5. NMR spectra S78
6. HPLC traces of selected compounds S135

1. Purity data of prepared compounds

Purity by HPLC

compound	purity
anti-**7**	96 %
syn-**7**	96%
8-*anti*-**4b**	96 %
8-*anti*-**4c**	93 %
8-*anti*-**4d**	89 %
8-*anti*-**4e**	91 %
8-*anti*-**4f**	92 %
8-*anti*-**4g**	98 %
8-*anti*-**4h**	98 %
8-*anti*-**4i**	91 %
8-*syn*-**4i**	98 %
8-*anti*-**4k**	66 %
8-*syn*-**4k**	97 %
8-*anti*-**4l**	98 %
8-*anti*-**4m**	96 %
8-*anti*-**4n**	95 %
8-*anti*-**4o**	98 %
8-*anti*-**4p**	97 %
8-*anti*-**4q**	98 %
8-*anti*-**4r**	92 %
8-*anti*-**4s**	93 %
8-*anti*-**4t**	94 %
8-*anti*-**4u**	96 %
11-*anti*-**13**	94 %
11-*syn*-**13**	93 %
anti-**14**	98 %
syn-**14**	84 %

**15**	97 %
**17**	96 %
**18**	97 %
syn-**20**	75 %
anti-**20**	74 %
syn-**21**	95 %
anti-**21**	81 %
syn,anti-**22**	95 %
syn,syn-**22**	97 %
anti,anti-**22**	96 %
anti,syn-**22**	94 %
**23a**	97 %
**23b**	98 %
**23c**	94 %
**23d**	98 %
syn-**25**	93 %
anti-**25**	99 %
syn,anti-**26**	99 %
syn,syn-**26**	99 %
anti,anti-**26**	97 %
anti,syn-**26**	94 %

2. Receptor binding studies

2.1. Materials

Guinea pig brains and rat livers were commercially available (Harlan-Winkelmann, Borchen, Germany). Homogenizers: Elvehjem Potter (B. Braun Biotech International, Melsungen, Germany) and Soniprep® 150 (MSE, London, UK). Centrifuges: Cooling centrifuge model Eppendorf 5427R (Eppendorf, Hamburg, Germany) and High-speed cooling centrifuge model Sorvall® RC-5C plus (Thermo Fisher Scientific, Langenselbold, Germany). Multiplates: standard 96 well multiplates (Diagonal, Muenster, Germany). Shaker: self-made device with adjustable temperature and tumbling speed (scientific workshop of the institute). Harvester: MicroBeta® FilterMate 96 Harvester. Filter: Printed Filtermat Typ A and B. Scintillator: Meltilex® (Typ A or B) solid state scintillator. Scintillation analyzer: MicroBeta® Trilux (all Perkin Elmer LAS, Rodgau-Jügesheim, Germany).

2.2. Preparation of membrane homogenates from guinea pig brain

5 guinea pig brains were homogenized with the potter (500-800 rpm, 10 up and down strokes) in 6 volumes of cold 0.32 M sucrose. The suspension was centrifuged at 1,200 x g for 10 min at 4 °C. The supernatant was separated and centrifuged at 23,500 x g for 20 min at 4 °C. The pellet was resuspended in 5-6 volumes of buffer (50 mM TRIS, pH 7.4) and centrifuged again at 23,500 x g (20 min, 4 °C). This procedure was repeated twice. The final pellet was resuspended in 5-6 volumes of buffer and frozen (-80 °C) in 1.5 mL portions containing about 1.5 mg protein/mL.

2.3. Preparation of membrane homogenates from rat liver

Two rat livers were cut into small pieces and homogenized with the potter (500-800 rpm, 10 up and down strokes) in 6 volumes of cold 0.32 M sucrose. The suspension was centrifuged at 1,200 x g for 10 min at 4 °C. The supernatant was separated and centrifuged at 31,000 x g for 20 min at 4 °C. The pellet was resuspended in 5-6 volumes of buffer (50 mM TRIS, pH 8.0) and incubated at rt for 30 min. After the incubation, the suspension was centrifuged again at 31,000 x g for 20 min at 4 °C. The final pellet was

resuspended in 5-6 volumes of buffer and stored at -80 °C in 1.5 mL portions containing about 2 mg protein/mL.

2.4. Protein determination

The protein concentration was determined by the method of Bradford,[1] modified by Stoscheck.[2] The Bradford solution was prepared by dissolving 5 mg of Coomassie Brilliant Blue G 250 in 2.5 mL of EtOH (95 %, v/v). 10 mL deionized H_2O and 5 mL phosphoric acid (85 %, m/v) were added to this solution, the mixture was stirred and filled to a total volume of 50 mL with deionized water. The calibration was carried out using bovine serum albumin as a standard in 9 concentrations (0.1, 0.2, 0.4, 0.6, 0.8, 1.0, 1.5, 2.0 and 4.0 mg /mL). In a 96 well standard multiplate, 10 µL of the calibration solution or 10 µL of the membrane receptor preparation were mixed with 190 µL of the Bradford solution, respectively. After 5 min, the UV absorption of the protein-dye complex at λ = 595 nm was measured with a plate reader (Tecan Genios®, Tecan, Crailsheim, Germany).

2.5. General procedures for the binding assays

The test compound solutions were prepared by dissolving approximately 10 µmol (usually 2-4 mg) of test compound in DMSO so that a 10 mM stock solution was obtained. To obtain the required test solutions for the assay, the DMSO stock solution was diluted with the respective assay buffer. The filtermats were presoaked in 0.5 % aqueous polyethylenimine solution for 2 h at rt before use. All binding experiments were carried out in duplicates in the 96 well multiplates. The concentrations given are the final concentration in the assay. Generally, the assays were performed by addition of 50 µL of the respective assay buffer, 50 µL of test compound solution in various concentrations (10^{-5}, 10^{-6}, 10^{-7}, 10^{-8}, 10^{-9} and 10^{-10} mol/L), 50 µL of the corresponding radioligand solution and 50 µL of the respective receptor preparation into each well of the multiplate (total volume 200 µL). The receptor preparation was always added last. During the incubation, the multiplates were shaken at a speed of 500-600 rpm at the specified temperature. Unless otherwise noted, the assays were terminated after 120 min by rapid filtration using the harvester. During the filtration, each well was washed five times with 300 µL of water.

Subsequently, the filtermats were dried at 95 °C. The solid scintillator was melted on the dried filtermats at a temperature of 95 °C for 5 min. After solidifying of the scintillator at rt, the trapped radioactivity in the filtermats was measured with the scintillation analyzer. Each position on the filtermat corresponding to one well of the multiplate was measured for 5 min with the [^{3}H]-counting protocol. The overall counting efficiency was 20 %. The IC_{50} values were calculated with the program GraphPad Prism® 3.0 (GraphPad Software, San Diego, CA, USA) by non-linear regression analysis. Subsequently, the IC_{50} values were transformed into K_i values using the equation of Cheng and Prusoff.[3] The K_i values are given as mean value ± SEM from three independent experiments.

2.6. σ_1 receptor assay

The assay was performed with the radioligand [^{3}H]-(+)-pentazocine (22.0 Ci/mmol; Perkin Elmer). The thawed membrane preparation of guinea pig brain (about 100 µg of the protein) was incubated with various concentrations of test compounds, 2 nM [^{3}H]-(+)-pentazocine, and TRIS buffer (50 mM, pH 7.4) at 37 °C. The non-specific binding was determined with 10 µM unlabeled (+)-pentazocine. The K_d value of (+)-pentazocine is 2.9 nM.[4]

2.7. σ_2 receptor assay

The assays were performed with the radioligand [^{3}H]di-*o*-tolylguanidine (specific activity 50 Ci/mmol; ARC, St. Louis, MO, USA). The thawed rat liver membrane preparation (about 100 µg protein) was incubated with various concentrations of the test compound, 3 nM [^{3}H]di-*o*-tolylguanidine and buffer containing (+)-pentazocine (500 nM (+)-pentazocine in TRIS buffer (50 mM TRIS, pH 8.0)) at rt. The non-specific binding was determined with 10 µM non-labeled di-*o*-tolylguanidine. The K_d value of di-*o*-tolylguanidine is 17.9 nM.[5]

2.8. References for receptor binding studies

1. M. M. Bradford, A rapid and sensitive method for the quantitation of microgram quantities of protein utilizing the principle of protein-dye binding, Anal. Biochem. 72 (1976) 248–254.
2. C. Stoscheck, Quantification of protein, Methods Enzymol. 182 (1990) 50–68.
3. Y.-C. Cheng, W. H. Prusoff, Relationship between the inhibition constant (KI) and the concentration of inhibitor which causes 50 per cent inhibition (I50) of an enzymatic reaction, Biochem. Pharmacol. 22 (1973) 3099–3108.
4. D. L. DeHaven-Hudkins, L. C. Fleissner, F. Y. Ford-Rice, Characterization of the binding of [3H](+)-pentazocine to σ recognition sites in guinea pig brain, Eur. J. Pharmacol. Mol. Pharmacol. 227 (1992) 371–378.
5. R. H. Mach, C. R. Smith, S. R. Childers, Ibogaine possesses a selective affinity for σ2 receptors, Life Sci. 57 (1995) PL57–PL62.

3. Crystallography section

3.1. General

X-Ray diffraction: Data sets for compounds *syn*-**7**, *anti*-**21**, *syn,anti*-**22**, *anti,syn*-**22** and *syn*-**23** were collected with a Nonius Kappa CCD diffractometer. Programs used: data collection, COLLECT;[1] data reduction Denzo-SMN;[2] absorption correction;[3] structure solution *SHELXT-2015*;[4] structure refinement *SHELXL-2015*[5] and graphics, *XP*.[6] *R*-values are given for observed reflections, and wR^2 values are given for all reflections.

3.2. X-ray crystal structure of *syn*-7

Carbamate *syn*-**7** was crystallized from EiOAc. Colorless crystals. Code DAN7291

X-ray crystal structure analysis of syn-**7** *(dan7291):* A colorless plate-like specimen of $C_{21}H_{27}NO_4$, approximate dimensions 0.100 mm x 0.200 mm x 0.350 mm, was used for the X-ray crystallographic analysis. The X-ray intensity data were measured.

The integration of the data using a monoclinic unit cell yielded a total of 3302 reflections to a maximum θ angle of 67.19° (0.84 Å resolution), of which 3302 were independent (average redundancy 1.000, completeness = 97.6%, R_{sig} = 2.02%) and 3062 (92.73%) were greater than 2σ(F^2).

The final cell constants of a = 6.9404(2) Å, b = 12.2651(4) Å, c = 22.3938(10) Å, β = 98.330(2)°, volume = 1886.15(12) $Å^3$, are based upon the refinement of the XYZ-centroids of reflections above 20 σ(I). Data were corrected for absorption effects using the multi-scan method (SADABS). The calculated minimum and maximum transmission coefficients (based on crystal size) are 0.7920 and 0.9330.

The structure was solved and refined using the Bruker SHELXTL Software Package, using the space group P 1 21/c 1, with Z = 4 for the formula unit, $C_{21}H_{27}NO_4$. The final anisotropic full-matrix least-squares refinement on F^2 with 241 variables converged at R1 = 3.98%, for the observed data and wR2 = 9.96% for all data. The goodness-of-fit was 1.054. The largest peak in the final difference electron density synthesis was 0.198 $e^-/Å^3$ and the largest hole was -0.192 $e^-/Å^3$ with an RMS deviation of 0.034 $e^-/Å^3$. On the basis of the final model, the calculated density was 1.259 g/cm^3 and F(000), 768 e^-. CCDC number: 2073466.

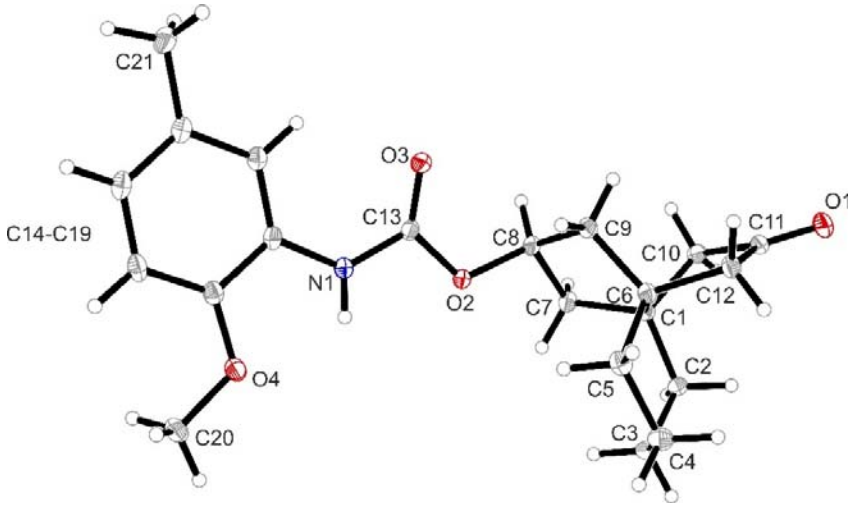

Table S1. Sample and crystal data for *syn*-**7**.

Identification code	dan7291	
Chemical formula	$C_{21}H_{27}NO_4$	
Formula weight	357.43 g/mol	
Temperature	223(2) K	
Wavelength	1.54178 Å	
Crystal size	0.100 x 0.200 x 0.350 mm	
Crystal habit	colorless plate	
Crystal system	monoclinic	
Space group	P 1 21/c 1	
Unit cell dimensions	a = 6.9404(2) Å	α = 90°
	b = 12.2651(4) Å	β = 98.330(2)°
	c = 22.3938(10) Å	γ = 90°
Volume	1886.15(12) $Å^3$	
Z	4	
Density (calculated)	1.259 g/cm^3	
Absorption coefficient	0.699 mm^{-1}	
F(000)	768	

Table S2. Data collection and structure refinement for *syn*-**7**.

Theta range for data collection	3.99 to 67.19°	
Reflections collected	3302	
Absorption correction	multi-scan	
Max. and min. transmission	0.9330 and 0.7920	
Structure solution technique	direct methods	
Structure solution program	SHELXL-2014/7 (Sheldrick, 2014)	
Refinement method	Full-matrix least-squares on F^2	
Refinement program	SHELXL-2014/7 (Sheldrick, 2014)	
Function minimized	$\Sigma w(F_o^2 - F_c^2)^2$	
Data / restraints / parameters	3302 / 0 / 241	
Goodness-of-fit on F^2	1.054	
Final R indices	3062 data; I>2σ(I)	R1 = 0.0398, wR2 = 0.0974
	all data	R1 = 0.0423, wR2 = 0.0996
Weighting scheme	$w=1/[\sigma^2(F_o^2)+(0.0439P)^2+0.5589P]$ where $P=(F_o^2+2F_c^2)/3$	
Largest diff. peak and hole	0.198 and -0.192 eÅ^{-3}	
R.M.S. deviation from mean	0.034 eÅ^{-3}	

Table S3. Atomic coordinates and equivalent isotropic atomic displacement parameters (Å^2) for *syn*-**7**.

U(eq) is defined as one third of the trace of the orthogonalized U_{ij} tensor.

	x/a	y/b	z/c	U(eq)
O1	0.18777(18)	0.50705(9)	0.55471(6)	0.0634(3)
O2	0.13493(13)	0.07912(7)	0.37690(4)	0.0408(2)
O3	0.81958(15)	0.08786(8)	0.33377(5)	0.0521(3)
O4	0.10912(16)	0.73057(8)	0.32734(5)	0.0511(3)
N1	0.00244(19)	0.93361(10)	0.32961(6)	0.0439(3)
C1	0.34633(18)	0.28500(10)	0.47320(6)	0.0333(3)
C2	0.54633(19)	0.27699(12)	0.51195(6)	0.0416(3)
C3	0.5967(2)	0.16265(13)	0.53496(8)	0.0541(4)
C4	0.4417(3)	0.11989(14)	0.57038(8)	0.0583(4)
C5	0.2438(2)	0.11788(12)	0.53067(7)	0.0477(4)

	x/a	y/b	z/c	U(eq)
C6	0.18106(19)	0.22849(10)	0.50237(6)	0.0359(3)
C7	0.3370(2)	0.23240(12)	0.41101(6)	0.0406(3)
C8	0.12640(19)	0.19445(10)	0.39283(6)	0.0391(3)
C9	0.02181(19)	0.21310(11)	0.44765(6)	0.0416(3)
C10	0.2811(2)	0.40498(11)	0.47030(6)	0.0410(3)
C11	0.1958(2)	0.42160(11)	0.52794(7)	0.0436(3)
C12	0.1268(2)	0.31271(12)	0.54799(7)	0.0447(3)
C13	0.9699(2)	0.03815(11)	0.34554(6)	0.0387(3)
C14	0.8653(2)	0.85987(11)	0.29939(6)	0.0397(3)
C15	0.9243(2)	0.75097(11)	0.29824(6)	0.0425(3)
C16	0.7980(3)	0.67379(12)	0.26971(7)	0.0509(4)
C17	0.6139(2)	0.70453(13)	0.24203(7)	0.0517(4)
C18	0.5529(2)	0.81163(12)	0.24229(6)	0.0453(3)
C19	0.6811(2)	0.88905(11)	0.27142(6)	0.0427(3)
C20	0.1808(3)	0.62199(14)	0.32622(8)	0.0682(5)
C21	0.3550(2)	0.84624(15)	0.21131(8)	0.0588(4)

Table S4. Bond lengths (Å) for *syn*-**7.**

O1-C11	1.2128(17)	O2-C13	1.3507(16)
O2-C8	1.4621(15)	O3-C13	1.2041(16)
O4-C15	1.3751(18)	O4-C20	1.4231(19)
N1-C13	1.3586(18)	N1-C14	1.4125(18)
N1-H01	0.866(18)	C1-C7	1.5278(18)
C1-C2	1.5298(18)	C1-C10	1.5382(18)
C1-C6	1.5621(17)	C2-C3	1.517(2)
C2-H2A	0.98	C2-H2B	0.98
C3-C4	1.520(2)	C3-H3A	0.98
C3-H3B	0.98	C4-C5	1.525(2)
C4-H4A	0.98	C4-H4B	0.98
C5-C6	1.5335(19)	C5-H5A	0.98
C5-H5B	0.98	C6-C12	1.5375(18)

C6-C9	1.5384(19)	C7-C8	1.5319(18)
C7-H7A	0.98	C7-H7B	0.98
C8-C9	1.5311(19)	C8-H8	0.99
C9-H9A	0.98	C9-H9B	0.98
C10-C11	1.510(2)	C10-H10A	0.98
C10-H10B	0.98	C11-C12	1.509(2)
C12-H12A	0.98	C12-H12B	0.98
C14-C19	1.387(2)	C14-C15	1.3983(19)
C15-C16	1.382(2)	C16-C17	1.389(2)
C16-H16	0.94	C17-C18	1.380(2)
C17-H17	0.94	C18-C19	1.3966(19)
C18-C21	1.508(2)	C19-H19	0.94
C20-H20A	0.97	C20-H20B	0.97
C20-H20C	0.97	C21-H21A	0.97
C21-H21B	0.97	C21-H21C	0.97

Table S5. Bond angles (°) for *syn*-**7**.

C13-O2-C8	115.18(10)	C15-O4-C20	117.80(13)
C13-N1-C14	127.15(13)	C13-N1-H01	116.8(11)
C14-N1-H01	115.7(11)	C7-C1-C2	113.89(11)
C7-C1-C10	113.09(11)	C2-C1-C10	108.83(10)
C7-C1-C6	104.90(10)	C2-C1-C6	113.28(11)
C10-C1-C6	102.28(10)	C3-C2-C1	113.43(12)
C3-C2-H2A	108.9	C1-C2-H2A	108.9
C3-C2-H2B	108.9	C1-C2-H2B	108.9
H2A-C2-H2B	107.7	C2-C3-C4	110.51(13)
C2-C3-H3A	109.5	C4-C3-H3A	109.5
C2-C3-H3B	109.5	C4-C3-H3B	109.5
H3A-C3-H3B	108.1	C3-C4-C5	110.32(13)
C3-C4-H4A	109.6	C5-C4-H4A	109.6
C3-C4-H4B	109.6	C5-C4-H4B	109.6
H4A-C4-H4B	108.1	C4-C5-C6	113.87(12)

C4-C5-H5A	108.8	C6-C5-H5A	108.8
C4-C5-H5B	108.8	C6-C5-H5B	108.8
H5A-C5-H5B	107.7	C5-C6-C12	113.71(11)
C5-C6-C9	110.42(11)	C12-C6-C9	113.25(11)
C5-C6-C1	112.56(11)	C12-C6-C1	104.19(10)
C9-C6-C1	101.98(10)	C1-C7-C8	106.45(10)
C1-C7-H7A	110.4	C8-C7-H7A	110.4
C1-C7-H7B	110.4	C8-C7-H7B	110.4
H7A-C7-H7B	108.6	O2-C8-C9	112.39(11)
O2-C8-C7	106.74(11)	C9-C8-C7	106.66(10)
O2-C8-H8	110.3	C9-C8-H8	110.3
C7-C8-H8	110.3	C8-C9-C6	106.72(10)
C8-C9-H9A	110.4	C6-C9-H9A	110.4
C8-C9-H9B	110.4	C6-C9-H9B	110.4
H9A-C9-H9B	108.6	C11-C10-C1	104.04(11)
C11-C10-H10A	110.9	C1-C10-H10A	110.9
C11-C10-H10B	110.9	C1-C10-H10B	110.9
H10A-C10-H10B	109.0	O1-C11-C12	125.33(14)
O1-C11-C10	126.25(14)	C12-C11-C10	108.39(11)
C11-C12-C6	106.15(11)	C11-C12-H12A	110.5
C6-C12-H12A	110.5	C11-C12-H12B	110.5
C6-C12-H12B	110.5	H12A-C12-H12B	108.7
O3-C13-O2	124.61(12)	O3-C13-N1	126.31(13)
O2-C13-N1	109.08(12)	C19-C14-C15	119.43(13)
C19-C14-N1	124.36(13)	C15-C14-N1	116.21(13)
O4-C15-C16	125.45(13)	O4-C15-C14	114.87(12)
C16-C15-C14	119.67(14)	C15-C16-C17	120.08(14)
C15-C16-H16	120.0	C17-C16-H16	120.0
C18-C17-C16	121.27(14)	C18-C17-H17	119.4
C16-C17-H17	119.4	C17-C18-C19	118.31(14)
C17-C18-C21	121.70(13)	C19-C18-C21	119.98(14)
C14-C19-C18	121.24(14)	C14-C19-H19	119.4

C18-C19-H19	119.4	O4-C20-H20A	109.5
O4-C20-H20B	109.5	H20A-C20-H20B	109.5
O4-C20-H20C	109.5	H20A-C20-H20C	109.5
H20B-C20-H20C	109.5	C18-C21-H21A	109.5
C18-C21-H21B	109.5	H21A-C21-H21B	109.5
C18-C21-H21C	109.5	H21A-C21-H21C	109.5
H21B-C21-H21C	109.5		

Table S6. Torsion angles (°) for *syn*-**7.**

C7-C1-C2-C3	-71.62(15)	C10-C1-C2-C3	161.20(12)
C6-C1-C2-C3	48.17(16)	C1-C2-C3-C4	-56.53(17)
C2-C3-C4-C5	59.27(17)	C3-C4-C5-C6	-55.86(18)
C4-C5-C6-C12	-70.87(16)	C4-C5-C6-C9	160.55(12)
C4-C5-C6-C1	47.32(16)	C7-C1-C6-C5	82.04(13)
C2-C1-C6-C5	-42.76(15)	C10-C1-C6-C5	-159.72(11)
C7-C1-C6-C12	-154.31(11)	C2-C1-C6-C12	80.89(13)
C10-C1-C6-C12	-36.06(13)	C7-C1-C6-C9	-36.28(12)
C2-C1-C6-C9	-161.08(11)	C10-C1-C6-C9	81.97(11)
C2-C1-C7-C8	151.28(11)	C10-C1-C7-C8	-83.79(13)
C6-C1-C7-C8	26.86(14)	C13-O2-C8-C9	79.36(14)
C13-O2-C8-C7	-164.06(11)	C1-C7-C8-O2	-126.92(11)
C1-C7-C8-C9	-6.58(15)	O2-C8-C9-C6	100.00(12)
C7-C8-C9-C6	-16.64(14)	C5-C6-C9-C8	-87.41(13)
C12-C6-C9-C8	143.76(11)	C1-C6-C9-C8	32.42(13)
C7-C1-C10-C11	149.80(11)	C2-C1-C10-C11	-82.58(13)
C6-C1-C10-C11	37.53(12)	C1-C10-C11-O1	152.95(14)
C1-C10-C11-C12	-25.41(14)	O1-C11-C12-C6	-175.87(13)
C10-C11-C12-C6	2.51(15)	C5-C6-C12-C11	143.91(12)
C9-C6-C12-C11	-88.97(13)	C1-C6-C12-C11	21.01(14)
C8-O2-C13-O3	-4.48(19)	C8-O2-C13-N1	175.17(11)
C14-N1-C13-O3	-4.1(2)	C14-N1-C13-O2	176.25(12)
C13-N1-C14-C19	12.2(2)	C13-N1-C14-C15	-168.15(13)

C20-O4-C15-C16	2.6(2)	C20-O4-C15-C14	-178.00(13)
C19-C14-C15-O4	-179.89(12)	N1-C14-C15-O4	0.39(18)
C19-C14-C15-C16	-0.5(2)	N1-C14-C15-C16	179.79(13)
O4-C15-C16-C17	179.70(13)	C14-C15-C16-C17	0.4(2)
C15-C16-C17-C18	0.0(2)	C16-C17-C18-C19	-0.2(2)
C16-C17-C18-C21	178.73(14)	C15-C14-C19-C18	0.3(2)
N1-C14-C19-C18	179.95(13)	C17-C18-C19-C14	0.1(2)
C21-C18-C19-C14	-178.88(13)		

TableS 7. Anisotropic atomic displacement parameters (Å^2) for *syn*-**7.**

The anisotropic atomic displacement factor exponent takes the form: $-2\pi^2[h^2 a^{*2} U_{11} + ... + 2 h k a^* b^* U_{12}]$

	U_{11}	U_{22}	U_{33}	U_{23}	U_{13}	U_{12}
O1	0.0706(7)	0.0445(6)	0.0729(8)	-0.0219(5)	0.0033(6)	0.0064(5)
O2	0.0419(5)	0.0351(5)	0.0426(5)	-0.0071(4)	-0.0032(4)	0.0014(4)
O3	0.0469(6)	0.0394(5)	0.0639(7)	-0.0082(5)	-0.0123(5)	0.0037(5)
O4	0.0639(7)	0.0403(5)	0.0475(6)	-0.0042(4)	0.0029(5)	0.0075(5)
N1	0.0455(7)	0.0355(6)	0.0481(7)	-0.0070(5)	-0.0015(5)	0.0004(5)
C1	0.0323(6)	0.0323(6)	0.0349(6)	-0.0017(5)	0.0038(5)	-0.0019(5)
C2	0.0339(7)	0.0454(8)	0.0438(7)	-0.0031(6)	-0.0002(6)	0.0004(6)
C3	0.0472(8)	0.0546(9)	0.0570(9)	0.0034(7)	-0.0049(7)	0.0127(7)
C4	0.0738(11)	0.0477(9)	0.0515(9)	0.0128(7)	0.0026(8)	0.0096(8)
C5	0.0584(9)	0.0376(7)	0.0492(8)	0.0052(6)	0.0148(7)	-0.0027(6)
C6	0.0364(7)	0.0333(7)	0.0392(7)	-0.0032(5)	0.0090(5)	-0.0022(5)
C7	0.0405(7)	0.0448(8)	0.0369(7)	-0.0053(6)	0.0074(6)	-0.0055(6)
C8	0.0430(7)	0.0322(7)	0.0399(7)	-0.0051(5)	-0.0009(6)	-0.0015(5)
C9	0.0339(7)	0.0388(7)	0.0520(8)	-0.0091(6)	0.0056(6)	-0.0041(5)
C10	0.0418(7)	0.0325(7)	0.0465(8)	0.0009(6)	-0.0005(6)	-0.0035(6)
C11	0.0391(7)	0.0393(7)	0.0497(8)	-0.0101(6)	-0.0025(6)	0.0058(6)
C12	0.0441(8)	0.0466(8)	0.0450(8)	-0.0093(6)	0.0119(6)	-0.0010(6)
C13	0.0443(8)	0.0353(7)	0.0347(7)	-0.0023(5)	-0.0002(6)	-0.0023(6)
C14	0.0508(8)	0.0357(7)	0.0330(6)	-0.0026(5)	0.0078(6)	-0.0063(6)

C15	0.0564(9)	0.0388(7)	0.0327(7)	-0.0001(5)	0.0076(6)	-0.0008(6)
C16	0.0748(11)	0.0332(7)	0.0455(8)	-0.0045(6)	0.0119(7)	-0.0061(7)
C17	0.0646(10)	0.0452(8)	0.0452(8)	-0.0075(6)	0.0076(7)	-0.0168(7)
C18	0.0526(8)	0.0458(8)	0.0382(7)	-0.0037(6)	0.0088(6)	-0.0120(7)
C19	0.0507(8)	0.0379(7)	0.0393(7)	-0.0022(6)	0.0060(6)	-0.0053(6)
C20	0.0945(14)	0.0513(10)	0.0550(10)	-0.0099(8)	-0.0023(9)	0.0252(9)
C21	0.0534(9)	0.0602(10)	0.0613(10)	-0.0089(8)	0.0029(8)	-0.0131(8)

Table S8. Hydrogen atomic coordinates and isotropic atomic displacement parameters (Å^2) for *syn*-**7.**

	x/a	y/b	z/c	U(eq)
H01	0.116(3)	-0.0936(14)	0.3429(8)	0.053(5)
H2A	0.6458	0.3016	0.4881	0.05
H2B	0.5496	0.3263	0.5465	0.05
H3A	0.7232	0.1635	0.5609	0.065
H3B	0.6068	0.1142	0.5008	0.065
H4A	0.4352	0.1667	0.6055	0.07
H4B	0.4762	0.0461	0.5850	0.07
H5A	0.1455	0.0934	0.5550	0.057
H5B	0.2479	0.0645	0.4983	0.057
H7A	0.4265	0.1703	0.4127	0.049
H7B	0.3730	0.2853	0.3817	0.049
H8	0.0630	0.2371	0.3579	0.047
H9A	-0.0601	0.1502	0.4539	0.05
H9B	-0.0609	0.2781	0.4417	0.05
H10A	0.3920	0.4538	0.4686	0.049
H10B	0.1832	0.4182	0.4349	0.049
H12A	-0.0144	0.3137	0.5480	0.054
H12B	0.1909	0.2952	0.5888	0.054
H16	-0.1632	-0.3996	0.2690	0.061
H17	-0.4708	-0.3486	0.2227	0.062
H19	-0.3582	-0.0377	0.2721	0.051

H20A	0.0997	-0.4261	0.3463	0.102
H20B	0.3136	-0.3807	0.3469	0.102
H20C	0.1780	-0.4012	0.2847	0.102
H21A	-0.7151	-0.2170	0.1935	0.088
H21B	-0.6298	-0.1013	0.1799	0.088
H21C	-0.7173	-0.1207	0.2405	0.088

3.3. X-ray crystal structure of *anti*-21

Compound *anti*-**21** was crystallized from EtOAc. Colorless crystals. Code DAN6940

*X-ray crystal structure analysis of anti-***21** *(dan6940):* A colorless prism-like specimen of $C_{28}H_{34}N_2O_3$, approximate dimensions 0.060 mm x 0.240 mm x 0.260 mm, was used for the X-ray crystallographic analysis. The X-ray intensity data were measured.

The integration of the data using a monoclinic unit cell yielded a total of 4159 reflections to a maximum θ angle of 67.08° (0.84 Å resolution), of which 4159 were independent (average redundancy 1.000, completeness = 96.3%, R_{sig} = 2.90%) and 3509 (84.37%) were greater than 2σ(F^2).

The final cell constants of a = 8.5091(4) Å, b = 15.9061(5) Å, c = 17.8829(6) Å, β = 94.139(3)°, volume = 2414.08(16) Å^3, are based upon the refinement of the XYZ-centroids of reflections above 20 σ(I). Data were corrected for absorption effects using the multi-scan method (SADABS). The calculated minimum and maximum transmission coefficients (based on crystal size) are 0.8530 and 0.9630.

The structure was solved and refined using the Bruker SHELXTL Software Package, using the space group P 1 21/n 1, with Z = 4 for the formula unit, $C_{28}H_{34}N_2O_3$. The final anisotropic full-matrix least-squares refinement on F^2 with 304 variables converged at R1 = 7.05%, for the observed data and wR2 = 20.59% for all data. The goodness-of-fit was 1.041. The largest peak in the final difference electron density synthesis was 0.482 e^-/Å^3 and the largest hole was -0.353 e^-/Å^3 with an RMS deviation of 0.056 e^-/Å^3. On the basis of the final model, the calculated density was 1.229 g/cm^3 and F(000), 960 e^-. CCDC number: 2073467.

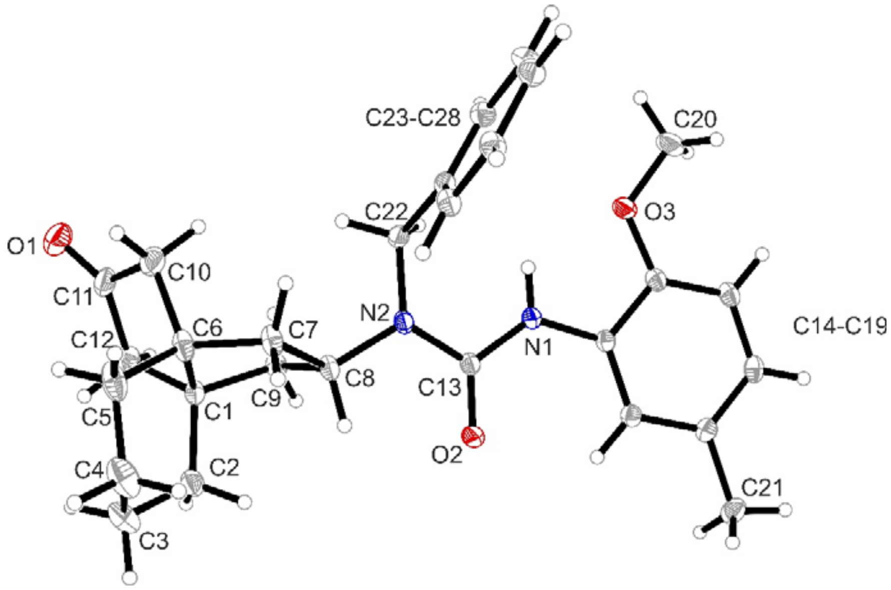

Table S9. Sample and crystal data for *anti*-**21**.

Identification code	dan6940	
Chemical formula	$C_{28}H_{34}N_2O_3$	
Formula weight	446.57 g/mol	
Temperature	223(2) K	
Wavelength	1.54178 Å	
Crystal size	0.060 x 0.240 x 0.260 mm	
Crystal habit	colorless prism	
Crystal system	Monoclinic	
Space group	P 1 21/n 1	
Unit cell dimensions	a = 8.5091(4) Å	α = 90°
	b = 15.9061(5) Å	β = 94.139(3)°
	c = 17.8829(6) Å	γ = 90°
Volume	2414.08(16) Å^3	
Z	4	
Density (calculated)	1.229 g/cm^3	
Absorption coefficient	0.629 mm^{-1}	
F(000)	960	

Table S10. Data collection and structure refinement for *anti*-**21.**

Theta range for data collection	4.96 to 67.08°	
Reflections collected	4159	
Absorption correction	multi-scan	
Max. and min. transmission	0.9630 and 0.8530	
Structure solution technique	direct methods	
Structure solution program	SHELXL-2014/7 (Sheldrick, 2014)	
Refinement method	Full-matrix least-squares on F^2	
Refinement program	SHELXL-2014/7 (Sheldrick, 2014)	
Function minimized	Σ w(F_o^2 - F_c^2)^2	
Data / restraints / parameters	4159 / 0 / 304	
Goodness-of-fit on F^2	1.041	
Final R indices	3509 data; I>2σ(I)	R1 = 0.0705, wR2 = 0.1949
	all data	R1 = 0.0803, wR2 = 0.2059
Weighting scheme	w=1/[σ^2(F_o^2)+(0.1136P)^2+1.6562P]<br>where P=(F_o^2+2F_c^2)/3	
Largest diff. peak and hole	0.482 and -0.353 eÅ^{-3}	
R.M.S. deviation from mean	0.056 eÅ^{-3}	

Table S11. Atomic coordinates and equivalent isotropic atomic displacement parameters (Å^2) for *anti*-**21.**

U(eq) is defined as one third of the trace of the orthogonalized U_{ij} tensor.

	x/a	y/b	z/c	U(eq)
N1	0.3799(2)	0.30176(12)	0.36623(12)	0.0432(5)
N2	0.4569(3)	0.43763(13)	0.39526(12)	0.0423(5)
O1	0.4124(4)	0.92214(16)	0.44969(18)	0.0965(9)
O2	0.1968(2)	0.40252(11)	0.38865(12)	0.0541(5)
O3	0.7226(2)	0.51224(12)	0.40155(12)	0.0567(5)
C1	0.1636(3)	0.10507(15)	0.41148(14)	0.0450(6)
C2	0.9881(3)	0.12482(19)	0.40805(18)	0.0592(7)
C3	0.8887(4)	0.0658(3)	0.3582(2)	0.0829(11)
C4	0.9525(5)	0.0604(3)	0.2809(2)	0.0902(13)
C5	0.1215(5)	0.0289(2)	0.28567(18)	0.0763(11)

C6	0.2283(4)	0.08500(18)	0.33462(16)	0.0606(8)
C7	0.2650(4)	0.17143(17)	0.30006(15)	0.0561(7)
C8	0.2580(3)	0.23632(16)	0.36431(14)	0.0464(6)
C9	0.2587(3)	0.18312(16)	0.43618(14)	0.0496(6)
C10	0.3798(4)	0.0327(2)	0.3561(2)	0.0737(9)
C11	0.3418(4)	0.98445(18)	0.4255(2)	0.0652(8)
C12	0.2084(4)	0.02703(18)	0.46021(17)	0.0608(8)
C13	0.3348(3)	0.38204(15)	0.38412(13)	0.0411(6)
C14	0.4514(3)	0.52415(15)	0.40908(12)	0.0392(5)
C15	0.5985(3)	0.56472(16)	0.41149(14)	0.0451(6)
C16	0.6081(3)	0.65029(17)	0.42117(15)	0.0520(7)
C17	0.4725(4)	0.69672(17)	0.42946(15)	0.0530(7)
C18	0.3273(3)	0.65832(16)	0.42929(14)	0.0468(6)
C19	0.3180(3)	0.57098(16)	0.41942(13)	0.0433(6)
C20	0.8757(4)	0.5477(3)	0.4045(3)	0.0830(11)
C21	0.1803(4)	0.70903(19)	0.4394(2)	0.0659(8)
C22	0.5443(3)	0.27650(16)	0.36937(15)	0.0454(6)
C23	0.6358(3)	0.30644(15)	0.30447(15)	0.0453(6)
C24	0.7924(3)	0.33020(19)	0.31714(17)	0.0561(7)
C25	0.8798(4)	0.3545(2)	0.25815(19)	0.0646(8)
C26	0.8111(4)	0.35490(19)	0.18602(18)	0.0622(8)
C27	0.6553(4)	0.33217(19)	0.17291(17)	0.0581(7)
C28	0.5672(3)	0.30840(17)	0.23149(15)	0.0519(7)

Table S12. Bond lengths (Å) for *anti*-**21**.

N1-C13	1.378(3)	N1-C22	1.452(3)
N1-C8	1.468(3)	N2-C13	1.368(3)
N2-C14	1.400(3)	N2-H01	0.87(4)
O1-C11	1.222(4)	O2-C13	1.227(3)
O3-C15	1.367(3)	O3-C20	1.417(3)
C1-C2	1.523(4)	C1-C9	1.530(3)
C1-C12	1.548(4)	C1-C6	1.550(3)

C2-C3	1.510(5)	C2-H2A	0.98
C2-H2B	0.98	C3-C4	1.524(5)
C3-H3A	0.98	C3-H3B	0.98
C4-C5	1.519(6)	C4-H4A	0.98
C4-H4B	0.98	C5-C6	1.508(4)
C5-H5A	0.98	C5-H5B	0.98
C6-C7	1.548(4)	C6-C10	1.559(5)
C7-C8	1.549(4)	C7-H7A	0.98
C7-H7B	0.98	C8-C9	1.538(4)
C8-H8	0.99	C9-H9A	0.98
C9-H9B	0.98	C10-C11	1.514(5)
C10-H10A	0.98	C10-H10B	0.98
C11-C12	1.495(4)	C12-H12A	0.98
C12-H12B	0.98	C14-C19	1.381(3)
C14-C15	1.406(3)	C15-C16	1.374(4)
C16-C17	1.387(4)	C16-H16	0.94
C17-C18	1.378(4)	C17-H17	0.94
C18-C19	1.402(4)	C18-C21	1.510(4)
C19-H19	0.94	C20-H20A	0.97
C20-H20B	0.97	C20-H20C	0.97
C21-H21A	0.97	C21-H21B	0.97
C21-H21C	0.97	C22-C23	1.520(4)
C22-H22A	0.98	C22-H22B	0.98
C23-C24	1.389(4)	C23-C28	1.391(4)
C24-C25	1.389(4)	C24-H24	0.94
C25-C26	1.377(5)	C25-H25	0.94
C26-C27	1.378(4)	C26-H26	0.94
C27-C28	1.384(4)	C27-H27	0.94
C28-H28	0.94		

Table S13. Bond angles (°) for *anti*-**21.**

C13-N1-C22	122.1(2)	C13-N1-C8	117.0(2)
C22-N1-C8	118.8(2)	C13-N2-C14	128.8(2)
C13-N2-H01	118.(2)	C14-N2-H01	112.(2)
C15-O3-C20	117.9(2)	C2-C1-C9	110.0(2)
C2-C1-C12	112.9(2)	C9-C1-C12	112.6(2)
C2-C1-C6	114.6(2)	C9-C1-C6	102.0(2)
C12-C1-C6	104.2(2)	C3-C2-C1	113.6(3)
C3-C2-H2A	108.8	C1-C2-H2A	108.8
C3-C2-H2B	108.8	C1-C2-H2B	108.8
H2A-C2-H2B	107.7	C2-C3-C4	110.5(3)
C2-C3-H3A	109.5	C4-C3-H3A	109.5
C2-C3-H3B	109.5	C4-C3-H3B	109.5
H3A-C3-H3B	108.1	C5-C4-C3	111.4(3)
C5-C4-H4A	109.3	C3-C4-H4A	109.3
C5-C4-H4B	109.3	C3-C4-H4B	109.3
H4A-C4-H4B	108.0	C6-C5-C4	111.4(3)
C6-C5-H5A	109.3	C4-C5-H5A	109.3
C6-C5-H5B	109.3	C4-C5-H5B	109.3
H5A-C5-H5B	108.0	C5-C6-C7	115.1(3)
C5-C6-C1	113.5(2)	C7-C6-C1	105.4(2)
C5-C6-C10	106.3(3)	C7-C6-C10	112.6(3)
C1-C6-C10	103.4(2)	C6-C7-C8	106.0(2)
C6-C7-H7A	110.5	C8-C7-H7A	110.5
C6-C7-H7B	110.5	C8-C7-H7B	110.5
H7A-C7-H7B	108.7	N1-C8-C9	114.2(2)
N1-C8-C7	115.1(2)	C9-C8-C7	104.8(2)
N1-C8-H8	107.4	C9-C8-H8	107.4
C7-C8-H8	107.4	C1-C9-C8	103.6(2)
C1-C9-H9A	111.0	C8-C9-H9A	111.0
C1-C9-H9B	111.0	C8-C9-H9B	111.0
H9A-C9-H9B	109.0	C11-C10-C6	104.5(3)
C11-C10-H10A	110.8	C6-C10-H10A	110.8
C11-C10-H10B	110.8	C6-C10-H10B	110.8
H10A-C10-H10B	108.9	O1-C11-C12	126.2(3)
O1-C11-C10	124.9(3)	C12-C11-C10	108.9(3)
C11-C12-C1	107.1(2)	C11-C12-H12A	110.3
C1-C12-H12A	110.3	C11-C12-H12B	110.3
C1-C12-H12B	110.3	H12A-C12-H12B	108.6
O2-C13-N2	122.6(2)	O2-C13-N1	123.1(2)
N2-C13-N1	114.3(2)	C19-C14-N2	126.3(2)

C19-C14-C15	119.1(2)	N2-C14-C15	114.5(2)
O3-C15-C16	125.6(2)	O3-C15-C14	114.2(2)
C16-C15-C14	120.2(2)	C15-C16-C17	119.9(2)
C15-C16-H16	120.0	C17-C16-H16	120.0
C18-C17-C16	121.1(2)	C18-C17-H17	119.5
C16-C17-H17	119.5	C17-C18-C19	118.8(2)
C17-C18-C21	120.9(2)	C19-C18-C21	120.3(3)
C14-C19-C18	120.8(2)	C14-C19-H19	119.6
C18-C19-H19	119.6	O3-C20-H20A	109.5
O3-C20-H20B	109.5	H20A-C20-H20B	109.5
O3-C20-H20C	109.5	H20A-C20-H20C	109.5
H20B-C20-H20C	109.5	C18-C21-H21A	109.5
C18-C21-H21B	109.5	H21A-C21-H21B	109.5
C18-C21-H21C	109.5	H21A-C21-H21C	109.5
H21B-C21-H21C	109.5	N1-C22-C23	115.4(2)
N1-C22-H22A	108.4	C23-C22-H22A	108.4
N1-C22-H22B	108.4	C23-C22-H22B	108.4
H22A-C22-H22B	107.5	C24-C23-C28	118.7(3)
C24-C23-C22	120.0(2)	C28-C23-C22	121.4(2)
C25-C24-C23	120.8(3)	C25-C24-H24	119.6
C23-C24-H24	119.6	C26-C25-C24	119.9(3)
C26-C25-H25	120.1	C24-C25-H25	120.1
C25-C26-C27	119.8(3)	C25-C26-H26	120.1
C27-C26-H26	120.1	C26-C27-C28	120.7(3)
C26-C27-H27	119.7	C28-C27-H27	119.7
C27-C28-C23	120.2(3)	C27-C28-H28	119.9
C23-C28-H28	119.9		

Table S14. Torsion angles (°) for *anti*-**21**.

C9-C1-C2-C3	158.3(3)	C12-C1-C2-C3	-75.1(3)
C6-C1-C2-C3	44.0(3)	C1-C2-C3-C4	-52.0(4)
C2-C3-C4-C5	59.4(5)	C3-C4-C5-C6	-58.3(4)
C4-C5-C6-C7	-72.5(3)	C4-C5-C6-C1	49.1(4)
C4-C5-C6-C10	162.0(3)	C2-C1-C6-C5	-42.4(4)
C9-C1-C6-C5	-161.2(3)	C12-C1-C6-C5	81.5(3)
C2-C1-C6-C7	84.5(3)	C9-C1-C6-C7	-34.3(3)
C12-C1-C6-C7	-151.7(3)	C2-C1-C6-C10	-157.1(2)
C9-C1-C6-C10	84.1(3)	C12-C1-C6-C10	-33.2(3)
C5-C6-C7-C8	138.7(3)	C1-C6-C7-C8	12.8(3)
C10-C6-C7-C8	-99.2(3)	C13-N1-C8-C9	-98.1(3)

C22-N1-C8-C9	65.8(3)	C13-N1-C8-C7	140.6(2)
C22-N1-C8-C7	-55.5(3)	C6-C7-C8-N1	139.9(3)
C6-C7-C8-C9	13.6(3)	C2-C1-C9-C8	-79.1(3)
C12-C1-C9-C8	154.0(2)	C6-C1-C9-C8	42.9(3)
N1-C8-C9-C1	-162.2(2)	C7-C8-C9-C1	-35.3(3)
C5-C6-C10-C11	-86.9(3)	C7-C6-C10-C11	146.1(3)
C1-C6-C10-C11	32.9(3)	C6-C10-C11-O1	161.6(3)
C6-C10-C11-C12	-20.1(4)	O1-C11-C12-C1	177.4(3)
C10-C11-C12-C1	-0.9(4)	C2-C1-C12-C11	146.5(3)
C9-C1-C12-C11	-88.2(3)	C6-C1-C12-C11	21.5(3)
C14-N2-C13-O2	-3.6(4)	C14-N2-C13-N1	175.3(2)
C22-N1-C13-O2	-173.0(2)	C8-N1-C13-O2	-9.7(4)
C22-N1-C13-N2	8.1(3)	C8-N1-C13-N2	171.3(2)
C13-N2-C14-C19	4.7(4)	C13-N2-C14-C15	-174.7(2)
C20-O3-C15-C16	2.9(4)	C20-O3-C15-C14	-178.9(3)
C19-C14-C15-O3	179.2(2)	N2-C14-C15-O3	-1.4(3)
C19-C14-C15-C16	-2.5(4)	N2-C14-C15-C16	177.0(2)
O3-C15-C16-C17	178.9(2)	C14-C15-C16-C17	0.8(4)
C15-C16-C17-C18	1.0(4)	C16-C17-C18-C19	-0.9(4)
C16-C17-C18-C21	179.2(3)	N2-C14-C19-C18	-176.9(2)
C15-C14-C19-C18	2.5(4)	C17-C18-C19-C14	-0.8(4)
C21-C18-C19-C14	179.1(2)	C13-N1-C22-C23	-76.9(3)
C8-N1-C22-C23	120.2(2)	N1-C22-C23-C24	143.3(2)
N1-C22-C23-C28	-38.6(3)	C28-C23-C24-C25	-0.7(4)
C22-C23-C24-C25	177.4(3)	C23-C24-C25-C26	-0.3(5)
C24-C25-C26-C27	0.8(5)	C25-C26-C27-C28	-0.4(5)
C26-C27-C28-C23	-0.6(4)	C24-C23-C28-C27	1.2(4)
C22-C23-C28-C27	-176.9(2)		

Table 15. Anisotropic atomic displacement parameters (Å^2) for *anti*-**21**.

The anisotropic atomic displacement factor exponent takes the form: $-2\pi^2[h^2 a^{*2} U_{11} + ... + 2 h k a^* b^* U_{12}]$

	U_{11}	U_{22}	U_{33}	U_{23}	U_{13}	U_{12}
N1	0.0455(11)	0.0352(10)	0.0496(12)	-0.0023(9)	0.0091(9)	-0.0064(9)
N2	0.0393(12)	0.0352(11)	0.0526(12)	-0.0036(9)	0.0042(9)	-0.0040(9)
O1	0.107(2)	0.0619(15)	0.121(2)	0.0118(14)	0.0112(17)	0.0179(14)
O2	0.0411(10)	0.0447(10)	0.0768(13)	-0.0103(9)	0.0053(9)	-0.0048(8)
O3	0.0401(10)	0.0526(11)	0.0782(14)	-0.0110(9)	0.0092(9)	-0.0077(8)

C1	0.0562(15)	0.0382(13)	0.0421(13)	-0.0037(10)	0.0141(11)	-0.0065(11)
C2	0.0612(17)	0.0529(16)	0.0651(18)	-0.0018(13)	0.0156(14)	-0.0097(13)
C3	0.065(2)	0.102(3)	0.082(2)	0.007(2)	0.0047(17)	-0.029(2)
C4	0.106(3)	0.106(3)	0.057(2)	0.0044(19)	-0.0087(19)	-0.042(2)
C5	0.113(3)	0.067(2)	0.0508(17)	-0.0112(15)	0.0190(17)	-0.038(2)
C6	0.088(2)	0.0478(15)	0.0492(15)	-0.0108(12)	0.0270(15)	-0.0236(15)
C7	0.079(2)	0.0472(15)	0.0434(14)	-0.0047(11)	0.0148(13)	-0.0201(14)
C8	0.0539(15)	0.0390(13)	0.0471(14)	-0.0030(11)	0.0090(11)	-0.0117(11)
C9	0.0616(16)	0.0449(14)	0.0436(14)	-0.0054(11)	0.0122(12)	-0.0092(12)
C10	0.083(2)	0.0538(18)	0.088(2)	-0.0078(16)	0.0340(19)	0.0024(16)
C11	0.078(2)	0.0391(15)	0.079(2)	0.0049(14)	0.0108(16)	0.0021(14)
C12	0.077(2)	0.0481(16)	0.0589(17)	0.0057(13)	0.0161(15)	-0.0035(14)
C13	0.0418(13)	0.0379(13)	0.0437(13)	-0.0012(10)	0.0038(10)	-0.0044(10)
C14	0.0450(13)	0.0371(12)	0.0353(12)	-0.0008(9)	0.0007(10)	-0.0041(10)
C15	0.0462(14)	0.0440(14)	0.0454(14)	-0.0039(11)	0.0060(11)	-0.0079(11)
C16	0.0563(16)	0.0453(15)	0.0551(16)	-0.0050(12)	0.0080(12)	-0.0151(12)
C17	0.0701(18)	0.0368(13)	0.0519(15)	-0.0038(11)	0.0036(13)	-0.0087(12)
C18	0.0567(15)	0.0421(13)	0.0409(13)	-0.0040(10)	-0.0010(11)	0.0012(11)
C19	0.0446(13)	0.0427(13)	0.0421(13)	-0.0020(10)	-0.0008(10)	-0.0033(10)
C20	0.0437(17)	0.079(2)	0.127(3)	-0.018(2)	0.0117(18)	-0.0168(16)
C21	0.0664(19)	0.0491(16)	0.081(2)	-0.0100(15)	-0.0049(16)	0.0104(14)
C22	0.0490(14)	0.0383(13)	0.0497(14)	0.0025(10)	0.0085(11)	0.0024(11)
C23	0.0494(14)	0.0365(13)	0.0509(15)	-0.0009(10)	0.0105(11)	0.0015(11)
C24	0.0516(16)	0.0569(16)	0.0597(17)	0.0029(13)	0.0034(13)	0.0006(13)
C25	0.0487(16)	0.070(2)	0.077(2)	0.0045(16)	0.0156(14)	-0.0040(14)
C26	0.0689(19)	0.0568(17)	0.0643(19)	0.0009(14)	0.0281(15)	-0.0016(14)
C27	0.0718(19)	0.0548(16)	0.0491(16)	-0.0010(12)	0.0135(13)	-0.0084(14)
C28	0.0577(16)	0.0473(15)	0.0510(15)	-0.0015(12)	0.0072(12)	-0.0095(12)

Table S16. Hydrogen atomic coordinates and isotropic atomic displacement parameters (Å^2) for *anti*-**21**.

	x/a	y/b	z/c	U(eq)
H01	0.551(4)	0.421(2)	0.3869(19)	0.068(10)
H2A	-0.0477	0.1222	0.4589	0.071
H2B	-0.0284	0.1824	0.3897	0.071
H3A	-0.2203	0.0861	0.3533	0.099
H3B	-0.1108	0.0098	0.3810	0.099
H4A	-0.1138	0.0222	0.2493	0.108
H4B	-0.0522	0.1160	0.2574	0.108
H5A	0.1251	-0.0283	0.3061	0.092
H5B	0.1592	0.0268	0.2352	0.092
H7A	0.1870	0.1850	0.2588	0.067
H7B	0.3699	0.1711	0.2807	0.067
H8	0.1545	0.2648	0.3578	0.056
H9A	0.3665	0.1680	0.4543	0.06
H9B	0.2087	0.2134	0.4759	0.06
H10A	0.4028	-0.0058	0.3155	0.088
H10B	0.4708	0.0697	0.3669	0.088
H12A	0.2405	0.0441	0.5117	0.073
H12B	0.1182	-0.0112	0.4613	0.073
H16	0.7065	0.6773	0.4222	0.062
H17	0.4797	0.7554	0.4353	0.064
H19	0.2198	0.5440	0.4198	0.052
H20A	0.8830	0.5864	0.3630	0.124
H20B	0.9530	0.5033	0.4012	0.124
H20C	0.8959	0.5777	0.4515	0.124
H21A	0.2079	0.7679	0.4458	0.099
H21B	0.1310	0.6892	0.4833	0.099
H21C	0.1074	0.7026	0.3955	0.099
H22A	0.5492	0.2150	0.3712	0.055
H22B	0.5969	0.2978	0.4161	0.055
H24	0.8400	0.3298	0.3662	0.067
H25	0.9857	0.3707	0.2674	0.078
H26	0.8703	0.3706	0.1459	0.075
H27	0.6084	0.3328	0.1237	0.07
H28	0.4607	0.2935	0.2219	0.062

Table S17. Hydrogen bond distances (Å) and angles (°) for *anti*-**21**.

	Donor-H	Acceptor-H	Donor-Acceptor	Angle
N2-H01···O3	0.87(4)	2.06(3)	2.549(3)	114.(3)

3.4. X-ray crystal structure of *syn,anti*-22

Compound *syn,anti*-**22** was crystallized from EtOAc. Colorless crystals. Code DAN6941.

X-ray crystal structure analysis of syn,anti-**22** *(dan6941):* A colorless plate-like specimen of $C_{28}H_{36}N_2O_3$, approximate dimensions 0.150 mm x 0.170 mm x 0.270 mm, was used for the X-ray crystallographic analysis. The X-ray intensity data were measured.

The integration of the data using a monoclinic unit cell yielded a total of 4152 reflections to a maximum θ angle of 67.18° (0.84 Å resolution), of which 4152 were independent (average redundancy 1.000, completeness = 96.1%, R_{sig} = 2.57%) and 3721 (89.62%) were greater than $2\sigma(F^2)$.

The final cell constants of a = 7.5886(3) Å, b = 16.0072(4) Å, c = 20.2378(5) Å, β = 100.377(2)°, volume = 2418.12(13) $Å^3$, are based upon the refinement of the XYZ-centroids of reflections above 20 σ(I). Data were corrected for absorption effects using the multi-scan method (SADABS). The calculated minimum and maximum transmission coefficients (based on crystal size) are 0.8490 and 0.9120.

The structure was solved and refined using the Bruker SHELXTL Software Package, using the space group P 1 21/c 1, with Z = 4 for the formula unit, $C_{28}H_{36}N_2O_3$. The final anisotropic full-matrix least-squares refinement on F^2 with 307 variables converged at R1 = 4.36%, for the observed data and wR2 = 11.81% for all data. The goodness-of-fit was 1.031. The largest peak in the final difference electron density synthesis was 0.175 $e^-/Å^3$ and the largest hole was -0.164 $e^-/Å^3$ with an RMS deviation of 0.032 $e^-/Å^3$. On the basis of the final model, the calculated density was 1.232 g/cm^3 and F(000), 968 e^-. CCDC number: 2073468.

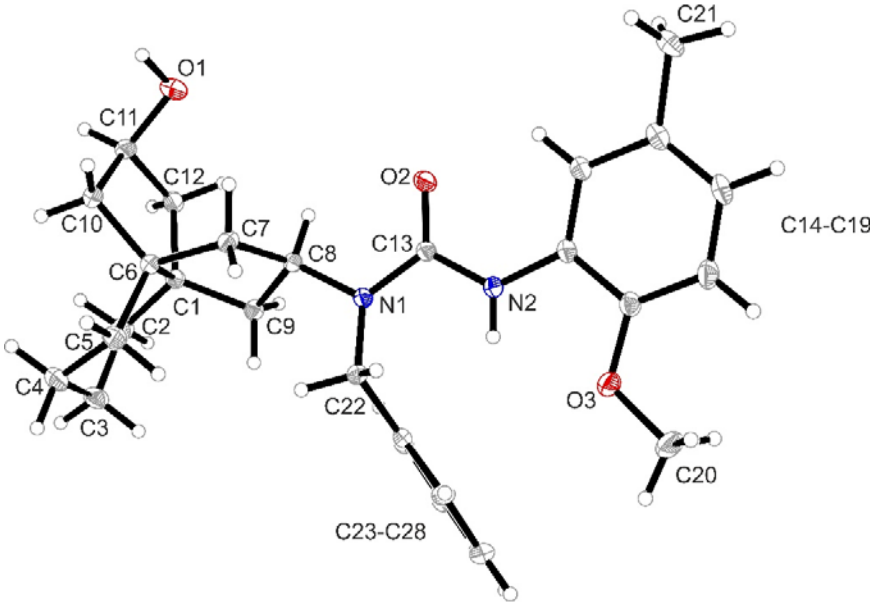

Table S18. Sample and crystal data for *syn,anti*-**22**.

Identification code	dan6941	
Chemical formula	$C_{28}H_{36}N_2O_3$	
Formula weight	448.59 g/mol	
Temperature	223(2) K	
Wavelength	1.54178 Å	
Crystal size	0.150 x 0.170 x 0.270 mm	
Crystal habit	colorless plate	
Crystal system	monoclinic	
Space group	P 1 21/c 1	
Unit cell dimensions	a = 7.5886(3) Å	α = 90°
	b = 16.0072(4) Å	β = 100.377(2)°
	c = 20.2378(5) Å	γ = 90°
Volume	2418.12(13) Å^3	
Z	4	
Density (calculated)	1.232 g/cm^3	
Absorption coefficient	0.628 mm^{-1}	
F(000)	968	

Table S19. Data collection and structure refinement for *syn,anti*-**22**.

Theta range for data collection	5.23 to 67.18°
Reflections collected	4152
Absorption correction	multi-scan
Max. and min. transmission	0.9120 and 0.8490
Structure solution technique	direct methods
Structure solution program	SHELXL-2014/7 (Sheldrick, 2014)
Refinement method	Full-matrix least-squares on F^2
Refinement program	SHELXL-2014/7 (Sheldrick, 2014)
Function minimized	$\Sigma\ w(F_o^2 - F_c^2)^2$
Data / restraints / parameters	4152 / 0 / 307
Goodness-of-fit on F^2	1.031
Final R indices	3721 data; I>2σ(I) R1 = 0.0436, wR2 = 0.1133
	all data R1 = 0.0482, wR2 = 0.1181
Weighting scheme	$w=1/[\sigma^2(F_o^2)+(0.0599P)^2+0.6637P]$ where $P=(F_o^2+2F_c^2)/3$
Largest diff. peak and hole	0.175 and -0.164 eÅ^{-3}
R.M.S. deviation from mean	0.032 eÅ^{-3}

Table S20. Atomic coordinates and equivalent isotropic atomic displacement parameters (Å^2) for *syn,anti*-**22**.

U(eq) is defined as one third of the trace of the orthogonalized U_{ij} tensor.

	x/a	y/b	z/c	U(eq)
N1	0.52565(15)	0.45528(7)	0.36735(6)	0.0391(3)
N2	0.45828(18)	0.31450(8)	0.36886(6)	0.0438(3)
O1	0.10164(18)	0.59954(8)	0.46807(8)	0.0700(4)
O2	0.74762(14)	0.35862(7)	0.39639(6)	0.0522(3)
O3	0.17642(15)	0.22331(7)	0.33623(6)	0.0568(3)
C1	0.73000(18)	0.66687(9)	0.35816(7)	0.0386(3)
C2	0.6781(2)	0.75064(10)	0.32314(9)	0.0527(4)
C3	0.4991(2)	0.78594(11)	0.33289(10)	0.0647(5)
C4	0.4936(3)	0.79321(12)	0.40727(11)	0.0659(5)

C5	0.5204(2)	0.70788(11)	0.44005(8)	0.0520(4)
C6	0.69374(19)	0.66362(9)	0.43156(7)	0.0386(3)
C7	0.6761(2)	0.56898(9)	0.44542(7)	0.0426(3)
C8	0.65820(18)	0.52341(9)	0.37733(7)	0.0385(3)
C9	0.6254(2)	0.59227(9)	0.32397(7)	0.0406(3)
C10	0.8626(2)	0.70253(10)	0.47289(8)	0.0469(4)
C11	0.0186(2)	0.67428(10)	0.43986(9)	0.0510(4)
C12	0.9360(2)	0.65542(10)	0.36673(8)	0.0474(4)
C13	0.58681(19)	0.37553(9)	0.37880(7)	0.0388(3)
C14	0.4794(2)	0.22892(9)	0.38484(7)	0.0406(3)
C15	0.3246(2)	0.18021(10)	0.36740(7)	0.0461(4)
C16	0.3298(3)	0.09611(10)	0.38245(9)	0.0584(4)
C17	0.4869(3)	0.06016(10)	0.41626(9)	0.0589(4)
C18	0.6390(2)	0.10712(10)	0.43552(8)	0.0494(4)
C19	0.6347(2)	0.19194(9)	0.41877(7)	0.0445(3)
C20	0.0111(3)	0.17978(14)	0.32061(11)	0.0731(6)
C21	0.8080(3)	0.07034(11)	0.47615(9)	0.0639(5)
C22	0.33739(18)	0.47587(9)	0.36664(7)	0.0400(3)
C23	0.20999(19)	0.45528(9)	0.30225(7)	0.0402(3)
C24	0.2517(2)	0.46987(10)	0.23931(8)	0.0502(4)
C25	0.1275(3)	0.45204(11)	0.18211(9)	0.0638(5)
C26	0.9620(3)	0.41952(12)	0.18715(11)	0.0712(6)
C27	0.9204(2)	0.40490(12)	0.24914(11)	0.0674(5)
C28	0.0438(2)	0.42204(10)	0.30640(9)	0.0522(4)

Table S21. Bond lengths (Å) for *syn,anti*-**22.**

N1-C13	1.3638(18)	N1-C22	1.4637(17)
N1-C8	1.4726(17)	N2-C13	1.3696(19)
N2-C14	1.4099(19)	N2-H02	0.84(2)
O1-C11	1.423(2)	O1-H01	0.97(2)
O2-C13	1.2380(17)	O3-C15	1.3724(19)
O3-C20	1.420(2)	C1-C9	1.529(2)

C1-C2	1.535(2)	C1-C12	1.552(2)
C1-C6	1.5594(19)	C2-C3	1.517(2)
C2-H2A	0.98	C2-H2B	0.98
C3-C4	1.518(3)	C3-H3A	0.98
C3-H3B	0.98	C4-C5	1.516(3)
C4-H4A	0.98	C4-H4B	0.98
C5-C6	1.531(2)	C5-H5A	0.98
C5-H5B	0.98	C6-C10	1.531(2)
C6-C7	1.551(2)	C7-C8	1.543(2)
C7-H7A	0.98	C7-H7B	0.98
C8-C9	1.5317(19)	C8-H8	0.99
C9-H9A	0.98	C9-H9B	0.98
C10-C11	1.529(2)	C10-H10A	0.98
C10-H10B	0.98	C11-C12	1.530(2)
C11-H11	0.99	C12-H12A	0.98
C12-H12B	0.98	C14-C19	1.385(2)
C14-C15	1.401(2)	C15-C16	1.379(2)
C16-C17	1.389(3)	C16-H16	0.94
C17-C18	1.373(3)	C17-H17	0.94
C18-C19	1.398(2)	C18-C21	1.512(2)
C19-H19	0.94	C20-H20A	0.97
C20-H20B	0.97	C20-H20C	0.97
C21-H21A	0.97	C21-H21B	0.97
C21-H21C	0.97	C22-C23	1.5125(19)
C22-H22A	0.98	C22-H22B	0.98
C23-C28	1.386(2)	C23-C24	1.387(2)
C24-C25	1.385(2)	C24-H24	0.94
C25-C26	1.381(3)	C25-H25	0.94
C26-C27	1.368(3)	C26-H26	0.94
C27-C28	1.380(2)	C27-H27	0.94
C28-H28	0.94		

Table S22. Bond angles (°) for *syn,anti*-**22**.

C13-N1-C22	120.93(12)	C13-N1-C8	117.82(11)
C22-N1-C8	118.42(11)	C13-N2-C14	127.66(13)
C13-N2-H02	117.2(13)	C14-N2-H02	115.1(13)
C11-O1-H01	105.5(14)	C15-O3-C20	118.26(14)
C9-C1-C2	113.85(12)	C9-C1-C12	112.87(12)
C2-C1-C12	108.92(12)	C9-C1-C6	103.82(11)
C2-C1-C6	113.23(12)	C12-C1-C6	103.68(11)
C3-C2-C1	115.27(13)	C3-C2-H2A	108.5
C1-C2-H2A	108.5	C3-C2-H2B	108.5
C1-C2-H2B	108.5	H2A-C2-H2B	107.5
C2-C3-C4	110.05(14)	C2-C3-H3A	109.6
C4-C3-H3A	109.6	C2-C3-H3B	109.6
C4-C3-H3B	109.6	H3A-C3-H3B	108.2
C5-C4-C3	109.84(15)	C5-C4-H4A	109.7
C3-C4-H4A	109.7	C5-C4-H4B	109.7
C3-C4-H4B	109.7	H4A-C4-H4B	108.2
C4-C5-C6	114.73(14)	C4-C5-H5A	108.6
C6-C5-H5A	108.6	C4-C5-H5B	108.6
C6-C5-H5B	108.6	H5A-C5-H5B	107.6
C5-C6-C10	113.69(12)	C5-C6-C7	109.14(12)
C10-C6-C7	112.94(12)	C5-C6-C1	113.21(12)
C10-C6-C1	103.48(11)	C7-C6-C1	103.93(11)
C8-C7-C6	107.26(11)	C8-C7-H7A	110.3
C6-C7-H7A	110.3	C8-C7-H7B	110.3
C6-C7-H7B	110.3	H7A-C7-H7B	108.5
N1-C8-C9	114.58(11)	N1-C8-C7	114.86(11)
C9-C8-C7	105.44(11)	N1-C8-H8	107.2
C9-C8-H8	107.2	C7-C8-H8	107.2
C1-C9-C8	103.76(11)	C1-C9-H9A	111.0
C8-C9-H9A	111.0	C1-C9-H9B	111.0
C8-C9-H9B	111.0	H9A-C9-H9B	109.0

C11-C10-C6	106.18(12)	C11-C10-H10A	110.5
C6-C10-H10A	110.5	C11-C10-H10B	110.5
C6-C10-H10B	110.5	H10A-C10-H10B	108.7
O1-C11-C10	113.27(14)	O1-C11-C12	107.28(13)
C10-C11-C12	105.46(12)	O1-C11-H11	110.2
C10-C11-H11	110.2	C12-C11-H11	110.2
C11-C12-C1	108.29(12)	C11-C12-H12A	110.0
C1-C12-H12A	110.0	C11-C12-H12B	110.0
C1-C12-H12B	110.0	H12A-C12-H12B	108.4
O2-C13-N1	122.77(13)	O2-C13-N2	121.68(13)
N1-C13-N2	115.55(12)	C19-C14-C15	119.13(14)
C19-C14-N2	125.46(14)	C15-C14-N2	115.31(13)
O3-C15-C16	125.48(15)	O3-C15-C14	114.61(13)
C16-C15-C14	119.91(15)	C15-C16-C17	120.06(17)
C15-C16-H16	120.0	C17-C16-H16	120.0
C18-C17-C16	121.08(15)	C18-C17-H17	119.5
C16-C17-H17	119.5	C17-C18-C19	118.74(16)
C17-C18-C21	121.87(15)	C19-C18-C21	119.36(16)
C14-C19-C18	121.04(15)	C14-C19-H19	119.5
C18-C19-H19	119.5	O3-C20-H20A	109.5
O3-C20-H20B	109.5	H20A-C20-H20B	109.5
O3-C20-H20C	109.5	H20A-C20-H20C	109.5
H20B-C20-H20C	109.5	C18-C21-H21A	109.5
C18-C21-H21B	109.5	H21A-C21-H21B	109.5
C18-C21-H21C	109.5	H21A-C21-H21C	109.5
H21B-C21-H21C	109.5	N1-C22-C23	115.51(12)
N1-C22-H22A	108.4	C23-C22-H22A	108.4
N1-C22-H22B	108.4	C23-C22-H22B	108.4
H22A-C22-H22B	107.5	C28-C23-C24	118.79(14)
C28-C23-C22	118.62(14)	C24-C23-C22	122.58(14)
C25-C24-C23	119.97(17)	C25-C24-H24	120.0
C23-C24-H24	120.0	C26-C25-C24	120.50(18)

C26-C25-H25	119.8	C24-C25-H25	119.8
C27-C26-C25	119.72(17)	C27-C26-H26	120.1
C25-C26-H26	120.1	C26-C27-C28	120.16(19)
C26-C27-H27	119.9	C28-C27-H27	119.9
C27-C28-C23	120.86(18)	C27-C28-H28	119.6
C23-C28-H28	119.6		

Table S23. Torsion angles (°) for *syn,anti*-**22**.

C9-C1-C2-C3	74.08(18)	C12-C1-C2-C3	-158.99(14)
C6-C1-C2-C3	-44.20(19)	C1-C2-C3-C4	55.0(2)
C2-C3-C4-C5	-59.5(2)	C3-C4-C5-C6	56.9(2)
C4-C5-C6-C10	71.62(18)	C4-C5-C6-C7	-161.31(14)
C4-C5-C6-C1	-46.09(19)	C9-C1-C6-C5	-85.61(14)
C2-C1-C6-C5	38.35(17)	C12-C1-C6-C5	156.23(12)
C9-C1-C6-C10	150.86(12)	C2-C1-C6-C10	-85.18(14)
C12-C1-C6-C10	32.71(14)	C9-C1-C6-C7	32.67(14)
C2-C1-C6-C7	156.63(12)	C12-C1-C6-C7	-85.48(13)
C5-C6-C7-C8	108.26(13)	C10-C6-C7-C8	-124.24(13)
C1-C6-C7-C8	-12.80(14)	C13-N1-C8-C9	138.94(13)
C22-N1-C8-C9	-59.89(16)	C13-N1-C8-C7	-98.74(15)
C22-N1-C8-C7	62.44(16)	C6-C7-C8-N1	-138.91(12)
C6-C7-C8-C9	-11.77(15)	C2-C1-C9-C8	-163.97(12)
C12-C1-C9-C8	71.19(14)	C6-C1-C9-C8	-40.41(14)
N1-C8-C9-C1	159.57(11)	C7-C8-C9-C1	32.27(14)
C5-C6-C10-C11	-159.30(13)	C7-C6-C10-C11	75.63(15)
C1-C6-C10-C11	-36.09(15)	C6-C10-C11-O1	-92.18(15)
C6-C10-C11-C12	24.84(16)	O1-C11-C12-C1	117.24(14)
C10-C11-C12-C1	-3.77(16)	C9-C1-C12-C11	-129.71(13)
C2-C1-C12-C11	102.81(14)	C6-C1-C12-C11	-18.03(15)
C22-N1-C13-O2	-161.40(13)	C8-N1-C13-O2	-0.7(2)
C22-N1-C13-N2	19.27(19)	C8-N1-C13-N2	179.96(12)
C14-N2-C13-O2	9.4(2)	C14-N2-C13-N1	-171.28(13)

C13-N2-C14-C19	5.2(2)	C13-N2-C14-C15	-178.46(14)
C20-O3-C15-C16	3.2(2)	C20-O3-C15-C14	-176.14(15)
C19-C14-C15-O3	177.65(13)	N2-C14-C15-O3	1.10(19)
C19-C14-C15-C16	-1.7(2)	N2-C14-C15-C16	-178.30(14)
O3-C15-C16-C17	-177.81(16)	C14-C15-C16-C17	1.5(3)
C15-C16-C17-C18	0.3(3)	C16-C17-C18-C19	-1.8(3)
C16-C17-C18-C21	176.10(16)	C15-C14-C19-C18	0.2(2)
N2-C14-C19-C18	176.37(14)	C17-C18-C19-C14	1.6(2)
C21-C18-C19-C14	-176.40(14)	C13-N1-C22-C23	-80.06(17)
C8-N1-C22-C23	119.37(14)	N1-C22-C23-C28	139.71(14)
N1-C22-C23-C24	-41.55(19)	C28-C23-C24-C25	0.7(2)
C22-C23-C24-C25	-178.06(14)	C23-C24-C25-C26	-0.2(3)
C24-C25-C26-C27	0.1(3)	C25-C26-C27-C28	-0.4(3)
C26-C27-C28-C23	0.9(3)	C24-C23-C28-C27	-1.1(2)
C22-C23-C28-C27	177.73(15)		

Table S24. Anisotropic atomic displacement parameters (Å^2) for *syn,anti*-**22**.
The anisotropic atomic displacement factor exponent takes the form: $-2\pi^2[h^2 a^{*2} U_{11} + ... + 2 h k a^* b^* U_{12}]$

	U_{11}	U_{22}	U_{33}	U_{23}	U_{13}	U_{12}
N1	0.0339(6)	0.0321(6)	0.0507(7)	0.0004(5)	0.0057(5)	-0.0001(5)
N2	0.0410(7)	0.0327(6)	0.0543(7)	0.0053(5)	-0.0007(6)	-0.0021(5)
O1	0.0562(8)	0.0626(8)	0.0850(9)	0.0073(7)	-0.0041(6)	0.0176(6)
O2	0.0376(6)	0.0411(6)	0.0768(7)	0.0099(5)	0.0074(5)	0.0026(5)
O3	0.0481(6)	0.0481(7)	0.0703(7)	0.0097(5)	0.0001(5)	-0.0110(5)
C1	0.0362(7)	0.0358(7)	0.0435(7)	0.0031(6)	0.0068(6)	-0.0013(6)
C2	0.0550(10)	0.0418(9)	0.0597(9)	0.0129(7)	0.0060(7)	-0.0024(7)
C3	0.0549(10)	0.0447(10)	0.0892(13)	0.0182(9)	-0.0015(9)	0.0072(8)
C4	0.0523(10)	0.0509(10)	0.0946(14)	-0.0055(9)	0.0131(9)	0.0143(8)
C5	0.0456(9)	0.0541(10)	0.0581(9)	-0.0058(7)	0.0144(7)	0.0057(7)
C6	0.0362(7)	0.0368(8)	0.0419(7)	-0.0015(6)	0.0045(6)	-0.0008(6)
C7	0.0440(8)	0.0429(8)	0.0392(7)	0.0038(6)	0.0027(6)	-0.0063(6)

C8	0.0338(7)	0.0344(7)	0.0465(8)	0.0015(6)	0.0052(6)	-0.0025(6)
C9	0.0434(8)	0.0398(8)	0.0385(7)	0.0009(6)	0.0073(6)	-0.0035(6)
C10	0.0463(9)	0.0408(8)	0.0504(8)	-0.0045(6)	-0.0003(6)	-0.0029(7)
C11	0.0368(8)	0.0428(8)	0.0698(10)	0.0023(7)	0.0001(7)	-0.0029(6)
C12	0.0405(8)	0.0426(8)	0.0613(9)	-0.0002(7)	0.0147(7)	-0.0020(6)
C13	0.0381(8)	0.0355(7)	0.0427(7)	0.0023(6)	0.0070(6)	0.0004(6)
C14	0.0498(8)	0.0329(7)	0.0397(7)	0.0007(6)	0.0098(6)	-0.0015(6)
C15	0.0527(9)	0.0395(8)	0.0456(8)	0.0032(6)	0.0079(7)	-0.0038(7)
C16	0.0687(11)	0.0410(9)	0.0653(10)	0.0030(8)	0.0110(8)	-0.0124(8)
C17	0.0797(12)	0.0330(8)	0.0659(10)	0.0076(7)	0.0180(9)	0.0018(8)
C18	0.0655(10)	0.0395(8)	0.0454(8)	0.0031(6)	0.0159(7)	0.0086(7)
C19	0.0516(9)	0.0383(8)	0.0437(8)	0.0007(6)	0.0091(6)	0.0029(7)
C20	0.0522(11)	0.0715(13)	0.0916(14)	0.0136(11)	0.0018(9)	-0.0200(9)
C21	0.0778(13)	0.0504(10)	0.0636(11)	0.0114(8)	0.0134(9)	0.0207(9)
C22	0.0344(7)	0.0391(8)	0.0464(8)	-0.0034(6)	0.0073(6)	-0.0007(6)
C23	0.0374(7)	0.0318(7)	0.0498(8)	-0.0028(6)	0.0034(6)	0.0051(6)
C24	0.0594(10)	0.0393(8)	0.0505(9)	-0.0009(7)	0.0064(7)	0.0017(7)
C25	0.0917(14)	0.0448(9)	0.0482(9)	-0.0018(7)	-0.0054(9)	0.0105(9)
C26	0.0731(13)	0.0485(10)	0.0756(13)	-0.0118(9)	-0.0305(10)	0.0126(9)
C27	0.0432(9)	0.0602(11)	0.0901(14)	-0.0088(10)	-0.0115(9)	0.0017(8)
C28	0.0382(8)	0.0497(9)	0.0667(10)	-0.0033(8)	0.0043(7)	0.0004(7)

Table S25. Hydrogen atomic coordinates and isotropic atomic displacement parameters (Å^2) for *syn,anti*-**22**.

	x/a	y/b	z/c	U(eq)
H02	0.355(3)	0.3287(12)	0.3503(9)	0.056(5)
H01	1.145(3)	0.6120(14)	0.5150(12)	0.084
H2A	0.7716	0.7916	0.3397	0.063
H2B	0.6757	0.7436	0.2749	0.063
H3A	0.4022	0.7493	0.3113	0.078
H3B	0.4812	0.8411	0.3118	0.078
H4A	0.5880	0.8313	0.4286	0.079
H4B	0.3778	0.8162	0.4133	0.079

H5A	0.4188	0.6723	0.4211	0.062
H5B	0.5195	0.7141	0.4882	0.062
H7A	0.7821	0.5490	0.4764	0.051
H7B	0.5702	0.5585	0.4657	0.051
H8	0.7763	0.4983	0.3752	0.046
H9A	0.6705	0.5757	0.2835	0.049
H9B	0.4975	0.6053	0.3117	0.049
H10A	0.8534	0.7636	0.4725	0.056
H10B	0.8796	0.6832	0.5196	0.056
H11	1.1085	0.7195	0.4420	0.061
H12A	0.9642	0.5980	0.3554	0.057
H12B	0.9848	0.6935	0.3367	0.057
H16	0.2267	0.0631	0.3698	0.07
H17	0.4893	0.0027	0.4261	0.071
H19	0.7388	0.2244	0.4307	0.053
H20A	0.0218	0.1350	0.2893	0.11
H20B	-0.0824	0.2181	0.3005	0.11
H20C	-0.0190	0.1565	0.3614	0.11
H21A	0.7878	0.0120	0.4856	0.096
H21B	0.8397	0.1007	0.5181	0.096
H21C	0.9047	0.0747	0.4509	0.096
H22A	0.3287	0.5358	0.3754	0.048
H22B	0.2978	0.4460	0.4036	0.048
H24	0.3643	0.4918	0.2355	0.06
H25	0.1561	0.4622	0.1395	0.077
H26	-0.1218	0.4075	0.1482	0.085
H27	-0.1926	0.3831	0.2527	0.081
H28	0.0147	0.4110	0.3488	0.063

Table S26. Hydrogen bond distances (Å) and angles (°) for *syn,anti*-**22**.

	Donor-H	Acceptor-H	Donor-Acceptor	Angle
N2-H02···O3	0.84(2)	2.150(19)	2.5754(16)	111.4(16)

3.5 X-ray crystal structure of *anti*,*syn*-22

Compound *anti,syn*-**22** was crystallized from EtOAc. Colorless crystals. Code DAN6939.

*X-ray crystal structure analysis of anti,syn-***22** *(dan6939):* A colorless needle-like specimen of $C_{28}H_{36}N_2O_3$, approximate dimensions 0.020 mm x 0.070 mm x 0.270 mm, was used for the X-ray crystallographic analysis. The X-ray intensity data were measured.

The integration of the data using a monoclinic unit cell yielded a total of 4164 reflections to a maximum θ angle of 66.92° (0.84 Å resolution), of which 4164 were independent (average redundancy 1.000, completeness = 95.5%, R_{sig} = 3.04%) and 3332 (80.02%) were greater than 2σ(F^2).

The final cell constants of a = 7.9714(2) Å, b = 27.7657(10) Å, c = 11.1029(5) Å, β = 95.285(3)°, volume = 2446.97(15) $Å^3$, are based upon the refinement of the XYZ-centroids of reflections above 20 σ(I). Data were corrected for absorption effects using the multi-scan method (SADABS). The calculated minimum and maximum transmission coefficients (based on crystal size) are 0.8500 and 0.9880.

The structure was solved and refined using the Bruker SHELXTL Software Package, using the space group P 1 21/n 1, with Z = 4 for the formula unit, $C_{28}H_{36}N_2O_3$. The final anisotropic full-matrix least-squares refinement on F^2 with 308 variables converged at R1 = 5.00%, for the observed data and wR2 = 13.22% for all data. The goodness-of-fit was 1.031. The largest peak in the final difference electron density synthesis was 0.131 $e^-/Å^3$ and the largest hole was -0.239 $e^-/Å^3$ with an RMS deviation of 0.044 $e^-/Å^3$. On the basis of the final model, the calculated density was 1.218 g/cm^3 and F(000), 968 e^-. CCDC number: 2073469.

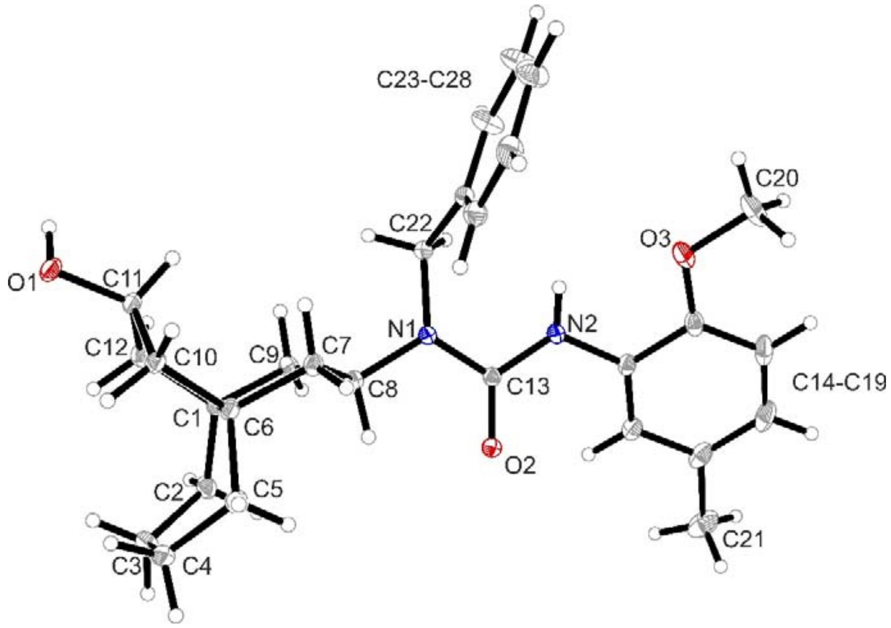

Table S27. Sample and crystal data for *anti,syn*-**22**.

Identification code	dan6939	
Chemical formula	$C_{28}H_{36}N_2O_3$	
Formula weight	448.59 g/mol	
Temperature	223(2) K	
Wavelength	1.54178 Å	
Crystal size	0.020 x 0.070 x 0.270 mm	
Crystal habit	colorless needle	
Crystal system	monoclinic	
Space group	P 1 21/n 1	
Unit cell dimensions	a = 7.9714(2) Å	α = 90°
	b = 27.7657(10) Å	β = 95.285(3)°
	c = 11.1029(5) Å	γ = 90°
Volume	2446.97(15) Å^3	
Z	4	
Density (calculated)	1.218 g/cm^3	
Absorption coefficient	0.621 mm^{-1}	
F(000)	968	

Table S28. Data collection and structure refinement for *anti,syn*-**22**.

Theta range for data collection	5.11 to 66.92°	
Reflections collected	4164	
Absorption correction	multi-scan	
Max. and min. transmission	0.9880 and 0.8500	
Structure solution technique	direct methods	
Structure solution program	SHELXL-2014/7 (Sheldrick, 2014)	
Refinement method	Full-matrix least-squares on F^2	
Refinement program	SHELXL-2014/7 (Sheldrick, 2014)	
Function minimized	$\Sigma\ w(F_o^2 - F_c^2)^2$	
Data / restraints / parameters	4164 / 0 / 308	
Goodness-of-fit on F^2	1.031	
Δ/σ_{max}	0.001	
Final R indices	3332 data; I>2σ(I)	R1 = 0.0500, wR2 = 0.1217
	all data	R1 = 0.0649, wR2 = 0.1322
Weighting scheme	$w=1/[\sigma^2(F_o^2)+(0.0665P)^2+0.8438P]$ where $P=(F_o^2+2F_c^2)/3$	
Largest diff. peak and hole	0.131 and -0.239 eÅ^{-3}	
R.M.S. deviation from mean	0.044 eÅ^{-3}	

Table S29. Atomic coordinates and equivalent isotropic atomic displacement parameters (Å^2) for *anti,syn*-**22**.

U(eq) is defined as one third of the trace of the orthogonalized U_{ij} tensor.

	x/a	y/b	z/c	U(eq)
N1	0.64333(17)	0.66107(5)	0.66179(13)	0.0351(4)
N2	0.7004(2)	0.59301(6)	0.55192(15)	0.0391(4)
O1	0.62123(18)	0.84538(5)	0.00751(13)	0.0483(4)
O2	0.45562(15)	0.63401(5)	0.50966(12)	0.0412(3)
O3	0.83515(19)	0.50887(5)	0.60362(14)	0.0561(4)
C1	0.4674(2)	0.78518(6)	0.71854(16)	0.0339(4)
C2	0.3310(3)	0.80252(8)	0.62167(18)	0.0479(5)
C3	0.1794(3)	0.82490(9)	0.6739(2)	0.0601(6)
C4	0.1015(3)	0.79185(9)	0.7639(2)	0.0589(6)

C5	0.2008(2)	0.74532(7)	0.79040(19)	0.0457(5)
C6	0.3906(2)	0.75337(6)	0.81909(15)	0.0329(4)
C7	0.4856(2)	0.70494(6)	0.81633(16)	0.0355(4)
C8	0.5295(2)	0.70093(6)	0.68618(16)	0.0340(4)
C9	0.5908(2)	0.75160(7)	0.66067(16)	0.0384(4)
C10	0.4347(2)	0.78070(7)	0.93773(16)	0.0365(4)
C11	0.5927(2)	0.80894(6)	0.91786(16)	0.0357(4)
C12	0.5567(2)	0.82729(7)	0.78911(17)	0.0408(4)
C13	0.5914(2)	0.62970(6)	0.57119(15)	0.0313(4)
C14	0.6675(2)	0.55576(6)	0.46606(17)	0.0367(4)
C15	0.7400(2)	0.51084(7)	0.4950(2)	0.0459(5)
C16	0.7147(3)	0.47312(8)	0.4143(3)	0.0626(7)
C17	0.6171(3)	0.47980(9)	0.3065(2)	0.0670(7)
C18	0.5456(3)	0.52383(9)	0.2751(2)	0.0564(6)
C19	0.5736(2)	0.56185(8)	0.35612(17)	0.0438(5)
C20	0.9038(3)	0.46313(9)	0.6420(3)	0.0718(7)
C21	0.4362(3)	0.53119(12)	0.1584(2)	0.0833(9)
C22	0.8179(2)	0.66203(6)	0.71540(17)	0.0368(4)
C23	0.8698(2)	0.62424(6)	0.80943(16)	0.0384(4)
C24	0.0389(3)	0.61220(10)	0.8255(2)	0.0675(7)
C25	0.0983(4)	0.58142(13)	0.9173(3)	0.0949(10)
C26	0.9917(4)	0.56260(10)	0.9939(3)	0.0766(8)
C27	0.8225(4)	0.57336(8)	0.9783(2)	0.0644(6)
C28	0.7620(3)	0.60423(7)	0.8852(2)	0.0504(5)

Table S30. Bond lengths (Å) for *anti,syn*-**22**.

N1-C13	1.366(2)	N1-C22	1.462(2)
N1-C8	1.472(2)	N2-C13	1.369(2)
N2-C14	1.414(2)	N2-H02	0.86(3)
O1-C11	1.423(2)	O1-H01	0.87(3)
O2-C13	1.231(2)	O3-C15	1.365(3)
O3-C20	1.432(2)	C1-C2	1.535(3)

C1-C9	1.538(2)	C1-C12	1.544(2)
C1-C6	1.590(2)	C2-C3	1.520(3)
C2-H2A	0.98	C2-H2B	0.98
C3-C4	1.530(4)	C3-H3A	0.98
C3-H3B	0.98	C4-C5	1.530(3)
C4-H4A	0.98	C4-H4B	0.98
C5-C6	1.534(2)	C5-H5A	0.98
C5-H5B	0.98	C6-C10	1.533(2)
C6-C7	1.545(2)	C7-C8	1.522(2)
C7-H7A	0.98	C7-H7B	0.98
C8-C9	1.524(3)	C8-H8	0.99
C9-H9A	0.98	C9-H9B	0.98
C10-C11	1.517(2)	C10-H10A	0.98
C10-H10B	0.98	C11-C12	1.520(3)
C11-H11	0.99	C12-H12A	0.98
C12-H12B	0.98	C14-C19	1.382(3)
C14-C15	1.400(3)	C15-C16	1.381(3)
C16-C17	1.379(4)	C16-H16	0.94
C17-C18	1.380(4)	C17-H17	0.94
C18-C19	1.392(3)	C18-C21	1.508(4)
C19-H19	0.94	C20-H20A	0.97
C20-H20B	0.97	C20-H20C	0.97
C21-H21A	0.97	C21-H21B	0.97
C21-H21C	0.97	C22-C23	1.511(2)
C22-H22A	0.98	C22-H22B	0.98
C23-C28	1.374(3)	C23-C24	1.384(3)
C24-C25	1.380(3)	C24-H24	0.94
C25-C26	1.361(4)	C25-H25	0.94
C26-C27	1.377(4)	C26-H26	0.94
C27-C28	1.394(3)	C27-H27	0.94
C28-H28	0.94		

Table S31. Bond angles (°) for *anti,syn*-**22**.

C13-N1-C22	121.59(14)	C13-N1-C8	117.70(13)
C22-N1-C8	119.40(14)	C13-N2-C14	124.67(15)
C13-N2-H02	116.8(17)	C14-N2-H02	114.7(17)
C11-O1-H01	105.9(17)	C15-O3-C20	117.67(19)
C2-C1-C9	109.67(15)	C2-C1-C12	112.43(15)
C9-C1-C12	112.86(15)	C2-C1-C6	111.87(15)
C9-C1-C6	105.02(14)	C12-C1-C6	104.69(14)
C3-C2-C1	113.44(17)	C3-C2-H2A	108.9
C1-C2-H2A	108.9	C3-C2-H2B	108.9
C1-C2-H2B	108.9	H2A-C2-H2B	107.7
C2-C3-C4	112.87(18)	C2-C3-H3A	109.0
C4-C3-H3A	109.0	C2-C3-H3B	109.0
C4-C3-H3B	109.0	H3A-C3-H3B	107.8
C5-C4-C3	113.45(18)	C5-C4-H4A	108.9
C3-C4-H4A	108.9	C5-C4-H4B	108.9
C3-C4-H4B	108.9	H4A-C4-H4B	107.7
C4-C5-C6	113.59(16)	C4-C5-H5A	108.8
C6-C5-H5A	108.8	C4-C5-H5B	108.8
C6-C5-H5B	108.8	H5A-C5-H5B	107.7
C10-C6-C5	113.22(14)	C10-C6-C7	111.96(14)
C5-C6-C7	110.19(14)	C10-C6-C1	104.83(14)
C5-C6-C1	111.81(15)	C7-C6-C1	104.39(13)
C8-C7-C6	103.79(14)	C8-C7-H7A	111.0
C6-C7-H7A	111.0	C8-C7-H7B	111.0
C6-C7-H7B	111.0	H7A-C7-H7B	109.0
N1-C8-C7	115.40(15)	N1-C8-C9	116.49(15)
C7-C8-C9	102.40(14)	N1-C8-H8	107.3
C7-C8-H8	107.3	C9-C8-H8	107.3
C8-C9-C1	104.67(14)	C8-C9-H9A	110.8
C1-C9-H9A	110.8	C8-C9-H9B	110.8
C1-C9-H9B	110.8	H9A-C9-H9B	108.9

C11-C10-C6	104.98(14)	C11-C10-H10A	110.8
C6-C10-H10A	110.8	C11-C10-H10B	110.8
C6-C10-H10B	110.8	H10A-C10-H10B	108.8
O1-C11-C10	110.33(14)	O1-C11-C12	115.08(15)
C10-C11-C12	102.87(14)	O1-C11-H11	109.4
C10-C11-H11	109.4	C12-C11-H11	109.4
C11-C12-C1	105.17(14)	C11-C12-H12A	110.7
C1-C12-H12A	110.7	C11-C12-H12B	110.7
C1-C12-H12B	110.7	H12A-C12-H12B	108.8
O2-C13-N1	122.55(15)	O2-C13-N2	121.58(16)
N1-C13-N2	115.87(15)	C19-C14-C15	119.37(18)
C19-C14-N2	123.88(17)	C15-C14-N2	116.72(17)
O3-C15-C16	125.4(2)	O3-C15-C14	115.01(17)
C16-C15-C14	119.6(2)	C17-C16-C15	119.9(2)
C17-C16-H16	120.0	C15-C16-H16	120.0
C16-C17-C18	121.7(2)	C16-C17-H17	119.2
C18-C17-H17	119.2	C17-C18-C19	118.1(2)
C17-C18-C21	122.0(2)	C19-C18-C21	119.9(2)
C14-C19-C18	121.3(2)	C14-C19-H19	119.3
C18-C19-H19	119.3	O3-C20-H20A	109.5
O3-C20-H20B	109.5	H20A-C20-H20B	109.5
O3-C20-H20C	109.5	H20A-C20-H20C	109.5
H20B-C20-H20C	109.5	C18-C21-H21A	109.5
C18-C21-H21B	109.5	H21A-C21-H21B	109.5
C18-C21-H21C	109.5	H21A-C21-H21C	109.5
H21B-C21-H21C	109.5	N1-C22-C23	117.28(15)
N1-C22-H22A	108.0	C23-C22-H22A	108.0
N1-C22-H22B	108.0	C23-C22-H22B	108.0
H22A-C22-H22B	107.2	C28-C23-C24	118.67(19)
C28-C23-C22	123.86(17)	C24-C23-C22	117.28(18)
C25-C24-C23	120.6(2)	C25-C24-H24	119.7
C23-C24-H24	119.7	C26-C25-C24	120.5(3)

C26-C25-H25	119.7	C24-C25-H25	119.7
C25-C26-C27	119.9(2)	C25-C26-H26	120.0
C27-C26-H26	120.0	C26-C27-C28	119.7(2)
C26-C27-H27	120.2	C28-C27-H27	120.2
C23-C28-C27	120.6(2)	C23-C28-H28	119.7
C27-C28-H28	119.7		

Table S32. Torsion angles (°) for *anti,syn*-**22.**

C9-C1-C2-C3	164.99(18)	C12-C1-C2-C3	-68.6(2)
C6-C1-C2-C3	48.9(2)	C1-C2-C3-C4	-54.0(3)
C2-C3-C4-C5	4.9(3)	C3-C4-C5-C6	48.0(2)
C4-C5-C6-C10	66.4(2)	C4-C5-C6-C7	-167.34(17)
C4-C5-C6-C1	-51.7(2)	C2-C1-C6-C10	-119.60(16)
C9-C1-C6-C10	121.51(15)	C12-C1-C6-C10	2.43(17)
C2-C1-C6-C5	3.4(2)	C9-C1-C6-C5	-115.45(16)
C12-C1-C6-C5	125.46(15)	C2-C1-C6-C7	122.54(15)
C9-C1-C6-C7	3.65(17)	C12-C1-C6-C7	-115.43(15)
C10-C6-C7-C8	-141.88(15)	C5-C6-C7-C8	91.16(17)
C1-C6-C7-C8	-29.03(17)	C13-N1-C8-C7	125.64(17)
C22-N1-C8-C7	-67.2(2)	C13-N1-C8-C9	-114.20(18)
C22-N1-C8-C9	53.0(2)	C6-C7-C8-N1	171.40(13)
C6-C7-C8-C9	43.81(17)	N1-C8-C9-C1	-168.45(14)
C7-C8-C9-C1	-41.56(17)	C2-C1-C9-C8	-97.29(18)
C12-C1-C9-C8	136.52(15)	C6-C1-C9-C8	23.06(17)
C5-C6-C10-C11	-148.83(16)	C7-C6-C10-C11	85.86(17)
C1-C6-C10-C11	-26.71(18)	C6-C10-C11-O1	164.46(14)
C6-C10-C11-C12	41.21(18)	O1-C11-C12-C1	-159.54(15)
C10-C11-C12-C1	-39.52(18)	C2-C1-C12-C11	144.31(16)
C9-C1-C12-C11	-91.00(18)	C6-C1-C12-C11	22.66(18)
C22-N1-C13-O2	-166.10(17)	C8-N1-C13-O2	0.8(3)
C22-N1-C13-N2	13.5(2)	C8-N1-C13-N2	-179.62(15)
C14-N2-C13-O2	-3.1(3)	C14-N2-C13-N1	177.32(16)

C13-N2-C14-C19	33.4(3)	C13-N2-C14-C15	-148.50(18)
C20-O3-C15-C16	-5.3(3)	C20-O3-C15-C14	176.04(18)
C19-C14-C15-O3	177.82(16)	N2-C14-C15-O3	-0.4(2)
C19-C14-C15-C16	-1.0(3)	N2-C14-C15-C16	-179.13(17)
O3-C15-C16-C17	-179.2(2)	C14-C15-C16-C17	-0.6(3)
C15-C16-C17-C18	1.3(4)	C16-C17-C18-C19	-0.4(3)
C16-C17-C18-C21	-178.8(2)	C15-C14-C19-C18	1.9(3)
N2-C14-C19-C18	179.90(17)	C17-C18-C19-C14	-1.2(3)
C21-C18-C19-C14	177.3(2)	C13-N1-C22-C23	-80.2(2)
C8-N1-C22-C23	113.15(18)	N1-C22-C23-C28	-30.1(3)
N1-C22-C23-C24	155.15(19)	C28-C23-C24-C25	-1.2(4)
C22-C23-C24-C25	173.9(3)	C23-C24-C25-C26	-0.4(5)
C24-C25-C26-C27	1.6(5)	C25-C26-C27-C28	-1.2(4)
C24-C23-C28-C27	1.5(3)	C22-C23-C28-C27	-173.15(19)
C26-C27-C28-C23	-0.4(4)		

Table S33. Anisotropic atomic displacement parameters (Å^2) for *anti,syn*-**22**.
The anisotropic atomic displacement factor exponent takes the form: $-2\pi^2[h^2 a^{*2} U_{11} + ... + 2 h k a^* b^* U_{12}]$

	U_{11}	U_{22}	U_{33}	U_{23}	U_{13}	U_{12}
N1	0.0324(8)	0.0334(8)	0.0386(8)	-0.0075(7)	-0.0022(6)	0.0060(6)
N2	0.0376(8)	0.0355(9)	0.0433(9)	-0.0092(7)	-0.0007(7)	0.0077(7)
O1	0.0386(8)	0.0521(9)	0.0538(8)	-0.0221(7)	0.0026(6)	-0.0095(6)
O2	0.0350(7)	0.0416(7)	0.0456(7)	-0.0102(6)	-0.0035(6)	0.0054(6)
O3	0.0579(9)	0.0398(8)	0.0714(10)	0.0065(7)	0.0101(8)	0.0136(7)
C1	0.0364(9)	0.0315(9)	0.0338(9)	-0.0018(7)	0.0036(7)	0.0035(7)
C2	0.0561(12)	0.0455(11)	0.0406(11)	-0.0029(9)	-0.0042(9)	0.0149(9)
C3	0.0503(12)	0.0582(14)	0.0690(15)	-0.0120(12)	-0.0093(11)	0.0198(11)
C4	0.0332(11)	0.0703(15)	0.0718(15)	-0.0275(13)	-0.0023(10)	0.0082(10)
C5	0.0345(10)	0.0508(12)	0.0522(12)	-0.0188(10)	0.0065(8)	-0.0065(8)
C6	0.0308(9)	0.0337(9)	0.0344(9)	-0.0073(8)	0.0044(7)	-0.0027(7)
C7	0.0375(10)	0.0312(9)	0.0385(10)	-0.0012(8)	0.0063(7)	-0.0038(7)
C8	0.0324(9)	0.0326(9)	0.0366(9)	-0.0065(8)	0.0019(7)	0.0045(7)

C9	0.0433(10)	0.0363(10)	0.0367(9)	0.0030(8)	0.0102(8)	0.0062(8)
C10	0.0348(9)	0.0403(10)	0.0347(10)	-0.0073(8)	0.0052(7)	-0.0043(8)
C11	0.0324(9)	0.0354(10)	0.0394(10)	-0.0098(8)	0.0027(7)	-0.0029(7)
C12	0.0433(10)	0.0326(10)	0.0468(11)	-0.0024(8)	0.0066(8)	-0.0019(8)
C13	0.0308(9)	0.0310(9)	0.0326(9)	-0.0018(7)	0.0054(7)	0.0005(7)
C14	0.0348(9)	0.0323(9)	0.0451(10)	-0.0075(8)	0.0142(8)	-0.0035(7)
C15	0.0441(11)	0.0349(11)	0.0617(13)	-0.0035(10)	0.0204(9)	-0.0008(8)
C16	0.0680(15)	0.0328(11)	0.0920(19)	-0.0138(12)	0.0350(14)	-0.0057(10)
C17	0.0702(16)	0.0558(15)	0.0798(18)	-0.0347(14)	0.0333(14)	-0.0200(12)
C18	0.0523(12)	0.0660(15)	0.0541(13)	-0.0251(12)	0.0219(10)	-0.0180(11)
C19	0.0423(10)	0.0471(11)	0.0437(11)	-0.0088(9)	0.0131(8)	-0.0055(9)
C20	0.0666(15)	0.0510(14)	0.102(2)	0.0288(14)	0.0315(14)	0.0230(12)
C21	0.0748(17)	0.122(3)	0.0538(14)	-0.0379(16)	0.0118(12)	-0.0230(17)
C22	0.0326(9)	0.0336(9)	0.0430(10)	-0.0019(8)	-0.0031(8)	0.0012(7)
C23	0.0427(10)	0.0331(10)	0.0383(10)	-0.0035(8)	-0.0013(8)	0.0020(8)
C24	0.0497(13)	0.0832(18)	0.0697(16)	0.0247(14)	0.0049(11)	0.0199(12)
C25	0.0675(18)	0.123(3)	0.093(2)	0.045(2)	-0.0013(16)	0.0352(18)
C26	0.088(2)	0.0741(18)	0.0651(16)	0.0210(14)	-0.0098(14)	0.0174(15)
C27	0.0902(19)	0.0458(13)	0.0574(14)	0.0091(11)	0.0082(12)	-0.0084(12)
C28	0.0521(12)	0.0397(11)	0.0590(13)	0.0040(10)	0.0026(10)	-0.0032(9)

Table S34. Hydrogen atomic coordinates and isotropic atomic displacement parameters (Å^2) for *anti,syn*-**22**.

	x/a	y/b	z/c	U(eq)
H02	0.774(3)	0.5860(9)	0.610(2)	0.070(8)
H01	0.727(4)	0.8536(9)	1.007(2)	0.074(8)
H2A	0.3804	0.8263	0.5701	0.058
H2B	0.2929	0.7751	0.5706	0.058
H3A	0.0938	0.8326	0.6076	0.072
H3B	0.2140	0.8551	0.7146	0.072
H4A	0.0944	0.8094	0.8399	0.071
H4B	-0.0135	0.7836	0.7317	0.071

H5A	0.1828	0.7239	0.7202	0.055
H5B	0.1567	0.7290	0.8592	0.055
H7A	0.5877	0.7053	0.8728	0.043
H7B	0.4138	0.6781	0.8371	0.043
H8	0.4228	0.6958	0.6347	0.041
H9A	0.5878	0.7574	0.5734	0.046
H9B	0.7062	0.7565	0.6972	0.046
H10A	0.3429	0.8024	0.9547	0.044
H10B	0.4558	0.7582	1.0055	0.044
H11	0.6905	0.7868	0.9221	0.043
H12A	0.6616	0.8358	0.7547	0.049
H12B	0.4837	0.8557	0.7869	0.049
H16	0.7638	0.4429	0.4329	0.075
H17	0.5988	0.4537	0.2530	0.08
H19	0.5277	0.5923	0.3357	0.053
H20A	0.8127	0.4410	0.6546	0.108
H20B	0.9749	0.4670	0.7171	0.108
H20C	0.9702	0.4503	0.5804	0.108
H21A	0.3254	0.5177	0.1659	0.125
H21B	0.4869	0.5153	0.0930	0.125
H21C	0.4258	0.5654	0.1413	0.125
H22A	0.8392	0.6937	0.7524	0.044
H22B	0.8915	0.6590	0.6498	0.044
H24	1.1139	0.6251	0.7735	0.081
H25	1.2133	0.5734	0.9269	0.114
H26	1.0336	0.5423	1.0574	0.092
H27	0.7482	0.5600	1.0301	0.077
H28	0.6464	0.6114	0.8742	0.061

Table S35. Hydrogen bond distances (Å) and angles (°) for *anti,syn*-**22**.

	Donor-H	Acceptor-H	Donor-Acceptor	Angle
O1-H01···O2	0.87(3)	1.85(3)	2.7243(19)	175.(2)

3.6 X-ray crystal structure of *syn*-25

Compound *syn*-**25** was crystallized from EtOAc. Colorless crystals. Code DAN7353

*X-ray crystal structure analysis of syn-***25** *(dan7353):* A colorless prism-like specimen of $C_{19}H_{22}F_2N_2O_2$, approximate dimensions 0.100 mm x 0.140 mm x 0.180 mm, was used for the X-ray crystallographic analysis. The X-ray intensity data were measured.

The integration of the data using a triclinic unit cell yielded a total of 9040 reflections to a maximum θ angle of 67.31° (0.84 Å resolution), of which 9040 were independent (average redundancy 1.000, completeness = 96.6%, R_{sig} = 2.87%) and 7415 (82.02%) were greater than 2σ(F^2).

The final cell constants of a = 12.9305(5) Å, b = 13.0504(5) Å, c = 17.0932(4) Å, α = 112.037(2)°, β = 97.638(2)°, γ = 97.0010(10)°, volume = 2603.27(16) $Å^3$, are based upon the refinement of the XYZ-centroids of reflections above 20 σ(I). Data were corrected for absorption effects using the multi-scan method (SADABS). The calculated minimum and maximum transmission coefficients (based on crystal size) are 0.8630 and 0.9200.

The structure was solved and refined using the Bruker SHELXTL Software Package, using the space group P -1, with Z = 6 for the formula unit, $C_{19}H_{22}F_2N_2O_2$. The final anisotropic full-matrix least-squares refinement on F^2 with 928 variables converged at R1 = 6.16%, for the observed data and wR2 = 18.77% for all data. The goodness-of-fit was 1.038. The largest peak in the final difference electron density synthesis was 1.261 $e^-/Å^3$ and the largest hole was -0.361 $e^-/Å^3$ with an RMS deviation of 0.044 $e^-/Å^3$. On the basis of the final model, the calculated density was 1.333 g/cm^3 and F(000), 1104 e^-. CCDC number: 2073470.

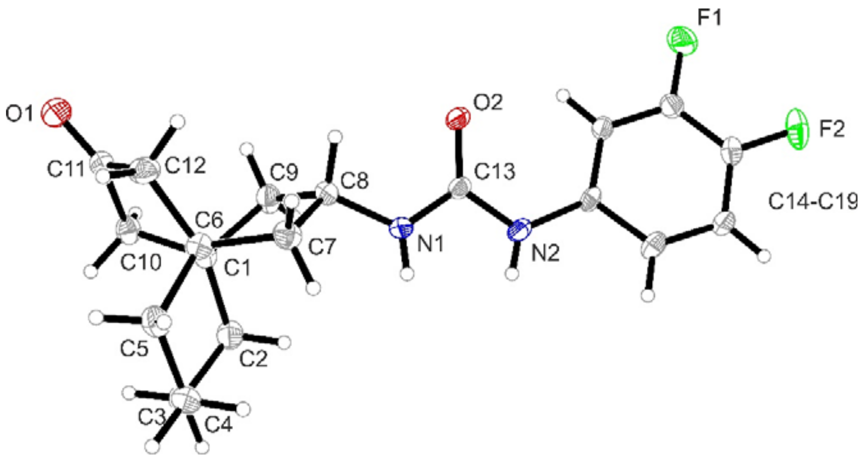

Table S36. Sample and crystal data for *syn*-**25**.

Identification code	dan7353	
Chemical formula	$C_{19}H_{22}F_2N_2O_2$	
Formula weight	348.38 g/mol	
Temperature	223(2) K	
Wavelength	1.54178 Å	
Crystal size	0.100 x 0.140 x 0.180 mm	
Crystal habit	colorless prism	
Crystal system	Triclinic	
Space group	P -1	
Unit cell dimensions	a = 12.9305(5) Å	α = 112.037(2)°
	b = 13.0504(5) Å	β = 97.638(2)°
	c = 17.0932(4) Å	γ = 97.0010(10)°
Volume	2603.27(16) $Å^3$	
Z	6	
Density (calculated)	1.333 g/cm^3	
Absorption coefficient	0.846 mm^{-1}	
F(000)	1104	

Table S37. Data collection and structure refinement for *syn*-**25**.

Theta range for data collection	3.51 to 67.31°	
Reflections collected	9040	
Absorption correction	multi-scan	
Max. and min. transmission	0.9200 and 0.8630	
Structure solution technique	direct methods	
Structure solution program	SHELXL-2014/7 (Sheldrick, 2014)	
Refinement method	Full-matrix least-squares on F^2	
Refinement program	SHELXL-2014/7 (Sheldrick, 2014)	
Function minimized	Σ w(F_o^2 - F_c^2)^2	
Data / restraints / parameters	9040 / 622 / 928	
Goodness-of-fit on F^2	1.038	
Final R indices	7415 data; I>2σ(I)	R1 = 0.0616, wR2 = 0.1764
	all data	R1 = 0.0716, wR2 = 0.1877
Weighting scheme	$w=1/[\sigma^2(F_o^2)+(0.1064P)^2+1.0647P]$ where $P=(F_o^2+2F_c^2)/3$	
Largest diff. peak and hole	1.261 and -0.361 eÅ^{-3}	
R.M.S. deviation from mean	0.044 eÅ^{-3}	

Table S38. Atomic coordinates and equivalent isotropic atomic displacement parameters (Å^2) for *syn*-**25**.

U(eq) is defined as one third of the trace of the orthogonalized U_{ij} tensor.

	x/a	y/b	z/c	U(eq)
N1A	0.24735(17)	0.81739(19)	0.70007(17)	0.0655(6)
O1A	0.50201(17)	0.4253(2)	0.58788(19)	0.0997(8)
O2A	0.37988(12)	0.96407(14)	0.72681(11)	0.0594(4)
C1A	0.29377(19)	0.5524(2)	0.67530(15)	0.0535(5)
C2A	0.2051(3)	0.5529(3)	0.72632(19)	0.0737(8)
C3A	0.1231(3)	0.4481(3)	0.6858(2)	0.0872(9)
C4A	0.0757(2)	0.4317(3)	0.5982(2)	0.0833(9)
C5A	0.1561(2)	0.4240(2)	0.54147(18)	0.0727(7)
C6A	0.25134(19)	0.5241(2)	0.57924(15)	0.0553(6)
C7A	0.2225(2)	0.6326(2)	0.57731(18)	0.0688(7)

C8A	0.3013(2)	0.7290(2)	0.6518(2)	0.0656(7)
C9A	0.3564(2)	0.6739(2)	0.7061(2)	0.0715(7)
C10A	0.3677(2)	0.4710(2)	0.67659(18)	0.0681(7)
C11A	0.4194(2)	0.4562(2)	0.5996(2)	0.0714(8)
C12A	0.3469(2)	0.4879(3)	0.5367(2)	0.0775(8)
C13A	0.29118(17)	0.9277(2)	0.73501(15)	0.0510(5)
N2A	0.22957(17)	0.9963(2)	0.78036(13)	0.0574(5)
C14A	0.2516(9)	0.1181(7)	0.8099(12)	0.050(2)
C15A	0.3486(8)	0.1846(6)	0.8209(8)	0.054(2)
C16A	0.3573(9)	0.2991(6)	0.8453(7)	0.067(2)
F1A	0.4513(8)	0.3646(7)	0.8587(8)	0.112(3)
C17A	0.2712(10)	0.3512(7)	0.8589(5)	0.064(2)
F2A	0.2816(10)	0.4633(6)	0.8848(6)	0.110(3)
C18A	0.1737(9)	0.2889(8)	0.8514(5)	0.060(2)
C19A	0.1650(8)	0.1738(8)	0.8251(7)	0.065(2)
C14D	0.2274(11)	0.1062(9)	0.8042(15)	0.057(3)
C15D	0.3166(11)	0.1775(9)	0.8081(10)	0.065(3)
C16D	0.3129(10)	0.2898(8)	0.8351(8)	0.073(3)
F1D	0.3998(12)	0.3617(9)	0.8375(8)	0.114(3)
C17D	0.2218(11)	0.3272(8)	0.8557(6)	0.057(2)
F2D	0.2215(12)	0.4399(8)	0.8809(7)	0.113(3)
C18D	0.1338(10)	0.2558(9)	0.8539(6)	0.066(2)
C19D	0.1373(9)	0.1439(9)	0.8288(8)	0.062(2)
O2B	0.97814(12)	0.23661(15)	0.31562(12)	0.0606(4)
N1B	0.15035(15)	0.27479(17)	0.30640(13)	0.0524(5)
N2B	0.09597(15)	0.13411(19)	0.34829(14)	0.0564(5)
F1B	0.79182(15)	0.0270(2)	0.45118(16)	0.1036(7)
F2B	0.84065(13)	0.82451(15)	0.42165(12)	0.0830(5)
C2B	0.2020(2)	0.2915(3)	0.09317(18)	0.0722(7)
C3B	0.3022(3)	0.3291(3)	0.0663(2)	0.0875(9)
C4B	0.3837(2)	0.4081(3)	0.1438(2)	0.0753(8)
C5B	0.3413(2)	0.5099(3)	0.1946(2)	0.0699(7)

C7B	0.25039(19)	0.4368(2)	0.29278(16)	0.0597(6)
C8B	0.13954(18)	0.3728(2)	0.28727(16)	0.0548(6)
C9B	0.07662(19)	0.3462(2)	0.19647(18)	0.0620(6)
C1B	0.15272(19)	0.3883(2)	0.14805(17)	0.0614(6)
C6B	0.23660(19)	0.4820(2)	0.22235(18)	0.0600(6)
C10B	0.0969(7)	0.4675(9)	0.1122(7)	0.064(2)
C11B	0.1139(7)	0.5835(8)	0.1822(8)	0.064(2)
O1B	0.0718(7)	0.6591(9)	0.1758(8)	0.104(3)
C12B	0.1851(8)	0.5868(7)	0.2604(7)	0.066(2)
C10E	0.1047(7)	0.4318(10)	0.0825(7)	0.059(2)
C11E	0.1037(7)	0.5495(10)	0.1435(9)	0.067(2)
O1E	0.0565(8)	0.6174(11)	0.1289(11)	0.119(3)
C12E	0.1779(9)	0.5804(8)	0.2280(8)	0.068(3)
C13B	0.06876(17)	0.2160(2)	0.32318(14)	0.0493(5)
C14B	0.02964(17)	0.0579(2)	0.36766(14)	0.0501(5)
C15B	0.93957(18)	0.0839(2)	0.40226(15)	0.0545(5)
C16B	0.87834(18)	0.0038(2)	0.41884(16)	0.0581(6)
C17B	0.9041(2)	0.9004(2)	0.40418(17)	0.0602(6)
C18B	0.9941(2)	0.8756(2)	0.37313(18)	0.0651(6)
C19B	0.0562(2)	0.9533(2)	0.35433(17)	0.0596(6)
O2C	0.31640(12)	0.15762(15)	0.36296(10)	0.0586(4)
F1C	0.70440(13)	0.13752(17)	0.64149(11)	0.0881(5)
F2C	0.55312(14)	0.18295(15)	0.73486(9)	0.0782(5)
N2C	0.47843(14)	0.11335(16)	0.39154(12)	0.0487(4)
N1C	0.40558(15)	0.08570(19)	0.25534(13)	0.0562(5)
C7C	0.2428(3)	0.9454(4)	0.1663(2)	0.0652(10)
C8C	0.3145(4)	0.0584(5)	0.1865(3)	0.0536(12)
C9C	0.3490(4)	0.0484(4)	0.1014(3)	0.0562(11)
C1C	0.3126(3)	0.9260(3)	0.0375(2)	0.0524(9)
C2C	0.4021(3)	0.8603(3)	0.0419(2)	0.0766(10)
C3C	0.3655(6)	0.7342(5)	0.0032(4)	0.0991(17)
C4C	0.2743(5)	0.7025(4)	0.0457(3)	0.1080(16)

C5C	0.1826(4)	0.7591(4)	0.0353(3)	0.0979(15)
C6C	0.2137(4)	0.8869(4)	0.0686(3)	0.0623(15)
C10C	0.2691(3)	0.9166(3)	0.9468(2)	0.0637(9)
C11C	0.1557(3)	0.9331(4)	0.9474(2)	0.0611(10)
O1C	0.0963(2)	0.9437(3)	0.89132(17)	0.0789(8)
C12C	0.1257(3)	0.9320(5)	0.0298(2)	0.0764(11)
C7F	0.2269(11)	0.9990(13)	0.1582(8)	0.063(4)
C8F	0.3314(13)	0.0822(18)	0.1826(10)	0.053(5)
C9F	0.3823(13)	0.0504(19)	0.1028(10)	0.055(5)
C1F	0.3002(10)	0.9629(11)	0.0289(8)	0.051(4)
C2F	0.3477(13)	0.8829(13)	0.9599(9)	0.085(5)
C3F	0.4032(16)	0.8006(19)	0.9868(15)	0.112(7)
C4F	0.3259(18)	0.7310(16)	0.0171(15)	0.078(6)
C5F	0.2824(13)	0.8117(12)	0.0915(10)	0.087(5)
C6F	0.2328(12)	0.9029(13)	0.0739(10)	0.063(7)
C10F	0.2164(9)	0.0130(11)	0.9897(9)	0.068(4)
C11F	0.1153(11)	0.9307(13)	0.9645(10)	0.062(4)
O1F	0.0362(11)	0.9225(11)	0.9112(9)	0.095(4)
C12F	0.1255(9)	0.8553(12)	0.0118(8)	0.057(3)
C13C	0.39475(16)	0.12025(18)	0.33830(14)	0.0455(5)
C14C	0.49182(16)	0.13647(17)	0.47987(14)	0.0442(5)
C15C	0.59182(18)	0.13142(19)	0.51874(15)	0.0532(5)
C16C	0.60924(19)	0.1465(2)	0.60386(16)	0.0576(6)
C17C	0.5315(2)	0.1686(2)	0.65107(15)	0.0572(6)
C18C	0.4330(2)	0.1737(2)	0.61363(16)	0.0581(6)
C19C	0.41263(18)	0.15746(19)	0.52769(15)	0.0516(5)

Table S39. Bond lengths (Å) for *syn*-**25**.

N1A-C13A	1.348(3)	N1A-C8A	1.460(3)
N1A-H01	0.803(14)	O1A-C11A	1.202(3)
O2A-C13A	1.233(3)	C1A-C10A	1.517(3)
C1A-C2A	1.529(4)	C1A-C6A	1.546(3)

C1A-C9A	1.548(4)	C2A-C3A	1.488(5)
C2A-H201	0.98	C2A-H202	0.98
C3A-C4A	1.466(5)	C3A-H301	0.98
C3A-H302	0.98	C4A-C5A	1.501(4)
C4A-H401	0.98	C4A-H402	0.98
C5A-C6A	1.550(4)	C5A-H501	0.98
C5A-H502	0.98	C6A-C7A	1.518(4)
C6A-C12A	1.551(4)	C7A-C8A	1.544(4)
C7A-H701	0.98	C7A-H702	0.98
C8A-C9A	1.529(4)	C8A-H8A	0.99
C9A-H901	0.98	C9A-H902	0.98
C10A-C11A	1.513(4)	C10A-H10A	0.98
C10A-H10B	0.98	C11A-C12A	1.540(4)
C12A-H12A	0.98	C12A-H12B	0.98
C13A-N2A	1.362(3)	N2A-C14D	1.342(10)
N2A-C14A	1.454(8)	N2A-H02	0.800(14)
C14A-C15A	1.383(10)	C14A-C19A	1.408(9)
C15A-C16A	1.378(9)	C15A-H15A	0.94
C16A-F1A	1.337(10)	C16A-C17A	1.375(9)
C17A-F2A	1.343(8)	C17A-C18A	1.377(8)
C18A-C19A	1.383(9)	C18A-H18A	0.94
C19A-H19A	0.94	C14D-C15D	1.368(11)
C14D-C19D	1.375(11)	C15D-C16D	1.371(10)
C15D-H15D	0.94	C16D-F1D	1.359(11)
C16D-C17D	1.367(10)	C17D-C18D	1.369(9)
C17D-F2D	1.370(9)	C18D-C19D	1.368(10)
C18D-H18D	0.94	C19D-H19D	0.94
O2B-C13B	1.235(3)	N1B-C13B	1.354(3)
N1B-C8B	1.451(3)	N1B-H03	0.806(14)
N2B-C13B	1.358(3)	N2B-C14B	1.396(3)
N2B-H04	0.803(14)	F1B-C16B	1.331(3)
F2B-C17B	1.354(3)	C2B-C3B	1.517(4)

C2B-C1B	1.532(4)	C2B-H211	0.98
C2B-H212	0.98	C3B-C4B	1.505(5)
C3B-H311	0.98	C3B-H312	0.98
C4B-C5B	1.502(5)	C4B-H411	0.98
C4B-H412	0.98	C5B-C6B	1.540(4)
C5B-H511	0.98	C5B-H512	0.98
C7B-C6B	1.526(4)	C7B-C8B	1.541(3)
C7B-H711	0.98	C7B-H712	0.98
C8B-C9B	1.544(4)	C8B-H8B	0.99
C9B-C1B	1.543(3)	C9B-H911	0.98
C9B-H912	0.98	C1B-C10E	1.531(8)
C1B-C6B	1.559(4)	C1B-C10B	1.582(8)
C6B-C12E	1.549(9)	C6B-C12B	1.552(8)
C10B-C11B	1.503(9)	C10B-H10C	0.98
C10B-H10D	0.98	C11B-O1B	1.216(8)
C11B-C12B	1.502(9)	C12B-H12D	0.98
C12B-H12C	0.98	C10E-C11E	1.501(9)
C10E-H10E	0.98	C10E-H10F	0.98
C11E-O1E	1.215(9)	C11E-C12E	1.507(10)
C12E-H12E	0.98	C12E-H12F	0.98
C14B-C19B	1.391(3)	C14B-C15B	1.397(3)
C15B-C16B	1.372(3)	C15B-H15B	0.94
C16B-C17B	1.367(4)	C17B-C18B	1.367(4)
C18B-C19B	1.375(4)	C18B-H18B	0.94
C19B-H19B	0.94	O2C-C13C	1.234(3)
F1C-C16C	1.350(3)	F2C-C17C	1.358(3)
N2C-C13C	1.353(3)	N2C-C14C	1.406(3)
N2C-H06	0.800(14)	N1C-C13C	1.350(3)
N1C-C8F	1.448(15)	N1C-C8C	1.453(4)
N1C-H05	0.802(13)	C7C-C6C	1.522(5)
C7C-C8C	1.535(6)	C7C-H7C1	0.98
C7C-H7C2	0.98	C8C-C9C	1.542(6)

C8C-H8C	0.99	C9C-C1C	1.529(6)
C9C-H9C1	0.98	C9C-H9C2	0.98
C1C-C10C	1.531(5)	C1C-C2C	1.532(5)
C1C-C6C	1.550(6)	C2C-C3C	1.510(6)
C2C-H2C1	0.98	C2C-H2C2	0.98
C3C-C4C	1.557(7)	C3C-H3C1	0.98
C3C-H3C2	0.98	C4C-C5C	1.495(7)
C4C-H4C1	0.98	C4C-H4C2	0.98
C5C-C6C	1.528(6)	C5C-H5C1	0.98
C5C-H5C2	0.98	C6C-C12C	1.528(6)
C10C-C11C	1.508(5)	C10C-H10G	0.98
C10C-H10H	0.98	C11C-O1C	1.207(4)
C11C-C12C	1.515(5)	C12C-H12G	0.98
C12C-H12H	0.98	C7F-C8F	1.526(14)
C7F-C6F	1.534(15)	C7F-H7F1	0.98
C7F-H7F2	0.98	C8F-C9F	1.532(14)
C8F-H8F	0.99	C9F-C1F	1.514(14)
C9F-H9F1	0.98	C9F-H9F2	0.98
C1F-C2F	1.512(12)	C1F-C10F	1.536(14)
C1F-C6F	1.557(15)	C2F-C3F	1.532(14)
C2F-H2F1	0.98	C2F-H2F2	0.98
C3F-C4F	1.537(14)	C3F-H3F1	0.98
C3F-H3F2	0.98	C4F-C5F	1.540(14)
C4F-H4F1	0.98	C4F-H4F2	0.98
C5F-C6F	1.524(13)	C5F-H5F1	0.98
C5F-H5F2	0.98	C6F-C12F	1.530(15)
C10F-C11F	1.487(13)	C10F-H10I	0.98
C10F-H10J	0.98	C11F-O1F	1.240(13)
C11F-C12F	1.497(13)	C12F-H12I	0.98
C12F-H12J	0.98	C14C-C19C	1.384(3)
C14C-C15C	1.394(3)	C15C-C16C	1.376(3)
C15C-H15C	0.94	C16C-C17C	1.363(4)

C17C-C18C	1.368(4)	C18C-C19C	1.386(3)
C18C-H18C	0.94	C19C-H19C	0.94

Table S40. Bond angles (°) for *syn*-**25**.

C13A-N1A-C8A	123.6(2)	C13A-N1A-H01	117.(2)
C8A-N1A-H01	120.(2)	C10A-C1A-C2A	115.4(2)
C10A-C1A-C6A	105.8(2)	C2A-C1A-C6A	112.9(2)
C10A-C1A-C9A	111.2(2)	C2A-C1A-C9A	109.0(2)
C6A-C1A-C9A	101.7(2)	C3A-C2A-C1A	112.7(2)
C3A-C2A-H201	109.0	C1A-C2A-H201	109.0
C3A-C2A-H202	109.0	C1A-C2A-H202	109.0
H201-C2A-H202	107.8	C4A-C3A-C2A	110.4(3)
C4A-C3A-H301	109.6	C2A-C3A-H301	109.6
C4A-C3A-H302	109.6	C2A-C3A-H302	109.6
H301-C3A-H302	108.1	C3A-C4A-C5A	112.7(3)
C3A-C4A-H401	109.1	C5A-C4A-H401	109.1
C3A-C4A-H402	109.1	C5A-C4A-H402	109.1
H401-C4A-H402	107.8	C4A-C5A-C6A	113.9(2)
C4A-C5A-H501	108.8	C6A-C5A-H501	108.8
C4A-C5A-H502	108.8	C6A-C5A-H502	108.8
H501-C5A-H502	107.7	C7A-C6A-C1A	104.8(2)
C7A-C6A-C5A	113.2(2)	C1A-C6A-C5A	111.1(2)
C7A-C6A-C12A	114.9(2)	C1A-C6A-C12A	103.0(2)
C5A-C6A-C12A	109.3(2)	C6A-C7A-C8A	106.0(2)
C6A-C7A-H701	110.5	C8A-C7A-H701	110.5
C6A-C7A-H702	110.5	C8A-C7A-H702	110.5
H701-C7A-H702	108.7	N1A-C8A-C9A	113.6(3)
N1A-C8A-C7A	111.3(2)	C9A-C8A-C7A	105.8(2)
N1A-C8A-H8A	108.6	C9A-C8A-H8A	108.6
C7A-C8A-H8A	108.6	C8A-C9A-C1A	107.2(2)
C8A-C9A-H901	110.3	C1A-C9A-H901	110.3
C8A-C9A-H902	110.3	C1A-C9A-H902	110.3

H901-C9A-H902	108.5	C11A-C10A-C1A	104.4(2)
C11A-C10A-H10A	110.9	C1A-C10A-H10A	110.9
C11A-C10A-H10B	110.9	C1A-C10A-H10B	110.9
H10A-C10A-H10B	108.9	O1A-C11A-C10A	127.5(3)
O1A-C11A-C12A	124.8(3)	C10A-C11A-C12A	107.8(2)
C11A-C12A-C6A	106.5(2)	C11A-C12A-H12A	110.4
C6A-C12A-H12A	110.4	C11A-C12A-H12B	110.4
C6A-C12A-H12B	110.4	H12A-C12A-H12B	108.6
O2A-C13A-N1A	123.0(2)	O2A-C13A-N2A	122.4(2)
N1A-C13A-N2A	114.6(2)	C14D-N2A-C13A	133.4(7)
C13A-N2A-C14A	123.9(5)	C14D-N2A-H02	114.(2)
C13A-N2A-H02	110.4(19)	C14A-N2A-H02	124.9(19)
C15A-C14A-C19A	116.5(7)	C15A-C14A-N2A	126.3(7)
C19A-C14A-N2A	117.1(7)	C16A-C15A-C14A	120.4(8)
C16A-C15A-H15A	119.8	C14A-C15A-H15A	119.8
F1A-C16A-C17A	117.2(7)	F1A-C16A-C15A	120.8(7)
C17A-C16A-C15A	121.9(7)	F2A-C17A-C16A	121.2(7)
F2A-C17A-C18A	119.0(7)	C16A-C17A-C18A	119.7(7)
C17A-C18A-C19A	117.9(7)	C17A-C18A-H18A	121.0
C19A-C18A-H18A	121.0	C18A-C19A-C14A	123.4(7)
C18A-C19A-H19A	118.3	C14A-C19A-H19A	118.3
N2A-C14D-C15D	119.0(9)	N2A-C14D-C19D	118.4(10)
C15D-C14D-C19D	122.5(9)	C14D-C15D-C16D	117.6(10)
C14D-C15D-H15D	121.2	C16D-C15D-H15D	121.2
F1D-C16D-C17D	121.2(9)	F1D-C16D-C15D	118.5(10)
C17D-C16D-C15D	120.3(9)	C16D-C17D-C18D	121.8(8)
C16D-C17D-F2D	118.0(9)	C18D-C17D-F2D	120.2(8)
C19D-C18D-C17D	118.5(8)	C19D-C18D-H18D	120.7
C17D-C18D-H18D	120.7	C18D-C19D-C14D	119.2(9)
C18D-C19D-H19D	120.4	C14D-C19D-H19D	120.4
C13B-N1B-C8B	122.27(19)	C13B-N1B-H03	116.4(18)
C8B-N1B-H03	119.9(18)	C13B-N2B-C14B	127.74(19)

C13B-N2B-H04	119.(2)	C14B-N2B-H04	113.(2)
C3B-C2B-C1B	114.2(3)	C3B-C2B-H211	108.7
C1B-C2B-H211	108.7	C3B-C2B-H212	108.7
C1B-C2B-H212	108.7	H211-C2B-H212	107.6
C4B-C3B-C2B	110.7(2)	C4B-C3B-H311	109.5
C2B-C3B-H311	109.5	C4B-C3B-H312	109.5
C2B-C3B-H312	109.5	H311-C3B-H312	108.1
C5B-C4B-C3B	111.2(3)	C5B-C4B-H411	109.4
C3B-C4B-H411	109.4	C5B-C4B-H412	109.4
C3B-C4B-H412	109.4	H411-C4B-H412	108.0
C4B-C5B-C6B	113.9(2)	C4B-C5B-H511	108.8
C6B-C5B-H511	108.8	C4B-C5B-H512	108.8
C6B-C5B-H512	108.8	H511-C5B-H512	107.7
C6B-C7B-C8B	105.47(19)	C6B-C7B-H711	110.6
C8B-C7B-H711	110.6	C6B-C7B-H712	110.6
C8B-C7B-H712	110.6	H711-C7B-H712	108.8
N1B-C8B-C7B	109.6(2)	N1B-C8B-C9B	114.7(2)
C7B-C8B-C9B	105.93(19)	N1B-C8B-H8B	108.8
C7B-C8B-H8B	108.8	C9B-C8B-H8B	108.8
C1B-C9B-C8B	107.49(18)	C1B-C9B-H911	110.2
C8B-C9B-H911	110.2	C1B-C9B-H912	110.2
C8B-C9B-H912	110.2	H911-C9B-H912	108.5
C10E-C1B-C2B	102.7(5)	C10E-C1B-C9B	117.4(4)
C2B-C1B-C9B	110.1(2)	C10E-C1B-C6B	111.5(5)
C2B-C1B-C6B	112.5(2)	C9B-C1B-C6B	103.0(2)
C2B-C1B-C10B	123.7(5)	C9B-C1B-C10B	107.6(4)
C6B-C1B-C10B	97.6(4)	C7B-C6B-C5B	113.3(2)
C7B-C6B-C12E	123.3(5)	C5B-C6B-C12E	105.4(5)
C7B-C6B-C12B	104.8(5)	C5B-C6B-C12B	112.4(4)
C7B-C6B-C1B	104.07(19)	C5B-C6B-C1B	113.2(2)
C12E-C6B-C1B	96.4(5)	C12B-C6B-C1B	108.4(4)
C11B-C10B-C1B	109.6(5)	C11B-C10B-H10C	109.8

C1B-C10B-H10C	109.8	C11B-C10B-H10D	109.8
C1B-C10B-H10D	109.8	H10C-C10B-H10D	108.2
O1B-C11B-C12B	126.8(8)	O1B-C11B-C10B	124.2(8)
C12B-C11B-C10B	108.9(6)	C11B-C12B-C6B	102.2(6)
C11B-C12B-H12D	111.3	C6B-C12B-H12D	111.3
C11B-C12B-H12C	111.3	C6B-C12B-H12C	111.3
H12D-C12B-H12C	109.2	C11E-C10E-C1B	98.4(6)
C11E-C10E-H10E	112.1	C1B-C10E-H10E	112.1
C11E-C10E-H10F	112.1	C1B-C10E-H10F	112.1
H10E-C10E-H10F	109.7	O1E-C11E-C10E	127.3(9)
O1E-C11E-C12E	121.4(9)	C10E-C11E-C12E	111.1(7)
C11E-C12E-C6B	109.0(6)	C11E-C12E-H12E	109.9
C6B-C12E-H12E	109.9	C11E-C12E-H12F	109.9
C6B-C12E-H12F	109.9	H12E-C12E-H12F	108.3
O2B-C13B-N1B	121.6(2)	O2B-C13B-N2B	124.2(2)
N1B-C13B-N2B	114.18(19)	C19B-C14B-N2B	118.6(2)
C19B-C14B-C15B	118.8(2)	N2B-C14B-C15B	122.5(2)
C16B-C15B-C14B	118.7(2)	C16B-C15B-H15B	120.6
C14B-C15B-H15B	120.6	F1B-C16B-C17B	118.6(2)
F1B-C16B-C15B	119.4(2)	C17B-C16B-C15B	122.0(2)
F2B-C17B-C18B	120.9(2)	F2B-C17B-C16B	119.5(2)
C18B-C17B-C16B	119.7(2)	C17B-C18B-C19B	119.8(2)
C17B-C18B-H18B	120.1	C19B-C18B-H18B	120.1
C18B-C19B-C14B	120.9(2)	C18B-C19B-H19B	119.5
C14B-C19B-H19B	119.5	C13C-N2C-C14C	129.36(19)
C13C-N2C-H06	114.8(19)	C14C-N2C-H06	115.8(19)
C13C-N1C-C8F	128.0(8)	C13C-N1C-C8C	121.2(3)
C13C-N1C-H05	119.7(18)	C8F-N1C-H05	112.(2)
C8C-N1C-H05	116.1(18)	C6C-C7C-C8C	106.2(3)
C6C-C7C-H7C1	110.5	C8C-C7C-H7C1	110.5
C6C-C7C-H7C2	110.5	C8C-C7C-H7C2	110.5
H7C1-C7C-H7C2	108.7	N1C-C8C-C7C	113.1(4)

N1C-C8C-C9C	111.6(4)	C7C-C8C-C9C	105.5(3)
N1C-C8C-H8C	108.8	C7C-C8C-H8C	108.8
C9C-C8C-H8C	108.8	C1C-C9C-C8C	107.6(4)
C1C-C9C-H9C1	110.2	C8C-C9C-H9C1	110.2
C1C-C9C-H9C2	110.2	C8C-C9C-H9C2	110.2
H9C1-C9C-H9C2	108.5	C9C-C1C-C10C	112.0(4)
C9C-C1C-C2C	109.6(3)	C10C-C1C-C2C	114.1(3)
C9C-C1C-C6C	102.6(3)	C10C-C1C-C6C	104.4(3)
C2C-C1C-C6C	113.5(4)	C3C-C2C-C1C	113.9(4)
C3C-C2C-H2C1	108.8	C1C-C2C-H2C1	108.8
C3C-C2C-H2C2	108.8	C1C-C2C-H2C2	108.8
H2C1-C2C-H2C2	107.7	C2C-C3C-C4C	109.5(4)
C2C-C3C-H3C1	109.8	C4C-C3C-H3C1	109.8
C2C-C3C-H3C2	109.8	C4C-C3C-H3C2	109.8
H3C1-C3C-H3C2	108.2	C5C-C4C-C3C	111.9(4)
C5C-C4C-H4C1	109.2	C3C-C4C-H4C1	109.2
C5C-C4C-H4C2	109.2	C3C-C4C-H4C2	109.2
H4C1-C4C-H4C2	107.9	C4C-C5C-C6C	113.5(4)
C4C-C5C-H5C1	108.9	C6C-C5C-H5C1	108.9
C4C-C5C-H5C2	108.9	C6C-C5C-H5C2	108.9
H5C1-C5C-H5C2	107.7	C7C-C6C-C5C	113.7(4)
C7C-C6C-C12C	112.8(4)	C5C-C6C-C12C	109.0(4)
C7C-C6C-C1C	104.3(4)	C5C-C6C-C1C	113.2(4)
C12C-C6C-C1C	103.4(3)	C11C-C10C-C1C	105.1(3)
C11C-C10C-H10G	110.7	C1C-C10C-H10G	110.7
C11C-C10C-H10H	110.7	C1C-C10C-H10H	110.7
H10G-C10C-H10H	108.8	O1C-C11C-C10C	126.5(3)
O1C-C11C-C12C	124.4(4)	C10C-C11C-C12C	109.1(3)
C11C-C12C-C6C	105.3(3)	C11C-C12C-H12G	110.7
C6C-C12C-H12G	110.7	C11C-C12C-H12H	110.7
C6C-C12C-H12H	110.7	H12G-C12C-H12H	108.8
C8F-C7F-C6F	105.5(11)	C8F-C7F-H7F1	110.6

C6F-C7F-H7F1	110.6	C8F-C7F-H7F2	110.6
C6F-C7F-H7F2	110.6	H7F1-C7F-H7F2	108.8
N1C-C8F-C7F	115.2(13)	N1C-C8F-C9F	109.9(14)
C7F-C8F-C9F	106.9(12)	N1C-C8F-H8F	108.2
C7F-C8F-H8F	108.2	C9F-C8F-H8F	108.2
C1F-C9F-C8F	106.9(11)	C1F-C9F-H9F1	110.3
C8F-C9F-H9F1	110.3	C1F-C9F-H9F2	110.3
C8F-C9F-H9F2	110.3	H9F1-C9F-H9F2	108.6
C2F-C1F-C9F	113.6(12)	C2F-C1F-C10F	111.0(11)
C9F-C1F-C10F	113.6(13)	C2F-C1F-C6F	113.1(11)
C9F-C1F-C6F	103.4(11)	C10F-C1F-C6F	101.4(9)
C1F-C2F-C3F	114.8(13)	C1F-C2F-H2F1	108.6
C3F-C2F-H2F1	108.6	C1F-C2F-H2F2	108.6
C3F-C2F-H2F2	108.6	H2F1-C2F-H2F2	107.6
C2F-C3F-C4F	110.5(15)	C2F-C3F-H3F1	109.6
C4F-C3F-H3F1	109.6	C2F-C3F-H3F2	109.6
C4F-C3F-H3F2	109.6	H3F1-C3F-H3F2	108.1
C3F-C4F-C5F	108.9(15)	C3F-C4F-H4F1	109.9
C5F-C4F-H4F1	109.9	C3F-C4F-H4F2	109.9
C5F-C4F-H4F2	109.9	H4F1-C4F-H4F2	108.3
C6F-C5F-C4F	116.1(13)	C6F-C5F-H5F1	108.3
C4F-C5F-H5F1	108.3	C6F-C5F-H5F2	108.3
C4F-C5F-H5F2	108.3	H5F1-C5F-H5F2	107.4
C5F-C6F-C12F	112.2(12)	C5F-C6F-C7F	110.9(12)
C12F-C6F-C7F	112.7(12)	C5F-C6F-C1F	113.3(12)
C12F-C6F-C1F	103.8(10)	C7F-C6F-C1F	103.4(11)
C11F-C10F-C1F	105.8(10)	C11F-C10F-H10I	110.6
C1F-C10F-H10I	110.6	C11F-C10F-H10J	110.6
C1F-C10F-H10J	110.6	H10I-C10F-H10J	108.7
O1F-C11F-C10F	126.3(12)	O1F-C11F-C12F	125.0(12)
C10F-C11F-C12F	108.6(9)	C11F-C12F-C6F	105.8(10)
C11F-C12F-H12I	110.6	C6F-C12F-H12I	110.6

C11F-C12F-H12J	110.6	C6F-C12F-H12J	110.6
H12I-C12F-H12J	108.7	O2C-C13C-N1C	122.4(2)
O2C-C13C-N2C	123.5(2)	N1C-C13C-N2C	114.06(19)
C19C-C14C-C15C	119.5(2)	C19C-C14C-N2C	124.80(19)
C15C-C14C-N2C	115.65(19)	C16C-C15C-C14C	119.1(2)
C16C-C15C-H15C	120.5	C14C-C15C-H15C	120.5
F1C-C16C-C17C	118.9(2)	F1C-C16C-C15C	119.8(2)
C17C-C16C-C15C	121.3(2)	F2C-C17C-C16C	118.8(2)
F2C-C17C-C18C	121.0(2)	C16C-C17C-C18C	120.1(2)
C17C-C18C-C19C	119.9(2)	C17C-C18C-H18C	120.1
C19C-C18C-H18C	120.1	C14C-C19C-C18C	120.1(2)
C14C-C19C-H19C	119.9	C18C-C19C-H19C	119.9

Table S41. Torsion angles (°) for *syn*-**25**.

C10A-C1A-C2A-C3A	-70.0(3)	C6A-C1A-C2A-C3A	51.8(3)
C9A-C1A-C2A-C3A	164.1(3)	C1A-C2A-C3A-C4A	-58.3(4)
C2A-C3A-C4A-C5A	58.8(4)	C3A-C4A-C5A-C6A	-53.3(4)
C10A-C1A-C6A-C7A	-154.4(2)	C2A-C1A-C6A-C7A	78.5(3)
C9A-C1A-C6A-C7A	-38.1(2)	C10A-C1A-C6A-C5A	83.0(3)
C2A-C1A-C6A-C5A	-44.1(3)	C9A-C1A-C6A-C5A	-160.7(2)
C10A-C1A-C6A-C12A	-33.9(3)	C2A-C1A-C6A-C12A	-161.0(2)
C9A-C1A-C6A-C12A	82.3(2)	C4A-C5A-C6A-C7A	-72.5(3)
C4A-C5A-C6A-C1A	45.0(3)	C4A-C5A-C6A-C12A	158.1(3)
C1A-C6A-C7A-C8A	31.7(3)	C5A-C6A-C7A-C8A	153.0(2)
C12A-C6A-C7A-C8A	-80.5(3)	C13A-N1A-C8A-C9A	95.9(3)
C13A-N1A-C8A-C7A	-144.7(2)	C6A-C7A-C8A-N1A	-136.1(2)
C6A-C7A-C8A-C9A	-12.2(3)	N1A-C8A-C9A-C1A	110.5(2)
C7A-C8A-C9A-C1A	-11.9(3)	C10A-C1A-C9A-C8A	143.0(2)
C2A-C1A-C9A-C8A	-88.7(3)	C6A-C1A-C9A-C8A	30.7(3)
C2A-C1A-C10A-C11A	160.4(2)	C6A-C1A-C10A-C11A	34.9(3)
C9A-C1A-C10A-C11A	-74.7(3)	C1A-C10A-C11A-O1A	158.1(3)
C1A-C10A-C11A-C12A	-21.9(3)	O1A-C11A-C12A-C6A	-179.1(3)

C10A-C11A-C12A-C6A	0.9(3)	C7A-C6A-C12A-C11A	133.2(2)
C1A-C6A-C12A-C11A	19.9(3)	C5A-C6A-C12A-C11A	-98.3(3)
C8A-N1A-C13A-O2A	2.5(4)	C8A-N1A-C13A-N2A	-177.5(2)
O2A-C13A-N2A-C14D	21.4(14)	N1A-C13A-N2A-C14D	-158.6(14)
O2A-C13A-N2A-C14A	11.7(10)	N1A-C13A-N2A-C14A	-168.3(9)
C13A-N2A-C14A-C15A	-21.(2)	C13A-N2A-C14A-C19A	155.9(10)
C19A-C14A-C15A-C16A	0.(2)	N2A-C14A-C15A-C16A	176.7(13)
C14A-C15A-C16A-F1A	177.9(13)	C14A-C15A-C16A-C17A	-0.6(19)
F1A-C16A-C17A-F2A	-0.3(14)	C15A-C16A-C17A-F2A	178.3(10)
F1A-C16A-C17A-C18A	-175.9(9)	C15A-C16A-C17A-C18A	2.6(15)
F2A-C17A-C18A-C19A	-179.6(8)	C16A-C17A-C18A-C19A	-3.8(14)
C17A-C18A-C19A-C14A	3.3(18)	C15A-C14A-C19A-C18A	-1.(2)
N2A-C14A-C19A-C18A	-178.4(11)	C13A-N2A-C14D-C15D	-22.(3)
C13A-N2A-C14D-C19D	162.0(11)	N2A-C14D-C15D-C16D	-178.0(15)
C19D-C14D-C15D-C16D	-2.(3)	C14D-C15D-C16D-F1D	-178.5(16)
C14D-C15D-C16D-C17D	-1.(2)	F1D-C16D-C17D-C18D	-179.7(11)
C15D-C16D-C17D-C18D	3.1(19)	F1D-C16D-C17D-F2D	-1.6(17)
C15D-C16D-C17D-F2D	-178.8(12)	C16D-C17D-C18D-C19D	-1.5(17)
F2D-C17D-C18D-C19D	-179.6(10)	C17D-C18D-C19D-C14D	-2.(2)
N2A-C14D-C19D-C18D	179.6(14)	C15D-C14D-C19D-C18D	4.(3)
C1B-C2B-C3B-C4B	55.5(4)	C2B-C3B-C4B-C5B	-58.5(4)
C3B-C4B-C5B-C6B	55.2(3)	C13B-N1B-C8B-C7B	166.5(2)
C13B-N1B-C8B-C9B	-74.5(3)	C6B-C7B-C8B-N1B	144.2(2)
C6B-C7B-C8B-C9B	19.9(3)	N1B-C8B-C9B-C1B	-117.7(2)
C7B-C8B-C9B-C1B	3.4(3)	C3B-C2B-C1B-C10E	73.0(5)
C3B-C2B-C1B-C9B	-161.2(2)	C3B-C2B-C1B-C6B	-47.0(3)
C3B-C2B-C1B-C10B	69.7(5)	C8B-C9B-C1B-C10E	-147.5(6)
C8B-C9B-C1B-C2B	95.5(2)	C8B-C9B-C1B-C6B	-24.6(3)
C8B-C9B-C1B-C10B	-127.0(5)	C8B-C7B-C6B-C5B	-158.7(2)
C8B-C7B-C6B-C12E	72.3(6)	C8B-C7B-C6B-C12B	78.4(4)
C8B-C7B-C6B-C1B	-35.4(3)	C4B-C5B-C6B-C7B	71.4(3)
C4B-C5B-C6B-C12E	-150.9(5)	C4B-C5B-C6B-C12B	-170.0(5)

C4B-C5B-C6B-C1B	-46.8(3)	C10E-C1B-C6B-C7B	163.6(5)
C2B-C1B-C6B-C7B	-81.7(3)	C9B-C1B-C6B-C7B	36.8(3)
C10B-C1B-C6B-C7B	146.8(5)	C10E-C1B-C6B-C5B	-73.0(5)
C2B-C1B-C6B-C5B	41.7(3)	C9B-C1B-C6B-C5B	160.2(2)
C10B-C1B-C6B-C5B	-89.7(5)	C10E-C1B-C6B-C12E	36.8(6)
C2B-C1B-C6B-C12E	151.5(5)	C9B-C1B-C6B-C12E	-90.0(5)
C2B-C1B-C6B-C12B	167.2(5)	C9B-C1B-C6B-C12B	-74.3(5)
C10B-C1B-C6B-C12B	35.7(5)	C2B-C1B-C10B-C11B	-147.6(5)
C9B-C1B-C10B-C11B	82.2(6)	C6B-C1B-C10B-C11B	-24.0(7)
C1B-C10B-C11B-O1B	-172.8(8)	C1B-C10B-C11B-C12B	4.3(10)
O1B-C11B-C12B-C6B	-165.2(8)	C10B-C11B-C12B-C6B	17.9(9)
C7B-C6B-C12B-C11B	-145.5(5)	C5B-C6B-C12B-C11B	91.0(6)
C1B-C6B-C12B-C11B	-34.9(7)	C2B-C1B-C10E-C11E	-156.1(6)
C9B-C1B-C10E-C11E	83.0(7)	C6B-C1B-C10E-C11E	-35.4(7)
C1B-C10E-C11E-O1E	-166.0(11)	C1B-C10E-C11E-C12E	18.5(10)
O1E-C11E-C12E-C6B	-172.6(10)	C10E-C11E-C12E-C6B	3.3(12)
C7B-C6B-C12E-C11E	-134.4(7)	C5B-C6B-C12E-C11E	93.4(8)
C1B-C6B-C12E-C11E	-22.8(8)	C8B-N1B-C13B-O2B	6.5(4)
C8B-N1B-C13B-N2B	-173.6(2)	C14B-N2B-C13B-O2B	1.8(4)
C14B-N2B-C13B-N1B	-178.1(2)	C13B-N2B-C14B-C19B	151.3(2)
C13B-N2B-C14B-C15B	-30.7(4)	C19B-C14B-C15B-C16B	-2.4(3)
N2B-C14B-C15B-C16B	179.6(2)	C14B-C15B-C16B-F1B	-179.5(2)
C14B-C15B-C16B-C17B	1.4(4)	F1B-C16B-C17B-F2B	0.9(4)
C15B-C16B-C17B-F2B	-179.9(2)	F1B-C16B-C17B-C18B	-178.3(2)
C15B-C16B-C17B-C18B	0.9(4)	F2B-C17B-C18B-C19B	178.8(2)
C16B-C17B-C18B-C19B	-2.1(4)	C17B-C18B-C19B-C14B	1.0(4)
N2B-C14B-C19B-C18B	179.4(2)	C15B-C14B-C19B-C18B	1.3(4)
C13C-N1C-C8C-C7C	-74.6(4)	C13C-N1C-C8C-C9C	166.6(3)
C6C-C7C-C8C-N1C	-137.1(4)	C6C-C7C-C8C-C9C	-14.8(5)
N1C-C8C-C9C-C1C	114.5(4)	C7C-C8C-C9C-C1C	-8.8(5)
C8C-C9C-C1C-C10C	139.7(4)	C8C-C9C-C1C-C2C	-92.7(4)
C8C-C9C-C1C-C6C	28.2(5)	C9C-C1C-C2C-C3C	162.3(4)

C10C-C1C-C2C-C3C	-71.2(5)	C6C-C1C-C2C-C3C	48.3(5)
C1C-C2C-C3C-C4C	-54.4(6)	C2C-C3C-C4C-C5C	57.8(6)
C3C-C4C-C5C-C6C	-55.0(5)	C8C-C7C-C6C-C5C	156.3(4)
C8C-C7C-C6C-C12C	-78.9(5)	C8C-C7C-C6C-C1C	32.5(5)
C4C-C5C-C6C-C7C	-71.5(5)	C4C-C5C-C6C-C12C	161.8(4)
C4C-C5C-C6C-C1C	47.3(5)	C9C-C1C-C6C-C7C	-37.1(5)
C10C-C1C-C6C-C7C	-154.2(3)	C2C-C1C-C6C-C7C	81.0(4)
C9C-C1C-C6C-C5C	-161.2(4)	C10C-C1C-C6C-C5C	81.7(4)
C2C-C1C-C6C-C5C	-43.1(5)	C9C-C1C-C6C-C12C	81.0(4)
C10C-C1C-C6C-C12C	-36.0(4)	C2C-C1C-C6C-C12C	-160.9(3)
C9C-C1C-C10C-C11C	-82.2(4)	C2C-C1C-C10C-C11C	152.6(3)
C6C-C1C-C10C-C11C	28.1(4)	C1C-C10C-C11C-O1C	171.7(4)
C1C-C10C-C11C-C12C	-9.6(5)	O1C-C11C-C12C-C6C	165.8(4)
C10C-C11C-C12C-C6C	-13.0(5)	C7C-C6C-C12C-C11C	142.0(4)
C5C-C6C-C12C-C11C	-90.7(4)	C1C-C6C-C12C-C11C	30.0(5)
C13C-N1C-C8F-C7F	-65.9(18)	C13C-N1C-C8F-C9F	173.3(10)
C6F-C7F-C8F-N1C	-107.9(15)	C6F-C7F-C8F-C9F	15.(2)
N1C-C8F-C9F-C1F	135.0(15)	C7F-C8F-C9F-C1F	9.(2)
C8F-C9F-C1F-C2F	-151.8(16)	C8F-C9F-C1F-C10F	80.1(19)
C8F-C9F-C1F-C6F	-28.9(19)	C9F-C1F-C2F-C3F	69.6(19)
C10F-C1F-C2F-C3F	-160.9(14)	C6F-C1F-C2F-C3F	-47.8(18)
C1F-C2F-C3F-C4F	57.(2)	C2F-C3F-C4F-C5F	-57.(2)
C3F-C4F-C5F-C6F	53.(2)	C4F-C5F-C6F-C12F	71.9(19)
C4F-C5F-C6F-C7F	-161.1(15)	C4F-C5F-C6F-C1F	-45.2(19)
C8F-C7F-C6F-C5F	89.8(16)	C8F-C7F-C6F-C12F	-143.5(14)
C8F-C7F-C6F-C1F	-32.0(16)	C2F-C1F-C6F-C5F	40.6(16)
C9F-C1F-C6F-C5F	-82.7(16)	C10F-C1F-C6F-C5F	159.5(12)
C2F-C1F-C6F-C12F	-81.3(14)	C9F-C1F-C6F-C12F	155.4(14)
C10F-C1F-C6F-C12F	37.5(12)	C2F-C1F-C6F-C7F	160.8(12)
C9F-C1F-C6F-C7F	37.5(15)	C10F-C1F-C6F-C7F	-80.3(12)
C2F-C1F-C10F-C11F	85.9(14)	C9F-C1F-C10F-C11F	-144.6(13)
C6F-C1F-C10F-C11F	-34.5(14)	C1F-C10F-C11F-O1F	-159.4(18)

C1F-C10F-C11F-C12F	18.5(17)	O1F-C11F-C12F-C6F	-176.3(18)
C10F-C11F-C12F-C6F	5.7(18)	C5F-C6F-C12F-C11F	-149.9(14)
C7F-C6F-C12F-C11F	84.0(16)	C1F-C6F-C12F-C11F	-27.2(15)
C8F-N1C-C13C-O2C	-2.0(10)	C8C-N1C-C13C-O2C	-18.8(4)
C8F-N1C-C13C-N2C	179.9(10)	C8C-N1C-C13C-N2C	163.1(3)
C14C-N2C-C13C-O2C	5.5(4)	C14C-N2C-C13C-N1C	-176.5(2)
C13C-N2C-C14C-C19C	9.2(4)	C13C-N2C-C14C-C15C	-174.2(2)
C19C-C14C-C15C-C16C	0.5(3)	N2C-C14C-C15C-C16C	-176.2(2)
C14C-C15C-C16C-F1C	177.4(2)	C14C-C15C-C16C-C17C	-1.4(4)
F1C-C16C-C17C-F2C	1.3(3)	C15C-C16C-C17C-F2C	-179.9(2)
F1C-C16C-C17C-C18C	-177.4(2)	C15C-C16C-C17C-C18C	1.4(4)
F2C-C17C-C18C-C19C	-179.3(2)	C16C-C17C-C18C-C19C	-0.6(4)
C15C-C14C-C19C-C18C	0.2(3)	N2C-C14C-C19C-C18C	176.7(2)
C17C-C18C-C19C-C14C	-0.2(4)		

Table S42. Anisotropic atomic displacement parameters (Å^2) for *syn*-**25**.

The anisotropic atomic displacement factor exponent takes the form: $-2\pi^2[h^2 a^{*2} U_{11} + ... + 2 h k a^* b^* U_{12}]$

	U_{11}	U_{22}	U_{33}	U_{23}	U_{13}	U_{12}
N1A	0.0456(11)	0.0607(13)	0.1030(17)	0.0416(12)	0.0263(11)	0.0138(10)
O1A	0.0668(13)	0.0897(15)	0.155(2)	0.0545(15)	0.0346(14)	0.0253(11)
O2A	0.0441(8)	0.0562(9)	0.0738(11)	0.0185(8)	0.0185(8)	0.0112(7)
C1A	0.0572(13)	0.0513(12)	0.0509(12)	0.0214(10)	0.0036(10)	0.0101(10)
C2A	0.093(2)	0.0724(17)	0.0668(16)	0.0327(14)	0.0288(15)	0.0245(15)
C3A	0.087(2)	0.080(2)	0.104(2)	0.0391(18)	0.0436(19)	0.0155(17)
C4A	0.0612(16)	0.0671(17)	0.111(3)	0.0276(17)	0.0160(16)	0.0001(13)
C5A	0.0741(18)	0.0649(16)	0.0641(16)	0.0164(13)	-0.0005(13)	0.0036(13)
C6A	0.0588(13)	0.0592(14)	0.0480(12)	0.0219(10)	0.0084(10)	0.0113(11)
C7A	0.0716(16)	0.0747(17)	0.0699(16)	0.0406(14)	0.0091(13)	0.0152(13)
C8A	0.0515(13)	0.0554(14)	0.099(2)	0.0386(14)	0.0192(13)	0.0112(11)
C9A	0.0617(15)	0.0558(14)	0.0841(19)	0.0209(13)	-0.0058(13)	0.0084(12)
C10A	0.0729(17)	0.0623(15)	0.0691(16)	0.0283(13)	0.0027(13)	0.0180(13)

C11A	0.0512(14)	0.0551(14)	0.109(2)	0.0307(15)	0.0210(14)	0.0121(11)
C12A	0.0770(19)	0.089(2)	0.0733(18)	0.0348(16)	0.0303(15)	0.0131(16)
C13A	0.0448(12)	0.0622(14)	0.0564(13)	0.0310(11)	0.0137(10)	0.0192(10)
N2A	0.0503(12)	0.0744(14)	0.0613(12)	0.0345(11)	0.0211(10)	0.0259(11)
C14A	0.051(4)	0.065(4)	0.041(4)	0.024(3)	0.008(4)	0.030(3)
C15A	0.058(4)	0.056(3)	0.057(4)	0.027(2)	0.018(4)	0.020(3)
C16A	0.081(5)	0.059(4)	0.068(4)	0.027(3)	0.031(4)	0.020(3)
F1A	0.130(5)	0.059(2)	0.142(6)	0.025(3)	0.062(5)	0.008(3)
C17A	0.084(5)	0.058(3)	0.055(3)	0.024(3)	0.012(3)	0.025(4)
F2A	0.177(7)	0.060(3)	0.096(3)	0.027(2)	0.027(5)	0.052(4)
C18A	0.051(5)	0.062(4)	0.065(3)	0.020(3)	0.009(3)	0.020(3)
C19A	0.052(4)	0.077(4)	0.060(3)	0.019(3)	0.005(3)	0.024(3)
C14D	0.058(5)	0.080(5)	0.040(4)	0.025(4)	0.007(5)	0.032(3)
C15D	0.072(6)	0.070(5)	0.059(5)	0.026(3)	0.018(5)	0.028(4)
C16D	0.084(5)	0.070(4)	0.069(4)	0.028(3)	0.023(5)	0.026(4)
F1D	0.153(8)	0.071(4)	0.118(5)	0.035(3)	0.038(6)	0.015(5)
C17D	0.064(6)	0.051(4)	0.059(3)	0.023(3)	0.013(4)	0.016(4)
F2D	0.172(8)	0.073(4)	0.092(3)	0.022(3)	0.020(5)	0.057(5)
C18D	0.065(5)	0.068(5)	0.064(3)	0.021(3)	0.008(3)	0.030(4)
C19D	0.062(5)	0.070(4)	0.056(3)	0.022(3)	0.010(3)	0.032(4)
O2B	0.0406(8)	0.0718(11)	0.0854(12)	0.0461(9)	0.0162(8)	0.0151(7)
N1B	0.0402(10)	0.0625(12)	0.0620(11)	0.0304(9)	0.0145(9)	0.0139(9)
N2B	0.0394(10)	0.0723(13)	0.0739(13)	0.0429(11)	0.0169(9)	0.0186(9)
F1B	0.0775(11)	0.1335(17)	0.1422(18)	0.0842(15)	0.0537(12)	0.0420(11)
F2B	0.0747(10)	0.0827(11)	0.1088(13)	0.0567(10)	0.0269(9)	0.0061(8)
C2B	0.0726(17)	0.0843(19)	0.0560(14)	0.0298(14)	0.0068(13)	0.0008(15)
C3B	0.087(2)	0.113(3)	0.0684(18)	0.0428(18)	0.0215(16)	0.0125(19)
C4B	0.0588(15)	0.100(2)	0.0820(19)	0.0515(17)	0.0201(14)	0.0095(15)
C5B	0.0510(14)	0.0785(18)	0.0911(19)	0.0530(16)	0.0050(13)	-0.0025(12)
C7B	0.0509(13)	0.0642(15)	0.0588(14)	0.0244(12)	0.0051(11)	-0.0023(11)
C8B	0.0467(12)	0.0573(13)	0.0627(14)	0.0258(11)	0.0142(10)	0.0077(10)
C9B	0.0430(12)	0.0682(15)	0.0811(17)	0.0418(14)	0.0034(11)	0.0028(11)

C1B	0.0477(12)	0.0725(16)	0.0719(16)	0.0457(14)	-0.0031(11)	-0.0002(11)
C6B	0.0463(12)	0.0580(14)	0.0797(17)	0.0351(13)	0.0086(11)	0.0030(10)
C10B	0.058(3)	0.069(5)	0.067(5)	0.037(4)	0.000(3)	0.006(3)
C11B	0.060(3)	0.063(4)	0.078(5)	0.041(4)	0.009(4)	0.011(3)
O1B	0.099(4)	0.106(5)	0.139(7)	0.074(5)	0.030(4)	0.048(4)
C12B	0.064(3)	0.063(3)	0.079(5)	0.041(3)	0.009(4)	0.008(2)
C10E	0.053(3)	0.060(4)	0.068(4)	0.031(4)	0.007(3)	0.011(3)
C11E	0.056(3)	0.069(5)	0.078(5)	0.030(4)	0.007(4)	0.016(3)
O1E	0.105(5)	0.112(6)	0.158(8)	0.068(6)	0.011(5)	0.060(5)
C12E	0.069(4)	0.064(4)	0.076(5)	0.033(4)	0.017(4)	0.011(3)
C13B	0.0411(11)	0.0591(13)	0.0519(12)	0.0255(10)	0.0121(9)	0.0104(9)
C14B	0.0431(11)	0.0632(14)	0.0496(12)	0.0282(10)	0.0080(9)	0.0122(10)
C15B	0.0490(12)	0.0672(14)	0.0599(13)	0.0344(12)	0.0162(10)	0.0201(11)
C16B	0.0431(12)	0.0799(17)	0.0645(14)	0.0386(13)	0.0177(10)	0.0183(11)
C17B	0.0556(14)	0.0682(15)	0.0649(15)	0.0370(13)	0.0108(11)	0.0074(12)
C18B	0.0704(16)	0.0625(15)	0.0742(16)	0.0364(13)	0.0192(13)	0.0194(13)
C19B	0.0572(14)	0.0697(15)	0.0654(15)	0.0352(12)	0.0200(11)	0.0244(12)
O2C	0.0453(8)	0.0776(11)	0.0529(9)	0.0217(8)	0.0102(7)	0.0246(8)
F1C	0.0665(10)	0.1115(14)	0.0797(11)	0.0343(10)	-0.0093(8)	0.0308(9)
F2C	0.0913(11)	0.0873(11)	0.0526(8)	0.0291(8)	0.0002(7)	0.0150(9)
N2C	0.0406(9)	0.0571(11)	0.0503(10)	0.0203(8)	0.0111(8)	0.0172(8)
N1C	0.0440(10)	0.0712(13)	0.0499(11)	0.0177(9)	0.0100(8)	0.0181(9)
C7C	0.061(2)	0.082(2)	0.0507(17)	0.0289(17)	0.0109(14)	-0.0034(18)
C8C	0.049(2)	0.066(3)	0.0465(19)	0.0210(17)	0.0101(15)	0.0151(18)
C9C	0.056(3)	0.062(2)	0.057(2)	0.0301(16)	0.0139(17)	0.0099(19)
C1C	0.0557(18)	0.058(2)	0.0451(17)	0.0232(15)	0.0094(13)	0.0093(16)
C2C	0.087(2)	0.083(2)	0.059(2)	0.0230(18)	0.0105(17)	0.033(2)
C3C	0.129(5)	0.081(3)	0.082(3)	0.021(2)	0.018(3)	0.039(3)
C4C	0.157(4)	0.064(2)	0.087(3)	0.024(2)	0.004(3)	0.002(3)
C5C	0.122(3)	0.086(3)	0.066(2)	0.032(2)	-0.008(2)	-0.033(2)
C6C	0.060(2)	0.077(3)	0.048(2)	0.0309(18)	0.0039(16)	-0.0098(19)
C10C	0.0624(19)	0.083(2)	0.0489(17)	0.0301(15)	0.0120(14)	0.0128(16)

C11C	0.053(2)	0.079(2)	0.0511(19)	0.0295(17)	0.0082(17)	0.0009(18)
O1C	0.0597(16)	0.120(2)	0.0667(15)	0.0510(15)	0.0052(13)	0.0143(15)
C12C	0.0530(18)	0.119(3)	0.062(2)	0.043(2)	0.0136(15)	0.007(2)
C7F	0.060(6)	0.069(6)	0.058(6)	0.025(5)	0.015(5)	0.004(5)
C8F	0.053(7)	0.052(7)	0.055(7)	0.028(5)	0.004(5)	0.007(5)
C9F	0.051(8)	0.065(7)	0.048(6)	0.022(5)	0.017(5)	0.007(5)
C1F	0.055(6)	0.056(6)	0.046(6)	0.023(5)	0.015(4)	0.010(5)
C2F	0.081(7)	0.092(7)	0.075(7)	0.023(5)	0.018(5)	0.018(6)
C3F	0.105(9)	0.115(9)	0.102(9)	0.025(6)	0.022(6)	0.027(7)
C4F	0.077(8)	0.070(7)	0.086(8)	0.026(5)	0.021(6)	0.017(6)
C5F	0.101(8)	0.074(7)	0.091(7)	0.044(6)	0.001(5)	0.016(5)
C6F	0.062(8)	0.063(8)	0.060(8)	0.024(5)	0.001(5)	0.006(6)
C10F	0.076(6)	0.071(6)	0.057(6)	0.029(5)	0.004(5)	0.013(5)
C11F	0.061(7)	0.066(6)	0.054(6)	0.024(5)	0.004(5)	0.006(5)
O1F	0.085(8)	0.086(7)	0.091(8)	0.026(6)	-0.023(6)	0.010(6)
C12F	0.054(5)	0.057(6)	0.054(6)	0.018(5)	0.007(4)	0.004(5)
C13C	0.0383(10)	0.0463(11)	0.0499(12)	0.0172(9)	0.0078(9)	0.0076(9)
C14C	0.0427(10)	0.0385(10)	0.0497(11)	0.0163(9)	0.0060(9)	0.0087(8)
C15C	0.0448(12)	0.0520(12)	0.0588(13)	0.0179(10)	0.0061(10)	0.0125(10)
C16C	0.0519(13)	0.0530(13)	0.0610(14)	0.0207(11)	-0.0069(11)	0.0101(10)
C17C	0.0668(15)	0.0515(13)	0.0486(12)	0.0182(10)	0.0018(11)	0.0104(11)
C18C	0.0619(14)	0.0578(14)	0.0554(13)	0.0223(11)	0.0131(11)	0.0133(11)
C19C	0.0469(12)	0.0539(12)	0.0532(13)	0.0203(10)	0.0071(10)	0.0122(10)

Table S42. Hydrogen atomic coordinates and isotropic atomic displacement parameters (Å^2) for *syn*-**25**.

	x/a	y/b	z/c	U(eq)
H01	0.1891(14)	-0.199(2)	0.7084(19)	0.070(9)
H201	0.2362	-0.4380	0.7846	0.088
H202	0.1711	-0.3824	0.7312	0.088
H301	0.1557	-0.6165	0.6842	0.105
H302	0.0677	-0.5469	0.7203	0.105

H401	0.0399	-0.5055	0.6007	0.1
H402	0.0219	-0.6376	0.5728	0.1
H501	0.1208	-0.5799	0.4857	0.087
H502	0.1831	-0.6460	0.5313	0.087
H701	0.1493	-0.3632	0.5854	0.083
H702	0.2293	-0.3631	0.5222	0.083
H8A	0.3551	-0.2381	0.6275	0.079
H901	0.3566	-0.2840	0.7672	0.086
H902	0.4301	-0.3272	0.6986	0.086
H10A	0.4211	-0.4976	0.7300	0.082
H10B	0.3279	-0.6009	0.6712	0.082
H12A	0.3225	-0.5767	0.4814	0.093
H12B	0.3853	-0.4501	0.5265	0.093
H02	0.1754(14)	-0.0400(19)	0.7809(16)	0.050(7)
H15A	0.4089	0.1515	0.8117	0.065
H18A	0.1148	0.3236	0.8637	0.072
H19A	0.0978	0.1304	0.8170	0.078
H15D	0.3782	0.1504	0.7927	0.078
H18D	0.0723	0.2831	0.8695	0.079
H19D	0.0787	0.0932	0.8285	0.074
H03	0.2085(13)	0.262(2)	0.3179(15)	0.048(7)
H04	0.1564(13)	0.125(2)	0.3514(17)	0.058(7)
H211	0.2186	0.2458	0.1257	0.087
H212	0.1494	0.2436	0.0413	0.087
H311	0.3316	0.2632	0.0349	0.105
H312	0.2849	0.3671	0.0278	0.105
H411	0.4040	0.3687	0.1806	0.09
H412	0.4474	0.4320	0.1251	0.09
H511	0.3299	0.5540	0.1598	0.084
H512	0.3949	0.5569	0.2461	0.084
H711	0.2752	0.4987	0.3494	0.072
H712	0.3018	0.3863	0.2830	0.072

H8B	0.1044	0.4230	0.3304	0.066
H911	0.0504	0.2651	0.1659	0.074
H912	0.0156	0.3844	0.2005	0.074
H10C	0.1268	0.4712	0.0634	0.076
H10D	0.0207	0.4369	0.0922	0.076
H12D	0.1443	0.5807	0.3033	0.079
H12C	0.2388	0.6561	0.2866	0.079
H10E	0.1499	0.4305	0.0405	0.071
H10F	0.0330	0.3900	0.0524	0.071
H12E	0.1376	0.5951	0.2745	0.082
H12F	0.2295	0.6488	0.2406	0.082
H15B	-0.0787	0.1550	0.4140	0.065
H18B	0.0135	-0.1944	0.3647	0.078
H19B	0.1175	-0.0646	0.3322	0.072
H06	0.5263(16)	0.091(2)	0.3688(15)	0.052(7)
H05	0.4569(15)	0.060(2)	0.2414(16)	0.051(7)
H7C1	0.2805	-0.1002	0.1902	0.078
H7C2	0.1787	-0.0426	0.1908	0.078
H8C	0.2721	0.1187	0.2032	0.064
H9C1	0.3165	0.0984	0.0790	0.067
H9C2	0.4264	0.0699	0.1108	0.067
H2C1	0.4557	-0.1203	0.0118	0.092
H2C2	0.4360	-0.1160	0.1023	0.092
H3C1	0.4250	-0.3024	0.0126	0.119
H3C2	0.3402	-0.2921	-0.0590	0.119
H4C1	0.2498	-0.3792	0.0196	0.13
H4C2	0.3017	-0.2756	0.1071	0.13
H5C1	0.1295	-0.2595	0.0662	0.117
H5C2	0.1497	-0.2708	-0.0258	0.117
H10G	0.3101	-0.0253	-0.0660	0.076
H10H	0.2719	-0.1575	-0.0963	0.076
H12G	0.0567	-0.1170	0.0176	0.092

H12H	0.1219	0.0080	0.0693	0.092
H7F1	0.1665	0.0350	0.1500	0.075
H7F2	0.2192	-0.0292	0.2032	0.075
H8F	0.3148	0.1584	0.1962	0.063
H9F1	0.4024	0.1167	0.0904	0.065
H9F2	0.4460	0.0196	0.1121	0.065
H2F1	0.3993	-0.0729	-0.0578	0.102
H2F2	0.2911	-0.1606	-0.0903	0.102
H3F1	0.4643	-0.1574	0.0335	0.134
H3F2	0.4292	-0.2499	-0.0620	0.134
H4F1	0.3628	-0.3190	0.0366	0.094
H4F2	0.2672	-0.3155	-0.0305	0.094
H5F1	0.2289	-0.2330	0.1072	0.104
H5F2	0.3407	-0.1517	0.1415	0.104
H10I	0.2095	0.0861	0.0319	0.081
H10J	0.2362	0.0232	-0.0607	0.081
H12I	0.1235	-0.2221	-0.0283	0.068
H12J	0.0677	-0.1444	0.0435	0.068
H15C	0.6466	0.1179	0.4872	0.064
H18C	0.3792	0.1882	0.6461	0.07
H19C	0.3449	0.1607	0.5019	0.062

Table S43. Hydrogen bond distances (Å) and angles (°) for *syn*-**25**.

	Donor-H	Acceptor-H	Donor-Acceptor	Angle
N1B-H03···O2C	0.806(14)	2.314(17)	3.040(3)	150.(2)
N2B-H04···O2C	0.803(14)	2.029(16)	2.796(2)	160.(3)
N1A-H01···O2B	0.803(14)	2.107(17)	2.867(3)	158.(3)
N2A-H02···O1C	0.800(14)	2.33(2)	2.932(4)	133.(2)
N1C-H05···O2A	0.802(13)	2.201(17)	2.928(3)	151.(2)
N2C-H06···O2A	0.800(14)	2.126(16)	2.894(2)	161.(2)

3.7. References for X-ray crystallography

(1) Hooft, R. W. W., Nonius B. V. *COLLECT, Program for Collecting Data on CCD Area Detectors*, **1998**, Delft, The Netherlands.

(2) Otwinowski, Z.; Minor, W. *Methods Enzymol.* **1997**, 276, 307 – 326.

(3) Otwinowski, Z.; Borek, D.; Majewski, W.; Minor, W. *Acta Crystallogr. Sect. A* **2003**, 59, 228 – 234.

(4) Sheldrick, G. M. *SHELXT – Integrated space-group and crystal-structure determination*, *Acta Cryst.,* **2015**, *A71*, 3-8.

(5) Sheldrick, G.M. *Crystal structure refinement with SHELXL*, *Acta Cryst.*, **2015**, *C71 (1)*, 3-8.

(6) *XP – Interactive molecular graphics, Version 5.1,* Bruker AXS Inc., Madison, Wisconsin, USA, **1998**.

4. Molecular Dynamics study to determine the binding mode of 18

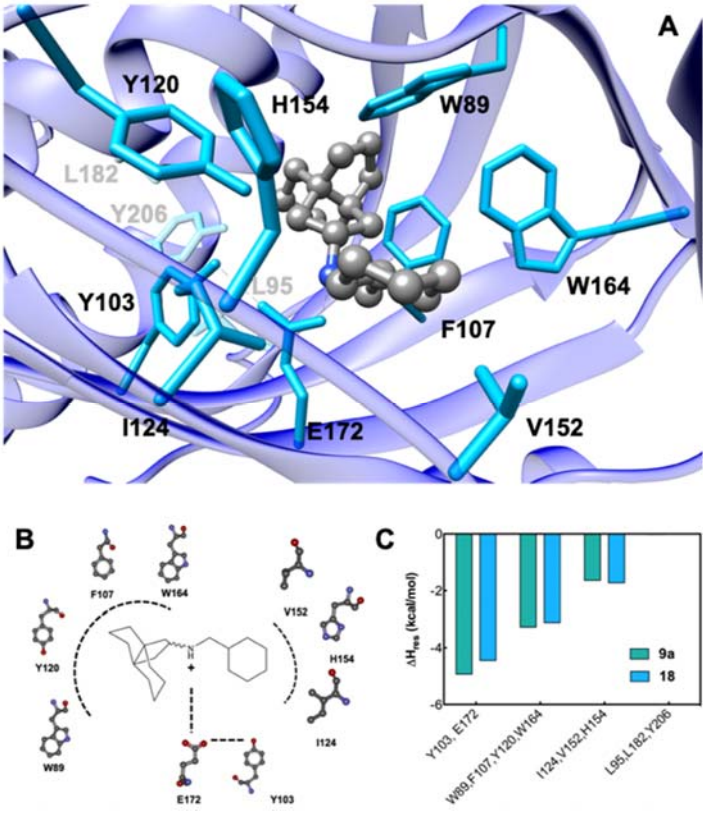

Figure S1. (A) Details of an equilibrated MD snapshot of **18** in the binding pocket of the σ_1 receptor. The compounds are shown as atom-colored sticks-and-balls (C, grey, N, blue, O, red) while the side chains of σ_1 residues mainly interacting with the ligands are depicted as colored sticks and labeled. Hydrogen atoms, water molecules, ions, and counter ions are omitted for clarity. (B) 2D schematic representation of the general stabilizing interactions between **18** and σ_1 receptor (C) Per-residue binding free energy decomposition (□H_{res}) of the main involved amino acids in σ_1/**9a** (light sea green) and σ_1/**18** (deep sky blue) complexes.

5. NMR spectra

NOESY spectrum (crucial part of oxocarbamate *syn*-**7**.

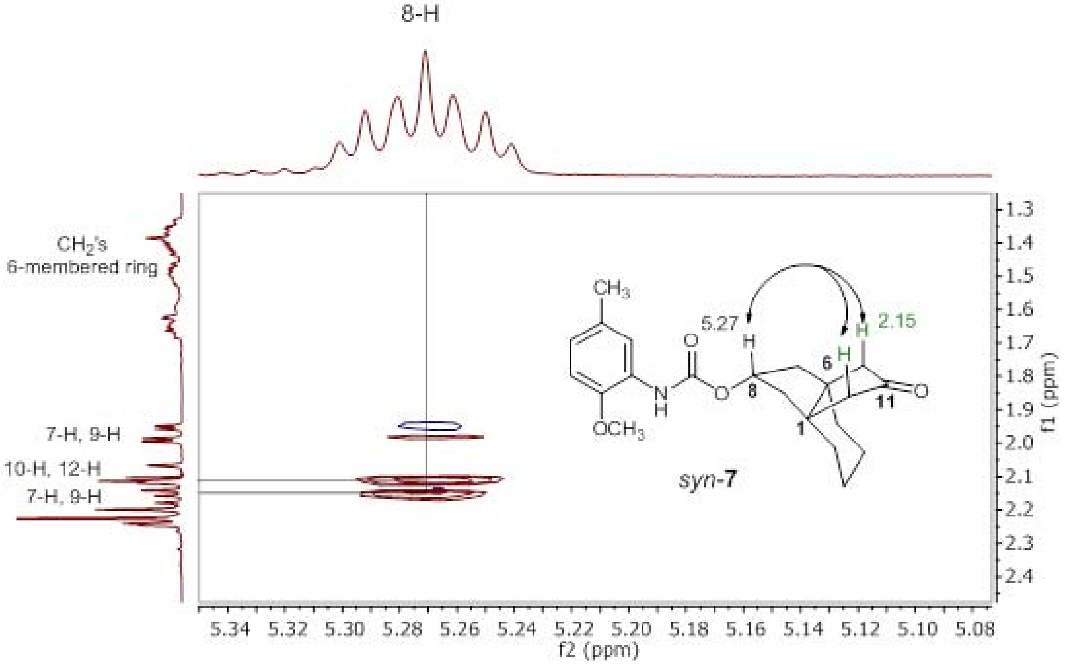

NOESY spectrum (crucial part of oxocarbamate *anti*-**7**.

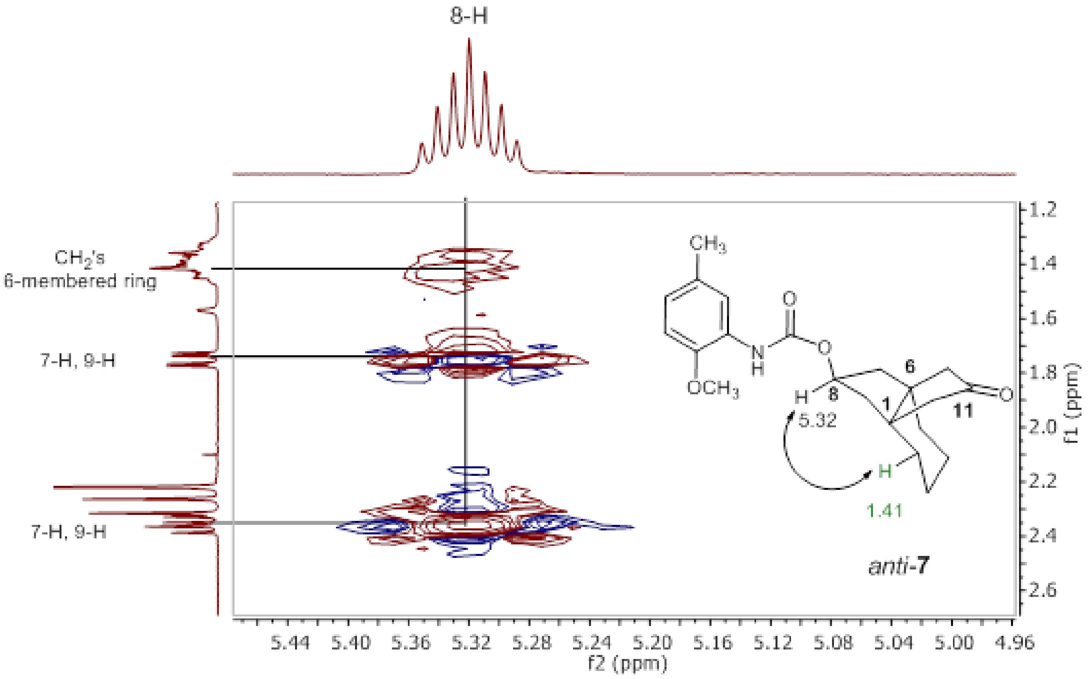

NOESY spectrum (crucial part of benzylaminocarbamate *anti,anti*-**4a**.

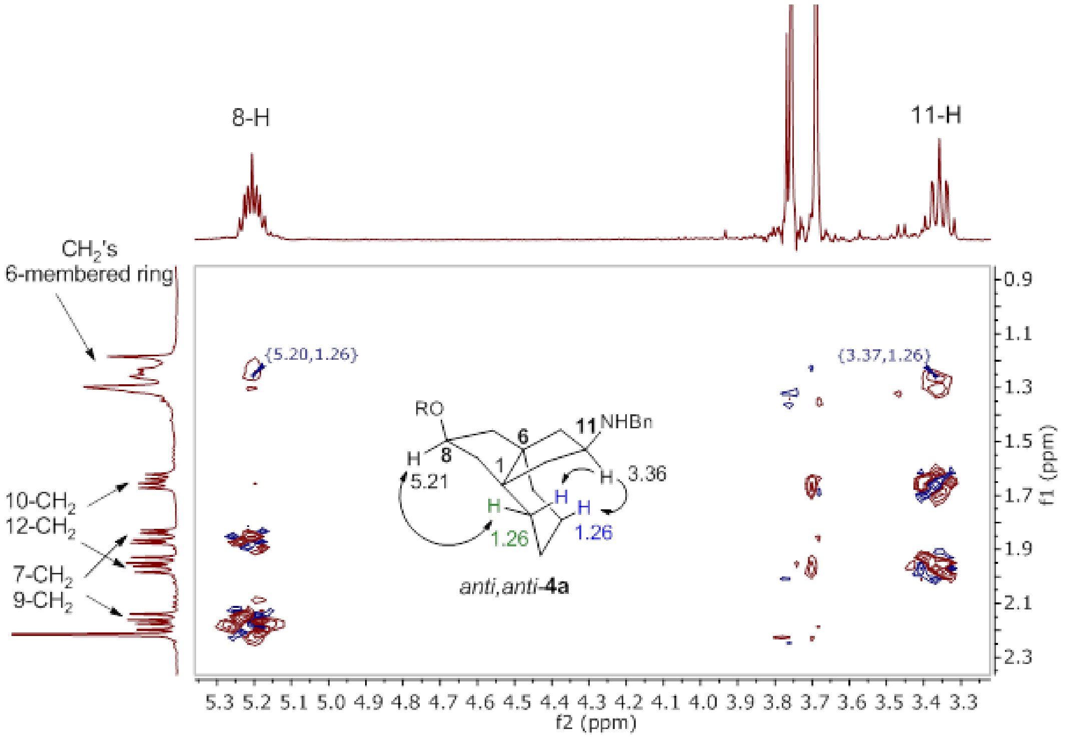

NOESY spectrum (crucial part of benzylaminocarbamate *anti,syn*-**4a**.

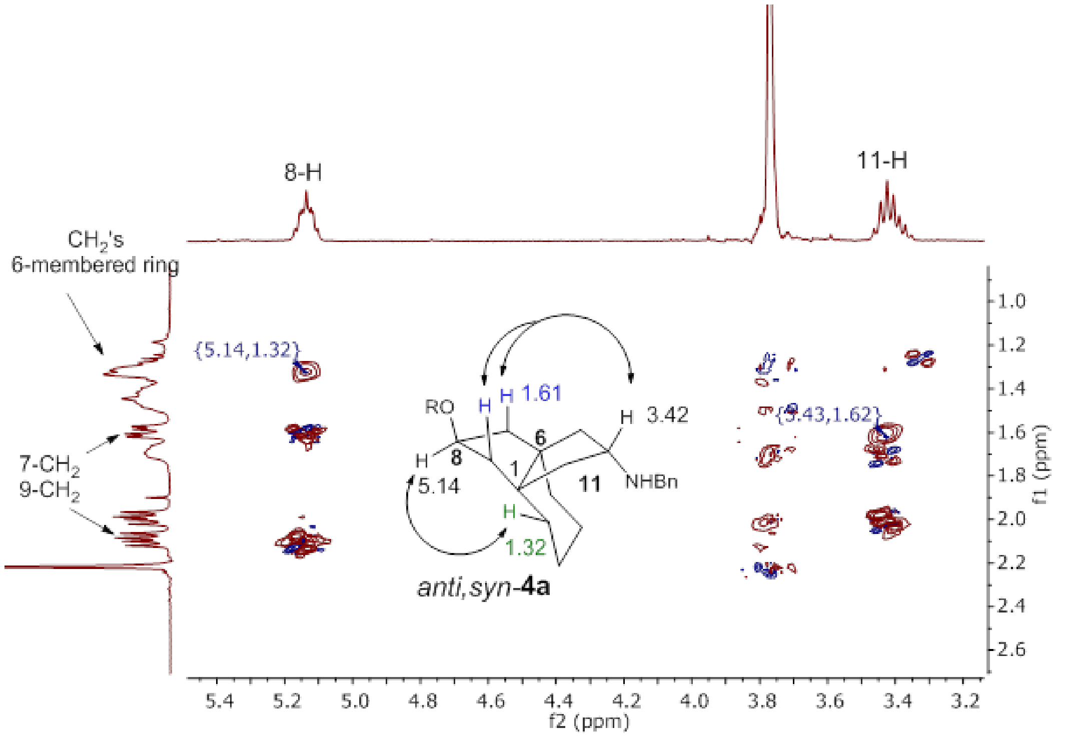

NOESY spectrum (crucial part of benzylaminocarbamate *syn,syn*-**4a**.

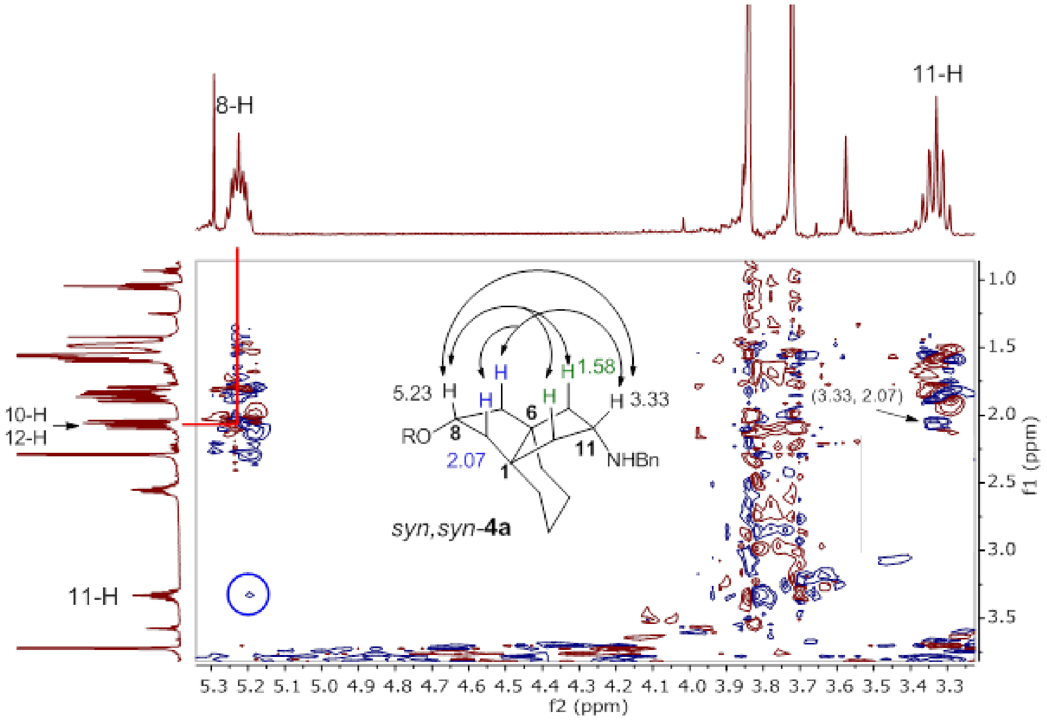

NOESY spectrum (crucial part of benzylaminocarbamate *syn,anti*-**4a**.

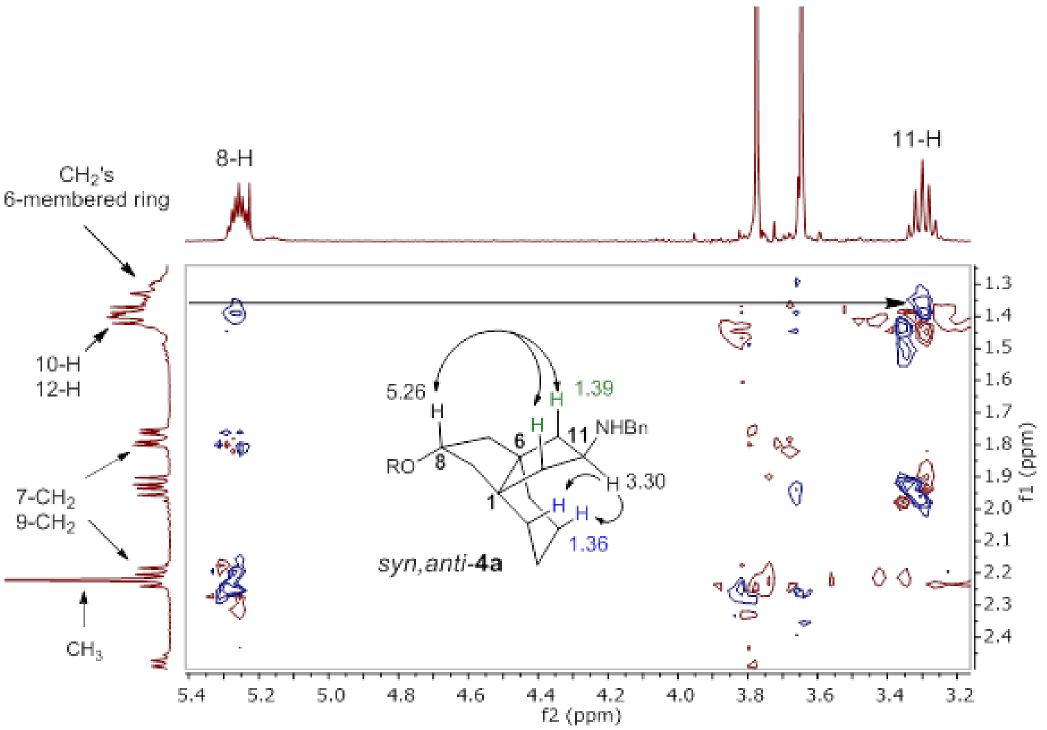

^{1}H NMR spectrum of [(8-*anti*-11-*anti* and 8-*anti*-11-*syn*)-11-(3,4-Dimethoxbenzylamino)[4.3.3]propellan-8-yl] N-(2-methoxy-5-methylphenyl)carbamate (8-*anti*-**4b**)

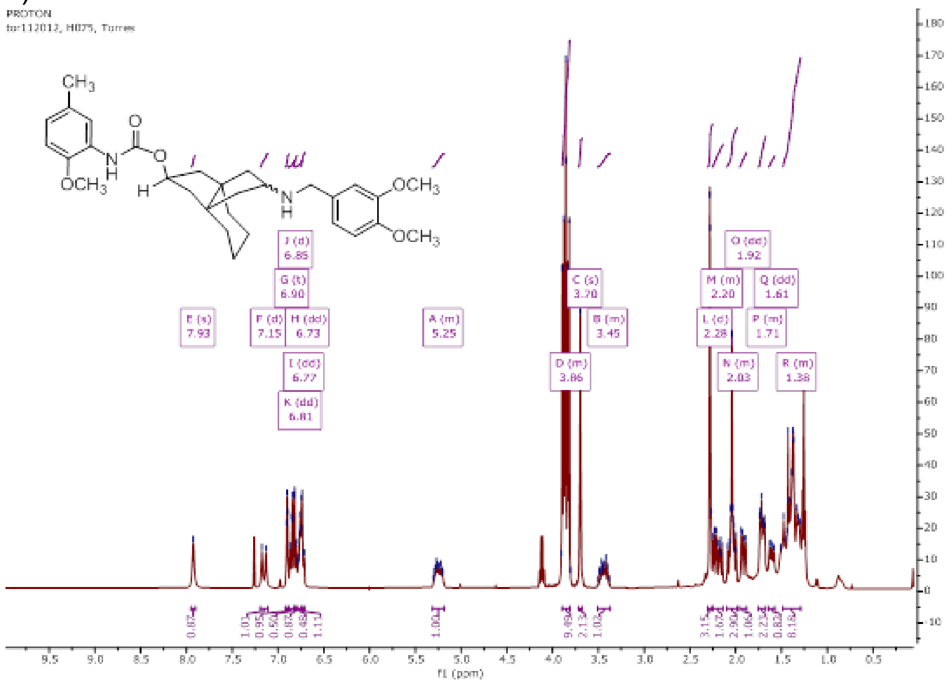

HSQC NMR spectrum of [(8-*anti*-11-*anti* and 8-*anti*-11-*syn*)-11-(3,4-Dimethoxbenzylamino)[4.3.3]propellan-8-yl] N-(2-methoxy-5-methylphenyl)carbamate (8-*anti*-**4b**)

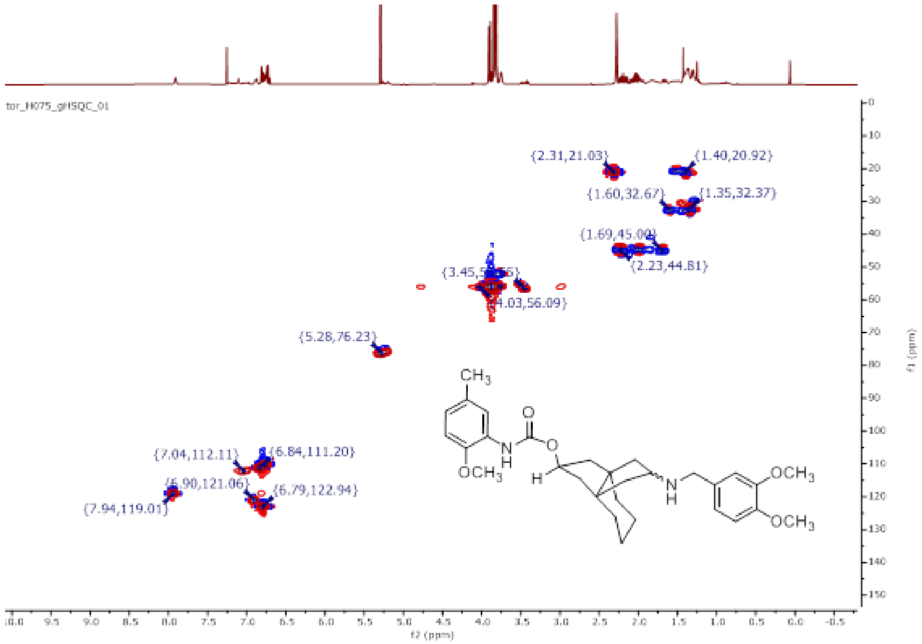

^{1}H NMR spectrum of [(8-*anti*-11-*anti* and 8-*anti*-11-*syn*)-11-(4-Chlorobenzylamino)[4.3.3]propellan-8-yl] N-(2-methoxy-5-methylphenyl)carbamate (8-*anti*-**4c**)

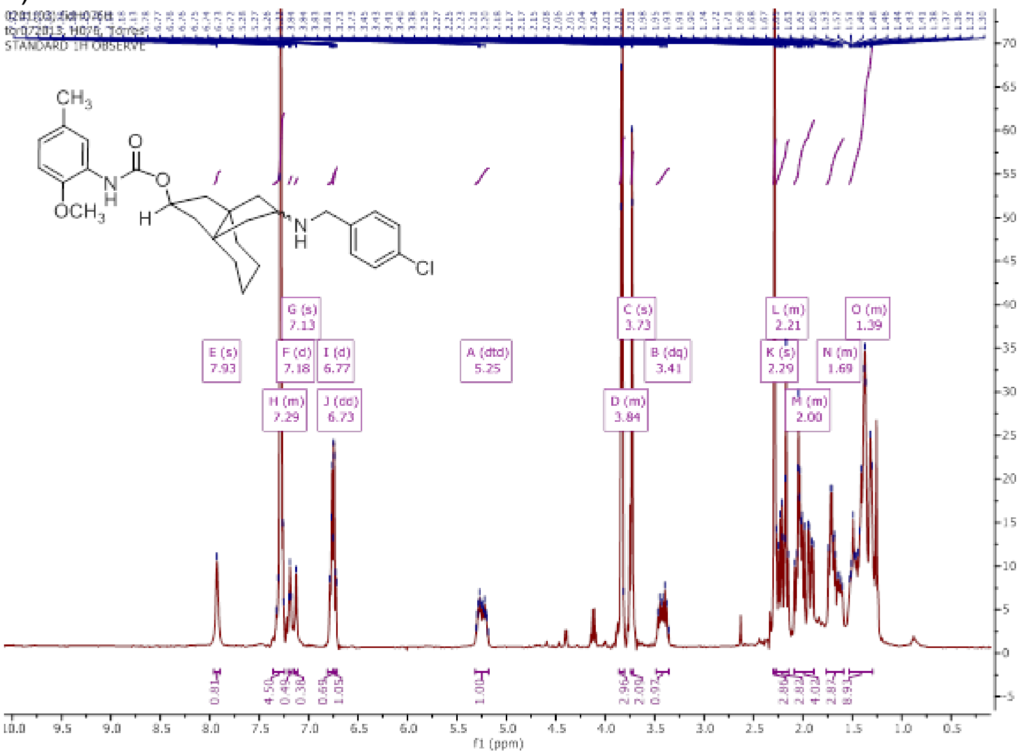

^{13}C NMR spectrum of [(8-*anti*-11-*anti* and 8-*anti*-11-*syn*)-11-(4-Chlorobenzylamino)[4.3.3]propellan-8-yl] N-(2-methoxy-5-methylphenyl)carbamate (8-*anti*-**4c**)

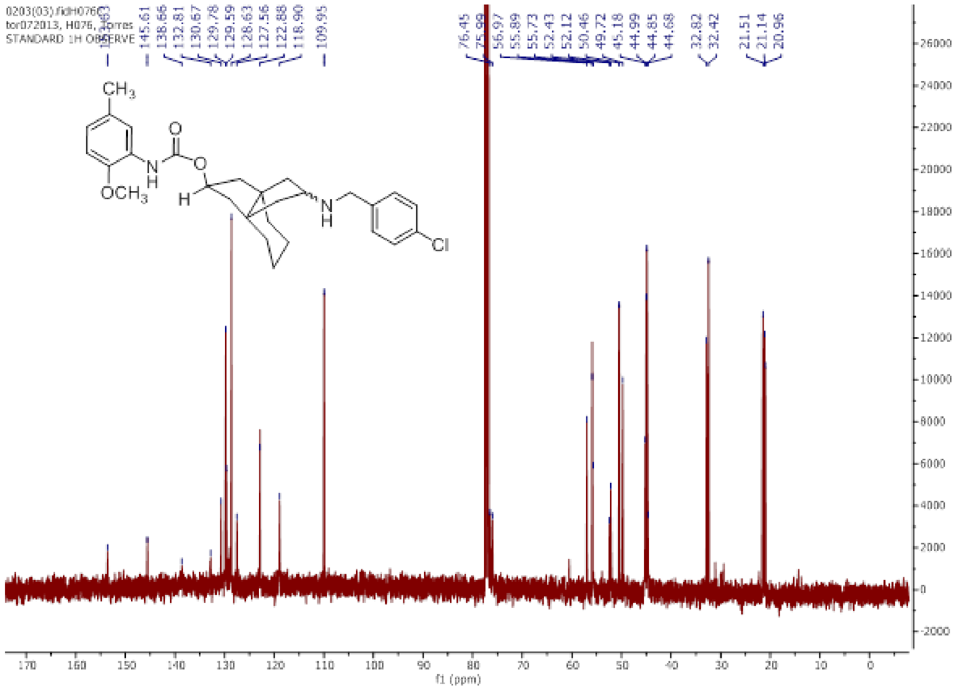

^{1}H NMR spectrum of [(8-*anti*-11-*anti* and 8-*anti*-11-*syn*)-11-(3,4-Dichlorobenzylamino)[4.3.3]propellan-8-yl] N-(2-methoxy-5-methylphenyl)carbamate (8-*anti*-**4d**)

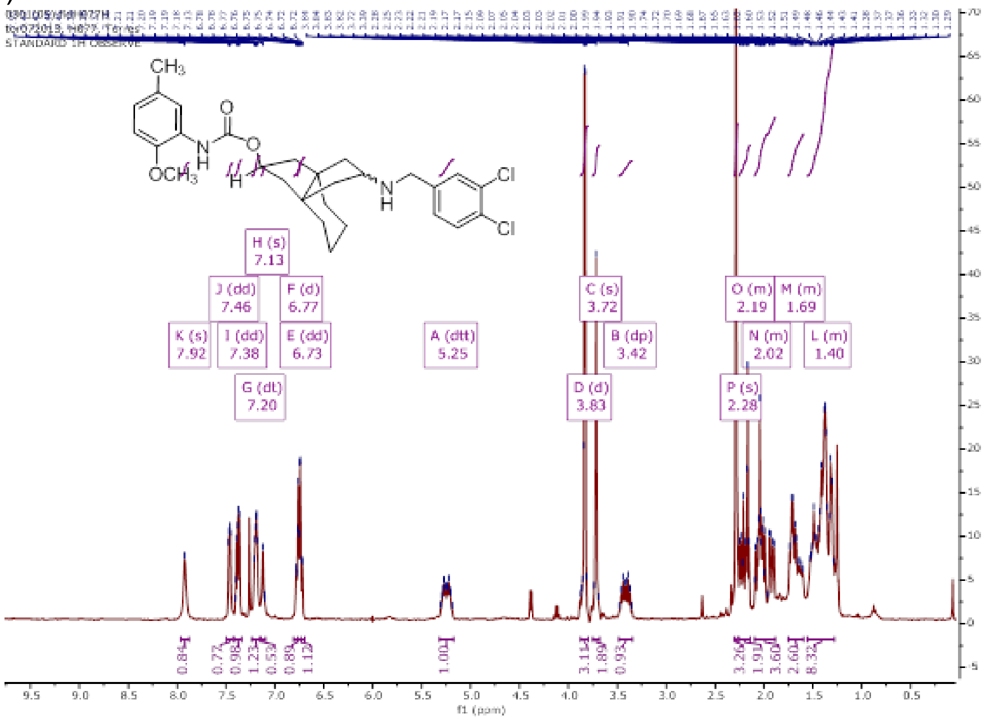

^{13}C NMR spectrum of [(8-*anti*-11-*anti* and 8-*anti*-11-*syn*)-11-(3,4-Dichlorobenzylamino)[4.3.3]propellan-8-yl] N-(2-methoxy-5-methylphenyl)carbamate (8-*anti*-**4d**)

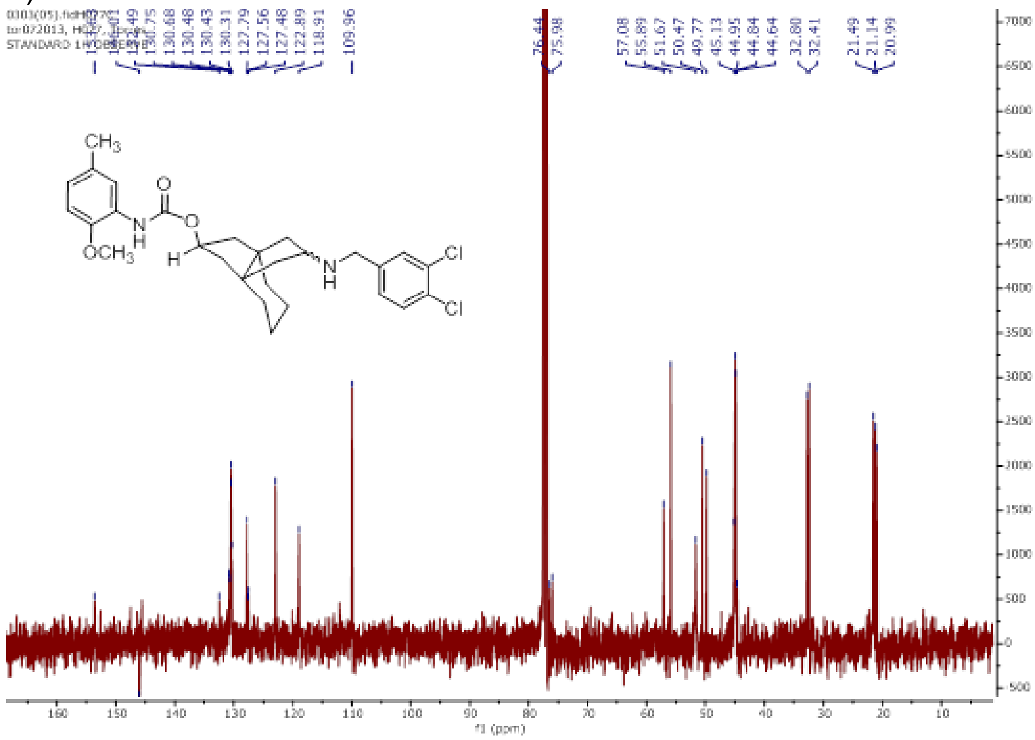

^{1}H NMR spectrum of [(8-*anti*-11-*anti* and 8-*anti*-11-*syn*)-11-(2,4-Dimethylbenzylamino)[4.3.3]propellan-8-yl] N-(2-methoxy-5-methylphenyl)carbamate (8-*anti*-**4e**)

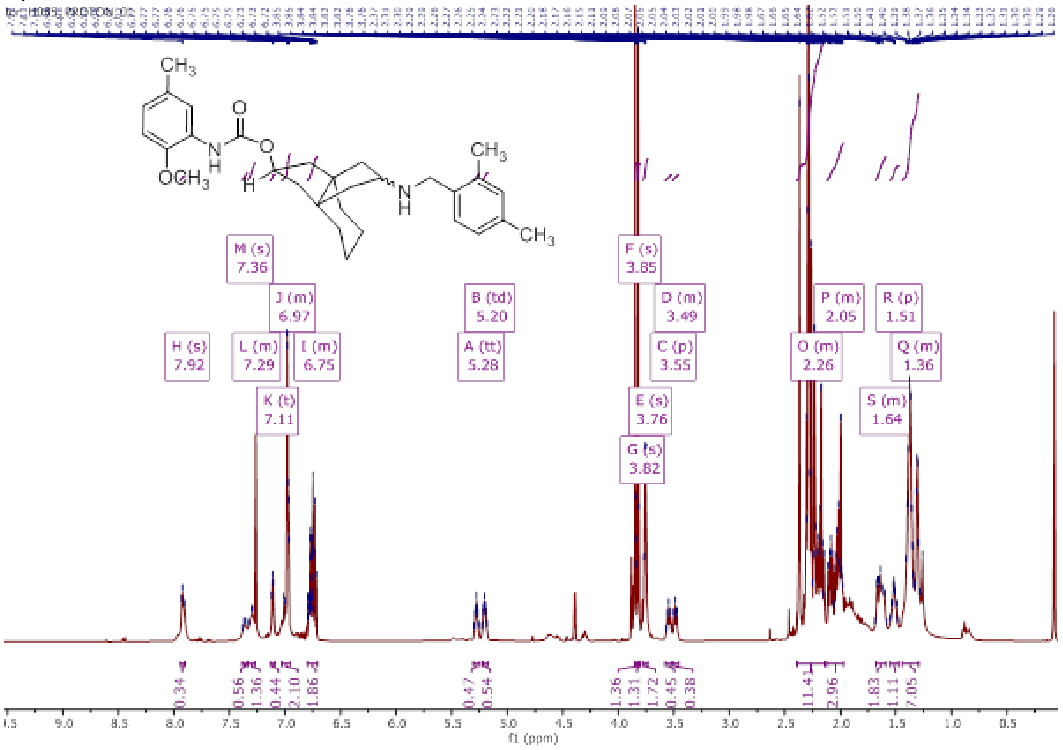

^{13}C NMR spectrum of [(8-*anti*-11-*anti* and 8-*anti*-11-*syn*)-11-(2,4-Dimethylbenzylamino)[4.3.3]propellan-8-yl] N-(2-methoxy-5-methylphenyl)carbamate (8-*anti*-**4e**)

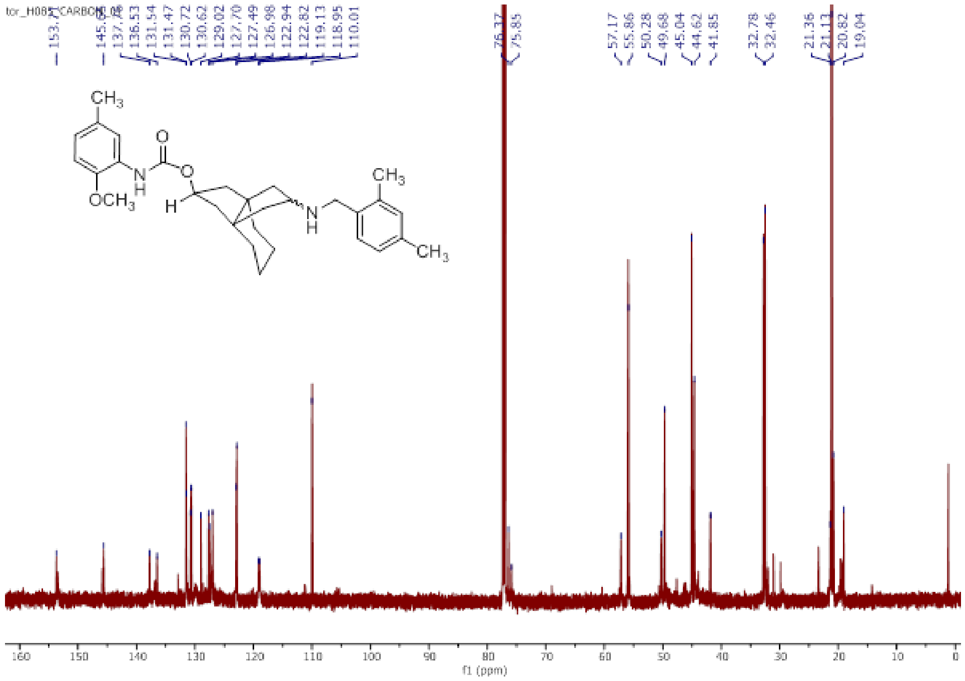

^{1}H NMR spectrum of [(8-*anti*-11-*anti* and 8-*anti*-11-*syn*)-11-[(3,5-Bis(trifluoromethyl)benzylamino][4.3.3]propellan-8-yl] N-(2-methoxy-5-methylphenyl)carbamate (8-*anti*-**4f**)

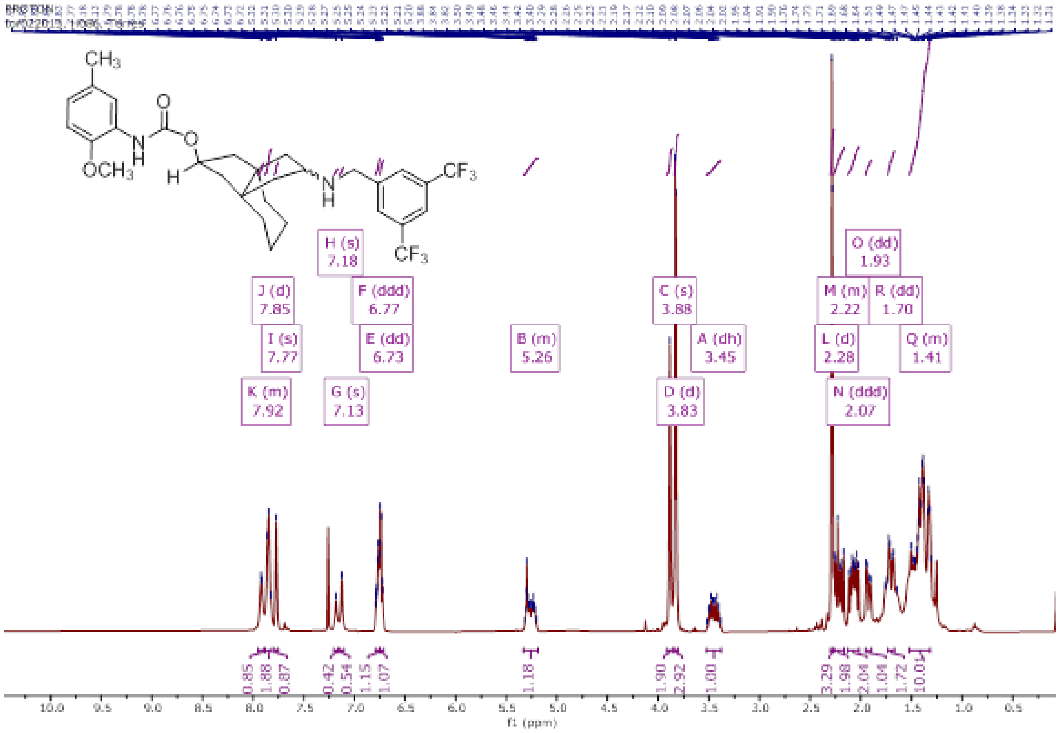

^{13}C NMR spectrum of [(8-*anti*-11-*anti* and 8-*anti*-11-*syn*)-11-[(3,5-Bis(trifluoromethyl)benzylamino][4.3.3]propellan-8-yl] N-(2-methoxy-5-methylphenyl)carbamate (8-*anti*-**4f**)

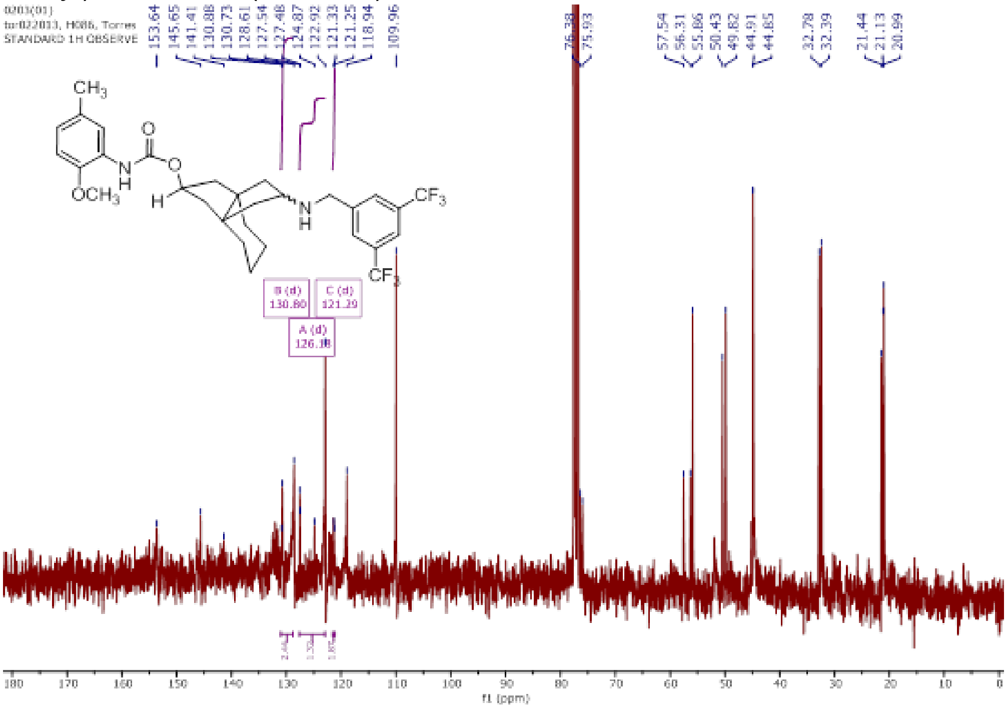

HSQC NMR spectrum of [(8-*anti*-11-*anti* and 8-*anti*-11-*syn*)-11-[(3,5-Bis(trifluoromethyl)benzylamino][4.3.3]propellan-8-yl] N-(2-methoxy-5-methylphenyl)carbamate (8-*anti*-**4f**)

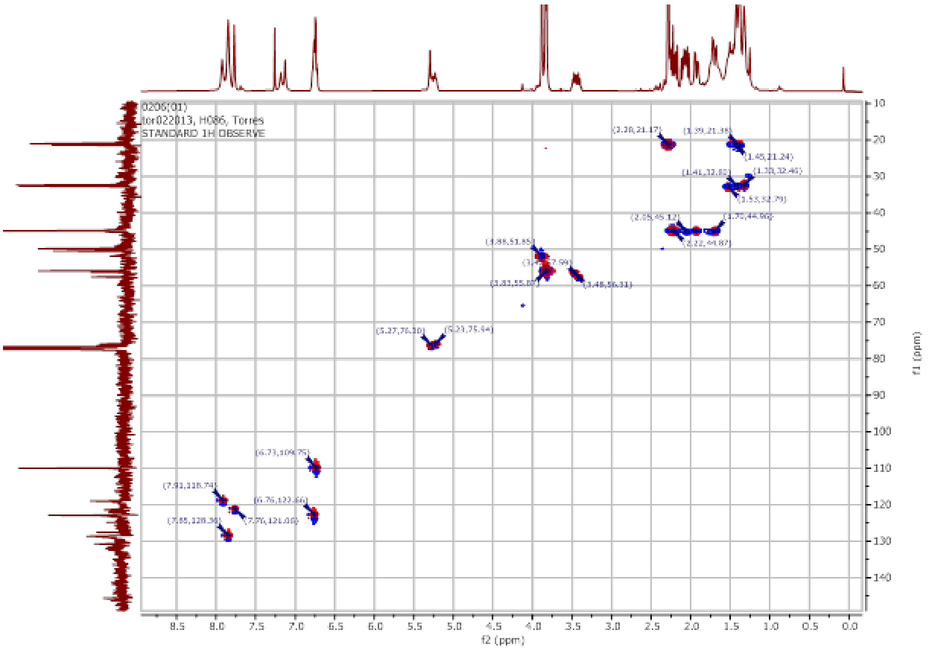

^{1}H NMR spectrum of [(8-*anti*-11-*anti* and 8-*anti*-11-*syn*)-11-(4-Nitrobenzylamino)[4.3.3]propellan-8-yl] N-(2-methoxy-5-methylphenyl)carbamate (8-*anti*-**4g**)

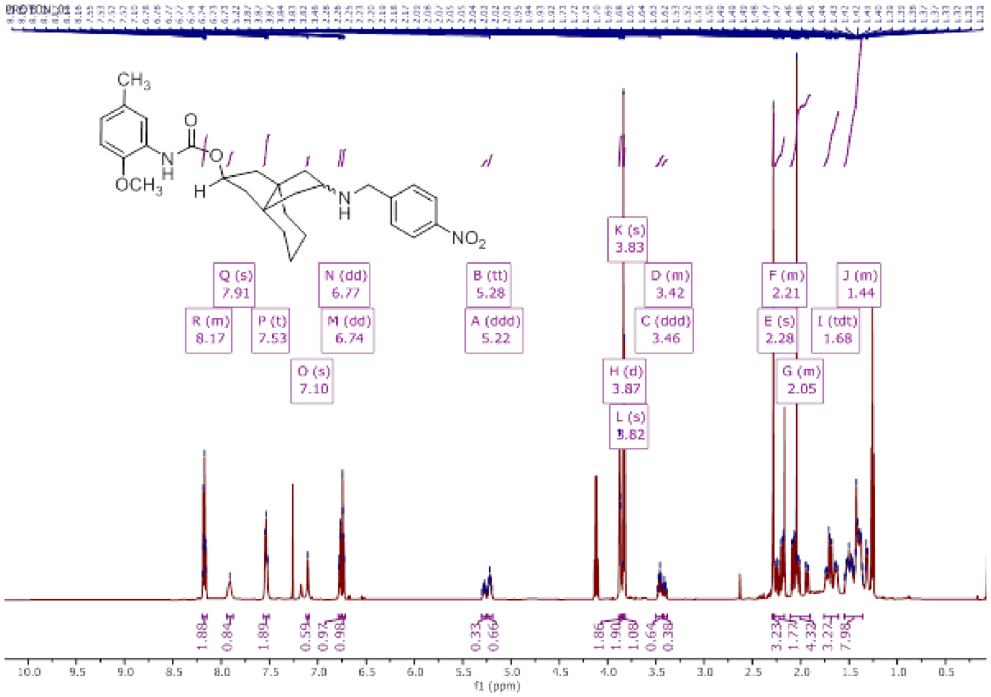

^{13}C NMR spectrum of [(8-*anti*-11-*anti* and 8-*anti*-11-*syn*)-11-(4-Nitrobenzylamino)[4.3.3]propellan-8-yl] N-(2-methoxy-5-methylphenyl)carbamate (8-*anti*-**4g**)

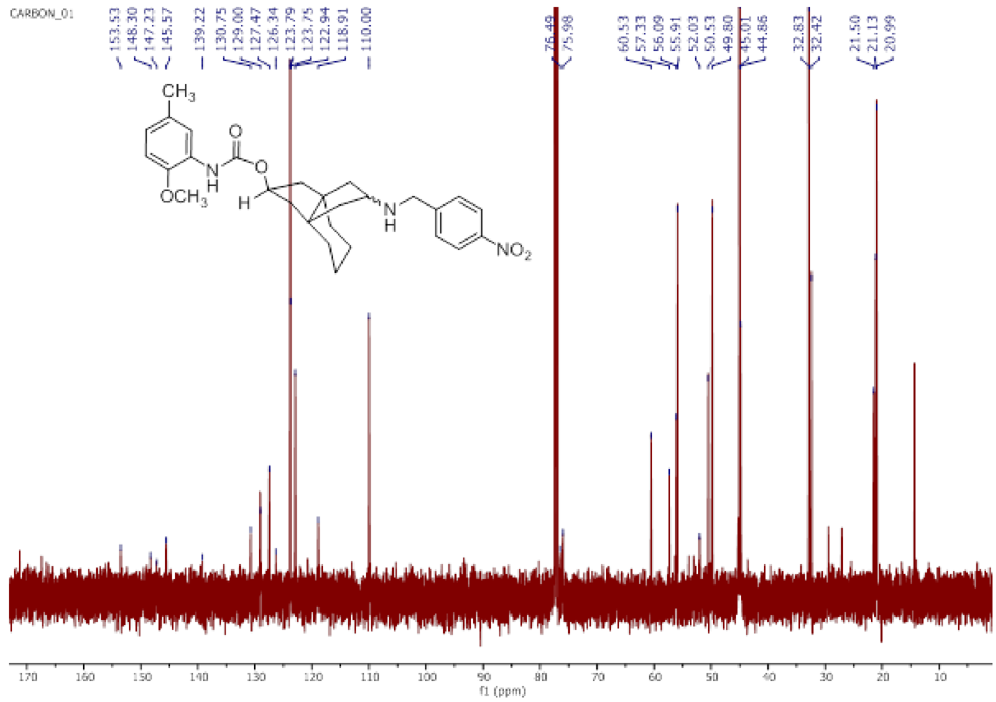

HSQC NMR spectrum of [(8-*anti*-11-*anti* and 8-*anti*-11-*syn*)-11-(4-Nitrobenzylamino)[4.3.3]propellan-8-yl] N-(2-methoxy-5-methylphenyl)carbamate (8-*anti*-**4g**)

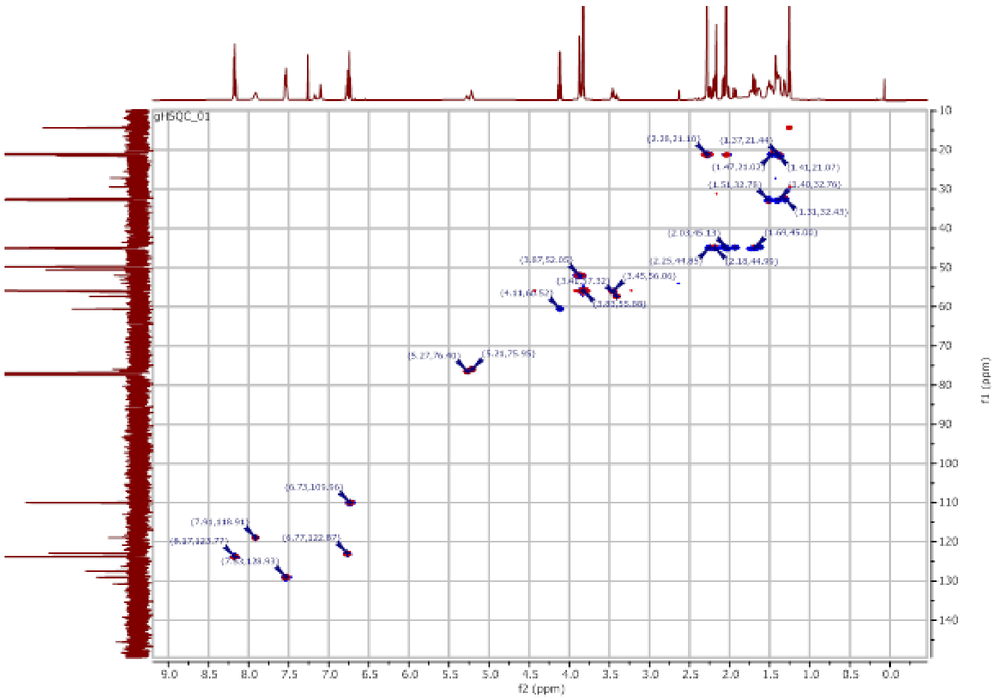

^{1}H NMR spectrum of [(8-*anti*-11-*anti* and 8-*anti*-11-*syn*)-11-[(4-Dimethylamino)benzylamino][4.3.3]propellan-8-yl] N-(2-methoxy-5-methylphenyl)carbamate (8-*anti*-**4h**)

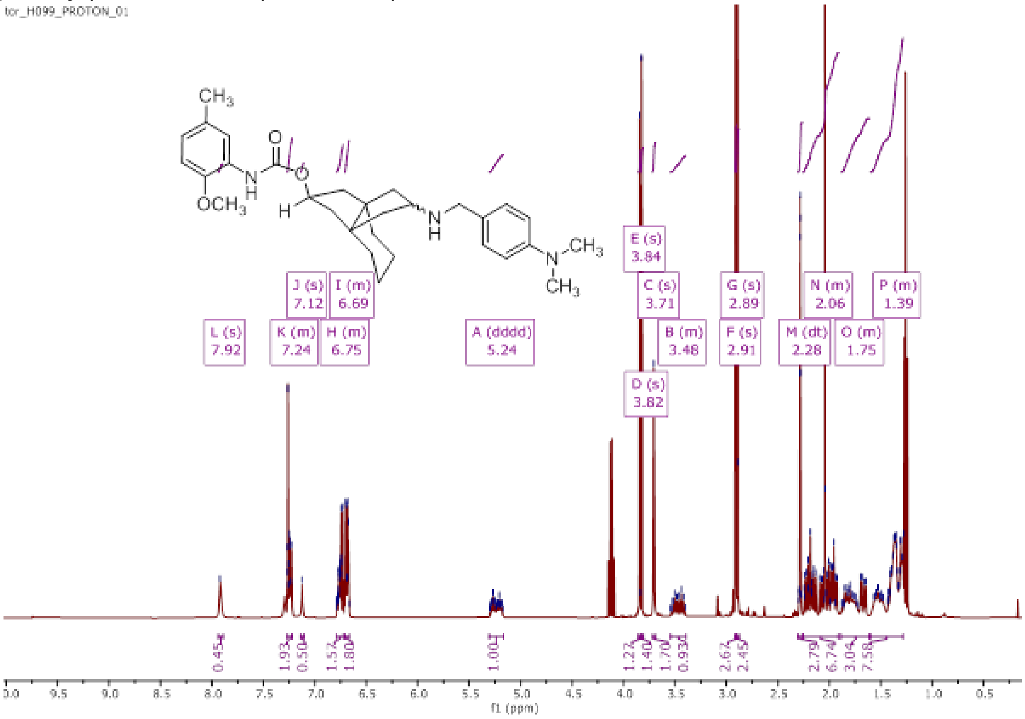

^{13}C NMR spectrum of [(8-*anti*-11-*anti* and 8-*anti*-11-*syn*)-11-[(4-Dimethylamino)benzylamino][4.3.3]propellan-8-yl] N-(2-methoxy-5-methylphenyl)carbamate (8-*anti*-**4h**)

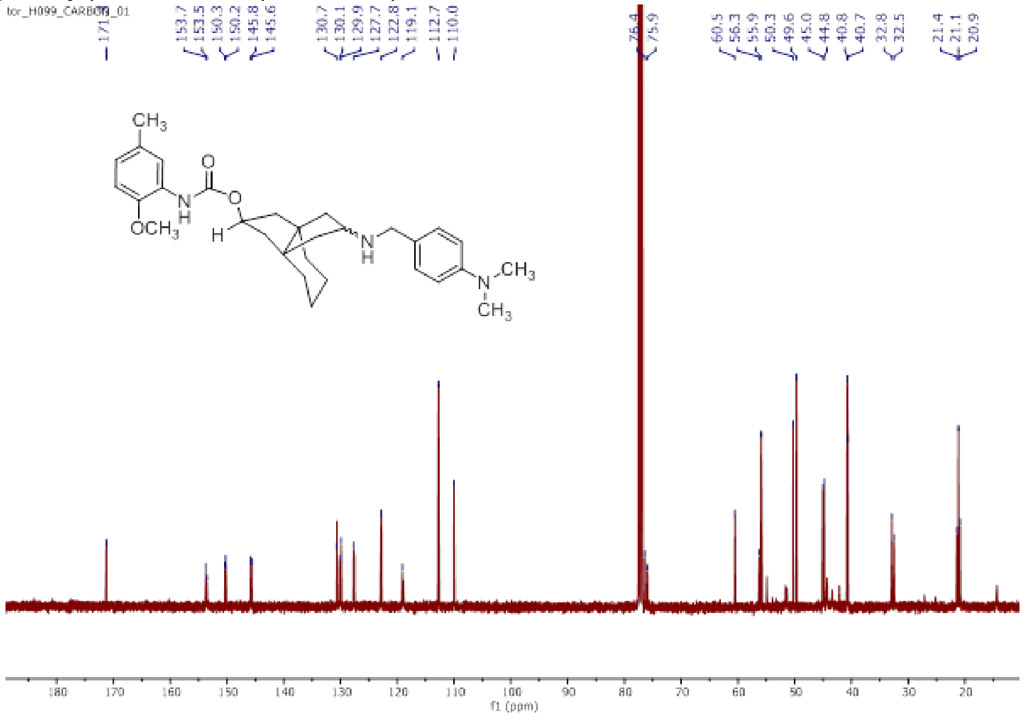

^{1}H NMR spectrum of [(8-*anti*-11-*anti* and 8-*anti*-11-*syn*)-11-[(Furan-2-yl-mehtyl)amino][4.3.3]propellan-8-yl] N-(2-methoxy-5-methylphenyl)carbamate (8-*anti*-**4i**)

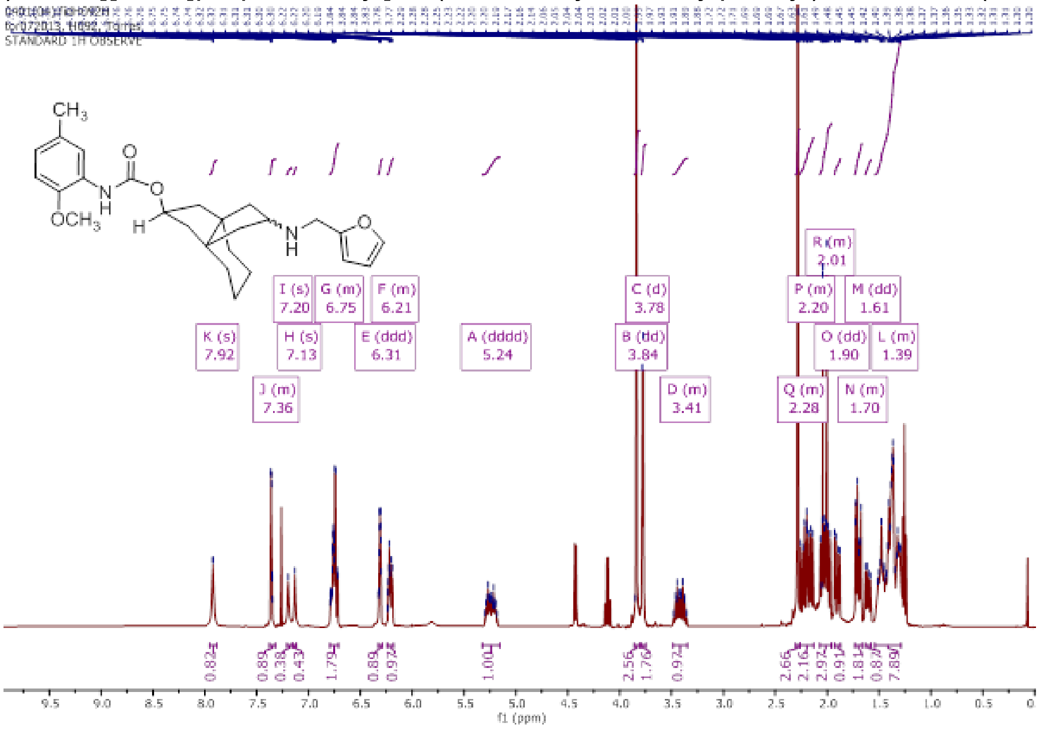

^{13}C NMR spectrum of [(8-*anti*-11-*anti* and 8-*anti*-11-*syn*)-11-[(Furan-2-yl-mehtyl)amino][4.3.3]propellan-8-yl] N-(2-methoxy-5-methylphenyl)carbamate (8-*anti*-**4i**)

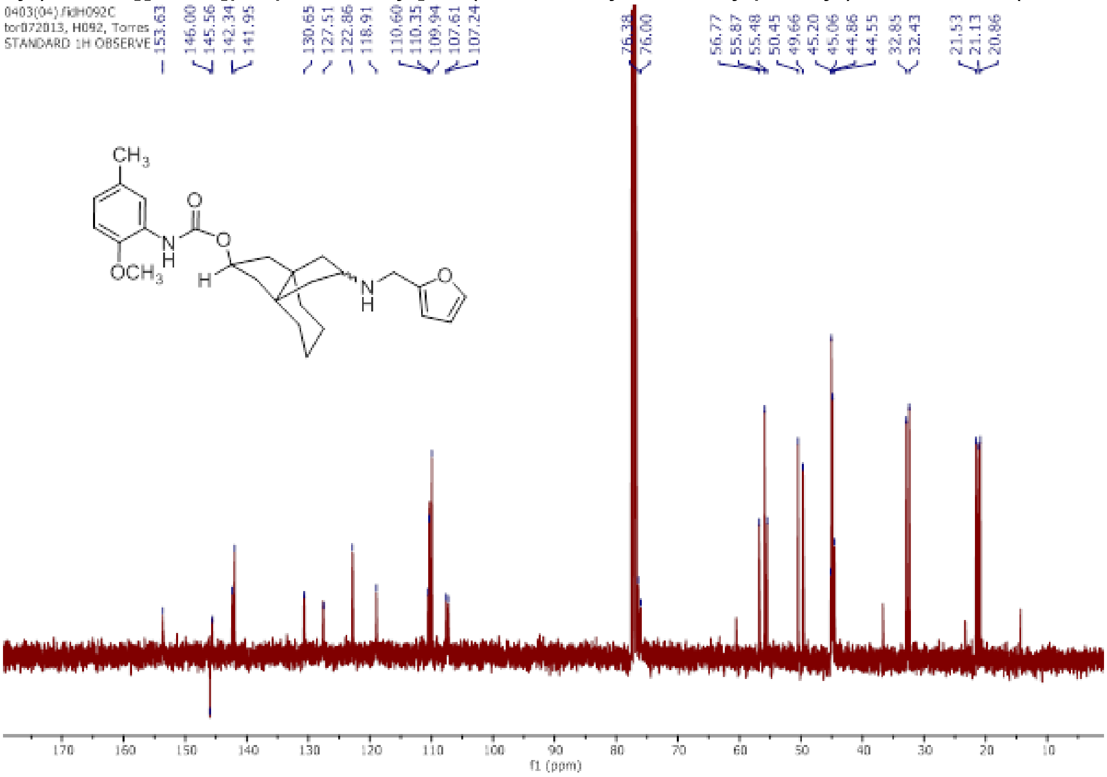

^{1}H NMR spectrum of 8-*anti*-**4i** showing the diastereoisomeric ratio based on the integrals for protons at 8- and 11-positions.

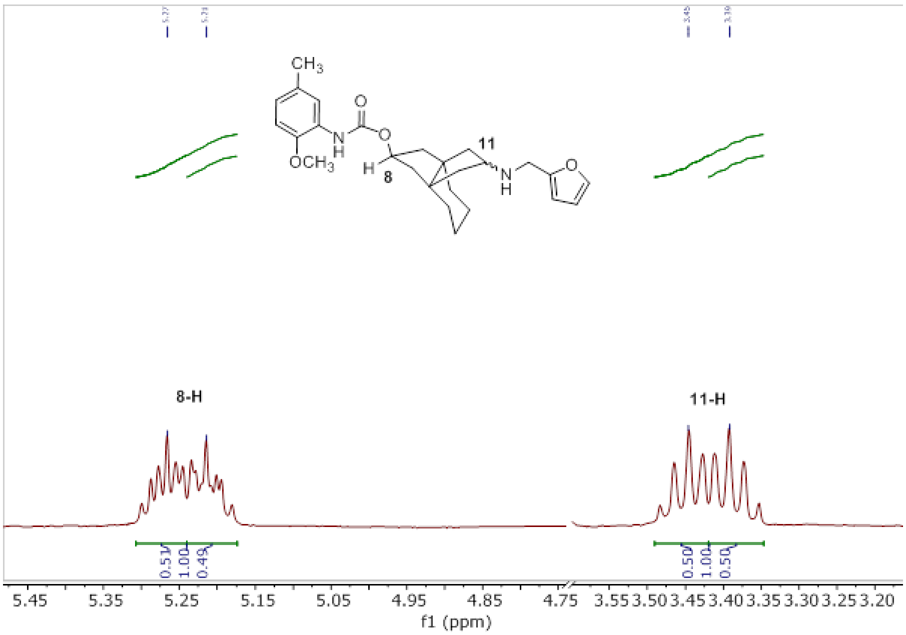

^{1}H NMR spectrum of [(8-*syn*-11-*syn* and 8-*syn*-11-*anti*)-11-[(Furan-2-yl-mehtyl)amino][4.3.3]propellan-8-yl] N-(2-methoxy-5-methylphenyl)carbamate (8-*syn*-**4i**)

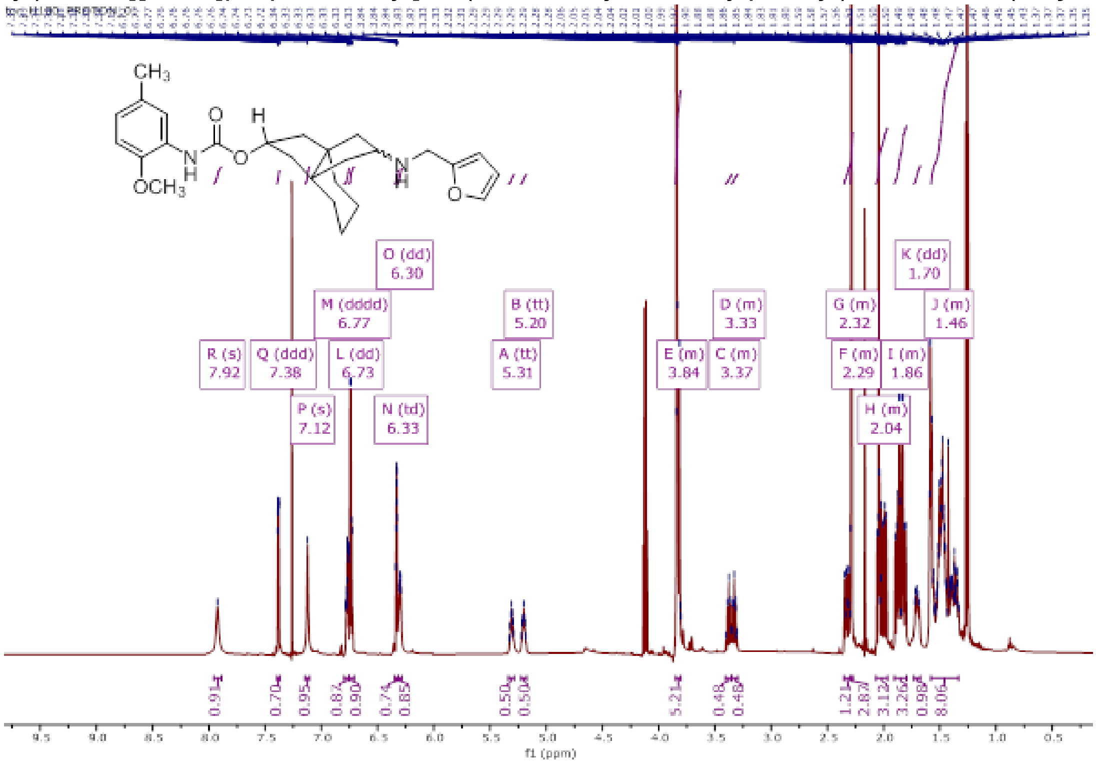

^{13}C NMR spectrum of [(8-*syn*-11-*syn* and 8-*syn*-11-*anti*)-11-[(Furan-2-yl-mehtyl)amino][4.3.3]propellan-8-yl] N-(2-methoxy-5-methylphenyl)carbamate (8-*syn*-4i)

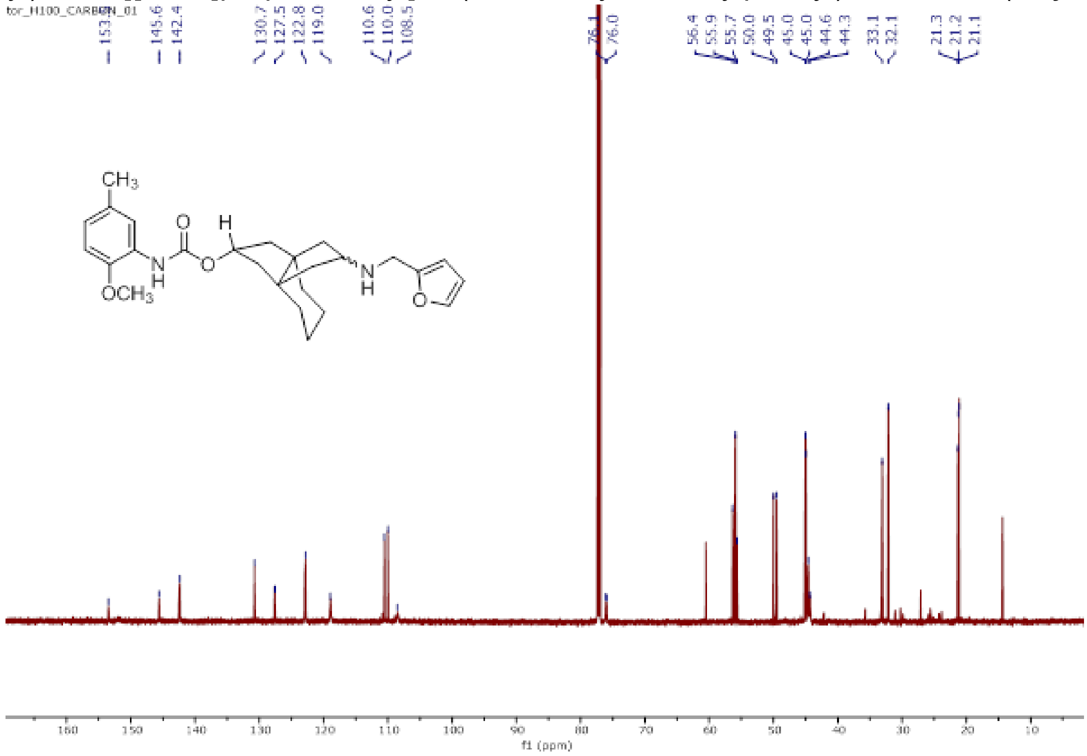

^{1}H NMR spectrum of 8-*syn*-**4i** showing the diastereoisomeric ratio based on the integrals for protons at 8- and 11-positions.

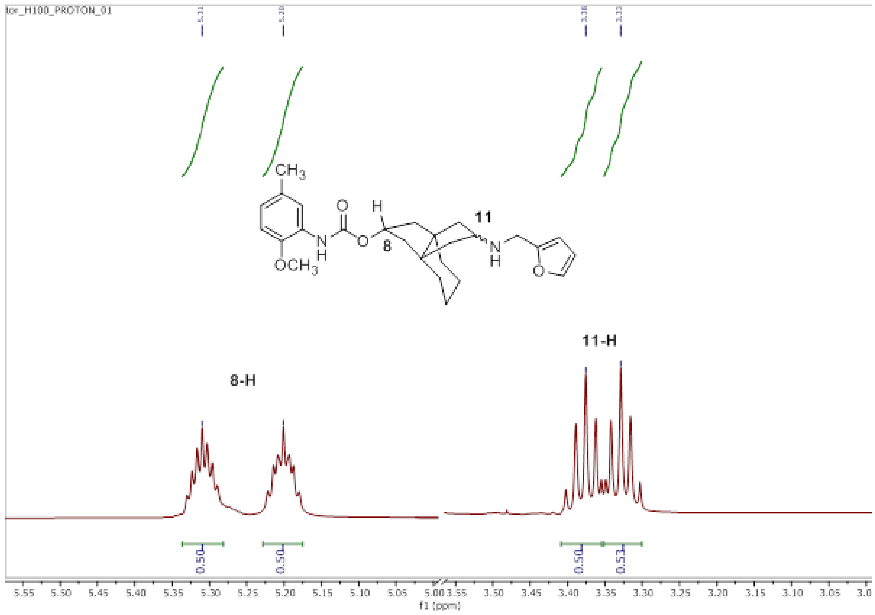

^{1}H NMR spectrum of [(8-*anti*-11-*anti* and 8-*anti*-11-*syn*)-11-[2-(Indol-3-yl)ethylamino][4.3.3]propellan-8-yl] N-(2-methoxy-5-methylphenyl)carbamate (8-*anti*-**4k**)

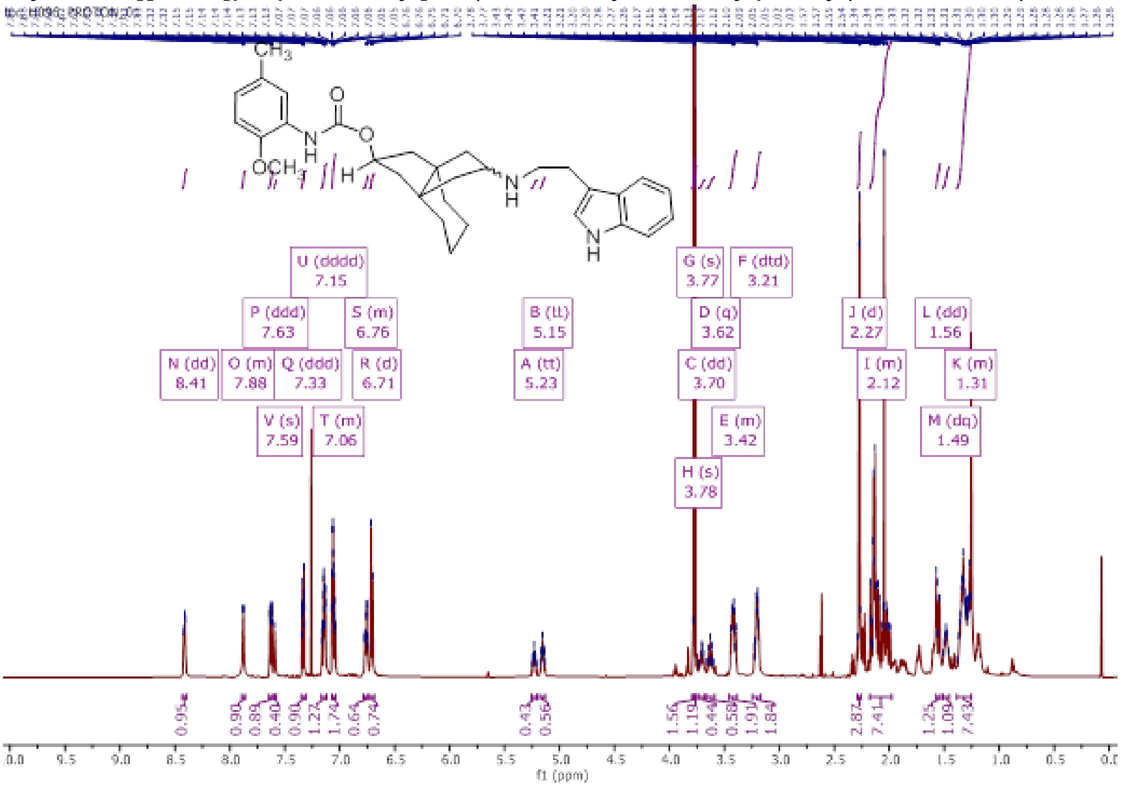

^{13}C NMR spectrum of [(8-*anti*-11-*anti* and 8-*anti*-11-*syn*)-11-[2-(Indol-3-yl)ethylamino][4.3.3]propellan-8-yl] N-(2-methoxy-5-methylphenyl)carbamate (8-*anti*-**4k**)

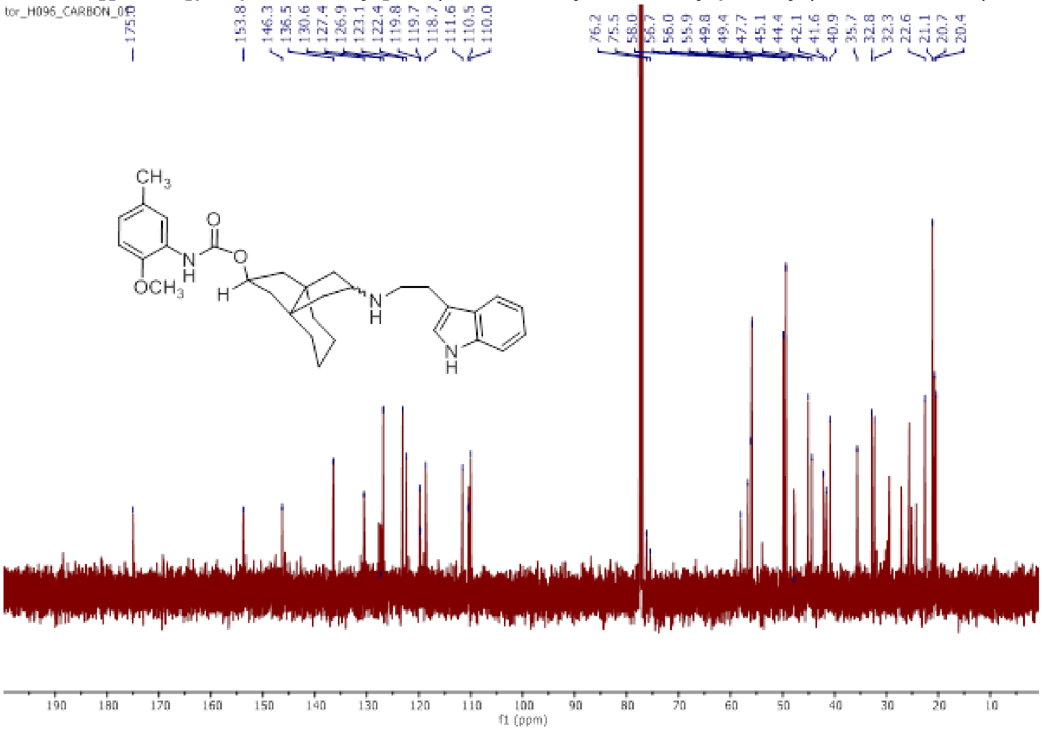

^{1}H NMR spectrum of 8-*anti*-**4k** showing the diastereoisomeric ratio based on the integrals for protons at 8- and 11-positions.

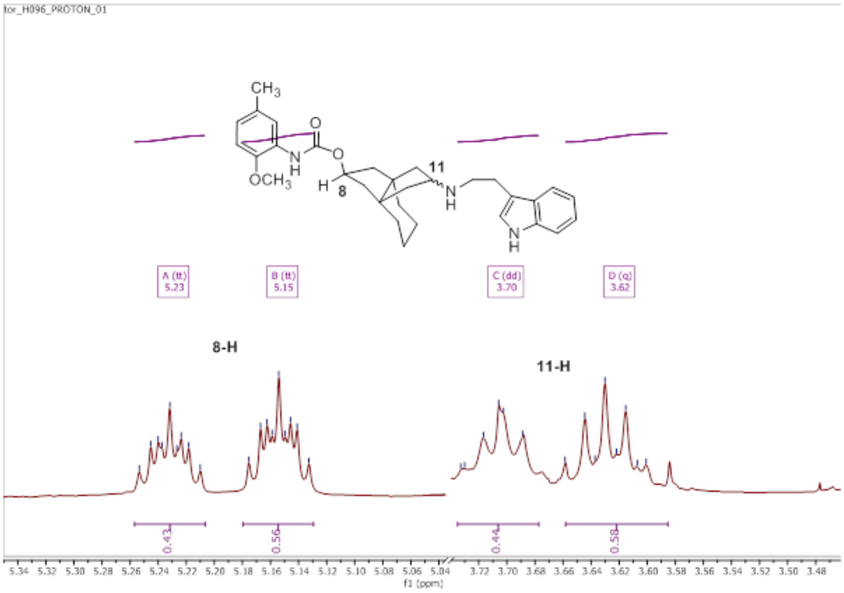

^{1}H NMR spectrum of [(8-*syn*-11-*syn* and 8-*syn*-11-*anti*)-11-[(2-(Indol-3-yl)ethylamino][4.3.3]propellan-8-yl] N-(2-methoxy-5-methylphenyl)carbamate (8-*syn*-**4k**)

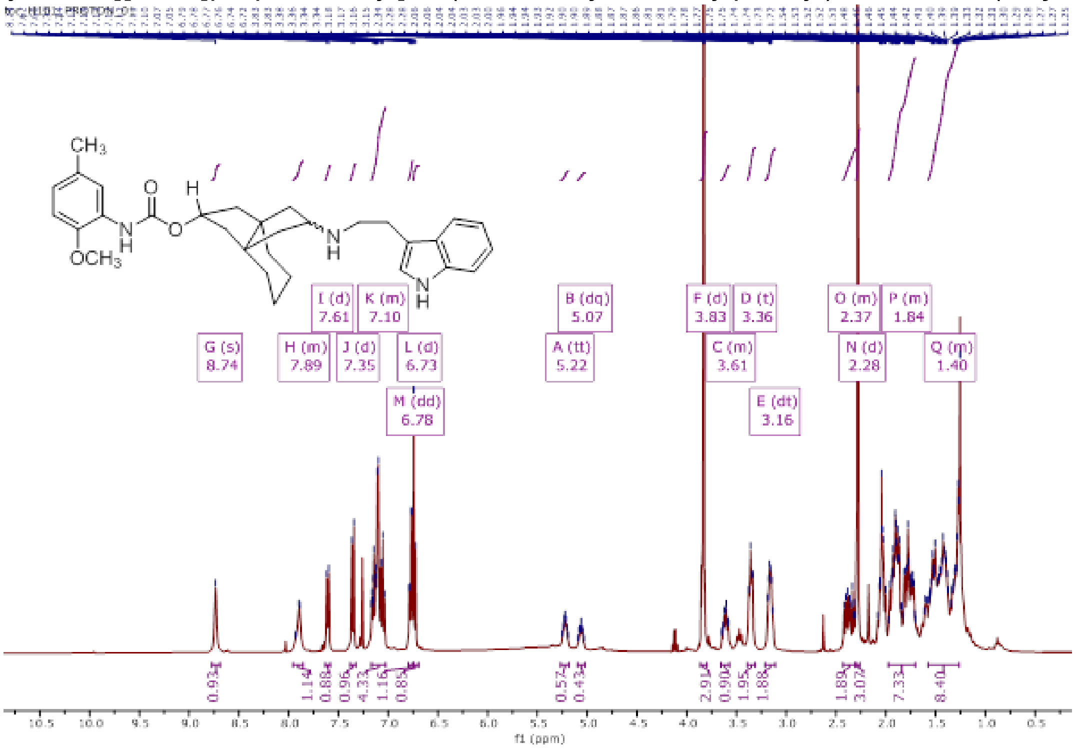

^{13}C NMR spectrum of [(8-*syn*-11-*syn* and 8-*syn*-11-*anti*)-11-[(2-(Indol-3-yl)ethylamino][4.3.3]propellan-8-yl] N-(2-methoxy-5-methylphenyl)carbamate (8-*syn*-**4k**)

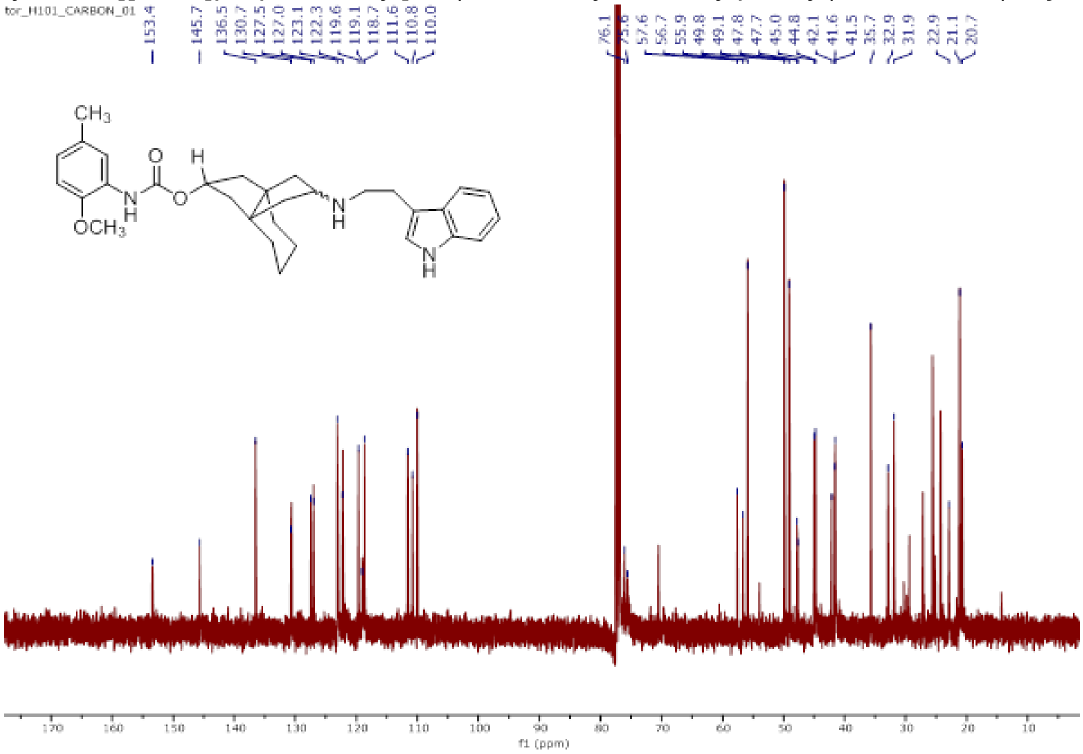

^{1}H NMR spectrum of 8-*syn*-**4k** showing the diastereoisomeric ratio based on the integrals for protons at 8- and 11-positions. In this case protons at 11-H position for both *anti* and *syn* isomers are overlapping.

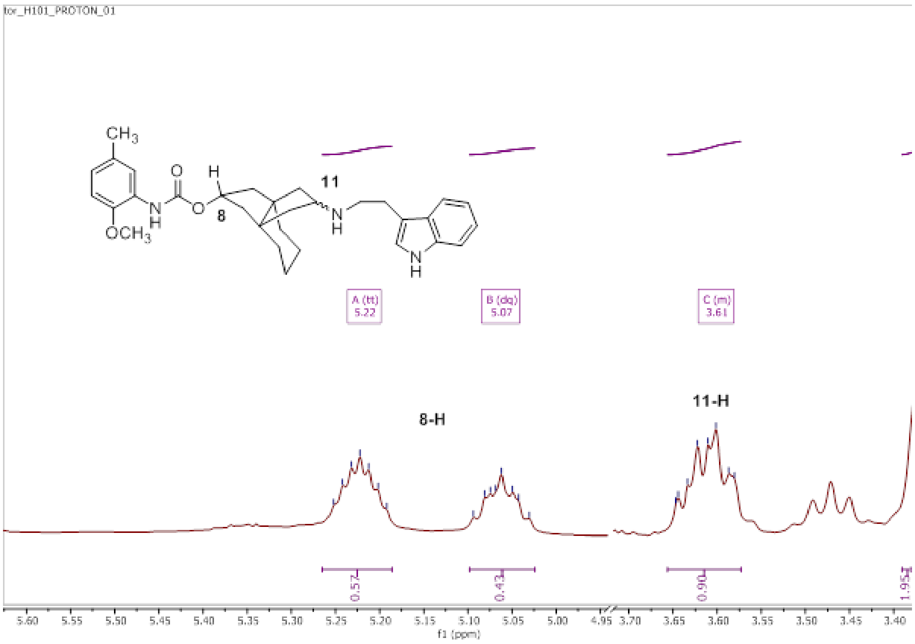

^{1}H NMR spectrum of [(8-*anti*-11-*anti* and 8-*anti*-11-*syn*)-11-(Phenylamino)[4.3.3]propellan-8-yl] N-(2-methoxy-5-methylphenyl)carbamate (8-*anti*-**4l**)

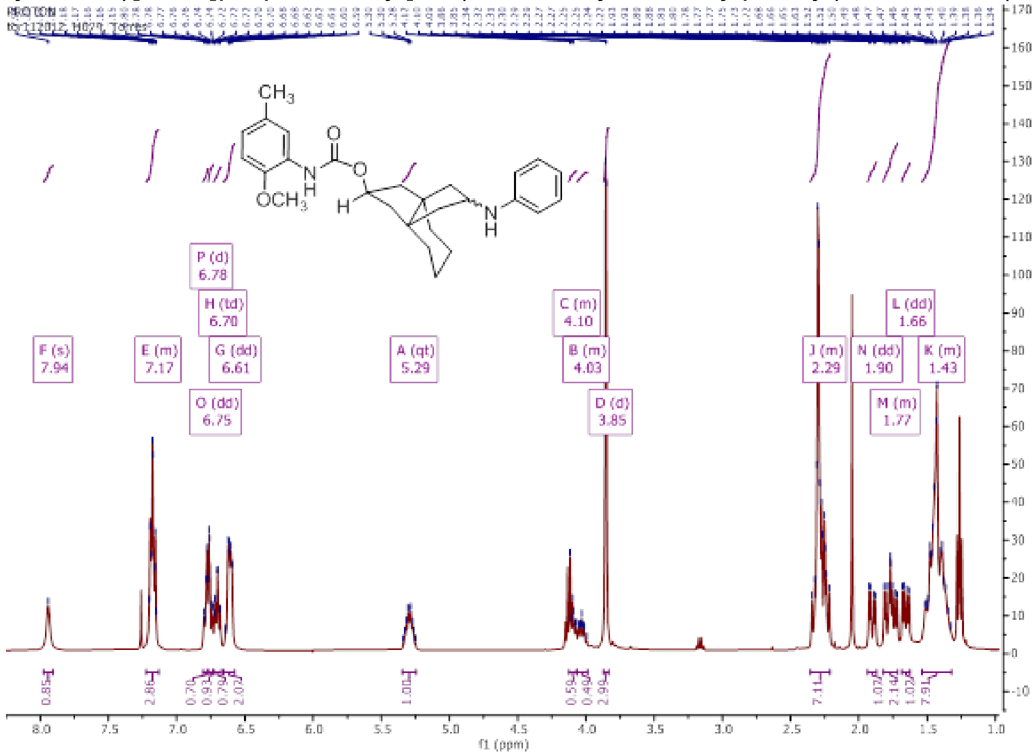

^{13}C NMR spectrum of [(8-*anti*-11-*anti* and 8-*anti*-11-*syn*)-11-(Phenylamino)[4.3.3]propellan-8-yl] N-(2-methoxy-5-methylphenyl)carbamate (8-*anti*-**4l**)

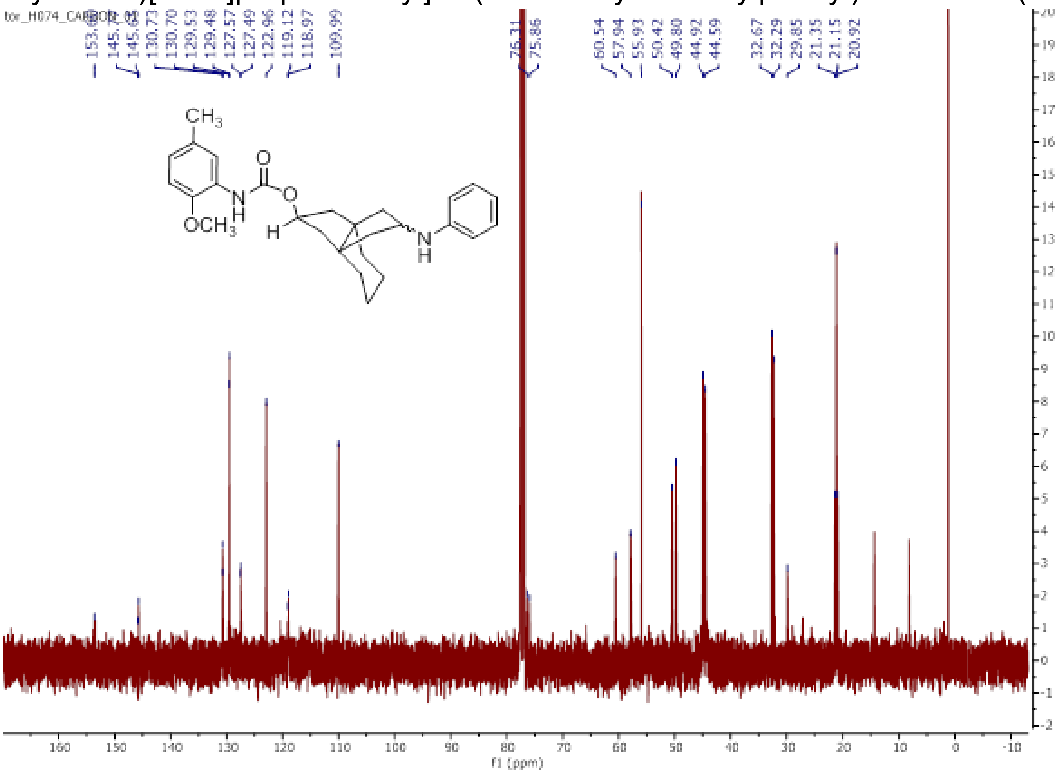

^{1}H NMR spectrum of [(8-*anti*-11-*anti* and 8-*anti*-11-*syn*)-11-(4-Methoxyphenylamino)[4.3.3]propellan-8-yl] N-(2-methoxy-5-methylphenyl)carbamate (8-*anti*-**4m**)

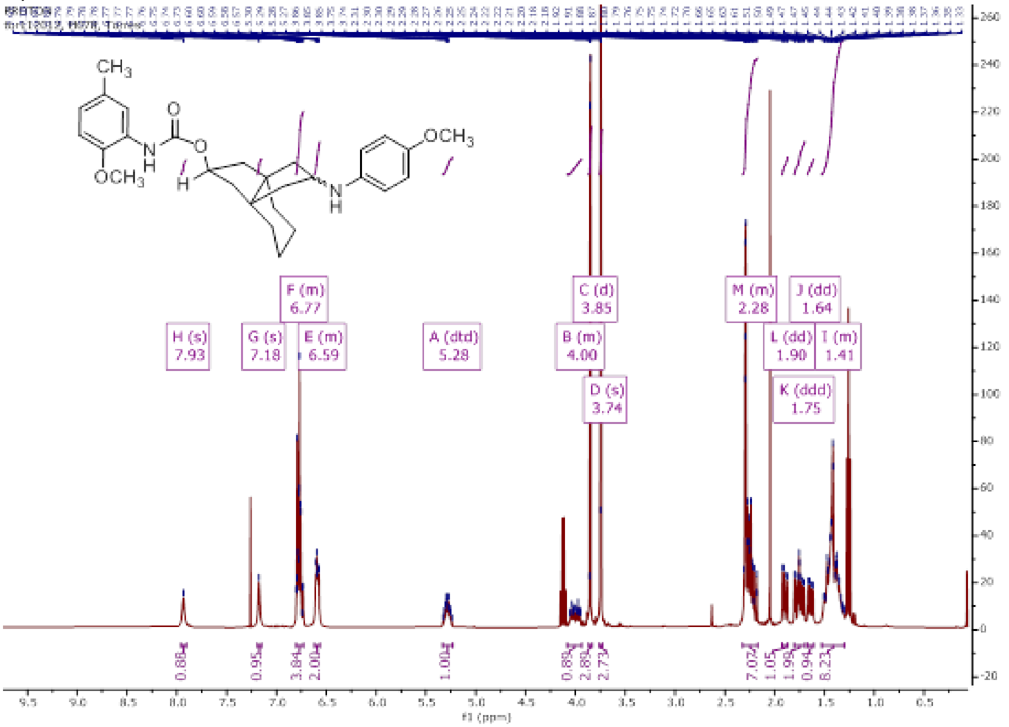

^{13}C NMR spectrum of [(8-*anti*-11-*anti* and 8-*anti*-11-*syn*)-11-(4-Methoxyphenylamino)[4.3.3]propellan-8-yl] N-(2-methoxy-5-methylphenyl)carbamate (8-*anti*-**4m**)

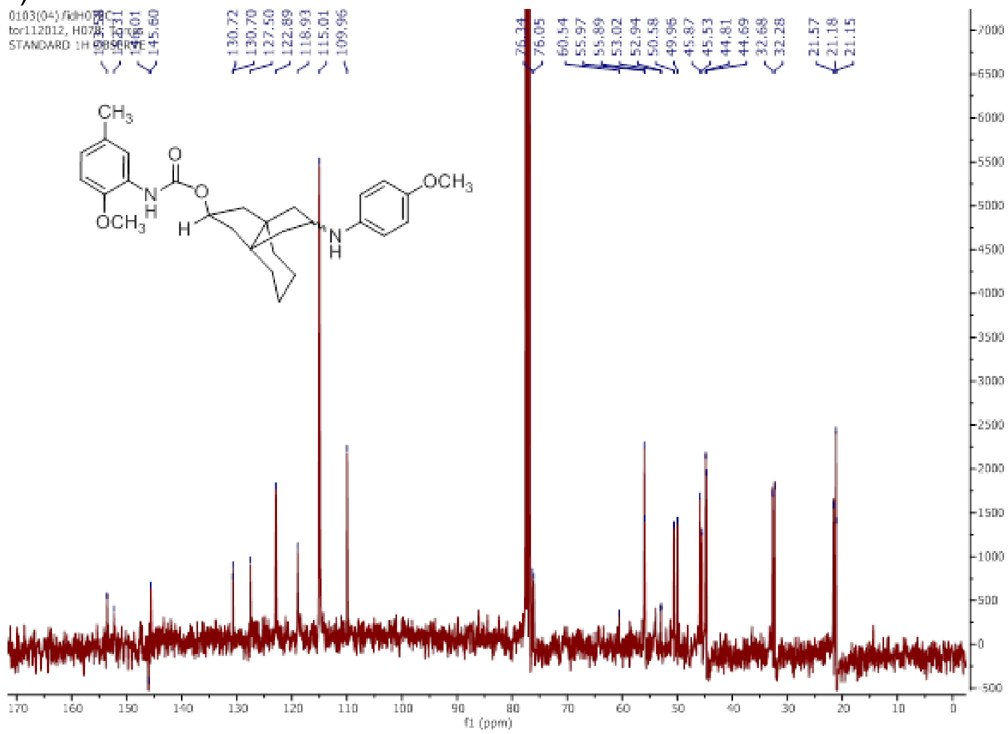

^{1}H NMR spectrum of [(8-*anti*-11-*anti* and 8-*anti*-11-*syn*)-11-(3-Chloro-4-methoxyphenylamino)[4.3.3]propellan-8-yl] N-(2-methoxy-5-methylphenyl)carbamate (8-*anti*-**4n**)

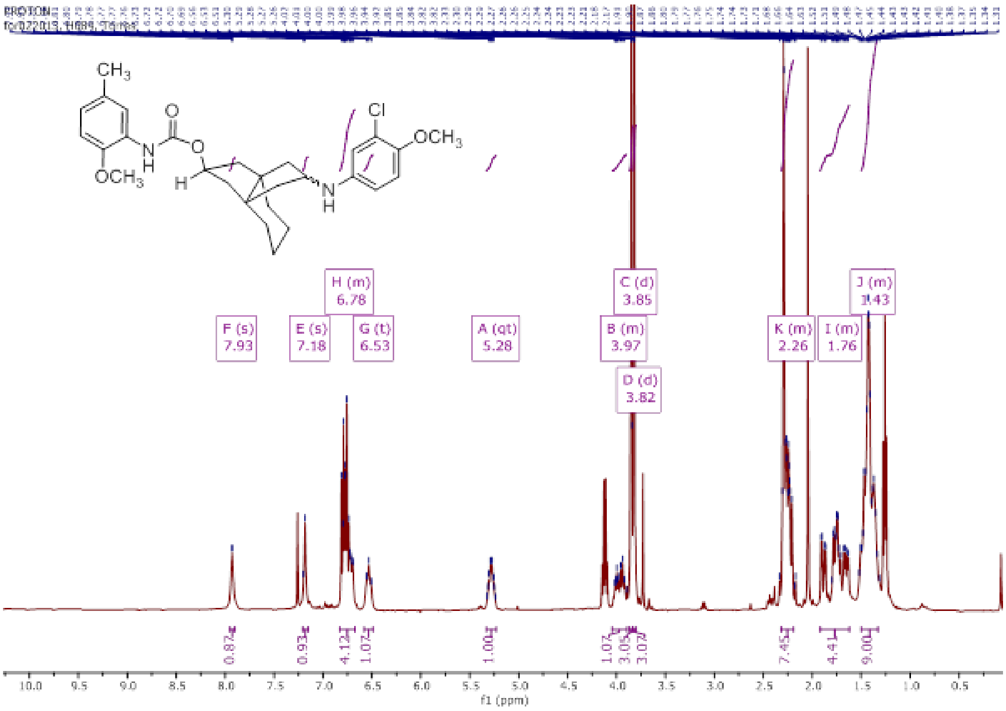

^{13}C NMR spectrum of [(8-*anti*-11-*anti* and 8-*anti*-11-*syn*)-11-(3-Chloro-4-methoxyphenylamino)[4.3.3]propellan-8-yl] N-(2-methoxy-5-methylphenyl)carbamate (8-*anti*-**4n**)

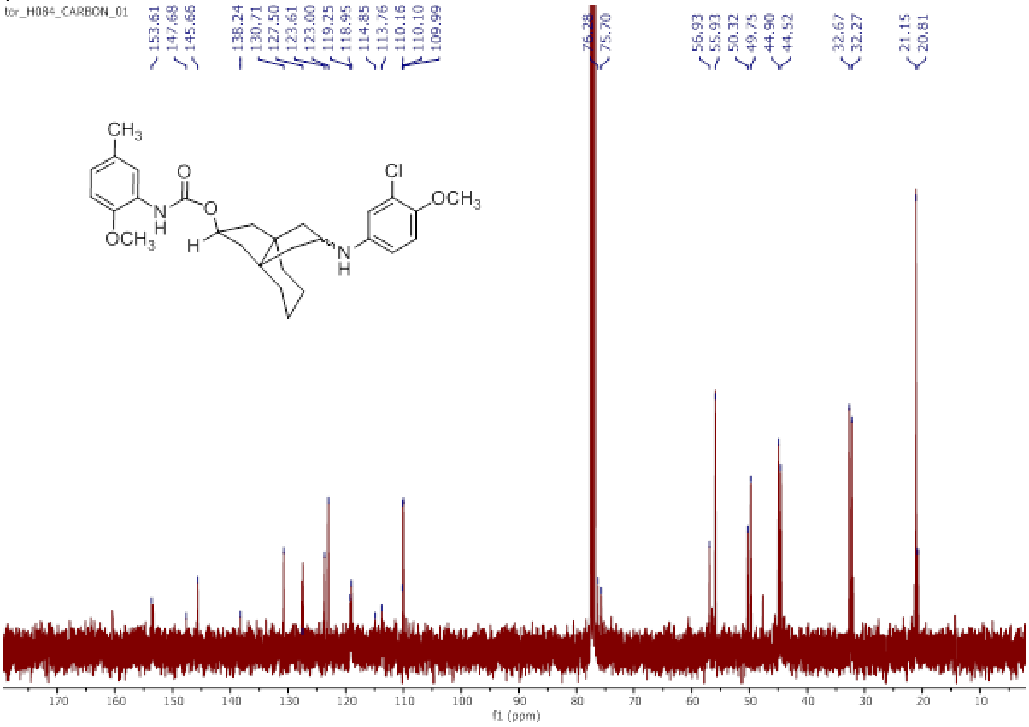

HSQC NMR spectrum of [(8-*anti*-11-*anti* and 8-*anti*-11-*syn*)-11-(3-Chloro-4-methoxyphenylamino)[4.3.3]propellan-8-yl] N-(2-methoxy-5-methylphenyl)carbamate (8-*anti*-**4n**)

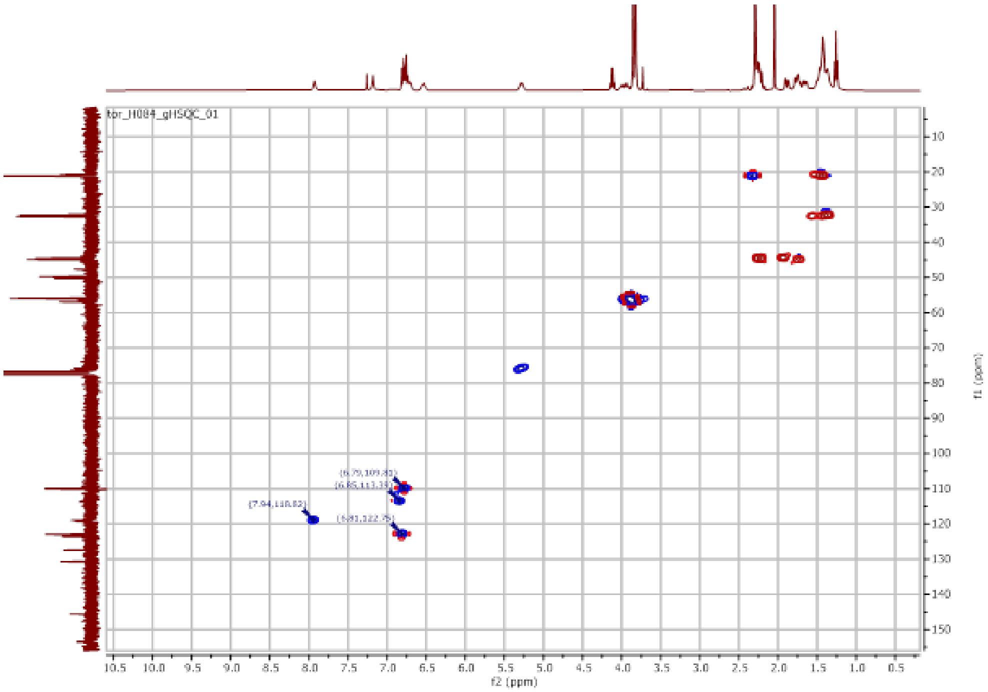

^{1}H NMR spectrum of [(8-*anti*-11-*anti* and 8-*anti*-11-*syn*)-11-(4-Aminophenylamino)[4.3.3]propellan-8-yl] N-(2-methoxy-5-methylphenyl)carbamate (8-*anti*-**4o**)

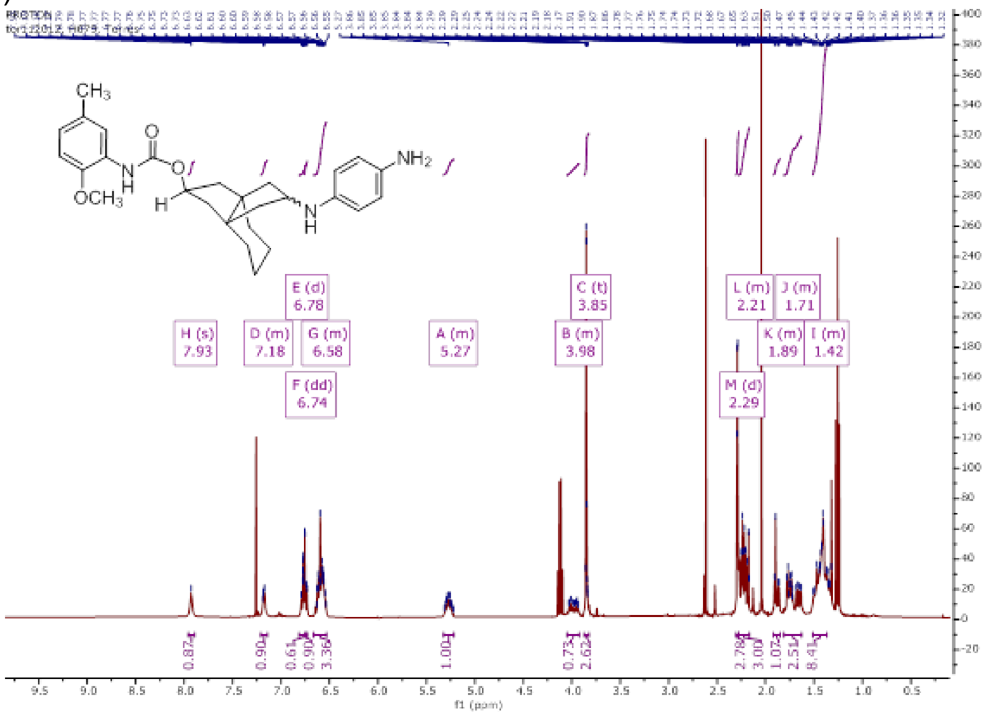

^{13}C NMR spectrum of [(8-*anti*-11-*anti* and 8-*anti*-11-*syn*)-11-(4-Aminophenylamino)[4.3.3]propellan-8-yl] N-(2-methoxy-5-methylphenyl)carbamate (8-*anti*-**4o**)

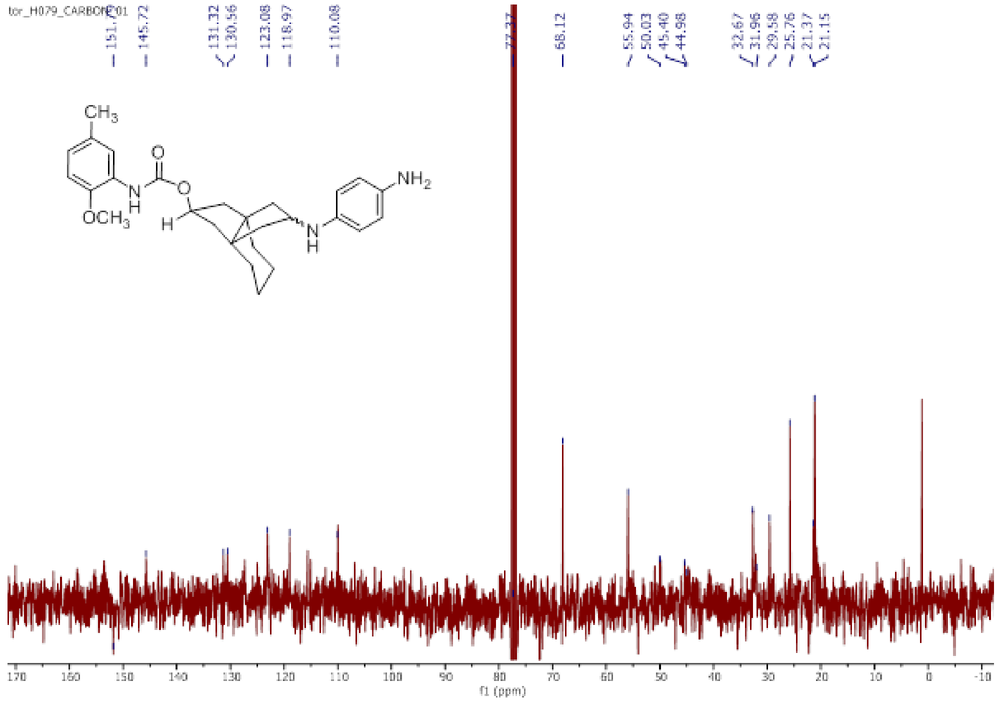

HSQC NMR spectrum of [(8-*anti*-11-*anti* and 8-*anti*-11-*syn*)-11-(4-Aminophenylamino)[4.3.3]propellan-8-yl] N-(2-methoxy-5-methylphenyl)carbamate (8-*anti*-**4o**)

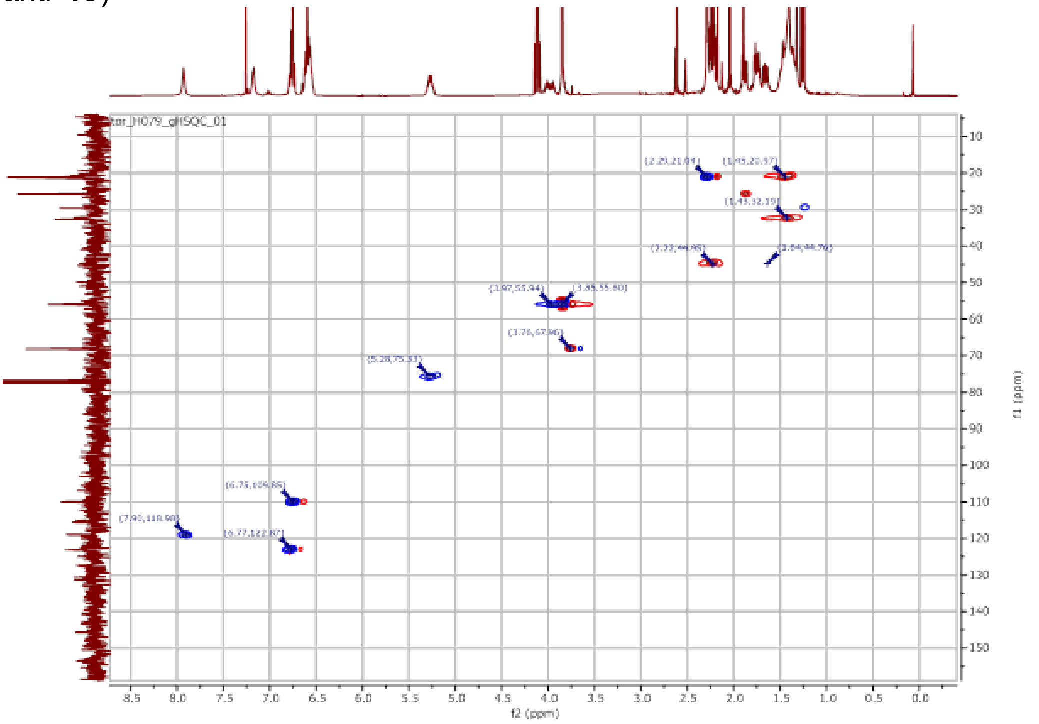

^{1}H NMR spectrum of [(8-*anti*-11-*anti* and 8-*anti*-11-*syn*)-11-(Phenethylamino)[4.3.3]propellan-8-yl] N-(2-methoxy-5-methylphenyl)carbamate (8-*anti*-**4p**)

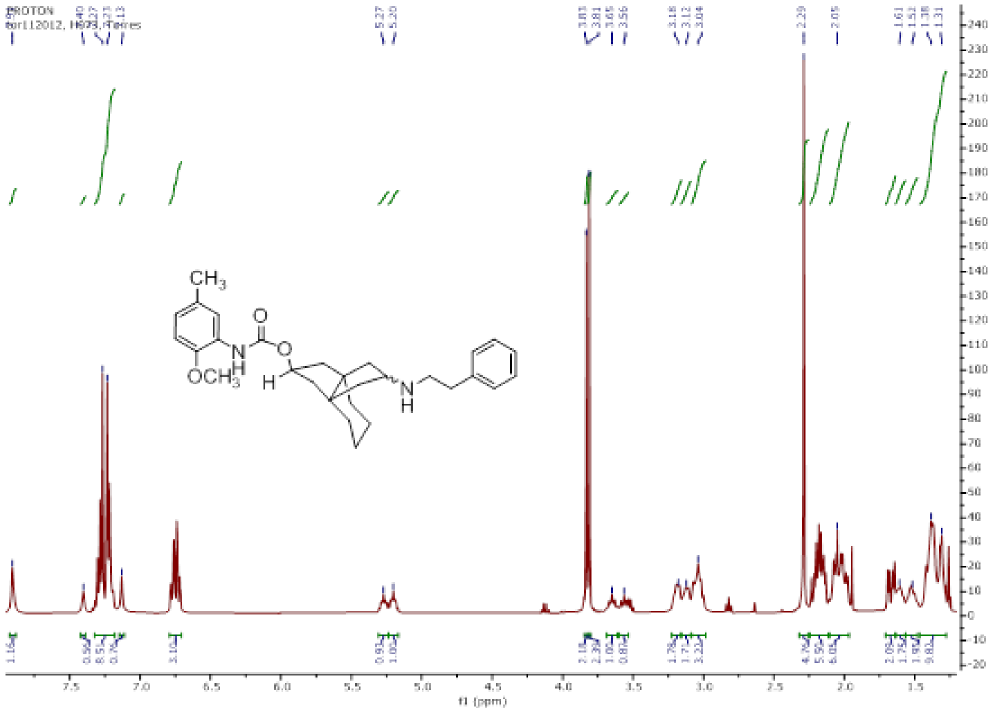

^{13}C NMR spectrum of [(8-*anti*-11-*anti* and 8-*anti*-11-*syn*)-11-(Phenethylamino)[4.3.3]propellan-8-yl] N-(2-methoxy-5-methylphenyl)carbamate (8-*anti*-**4p**)

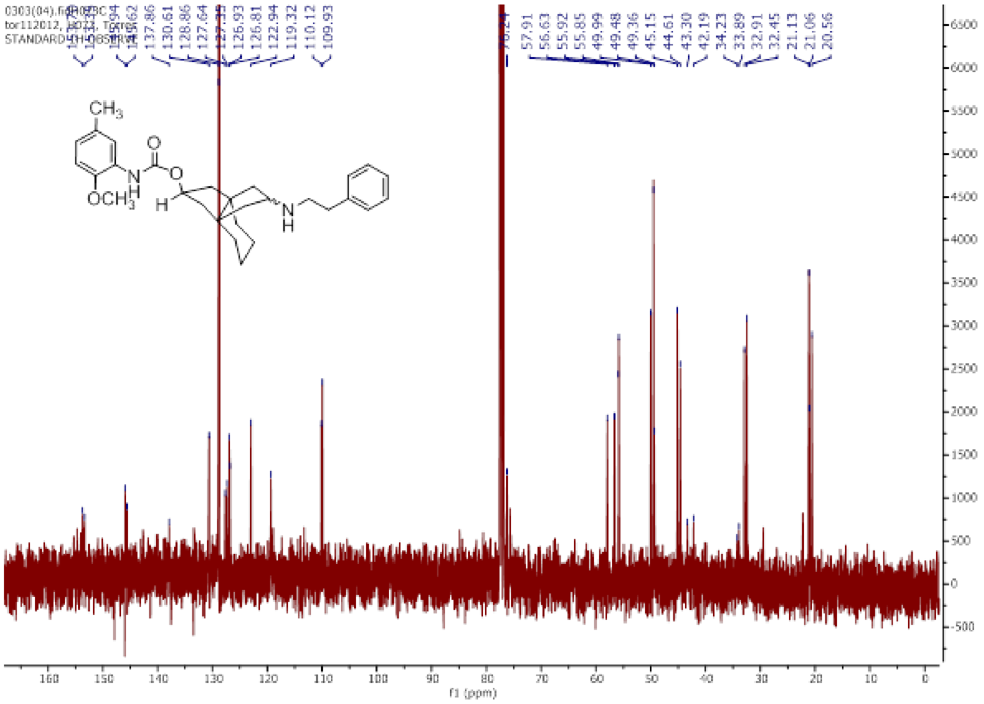

HSQC NMR spectrum of [(8-*anti*-11-*anti* and 8-*anti*-11-*syn*)-11-(Phenethylamino)[4.3.3]propellan-8-yl] N-(2-methoxy-5-methylphenyl)carbamate (8-*anti*-**4p**)

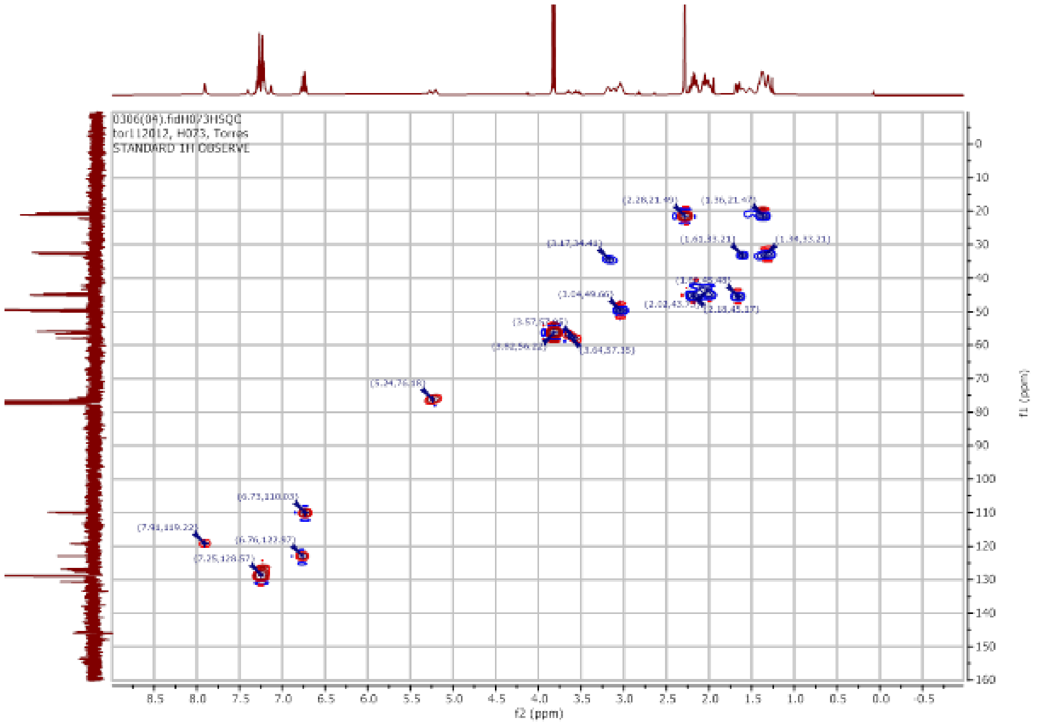

^{1}H NMR spectrum of [(8-*anti*-11-*anti* and 8-*anti*-11-*syn*)-11-(3-Phenylpropylamino)[4.3.3]propellan-8-yl] N-(2-methoxy-5-methylphenyl)carbamate (8-*anti*-**4q**)

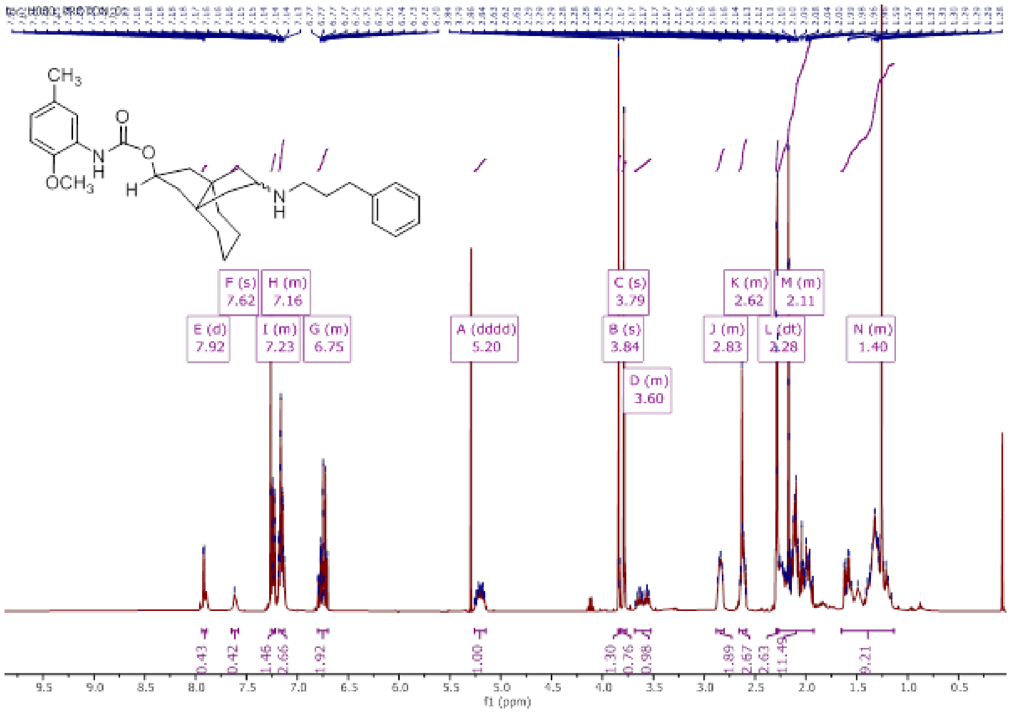

^{13}C NMR spectrum of [(8-*anti*-11-*anti* and 8-*anti*-11-*syn*)-11-(3-Phenylpropylamino)[4.3.3]propellan-8-yl] N-(2-methoxy-5-methylphenyl)carbamate (8-*anti*-**4q**)

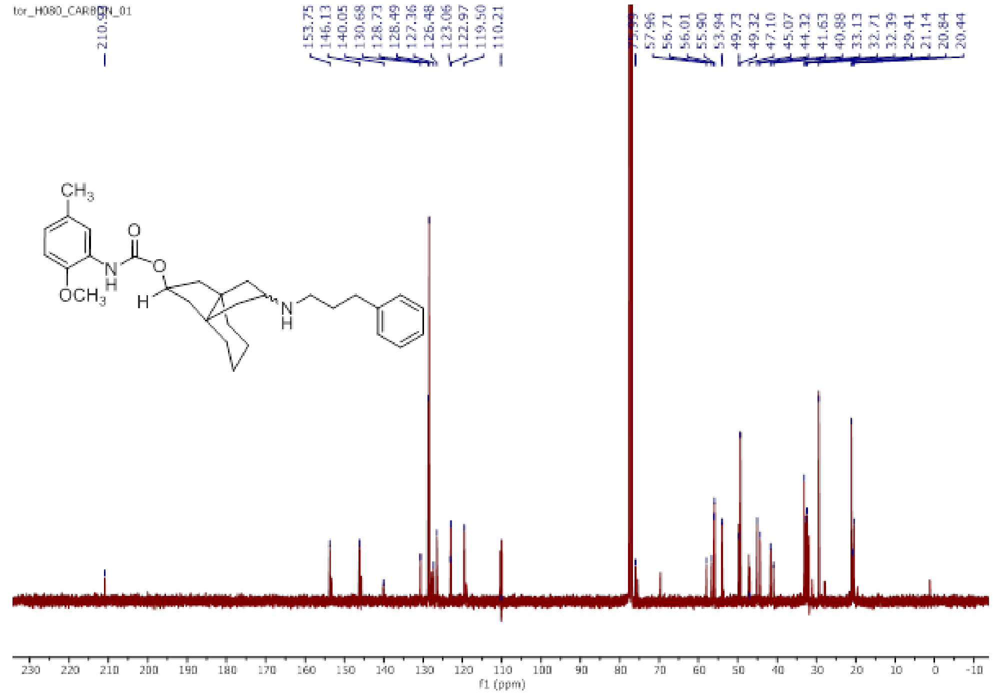

^{1}H NMR spectrum of [(8-*anti*-11-*anti* and 8-*anti*-11-*syn*)-11-[(3-Aminopropyl)amino][4.3.3]propellan-8-yl] N-(2-methoxy-5-methylphenyl)carbamate (8-*anti*-**4r**)

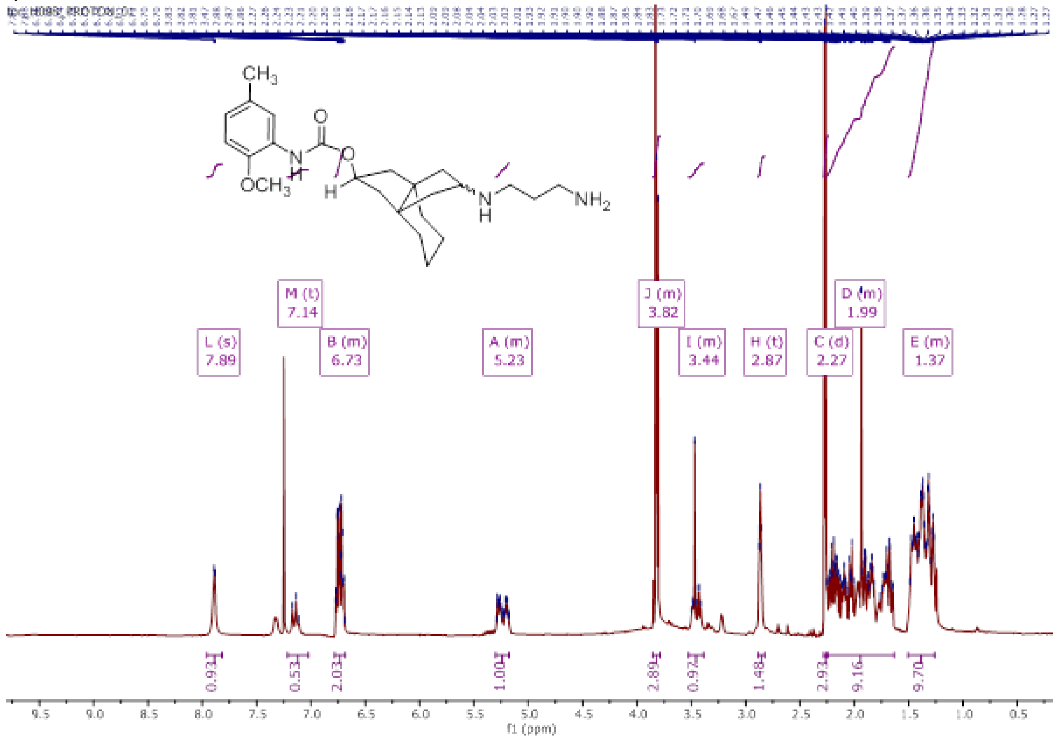

^{13}C NMR spectrum of [(8-*anti*-11-*anti* and 8-*anti*-11-*syn*)-11-[(3-Aminopropyl)amino][4.3.3]propellan-8-yl] N-(2-methoxy-5-methylphenyl)carbamate (8-*anti*-**4r**)

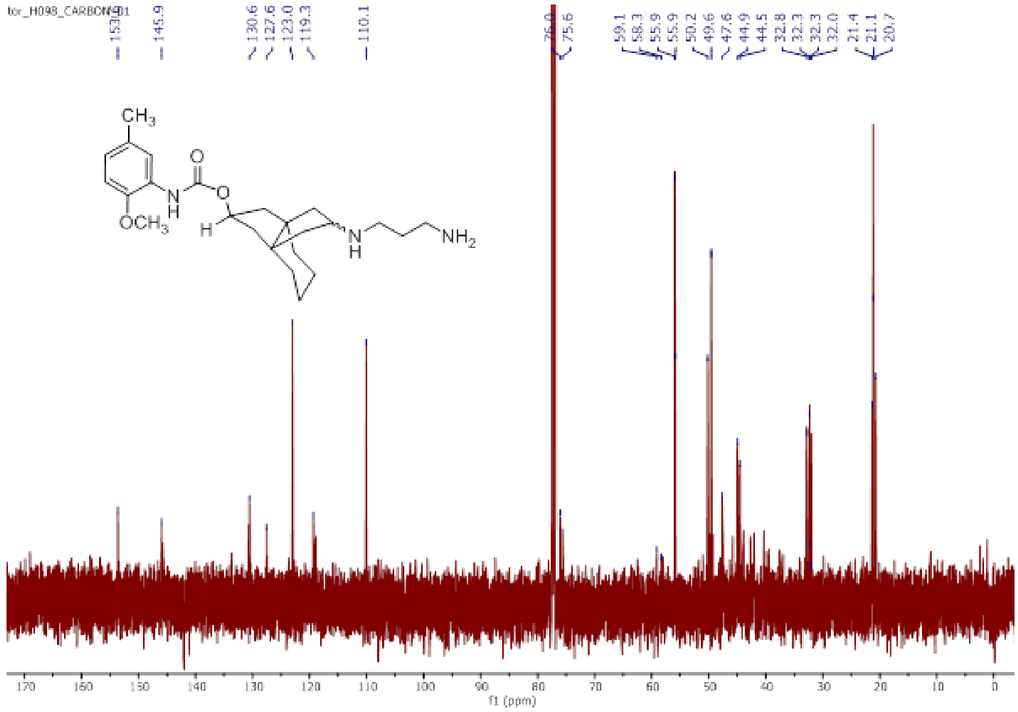

HSQC NMR spectrum of [(8-*anti*-11-*anti* and 8-*anti*-11-*syn*)-11-[(3-Aminopropyl)amino][4.3.3]propellan-8-yl] N-(2-methoxy-5-methylphenyl)carbamate (8-*anti*-**4r**)

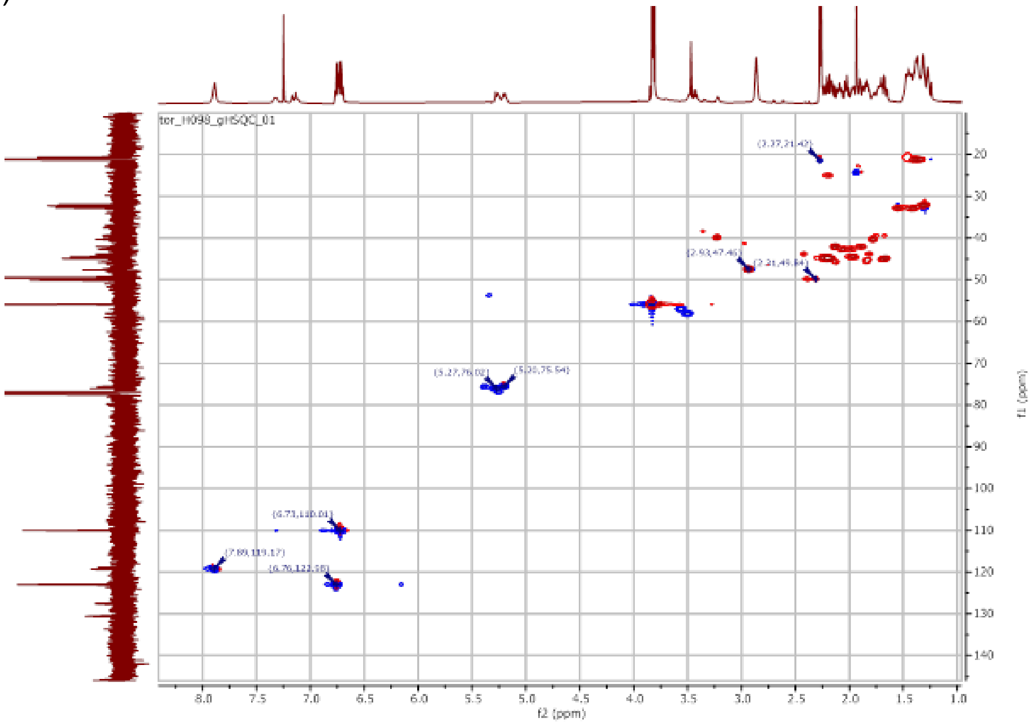

^{1}H NMR spectrum of [(8-*anti*-11-*anti* and 8-*anti*-11-*syn*)-11-(2-Hydroxyethyl-1-amino)[4.3.3]propellan-8-yl] N-(2-methoxy-5-methylphenyl)carbamate (8-*anti*-**4s**)

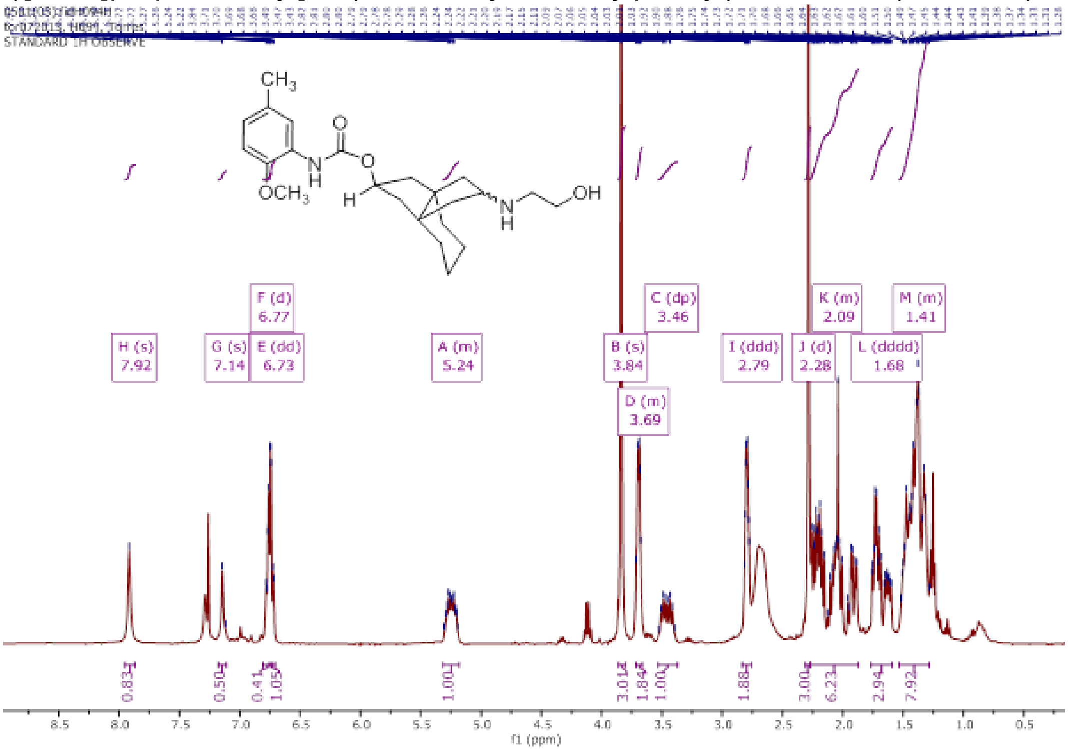

^{13}C NMR spectrum of [(8-*anti*-11-*anti* and 8-*anti*-11-*syn*)-11-(2-Hydroxyethyl-1-amino)[4.3.3]propellan-8-yl] N-(2-methoxy-5-methylphenyl)carbamate (8-*anti*-**4s**)

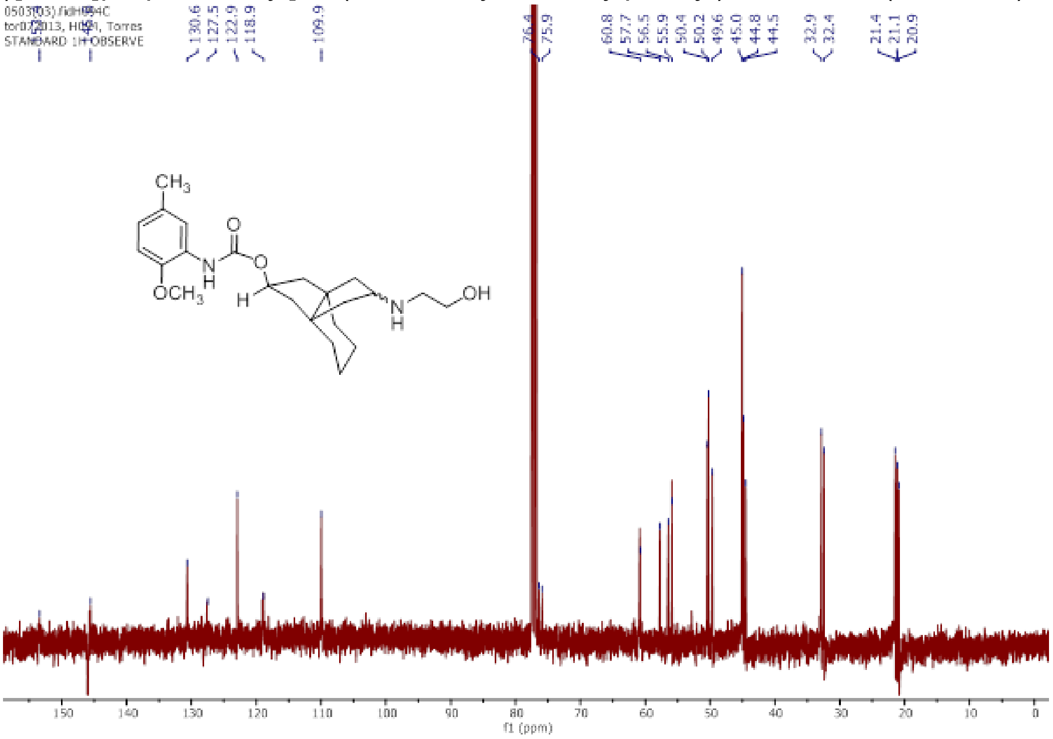

^{1}H NMR spectrum of [(8-*anti*-11-*anti* and 8-*anti*-11-*syn*)-11-(5-Hydroxypentylamino)[4.3.3]propellan-8-yl] N-(2-methoxy-5-methylphenyl)carbamate (8-*anti*-**4t**)

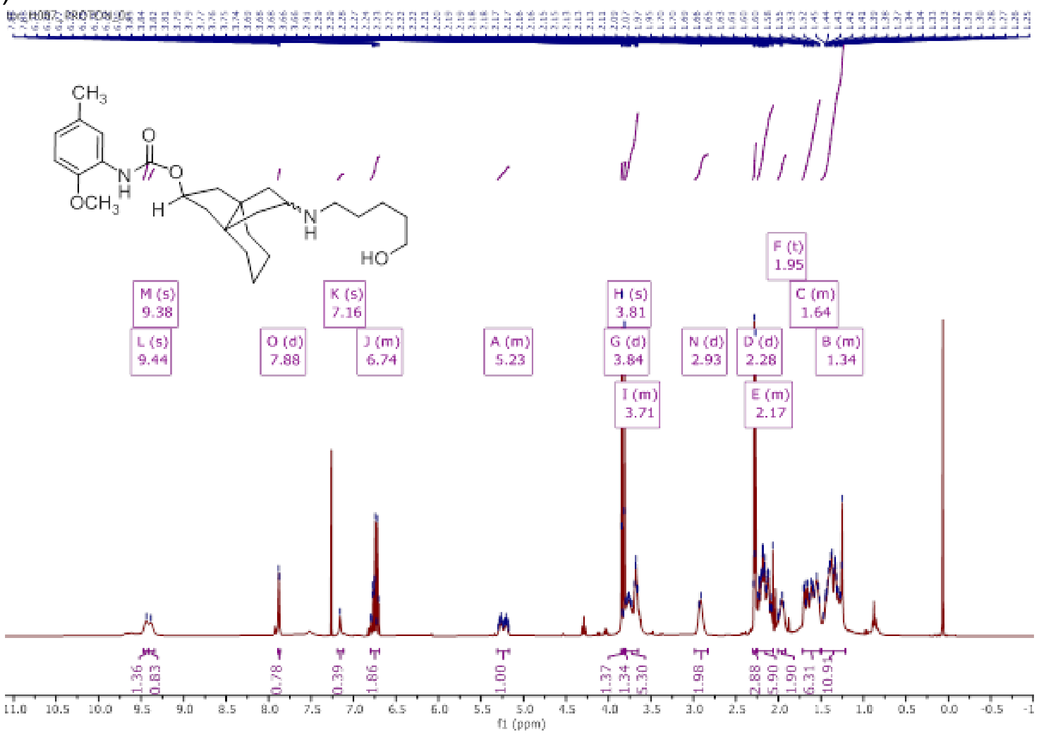

^{13}C NMR spectrum of [(8-*anti*-11-*anti* and 8-*anti*-11-*syn*)-11-(5-Hydroxypentylamino)[4.3.3]propellan-8-yl] N-(2-methoxy-5-methylphenyl)carbamate (8-*anti*-**4t**)

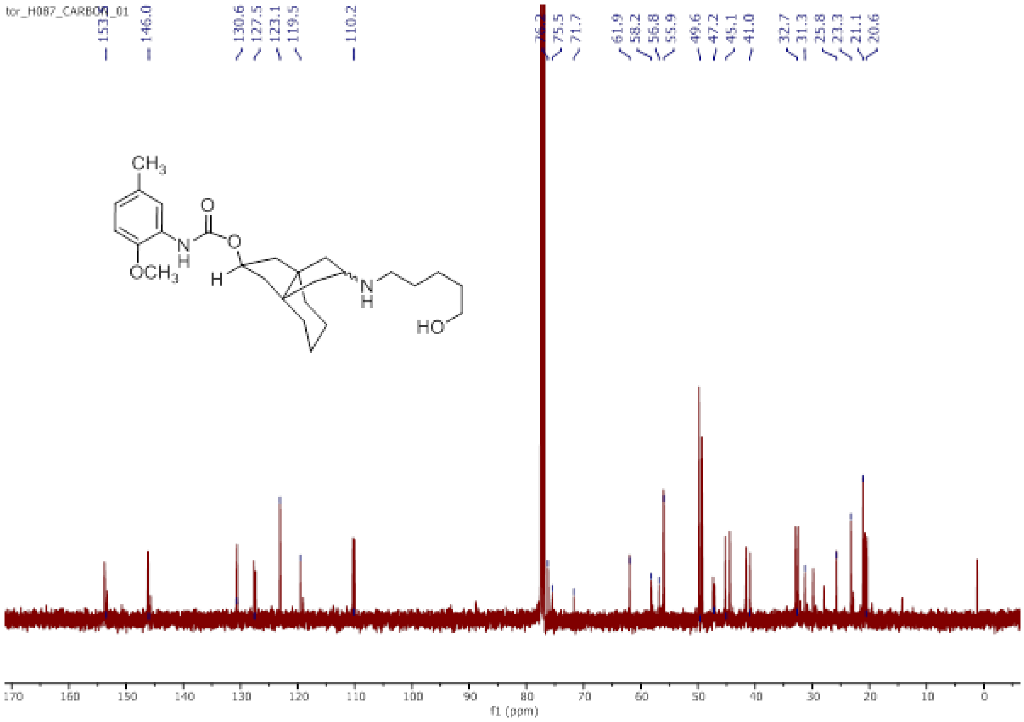

^{1}H NMR spectrum of [(8-*anti*-11-*anti* and 8-*anti*-11-*syn*)-11-(Isobutylamino)[4.3.3]propellan-8-yl] N-(2-methoxy-5-methylphenyl)carbamate (8-*anti*-**4u**)

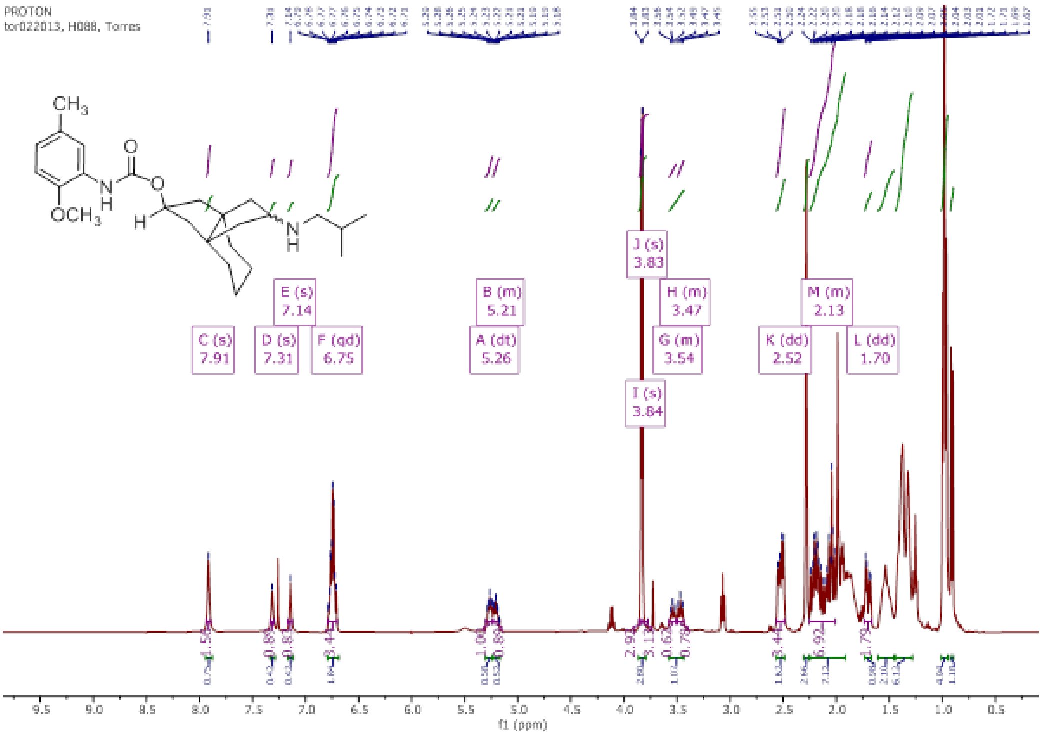

^{13}C NMR spectrum of[(8-*anti*-11-*anti* and 8-*anti*-11-*syn*)-11-(Isobutylamino)[4.3.3]propellan-8-yl] N-(2-methoxy-5-methylphenyl)carbamate (8-*anti*-**4u**)

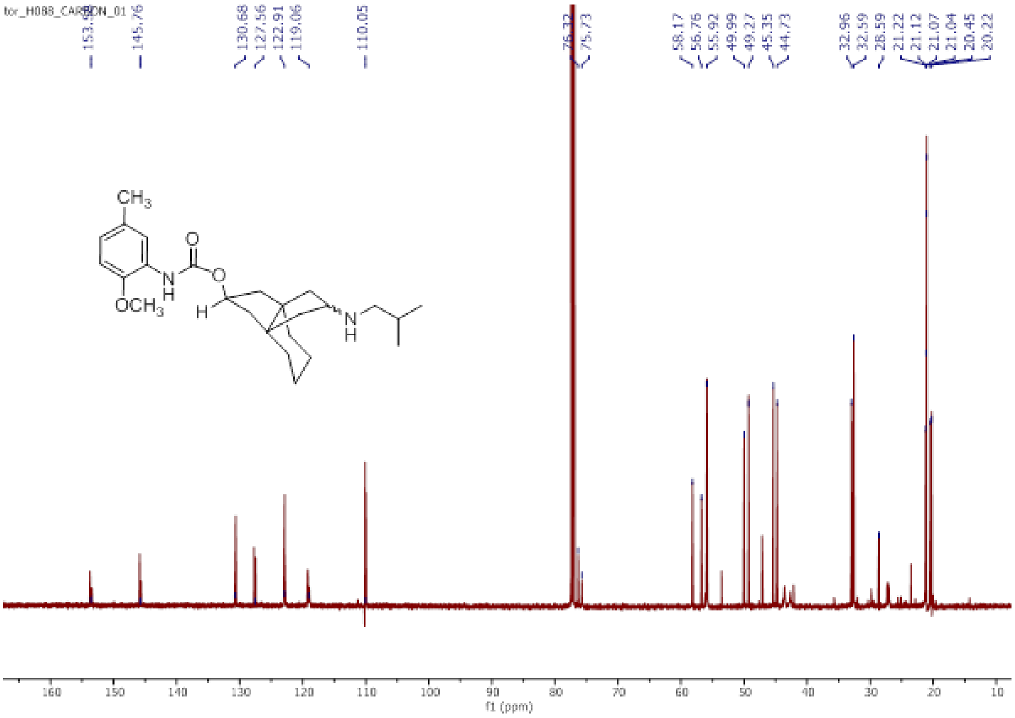

^{1}H NMR spectrum of [(8-*anti*-11-*anti* and 8-*anti*-11-*syn*)-11-(amino)[4.3.3]propellan-8-yl] N-(2-methoxy-5-methylphenyl)carbamate (8-*anti*-**4v**)

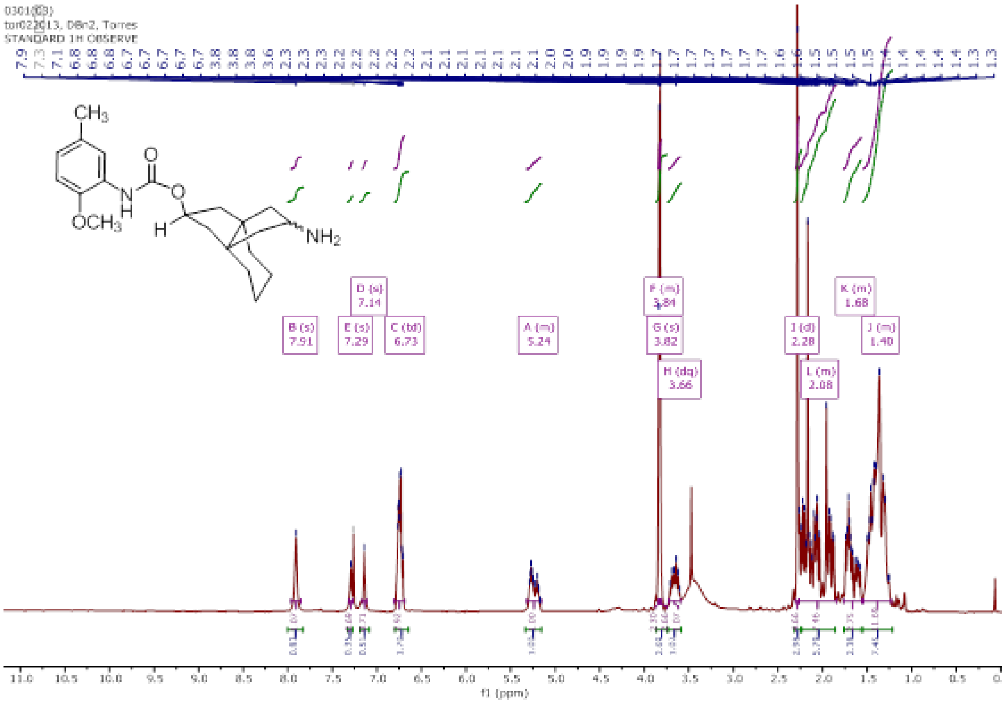

^{13}C NMR spectrum of [(8-*anti*-11-*anti* and 8-*anti*-11-*syn*)-11-(amino)[4.3.3]propellan-8-yl] N-(2-methoxy-5-methylphenyl)carbamate (8-*anti*-**4v**)

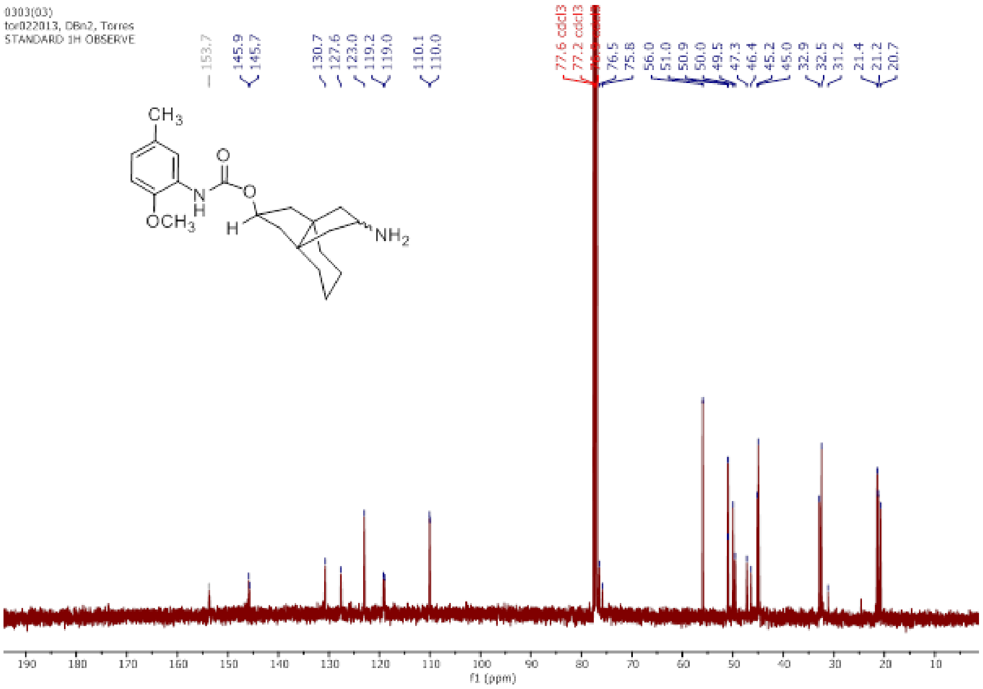

^{1}H NMR spectrum of [(8-*anti*-11-*anti* and 8-*anti*-11-*syn*)-11-(Benzylamino)[4.3.3]propellan-8-ol (11-*anti*-**13**)

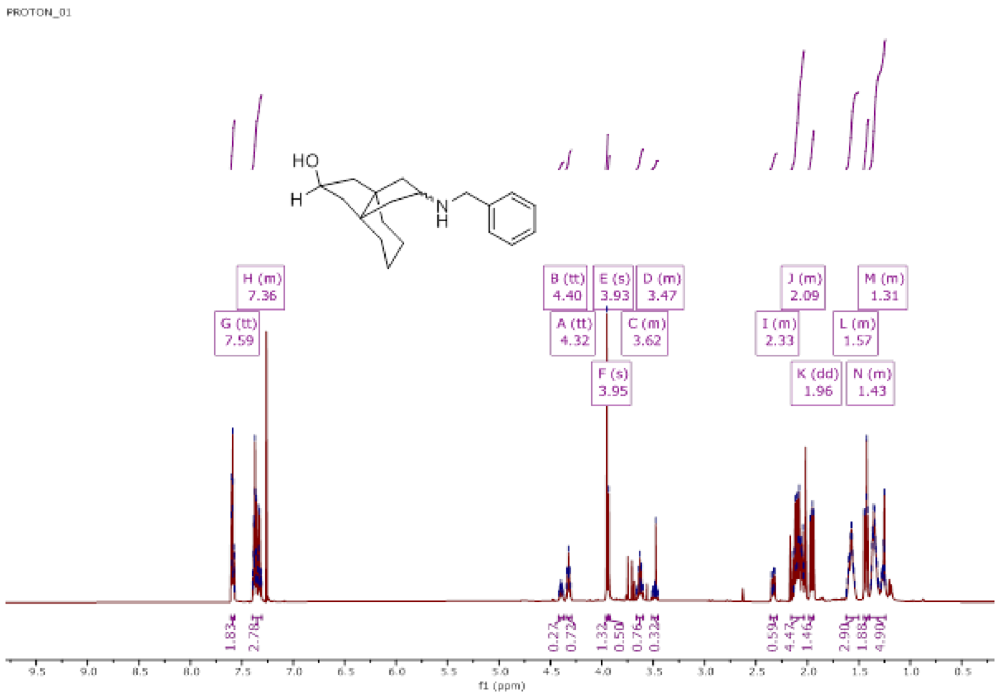

^{13}C NMR spectrum of [(8-*anti*-11-*anti* and 8-*anti*-11-*syn*)-11-(Benzylamino)[4.3.3]propellan-8-ol (11-*anti*-**13**)

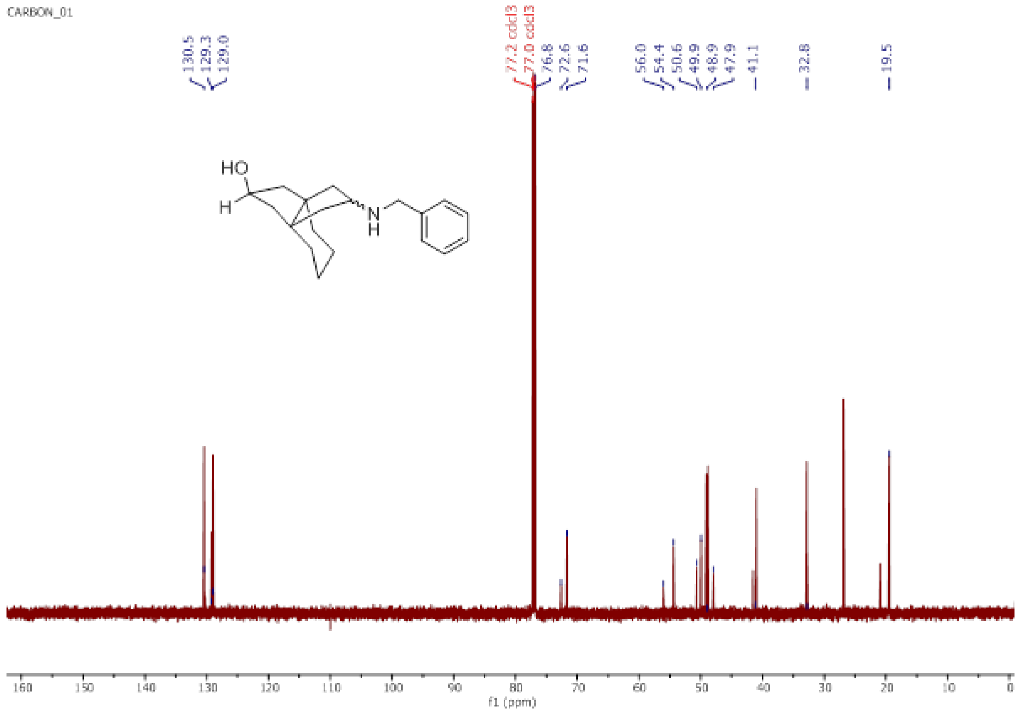

^{1}H NMR spectrum of [(8-*syn*-11-*syn* and 8-*syn*-11-*anti*)-11-(Benzylamino)[4.3.3]propellan-8-ol (11-*syn*-**13**)

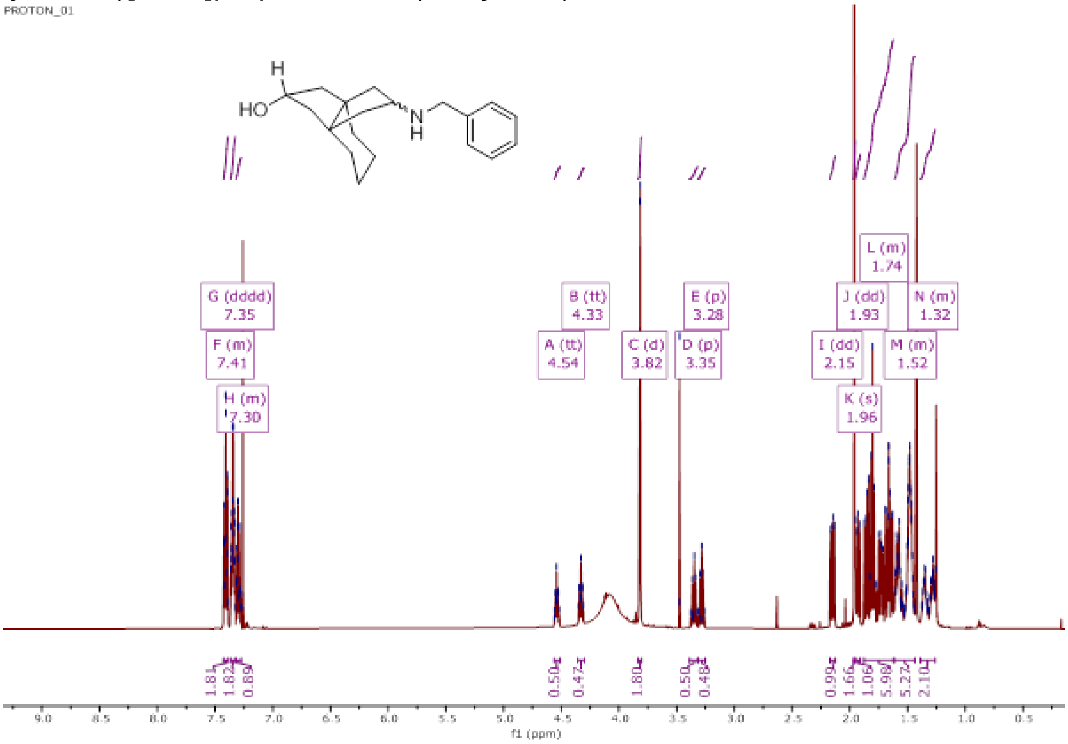

^{13}C NMR spectrum of [(8-*syn*-11-*syn* and 8-*syn*-11-*anti*)-11-(Benzylamino)[4.3.3]propellan-8-ol (11-*syn*-**13**)

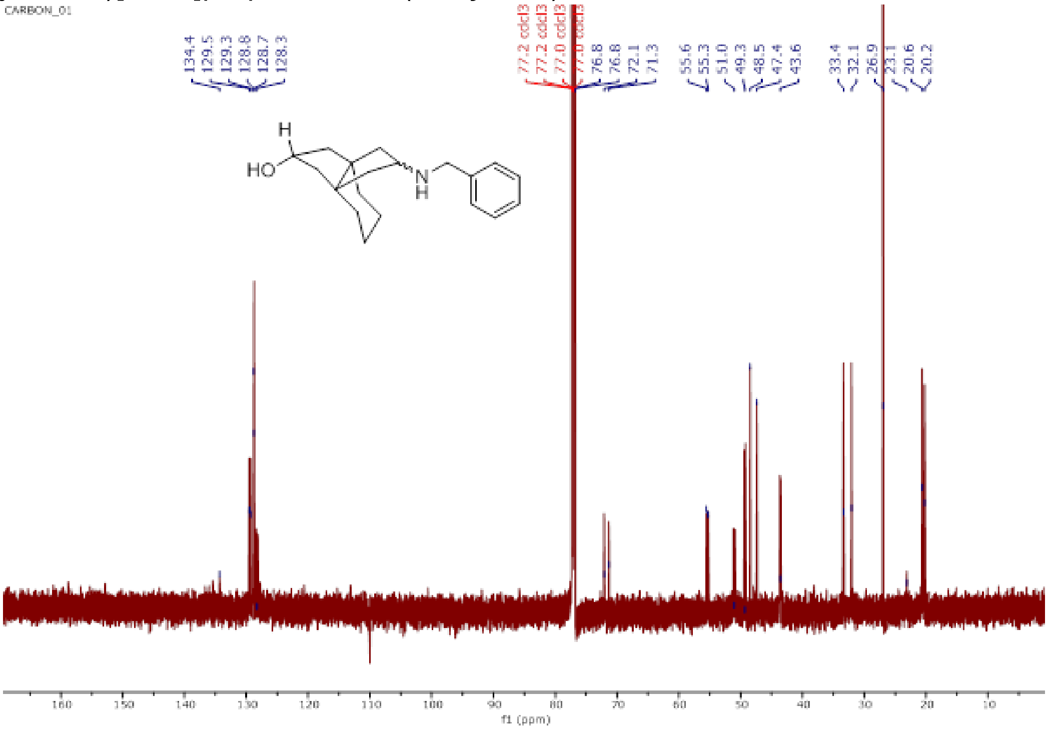

^{1}H NMR spectrum of (11'-*syn*)-Spiro-([1,3]-dioxolane-2,8'-(N-benzyl-[4.3.3]propellan))-11'-amine (*syn*-**14**)

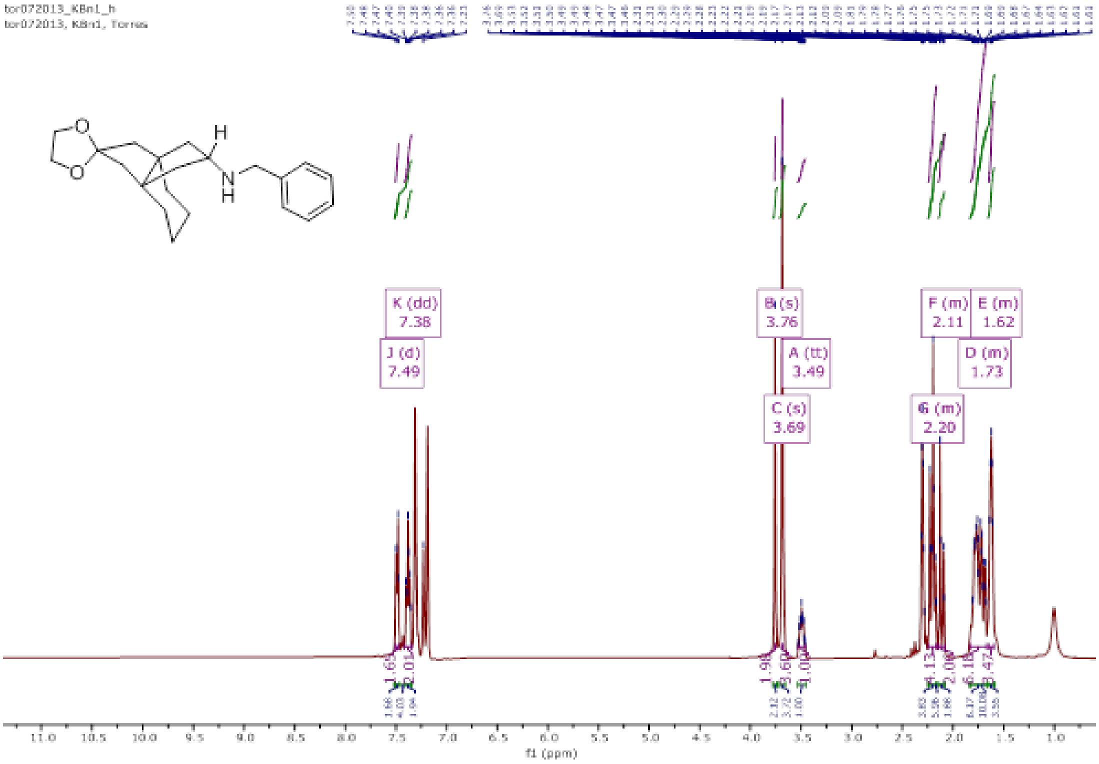

^{13}C NMR spectrum of (11'-*syn*)-Spiro-([1,3]-dioxolane-2,8'-(N-benzyl-[4.3.3]propellan))-11'-amine (*syn*-**14**)

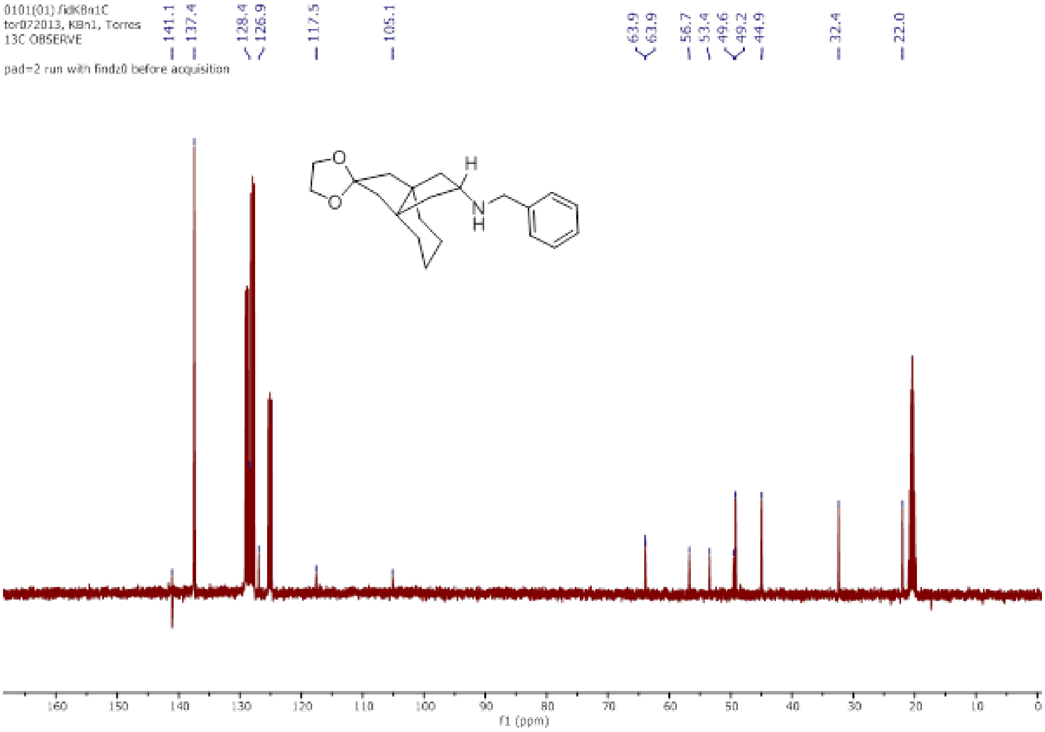

^{1}H NMR spectrum of (11'-*anti*)-Spiro-([1,3]-dioxolane-2,8'-(N-benzyl-[4.3.3]propellan))-11'-amine (*anti*-**14**)

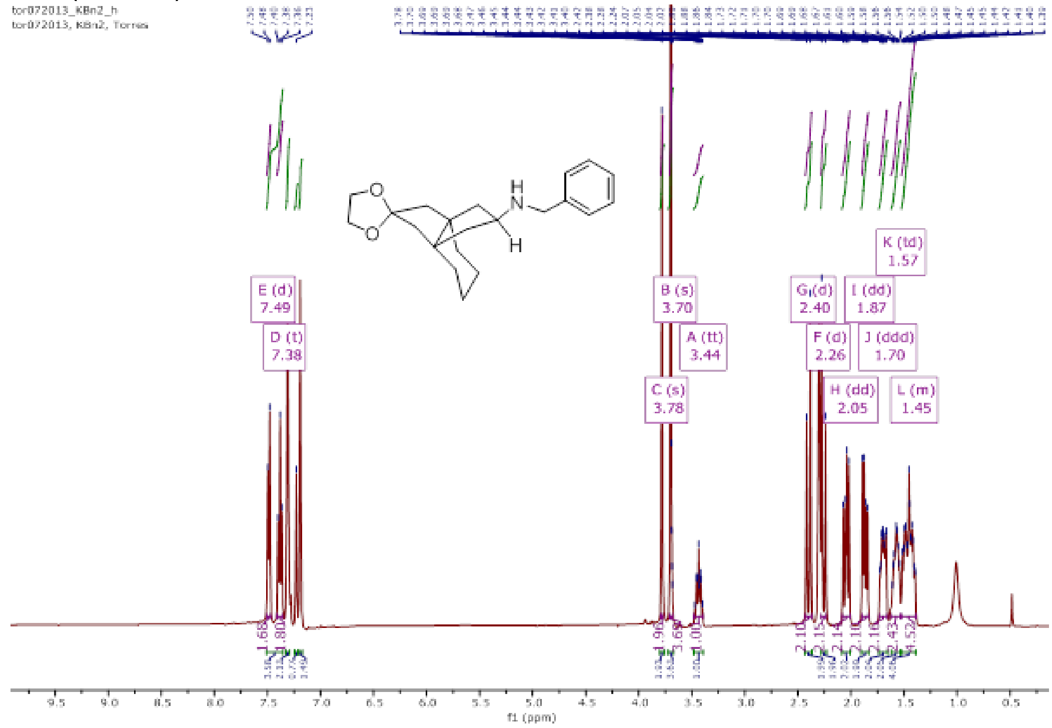

^{13}C NMR spectrum of (11'-*anti*)-Spiro-([1,3]-dioxolane-2,8'-(N-benzyl-[4.3.3]propellan))-11'-amine (*anti*-**14**)

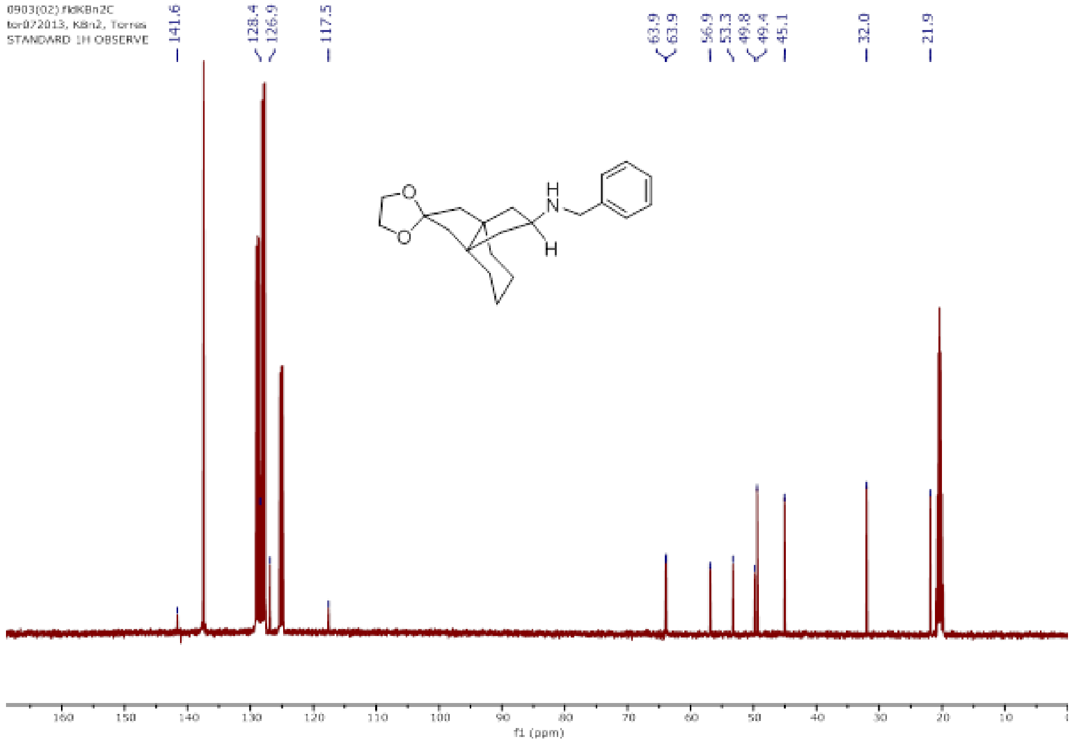

^{1}H NMR spectrum of *syn*- and *anti*-N-[2-(Indol-3-yl)ethyl]-[4.3.3]propellan-8-amine (**15**)

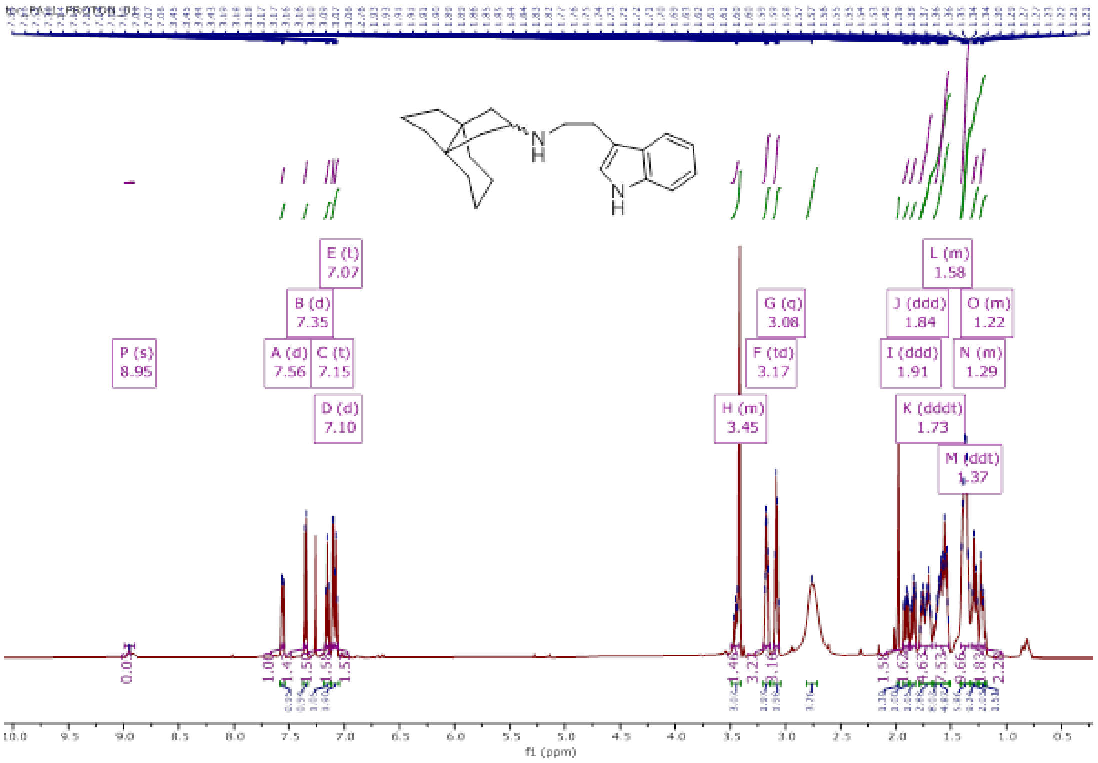

^{13}C NMR spectrum of *syn*- and *anti*-N-[2-(Indol-3-yl)ethyl]-[4.3.3]propellan-8-amine (**15**)

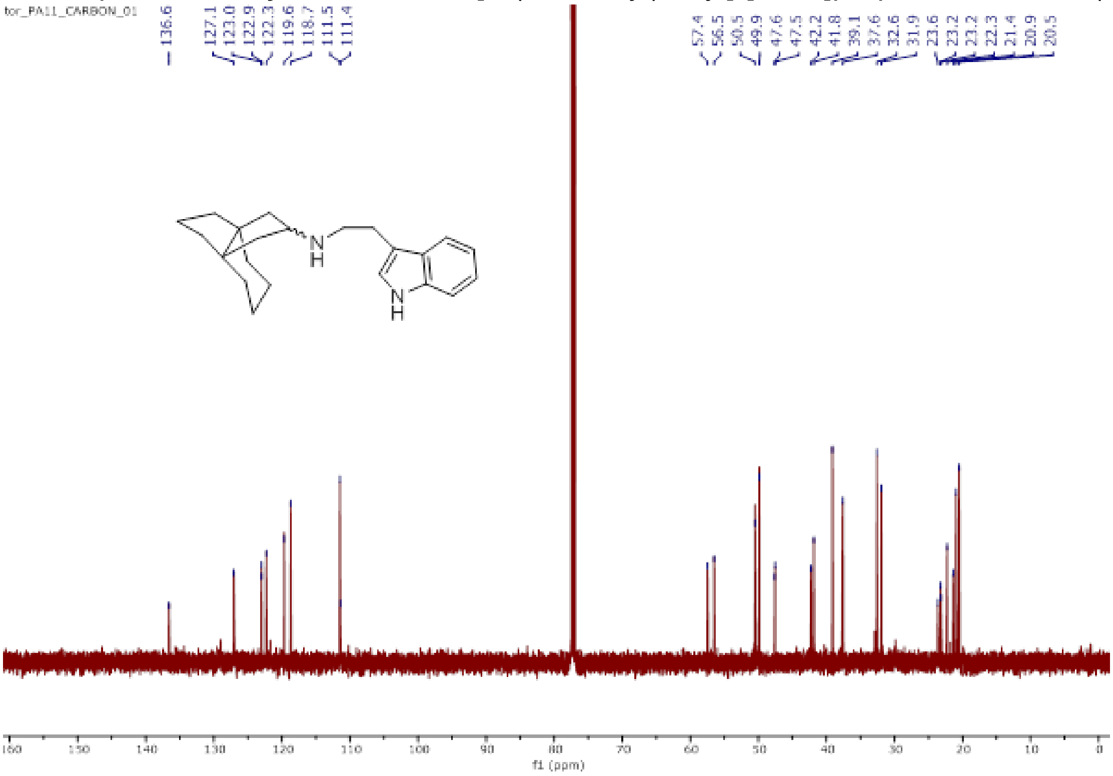

^{1}H NMR spectrum of *syn*- and *anti*-N-(4-(Dimethylaminobenzyl)-[4.3.3]propellan-8-amine (**17**)

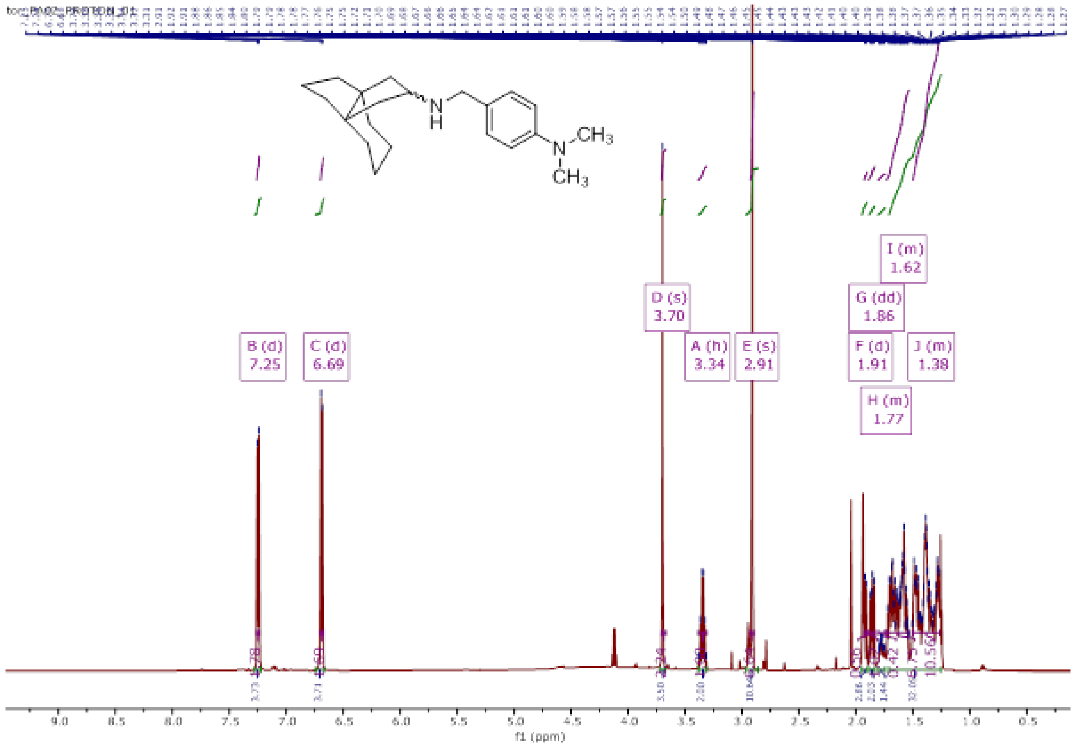

^{13}C NMR spectrum of *syn*- and *anti*-N-(4-(Dimethylaminobenzyl)-[4.3.3]propellan-8-amine (**17**)

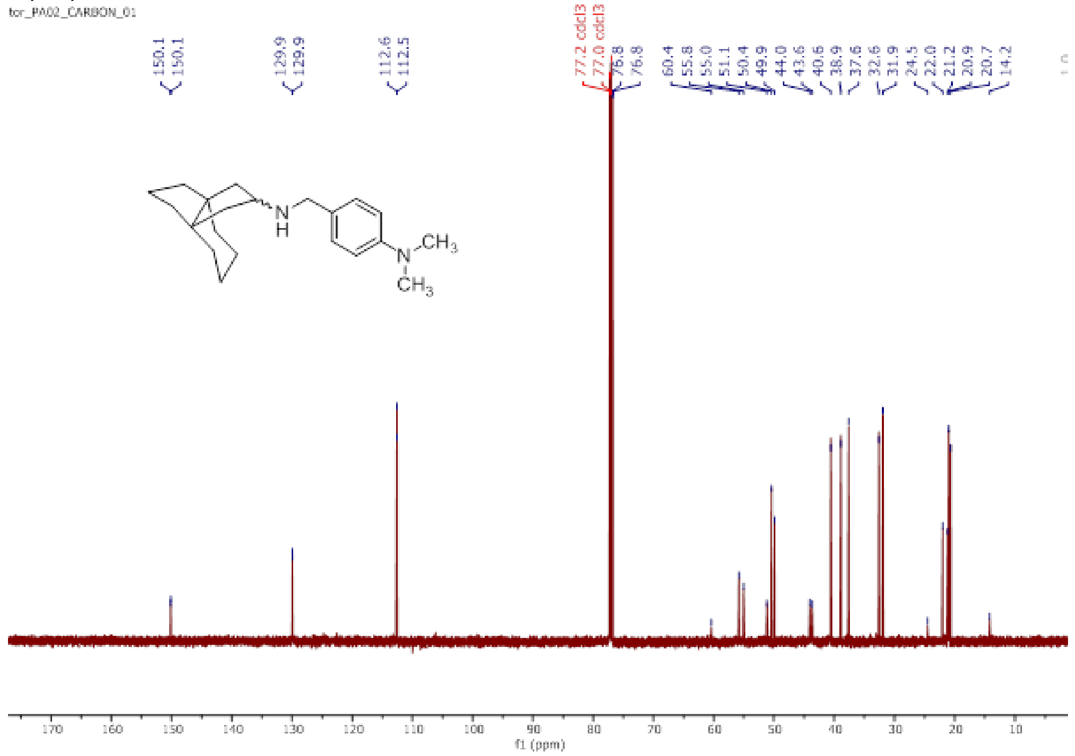

^{1}H NMR spectrum of *syn*- and *anti*-N-cyclohexylmethyl-[4.3.3]propellan-8-amine (**18**)

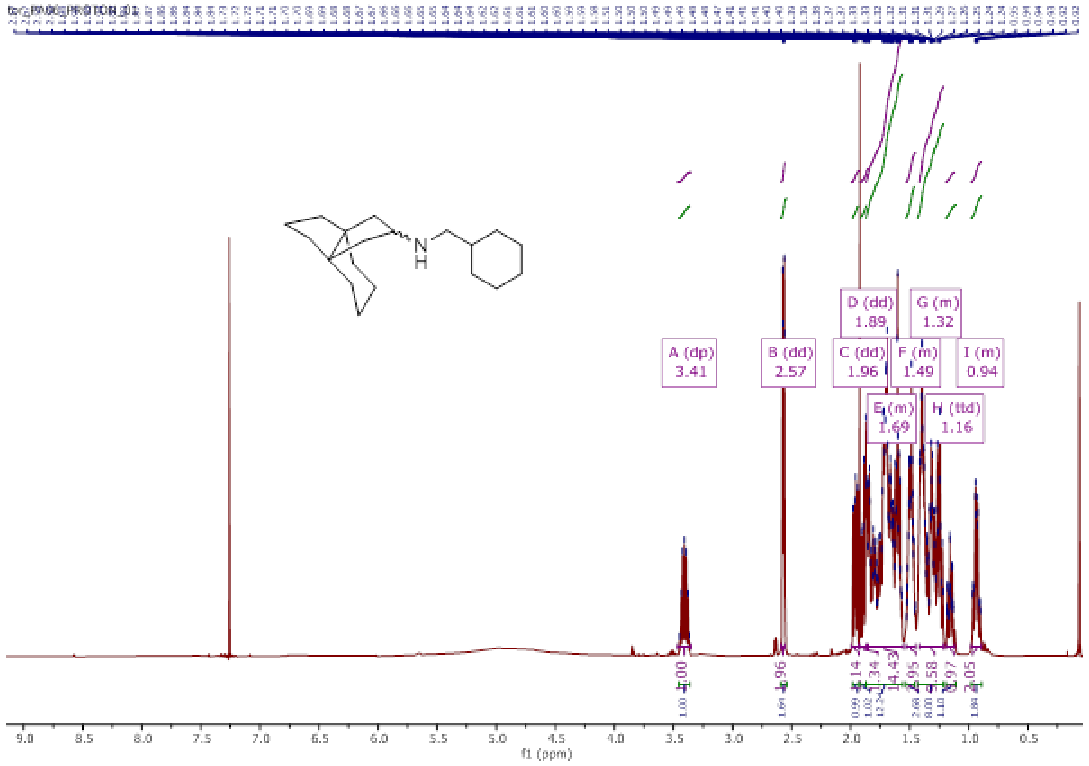

^{13}C NMR spectrum of *syn*- and *anti*-N-cyclohexylmethyl-[4.3.3]propellan-8-amine (**18**)

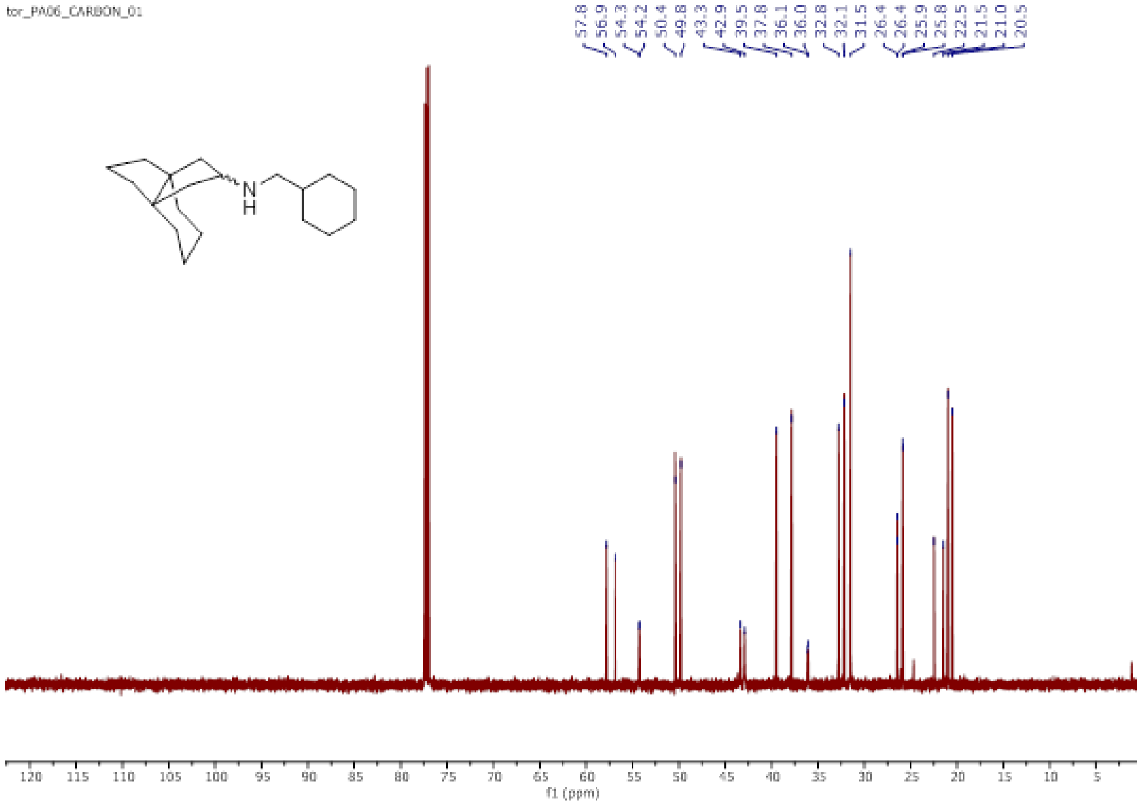

^{1}H NMR spectrum of 11-*syn*-11-Benzylamino[4.3.3]propellan-11-one (*syn*-**20**)

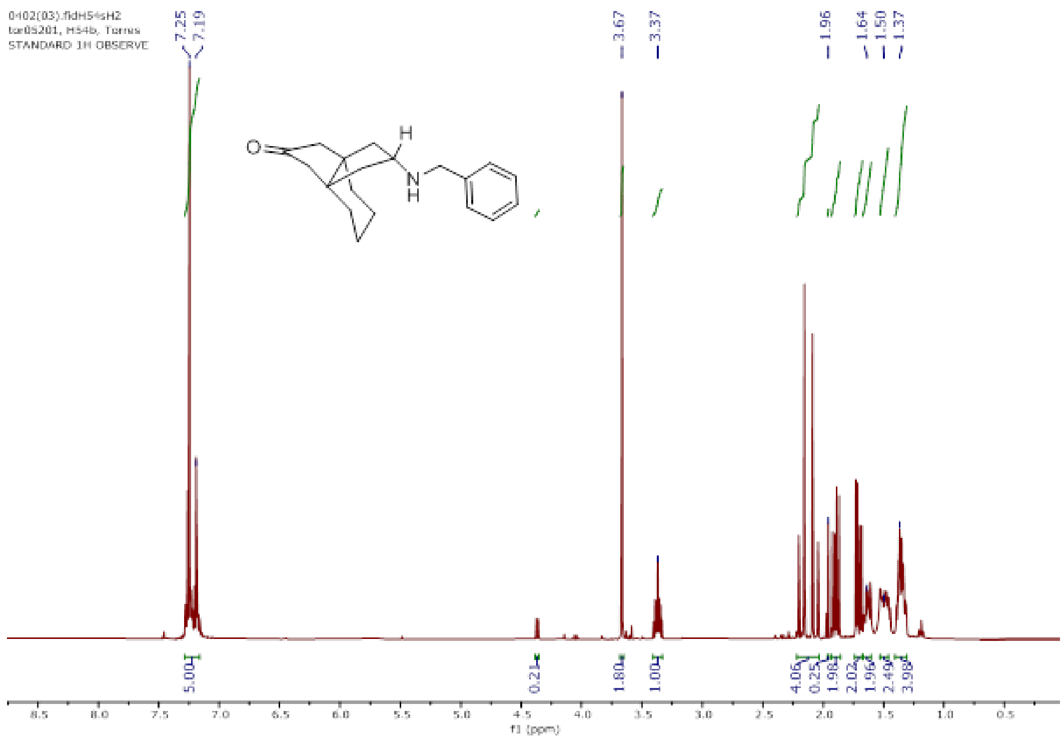

^{13}C NMR spectrum of 11-*syn*-11-Benzylamino[4.3.3]propellan-11-one (*syn*-**20**)

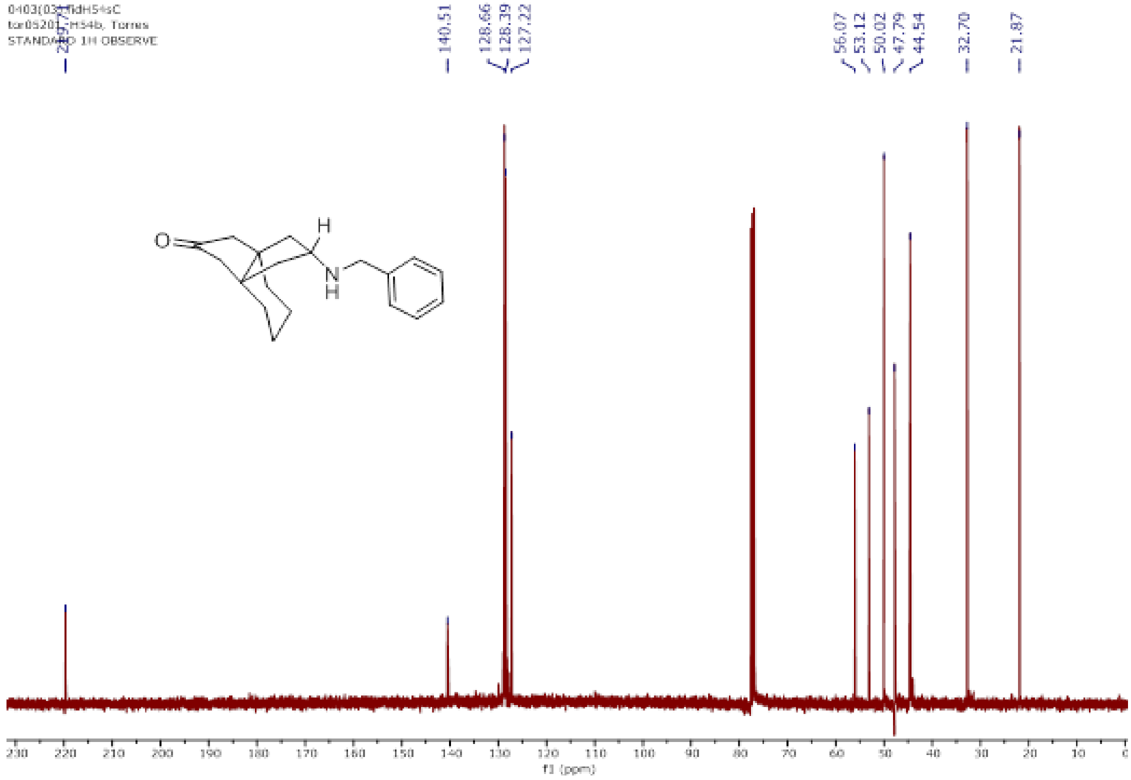

^{1}H NMR spectrum of 11-*anti*-11-Benzylamino[4.3.3]propellan-8-one (*anti*-**20**)

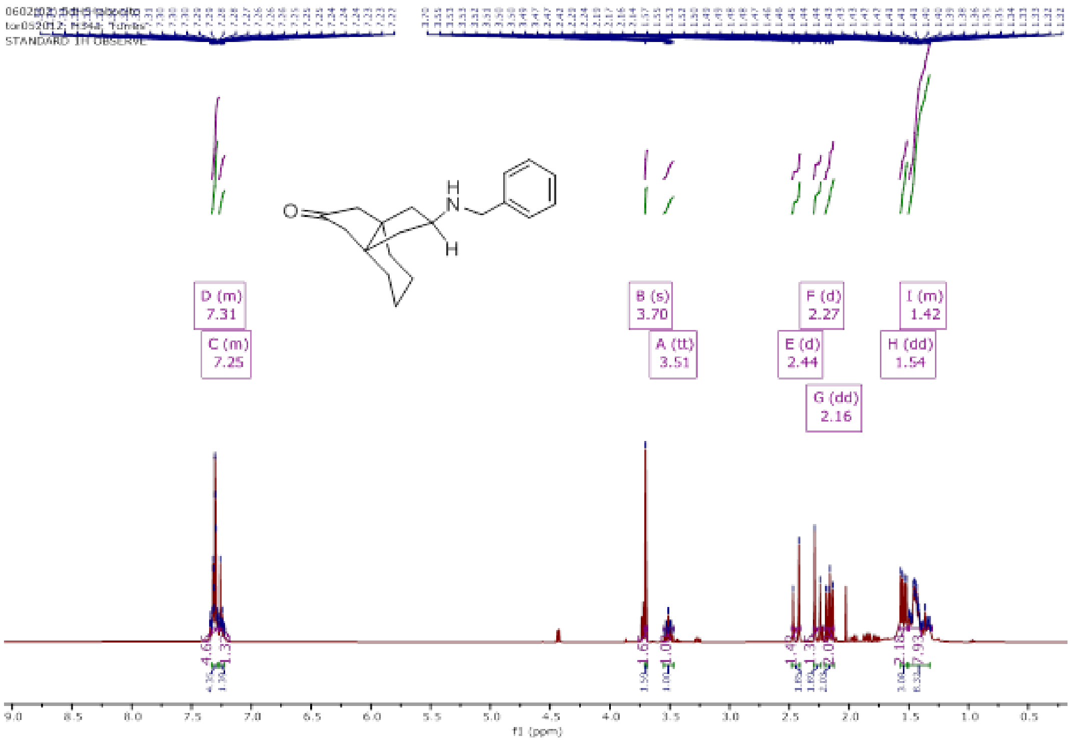

^{13}C NMR spectrum of 11-*anti*-11-Benzylamino[4.3.3]propellan-8-one (*anti*-**20**)

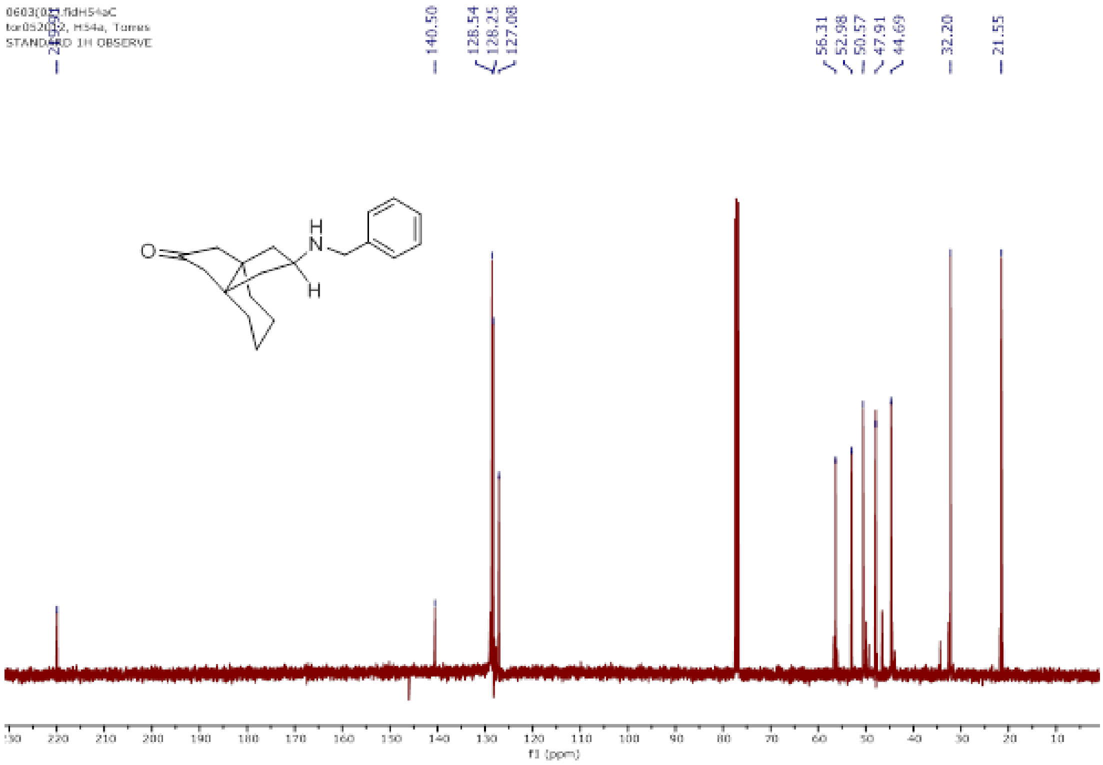

^{1}H NMR spectrum of 1-Benzyl-3-(2-Methoxy-5-methylphenyl)-1-(*syn*-11-oxo-[4.3.3]propellan-8-yl)urea (*syn*-**21**)

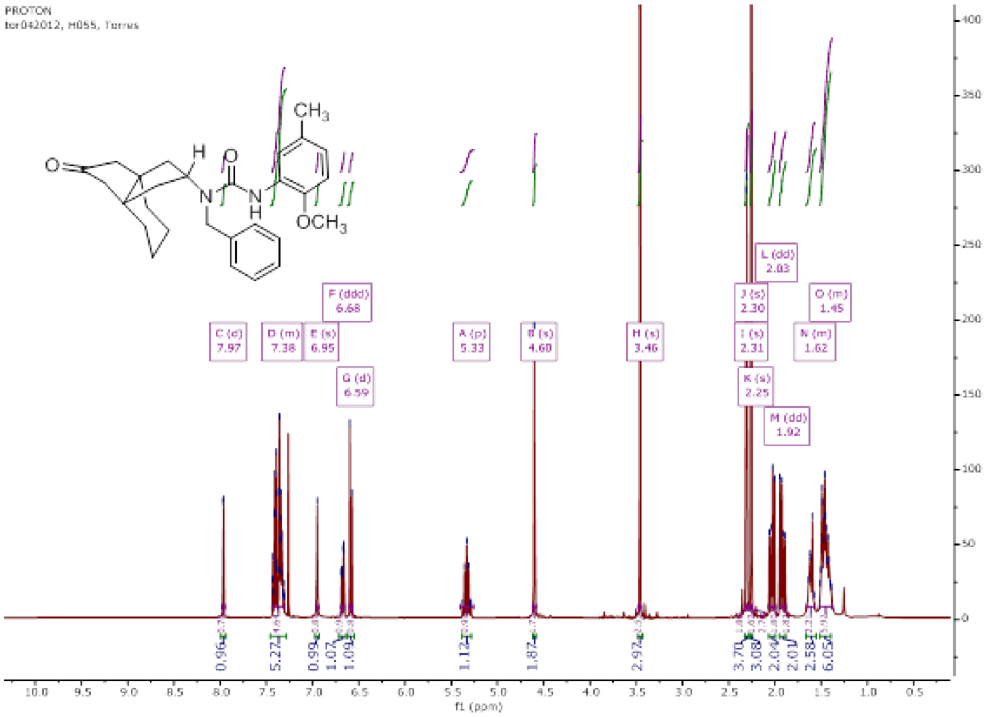

^{13}C NMR spectrum of 1-Benzyl-3-(2-Methoxy-5-methylphenyl)-1-(*syn*-11-oxo-[4.3.3]propellan-8-yl)urea (*syn*-**21**)

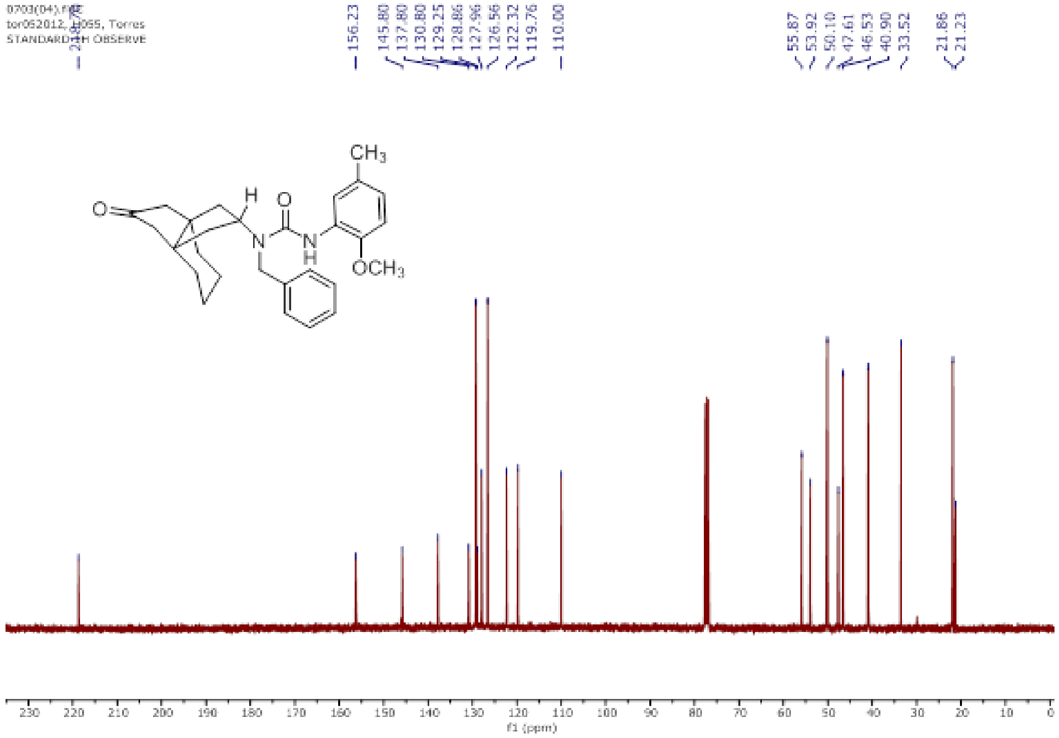

^{1}H NMR spectrum of 1-Benzyl-3-(2-Methoxy-5-methylphenyl)-1-(*anti*-11-oxo-[4.3.3]propellan-8-yl)urea (*anti*-**21**)

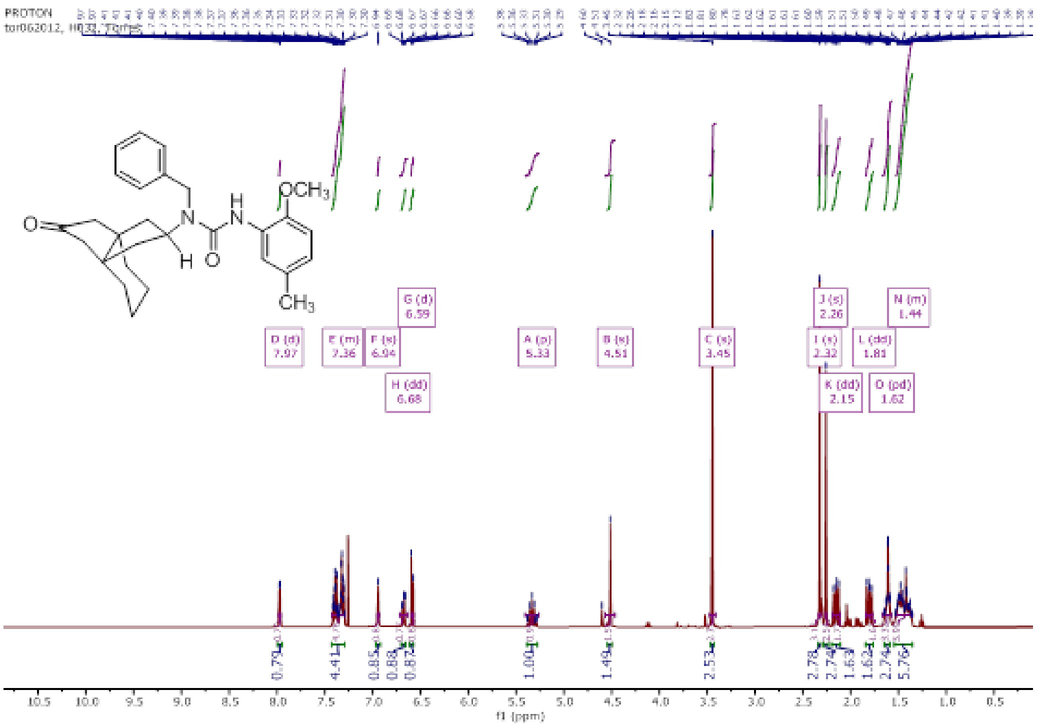

^{13}C NMR spectrum of 1-Benzyl-3-(2-Methoxy-5-methylphenyl)-1-(*anti*-11-oxo-[4.3.3]propellan-8-yl)urea (*anti*-**21**)

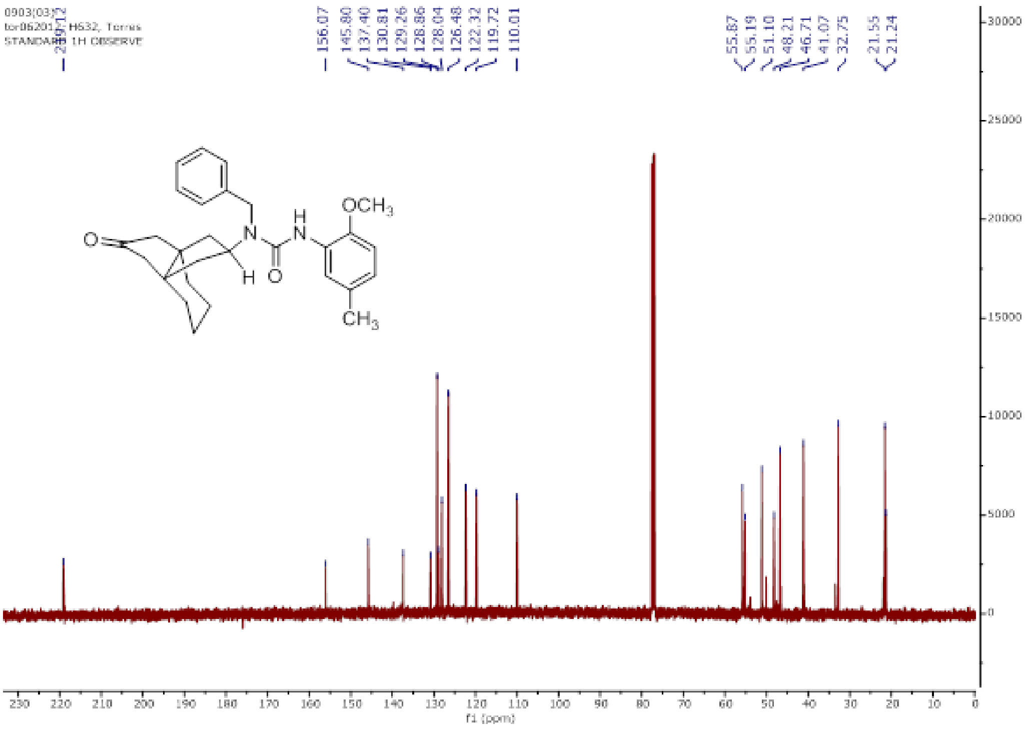

^{1}H NMR spectrum of 1-Benzyl-1-[(8-*syn*, 11-*anti*)-11-hydroxy[4.3.3]propellan-8yl]-3-(2-methoxy-5-methylphenyl)urea(*syn,anti*-**22**)

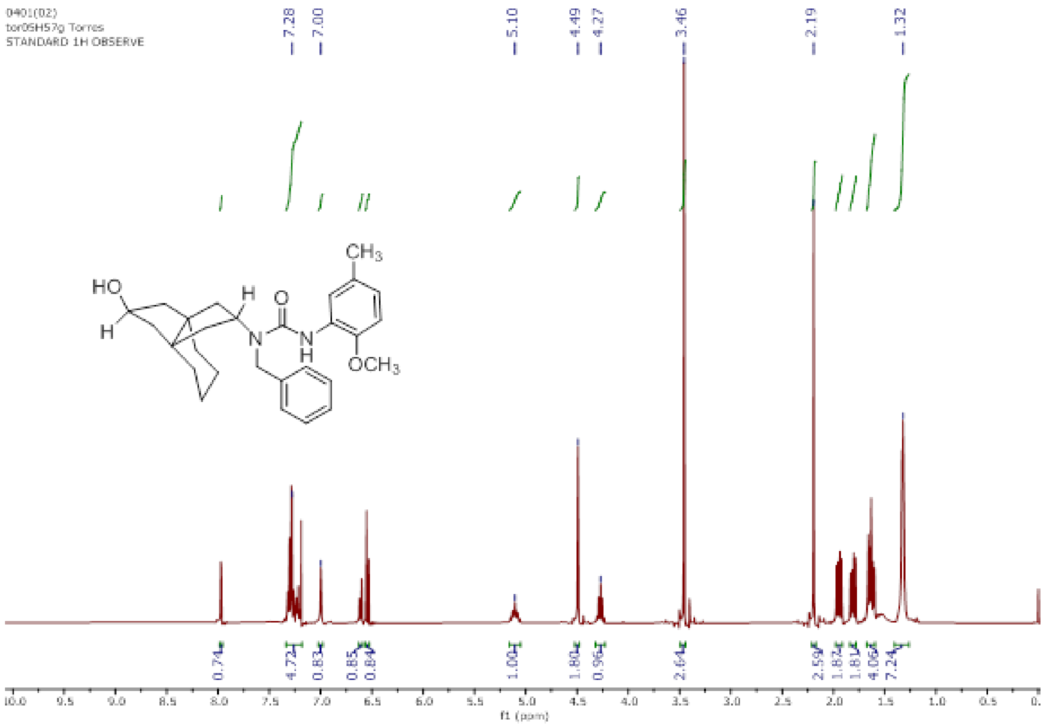

^{13}C NMR spectrum of 1-Benzyl-1-[(8-*syn*, 11-*anti*)-11-hydroxy[4.3.3]propellan-8yl]-3-(2-methoxy-5-methylphenyl)urea(*syn,anti*-**22**)

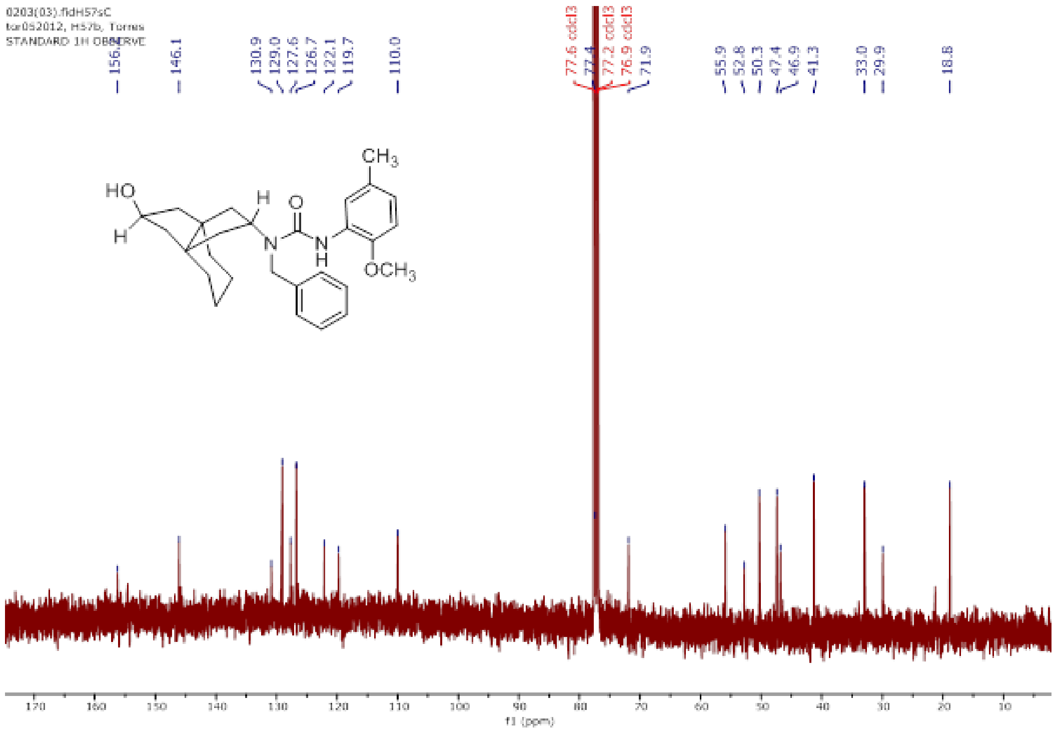

^{1}H NMR spectrum of 1-benzyl-1-[(8-*syn*, 11-*syn*)-11-hydroxy[4.3.3]propellan-8yl)]-3-(2-methoxy-5-methylphenyl)urea (*syn,syn*-**22**)

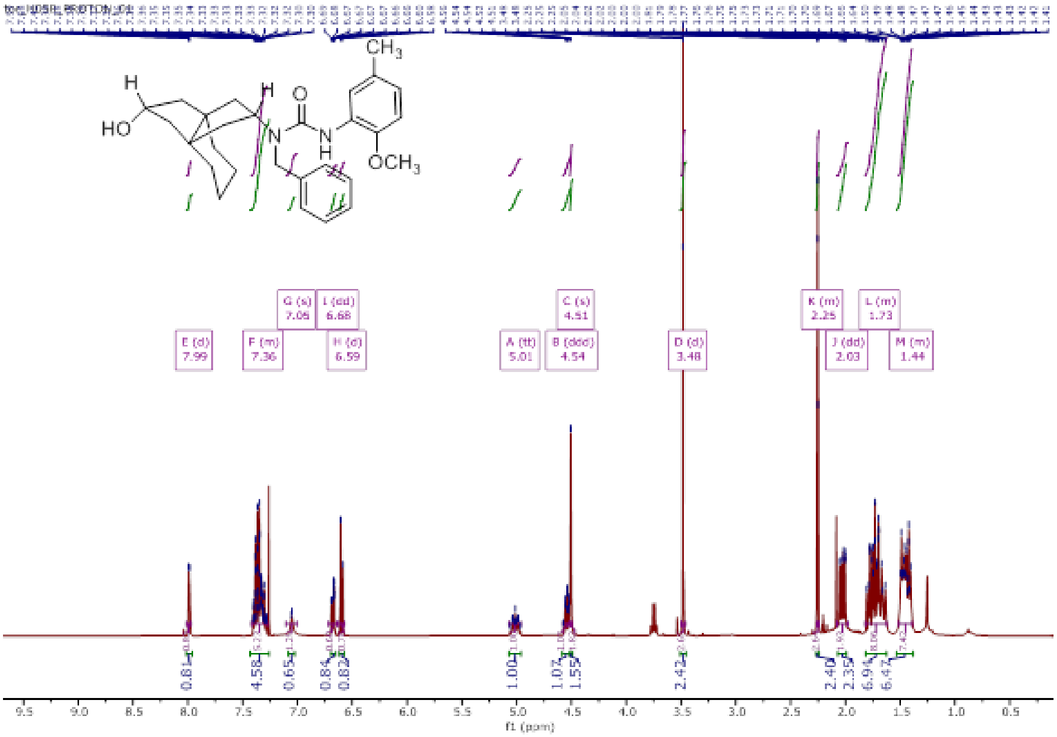

^{13}C NMR spectrum of 1-benzyl-1-[(8-*syn*, 11-*syn*)-11-hydroxy[4.3.3]propellan-8yl)]-3-(2-methoxy-5-methylphenyl)urea (*syn,syn*-**22**)

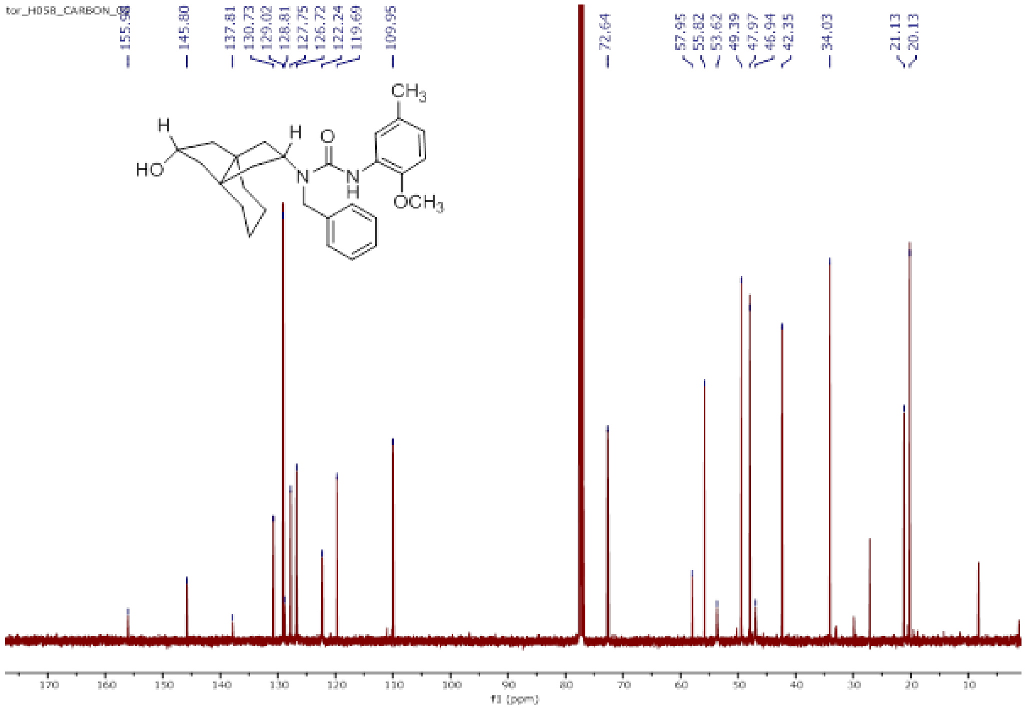

^{1}H NMR spectrum of 1-Benzyl-1-[(8-*anti*, 11-*anti*)-11-hydroxy[4.3.3]propellan-8yl]-3-(2-methoxy-5-methylphenyl)urea (*anti,anti*-**22**)

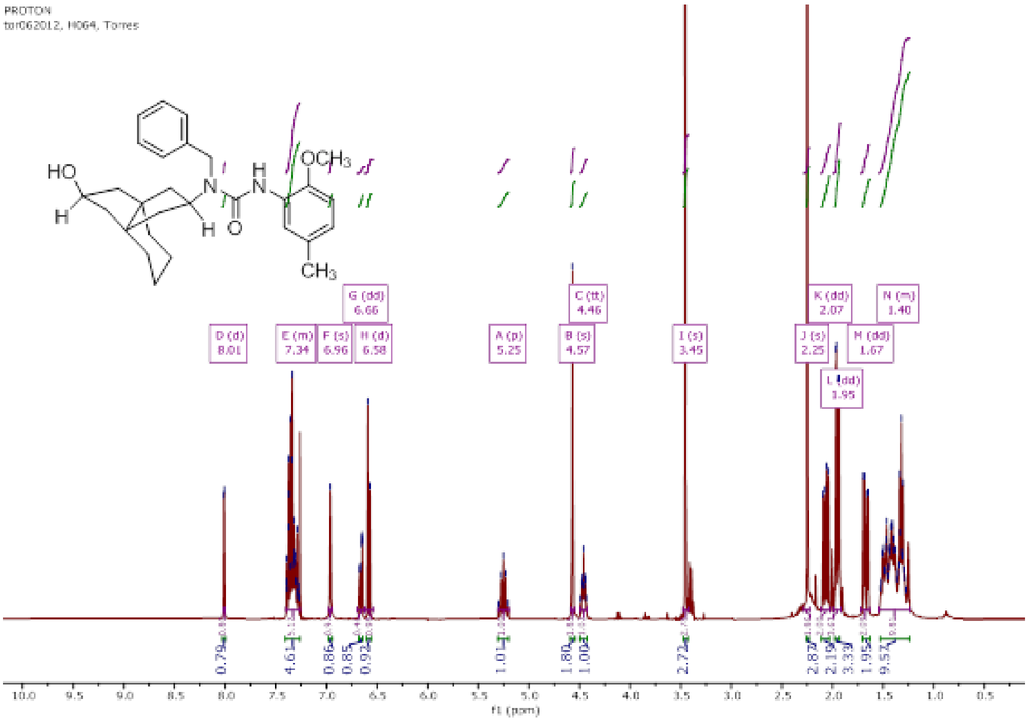

^{13}C NMR spectrum of 1-Benzyl-1-[(8-*anti*, 11-*anti*)-11-hydroxy[4.3.3]propellan-8yl]-3-(2-methoxy-5-methylphenyl)urea (*anti,anti*-**22**)

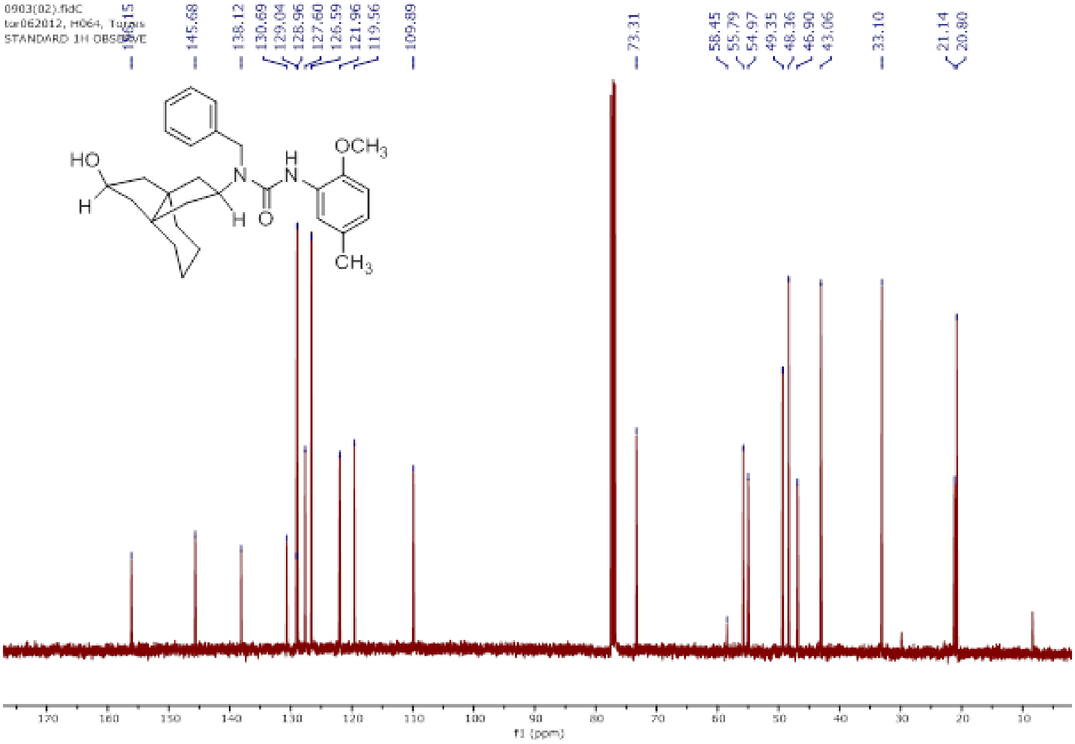

^{1}H NMR spectrum of 1-benzyl-1-[(8-syn, 11-*anti*)-11-hydroxy[4.3.3]propellan-8yl]-3-(2-methoxy-5-methylphenyl)urea (*anti,syn*-**22**)

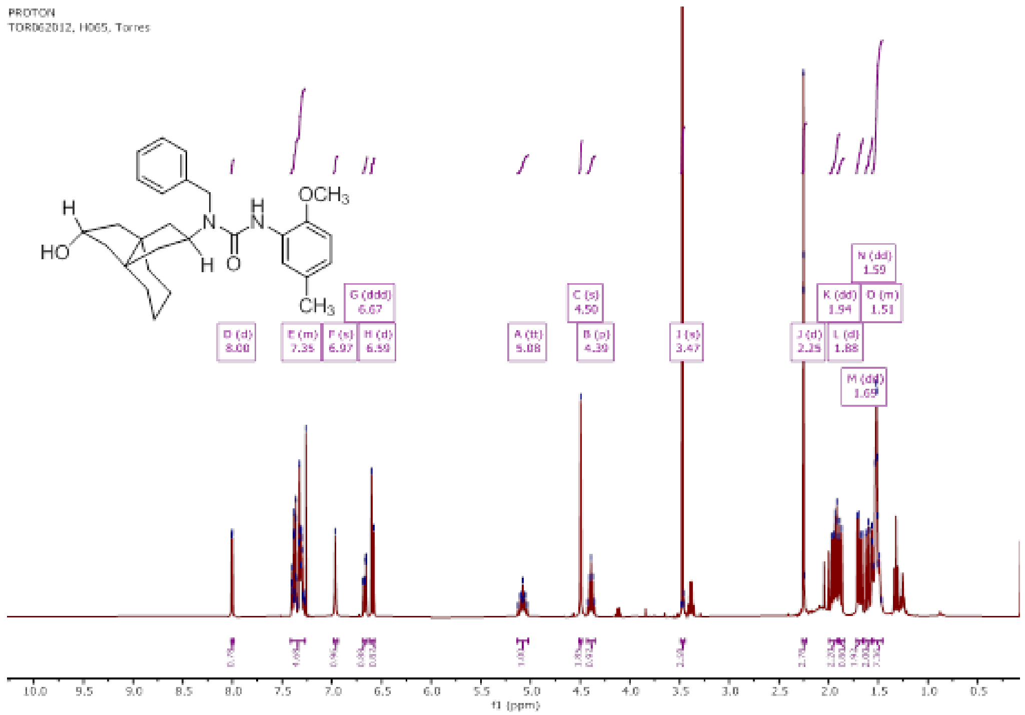

^{13}C NMR spectrum of 1-benzyl-1-[(8-syn, 11-*anti*)-11-hydroxy[4.3.3]propellan-8yl]-3-(2-methoxy-5-methylphenyl)urea (*anti,syn*-**22**)

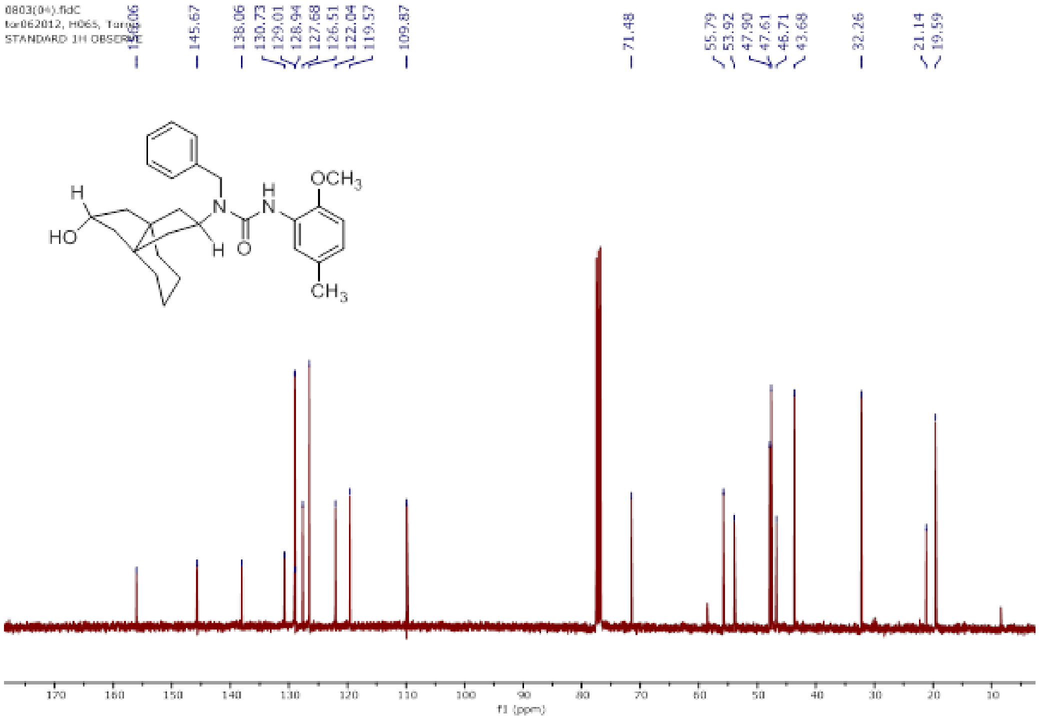

^{1}H NMR spectrum of 1-Phenyl-3-(*syn* and *anti*-[4.3.3]propellan-8-yl)urea (**23a**)

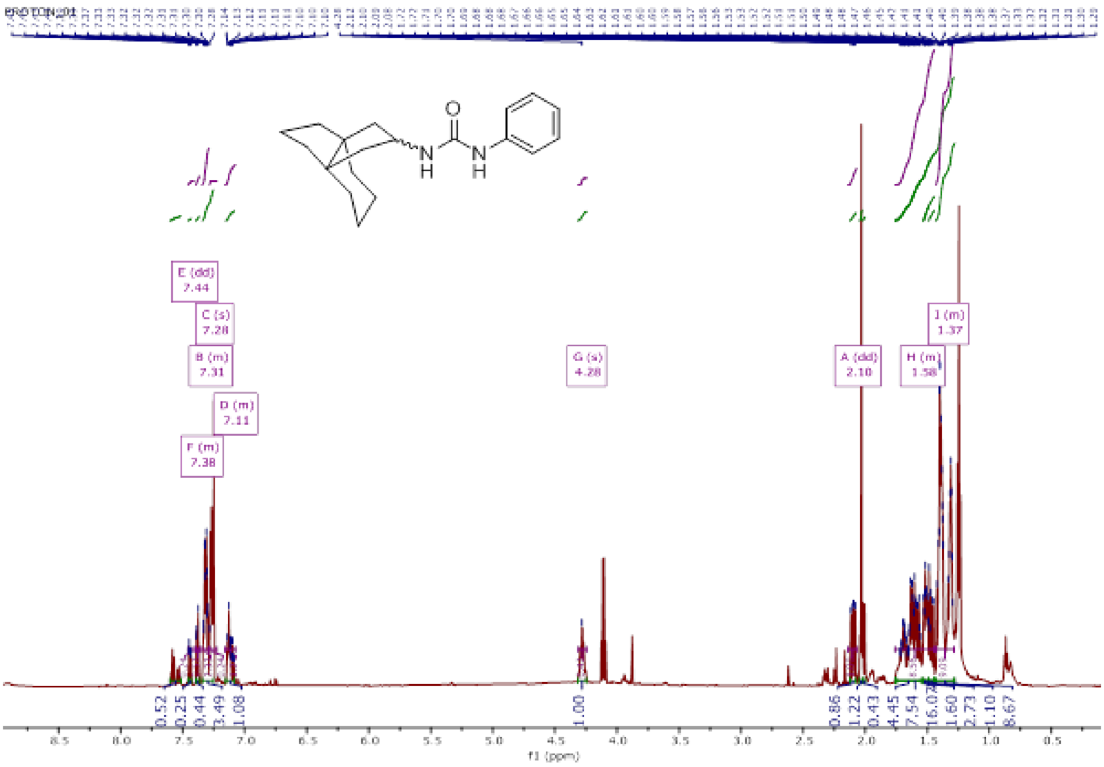

^{13}C NMR spectrum of 1-Phenyl-3-(*syn* and *anti*-[4.3.3]propellan-8-yl)urea (**23a**)

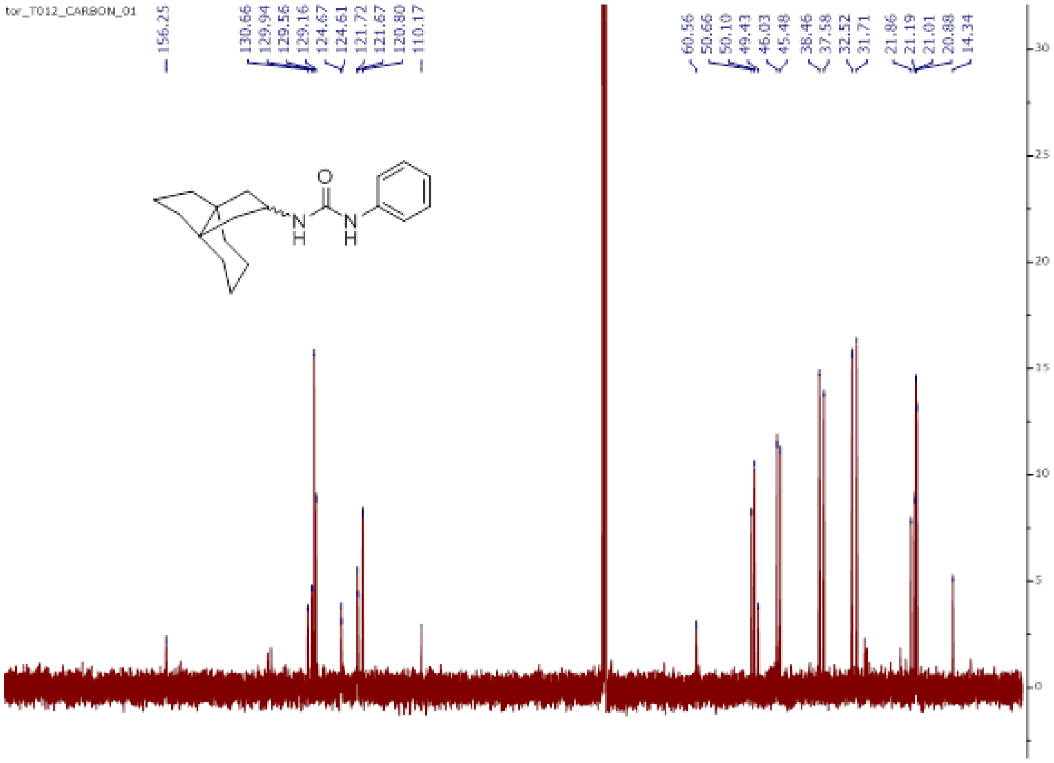

^{1}H NMR spectrum of 1-Cyclohexyl-3-(*syn* and *anti*-[4.3.3]propellan-8-yl)urea (**23b**)

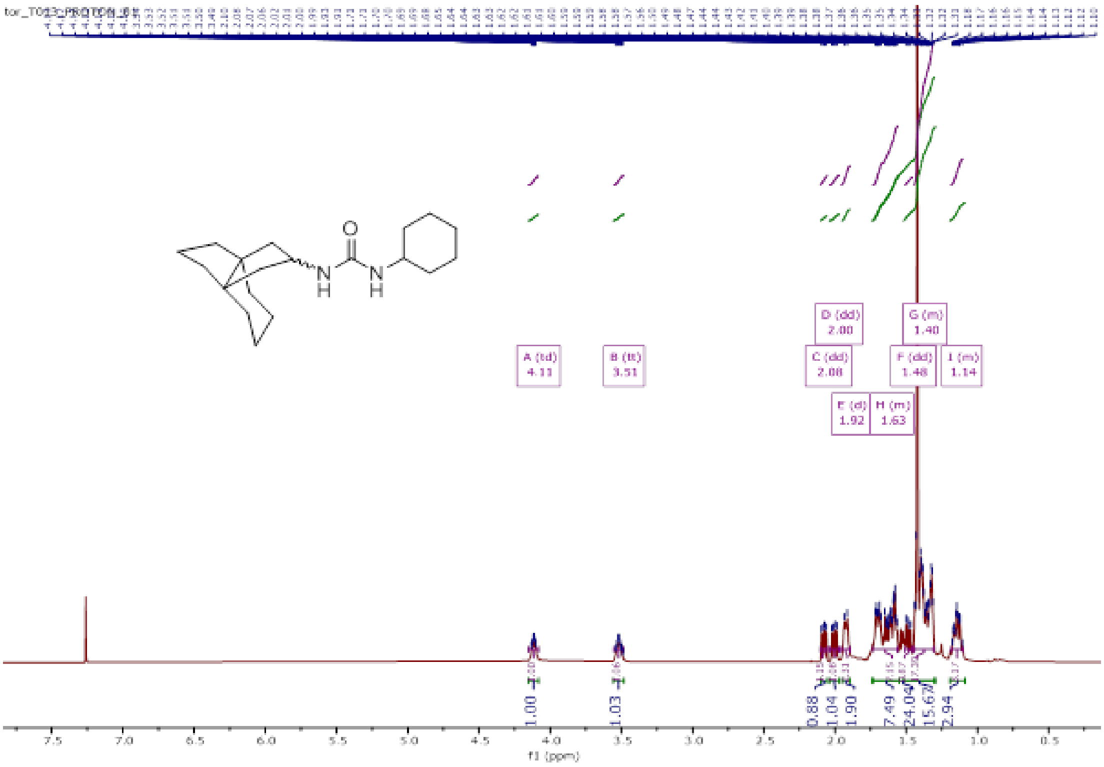

^{13}C NMR spectrum of 1-Cyclohexyl-3-(*syn* and *anti*-[4.3.3]propellan-8-yl)urea (**23b**)

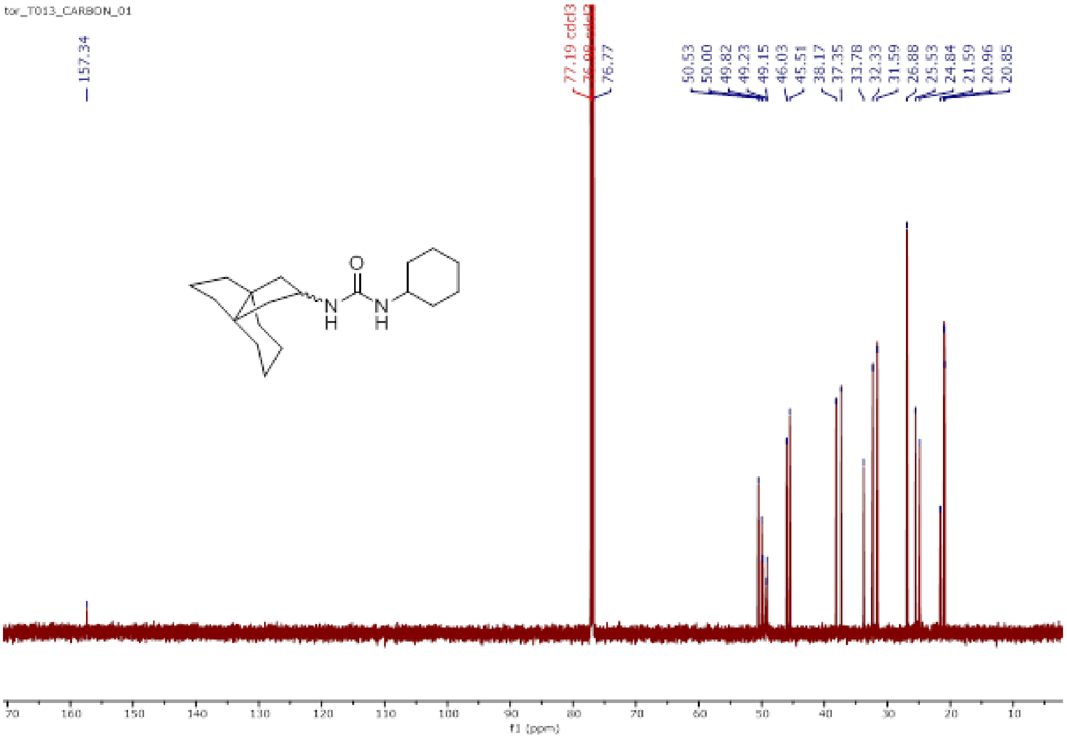

^{1}H NMR spectrum of 1-(2-Methoxy-5-methylphenyl)-3 –(*syn* and *anti*-[4.3.3]propellan-8-yl)urea (**23c**)

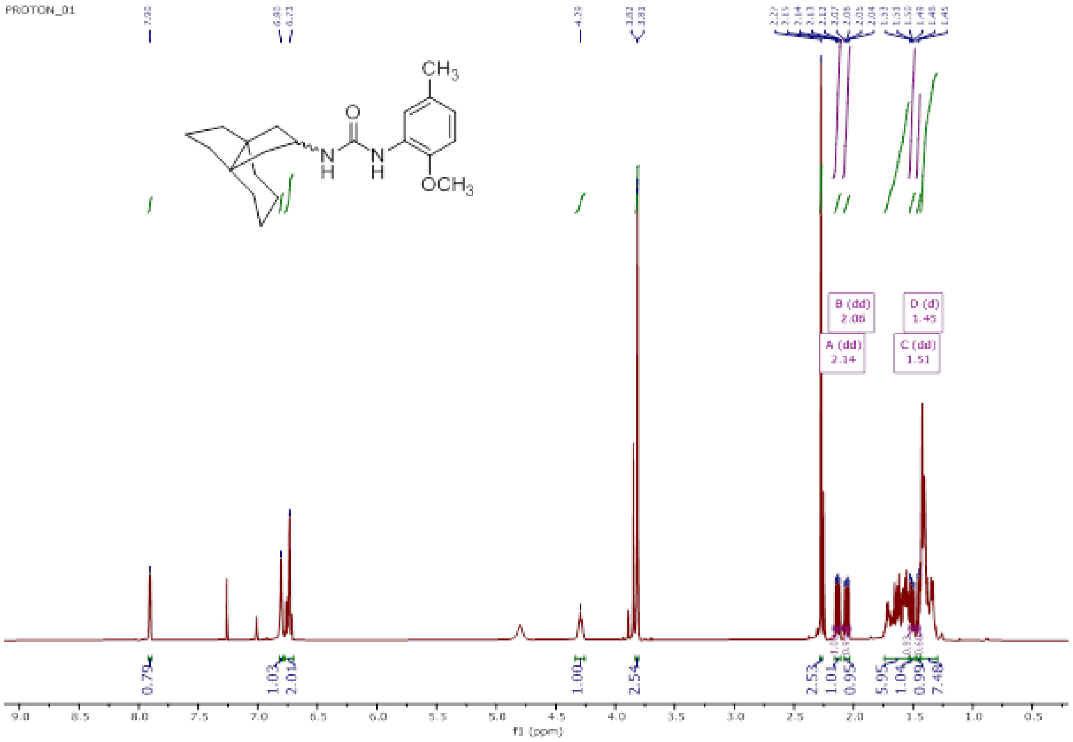

^{13}C NMR spectrum of 1-(2-Methoxy-5-methylphenyl)-3 –(*syn* and *anti*-[4.3.3]propellan-8-yl)urea (**23c**)

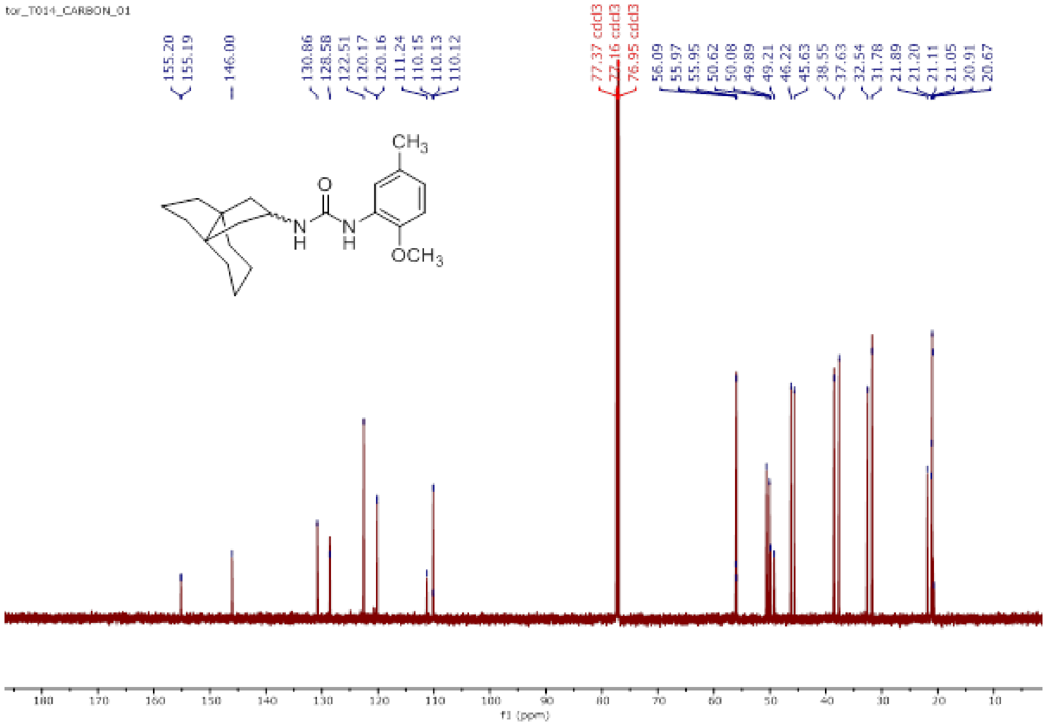

^{1}H NMR spectrum of 1-(3,4-difluorophenyl)-3-(*syn* and *anti*-[4.3.3]propellan-8-yl)urea (**23d**)

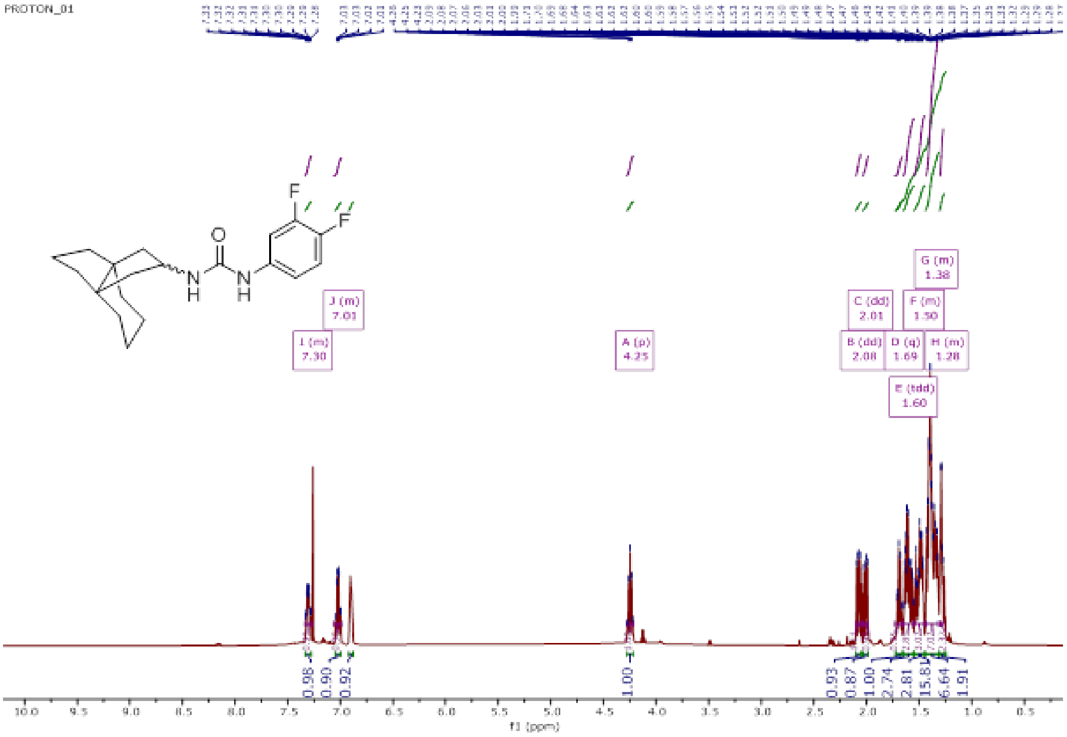

^{13}C NMR spectrum of 1-(3,4-difluorophenyl)-3-(*syn* and *anti*-[4.3.3]propellan-8-yl)urea (**23d**)

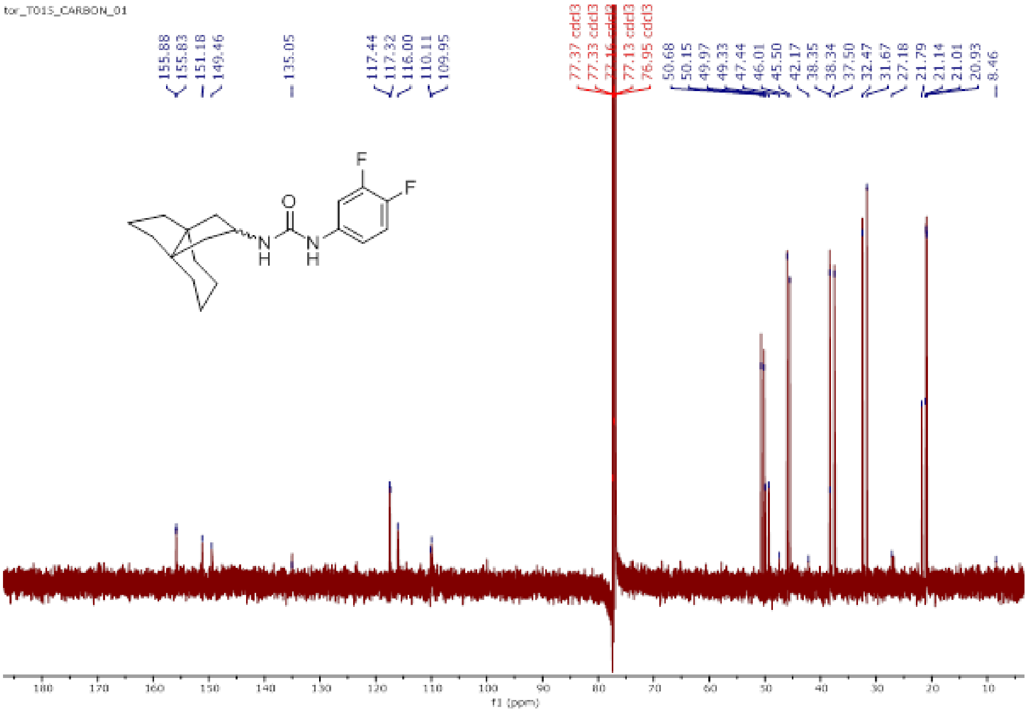

^{1}H NMR spectrum of (11’-*syn* and 11’-*anti*)-Spiro ([1,3]-dioxolane-2,8’-[4.3.3]propellan)-11’-amine (**24**)

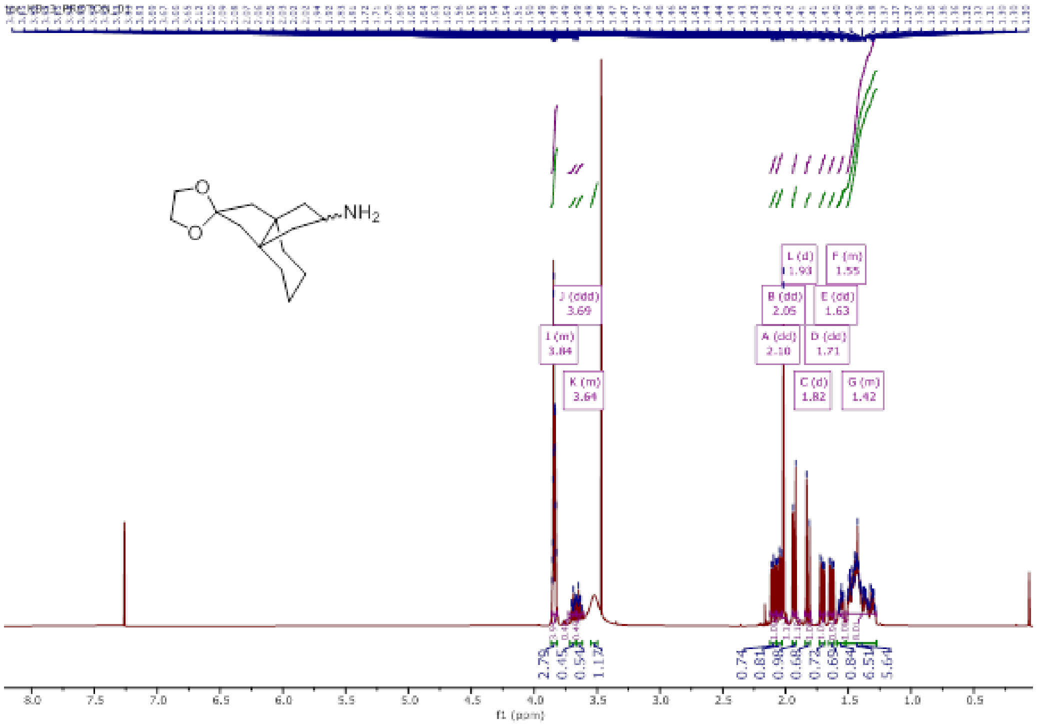

^{1}H NMR spectrum of 1-(3,4-Difluorophenyl)-3-(8-*syn*-11-oxo[4.3.3]propellan-8-yl)urea (*syn*-**25**)

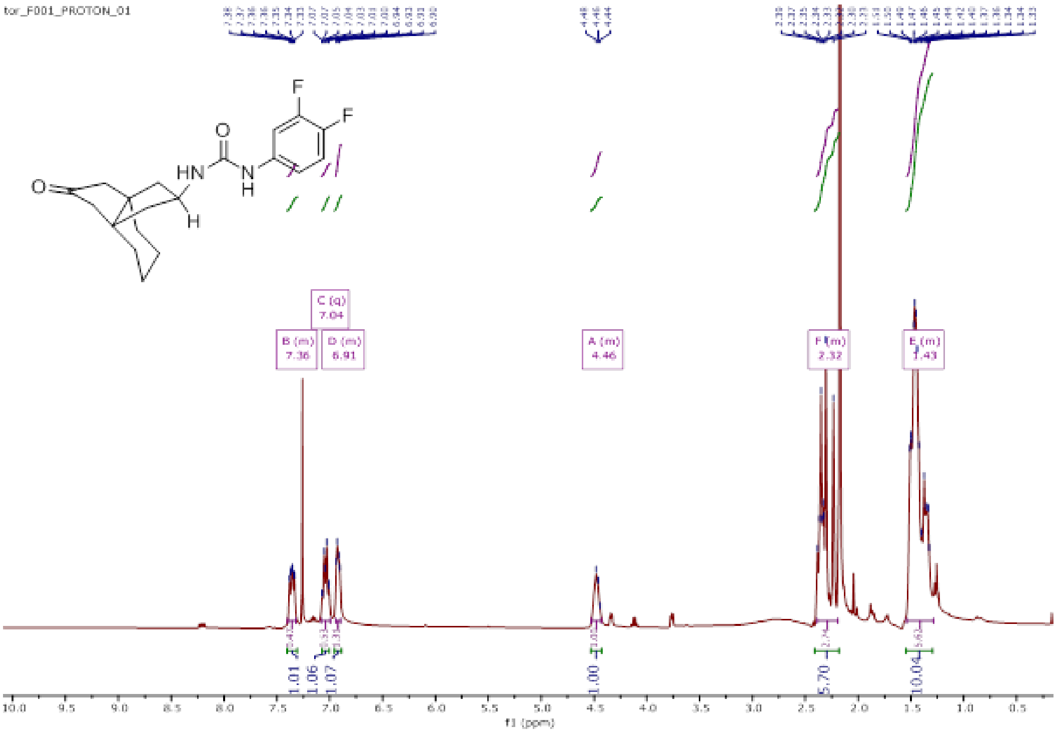

^{13}C NMR spectrum of 1-(3,4-Difluorophenyl)-3-(8-*syn*-11-oxo[4.3.3]propellan-8-yl)urea (*syn*-**25**)

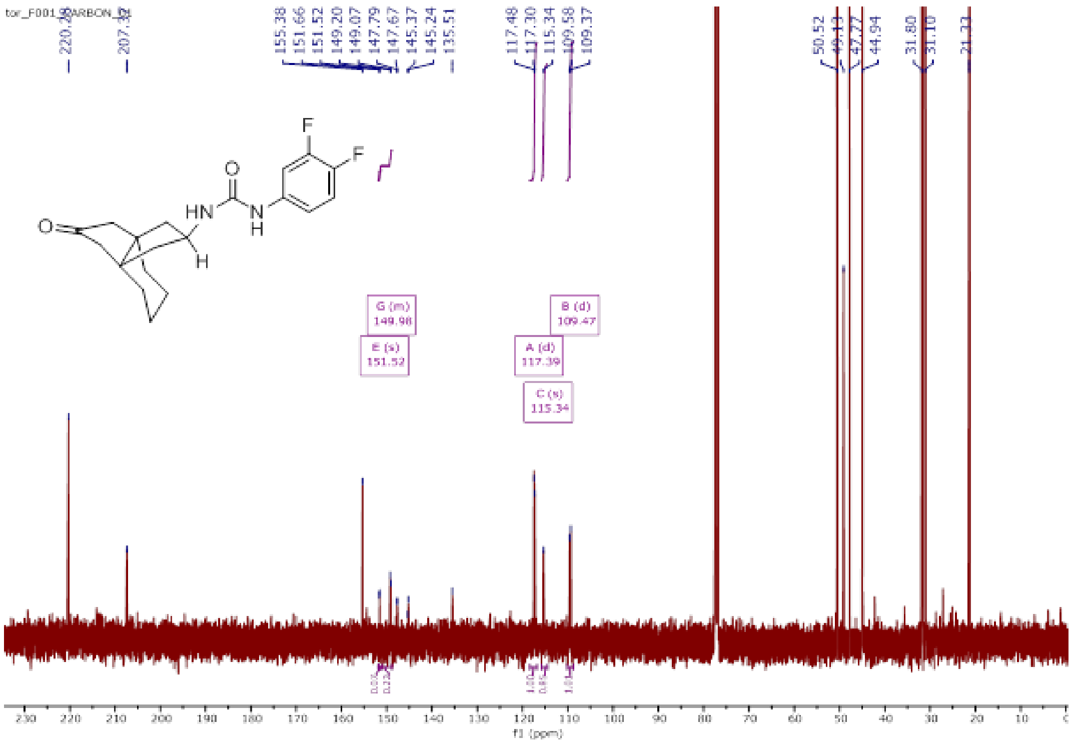

^{1}H NMR spectrum of 1-(3,4-Difluorophenyl)-3-(8-*anti*-11-oxo[4.3.3]propellan-8-yl)urea (*anti*-**25**)

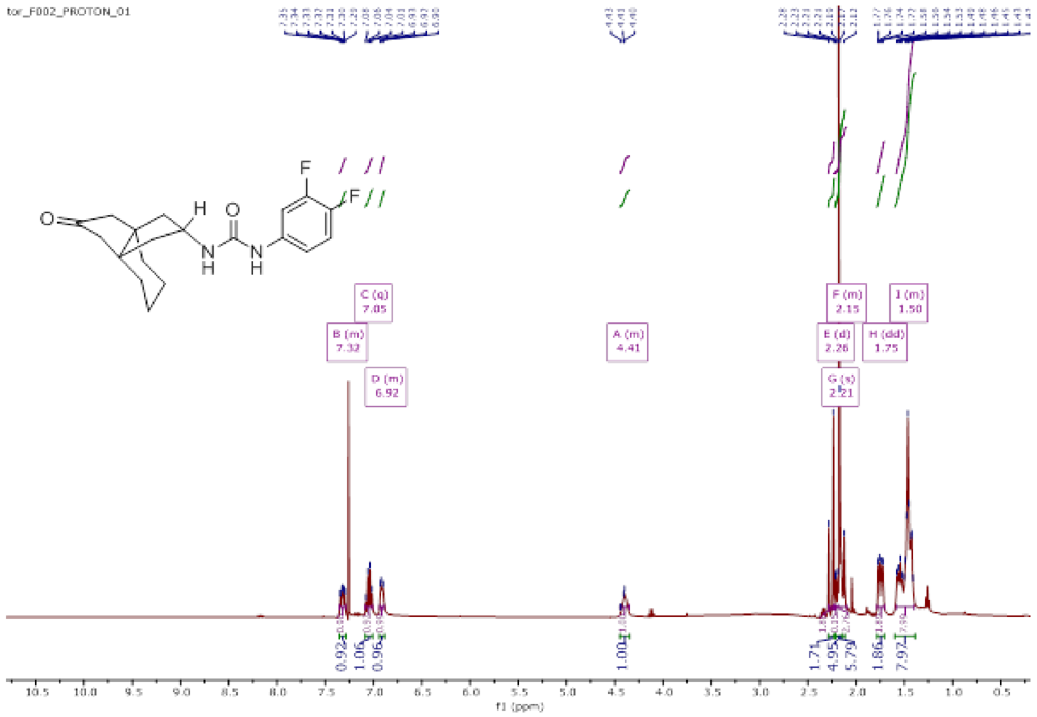

^{13}C NMR spectrum of 1-(3,4-Difluorophenyl)-3-(8-*anti*-11-oxo[4.3.3]propellan-8-yl)urea (*anti*-**25**)

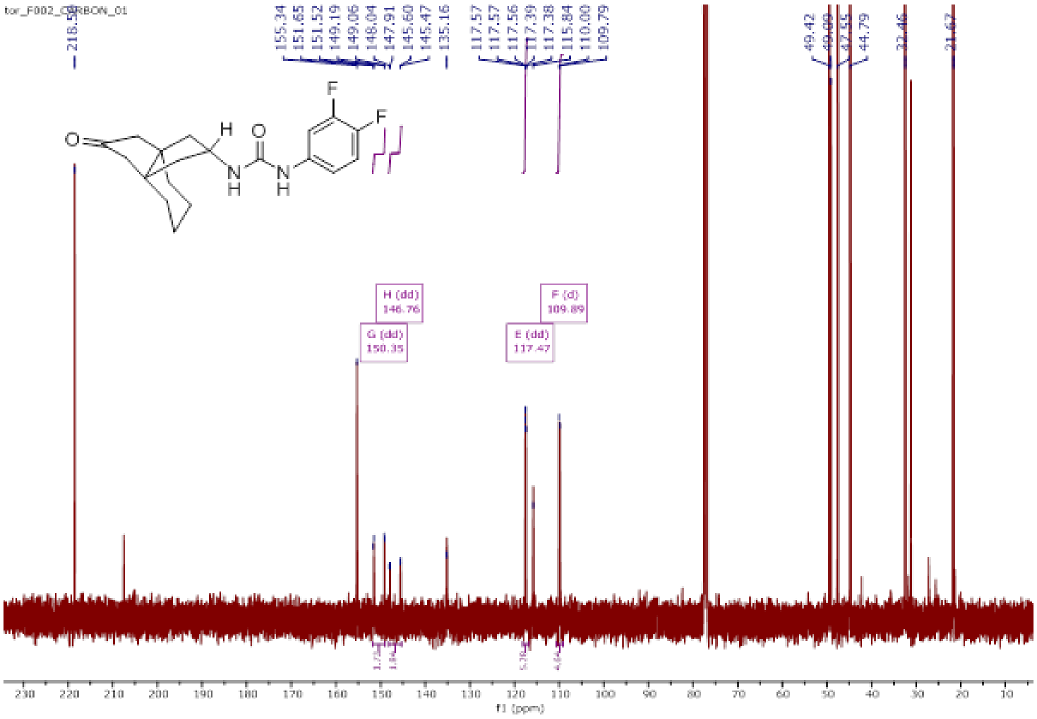

^{1}H NMR spectrum of 1-(3,4-Difluorophenyl)-3-(8-*anti*-11-*anti* and 8-*anti*-11-*syn*-11-hydroxy[4.3.3]propellan-8-yl)urea (*syn,syn*-**26** and *syn,anti*-**26**)

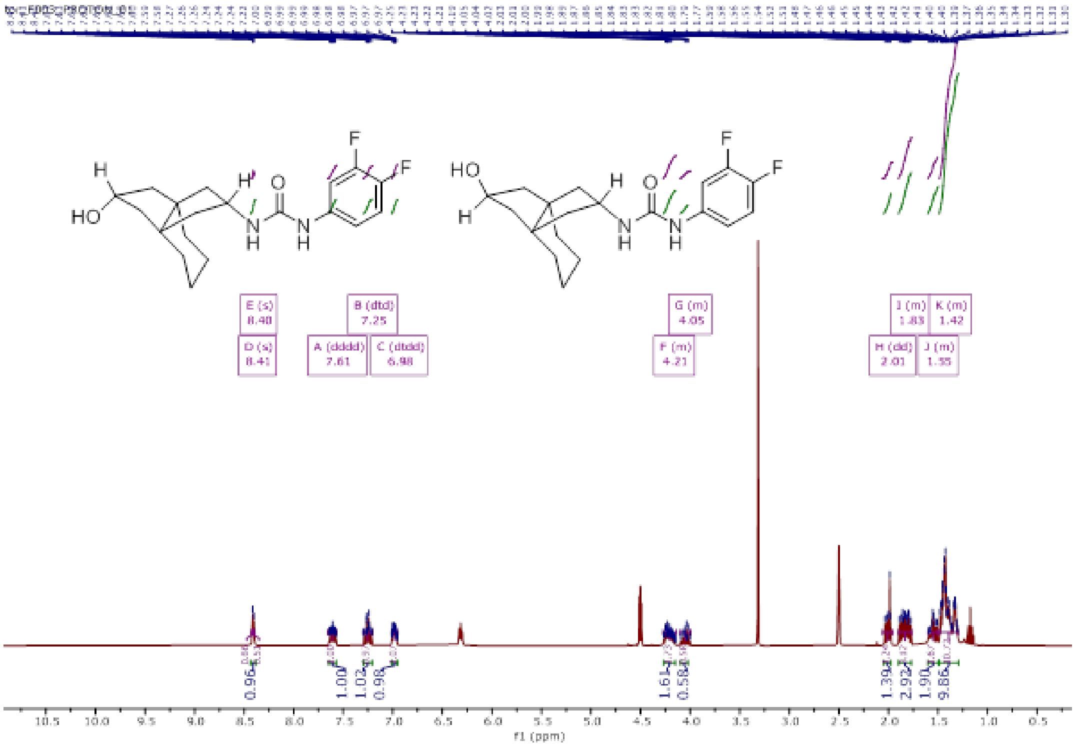

^{13}C NMR spectrum of 1-(3,4-Difluorophenyl)-3-(8-*anti*-11-*anti* and 8-*anti*-11-*syn*-11-hydroxy[4.3.3]propellan-8-yl)urea (*syn,syn*-**26** and *syn,anti*-**26**)

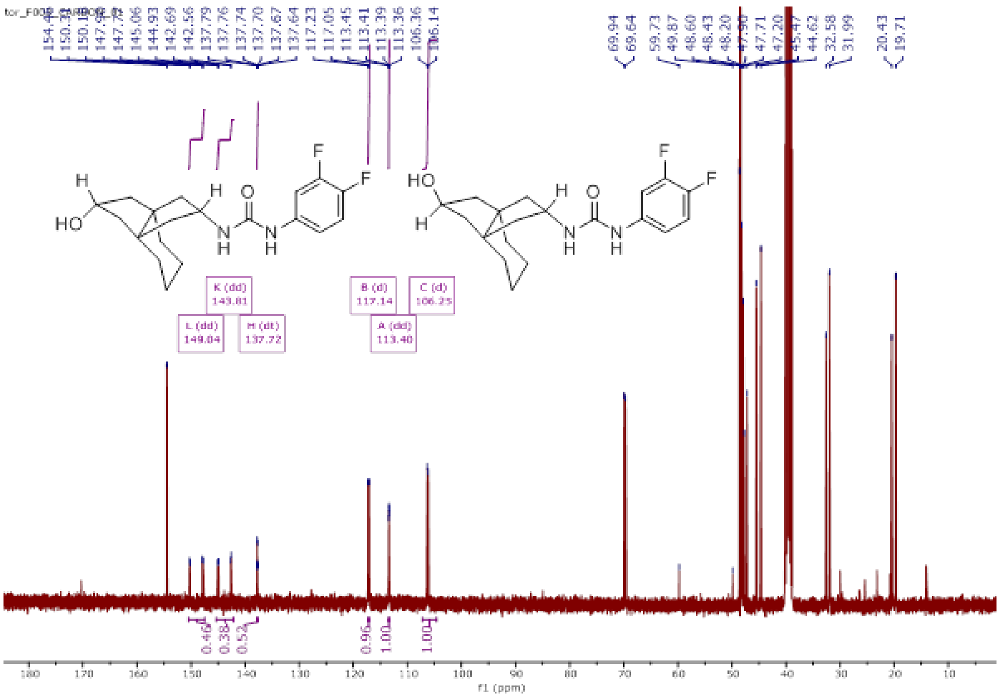

^{1}H NMR spectrum of 1-(3,4-Difluorophenyl)-3-(8-*anti*-11-*anti*-hydroxy[4.3.3]propellan-8-yl)urea (*anti,anti*-**26**)

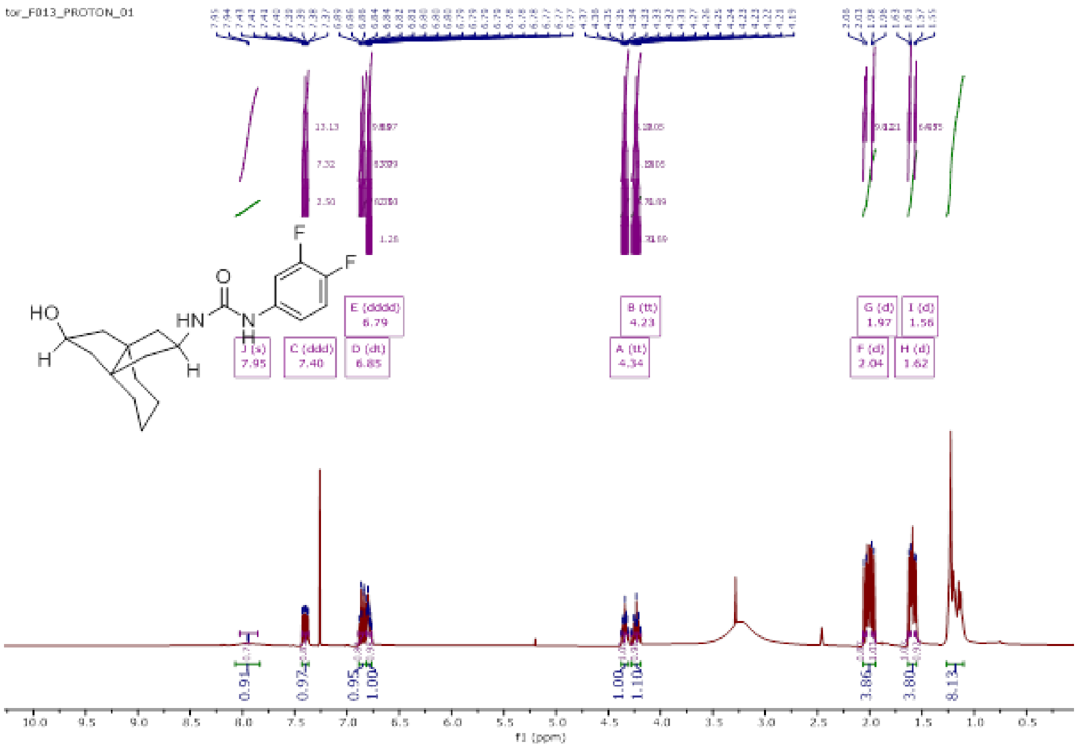

^{13}C NMR spectrum of 1-(3,4-Difluorophenyl)-3-(8-*anti*-11-*anti*-hydroxy[4.3.3]propellan-8-yl)urea (*anti,anti*-**26**)

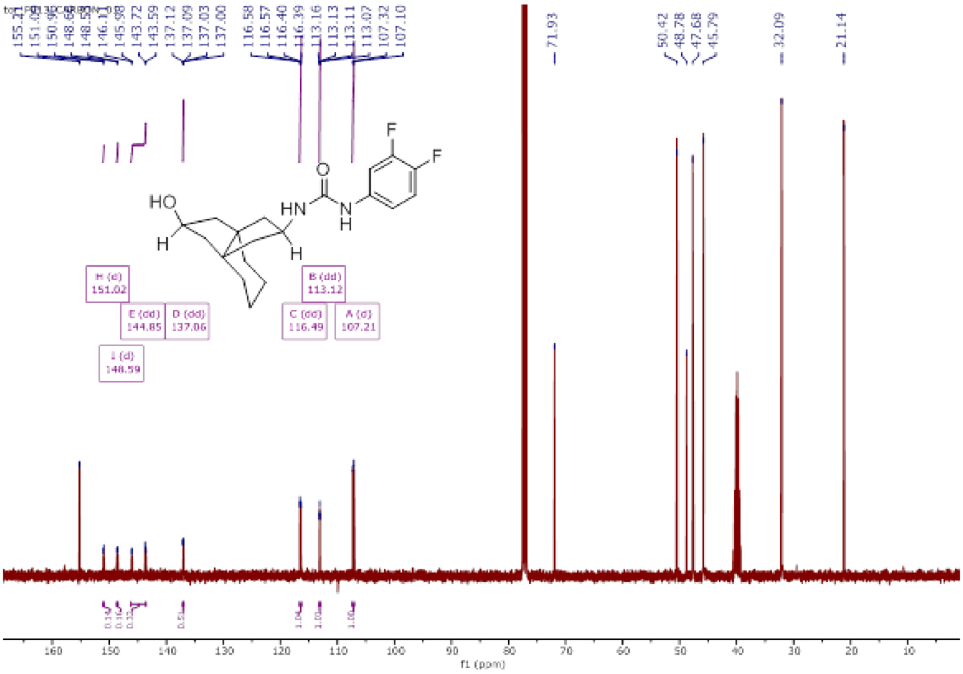

^{1}H NMR spectrum of 1-(3,4-Difluorophenyl)-3-(8-*syn*-11-*anti*-hydroxy[4.3.3]propellan-8-yl)urea (*anti,syn*-**26**)

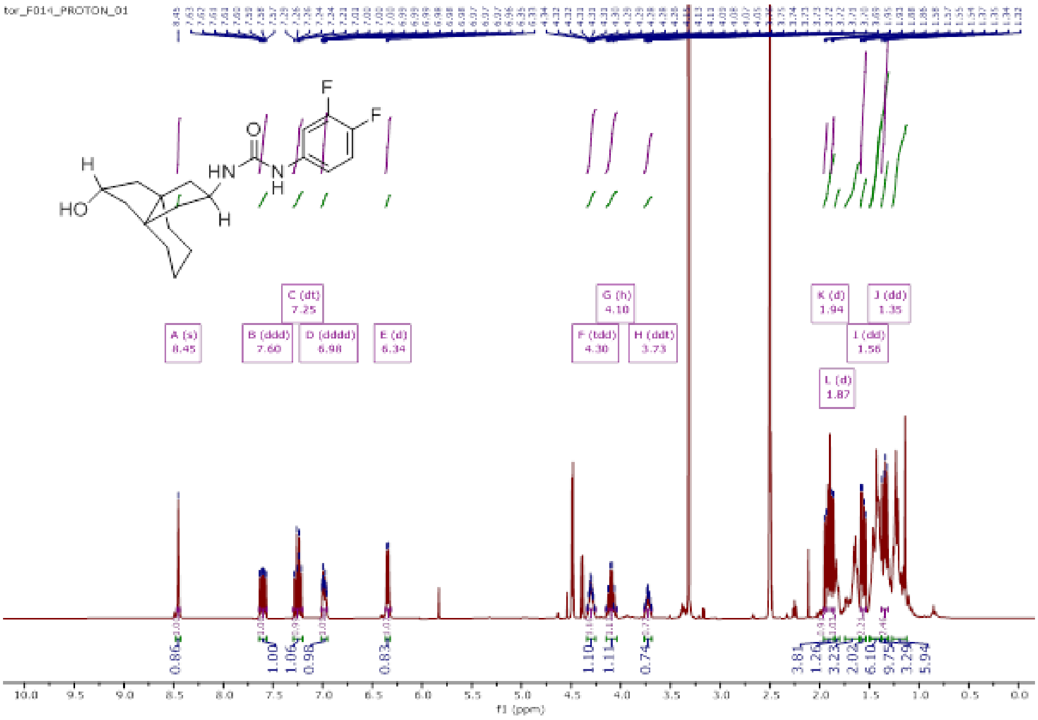

^{13}C NMR spectrum of 1-(3,4-Difluorophenyl)-3-(8-*syn*-11-*anti*-hydroxy[4.3.3]propellan-8-yl)urea (*anti,syn*-**26**)

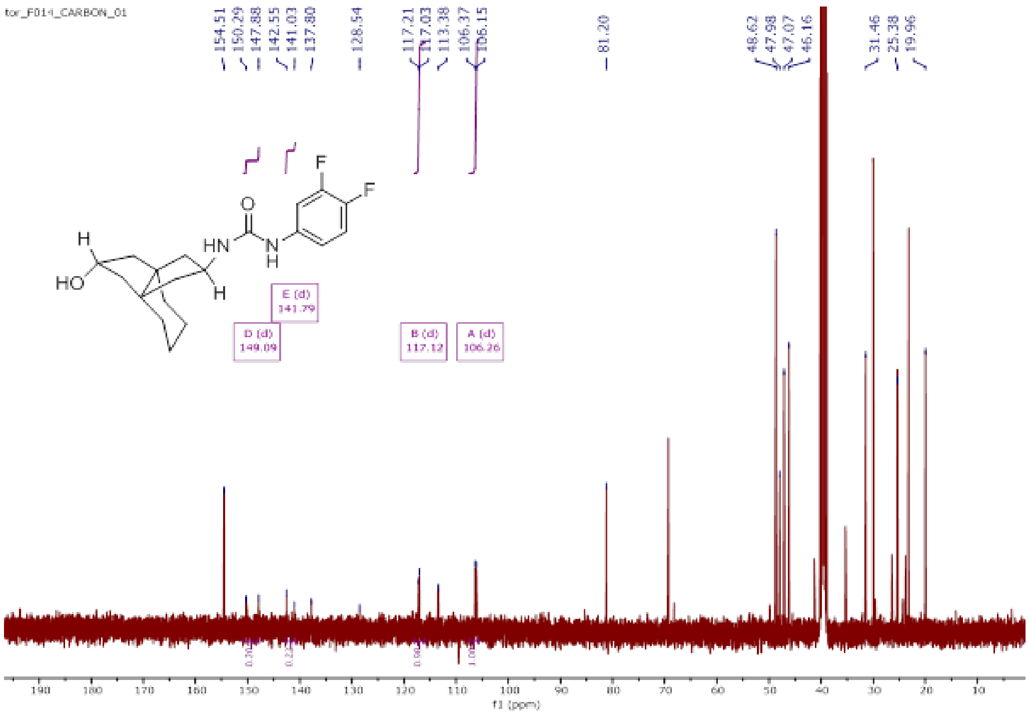

6. HPLC traces of selected compounds

HPLC trace of compound 8-*syn*-**4b**

HPLC

Analyzed: 06.12.12 02:40

Reported: 06.12.12 09:27
Processed: 06.12.12 09:27

Data Path: D:\WIN32APP\HSM\Chromni\DATA\5635\
Application: Chromni
Sample Name: H075
Injection from this vial: 1 of 1

Series:5635
Vial Number: 12
Vial Type: UNK
Volume: 5,0 ul

Chrom Type: HPLC Channel : 1

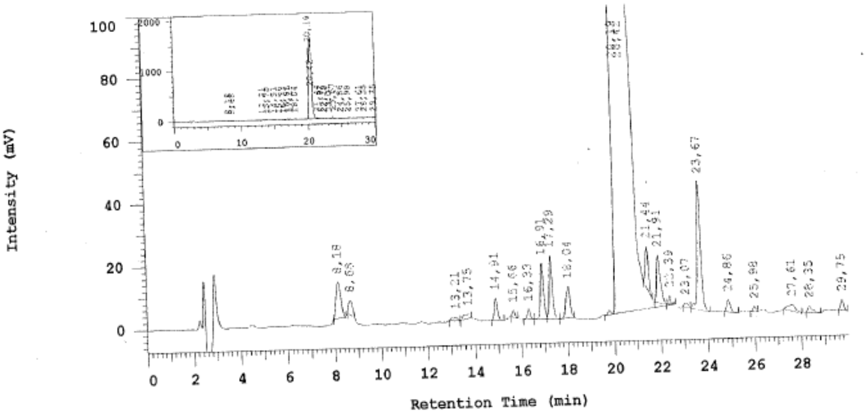

Acquisition Method: Chromni
Blank Subtr Sample Name: ACN
Column Type: 010
Solvent A: Wasser + 0,05%TFA

Developed by: Jens
Solvent B: ACN + 0,05%TFA

No.	RT	Area	Conc 1	BC
1	8,18	167488	0,380	BB
2	8,68	47540	0,108	BB
3	13,21	19128	0,043	BB
4	13,75	29455	0,067	BB
5	14,91	64447	0,146	BB
6	15,66	16481	0,037	BB
7	16,33	28292	0,064	BB
8	16,91	166523	0,378	BB
9	17,29	190380	0,432	BB
10	18,04	124935	0,283	BB
11	20,19	15227707	34,536	BV
12	20,42	27117286	61,502	VV
13	21,44	113499	0,257	TBB
14	21,91	169265	0,384	TBB
15	22,39	18265	0,041	TBB
16	23,07	15775	0,036	BB
17	23,67	417630	0,947	BB
18	24,86	41053	0,093	BB
19	25,98	11097	0,025	BB
20	27,61	49011	0,111	BB
21	28,35	26349	0,060	BB
22	29,75	30084	0,068	BB
		44091690	100,000	

Peak rejection level: 0

HPLC trace of compound 8-*anti*-**4g**

HPLC

Analyzed: 17.07.13 22:25

Reported: 18.07.13 09:37
Processed: 18.07.13 09:37

Data Path: D:\WIN32APP\HSM\Jens\DATA\2299\
Application: Jens
Sample Name: H091
Injection from this vial: 1 of 1

Series:2299
Vial Number: 8
Vial Type: UNK
Volume: 5,0 ul

Chrom Type: HPLC Channel : 1

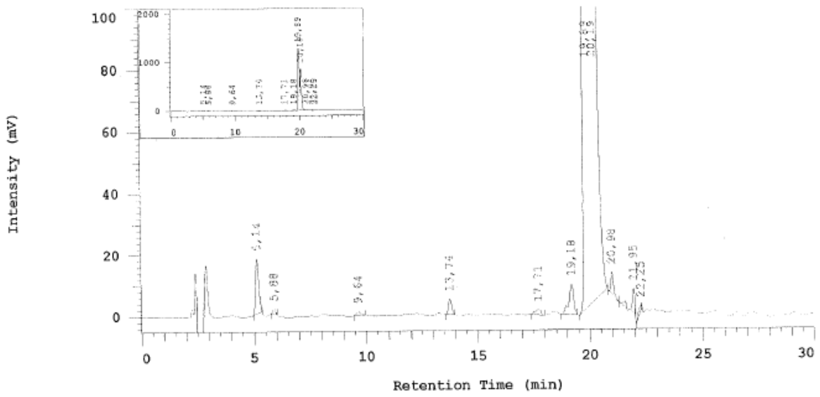

Acquisition Method: Chromni
Blank Subtr Sample Name: ACN
Column Type: 010
Solvent A: Wasser + 0,05%TFA

Developed by: Jens
Solvent B: ACN + 0,05%TFA

No.	RT	Area	Conc 1	BC
1	5,14	172756	0,637	BB
2	5,88	13001	0,048	MC
3	9,64	15322	0,057	MC
4	13,74	48150	0,178	BB
5	17,71	28556	0,105	BB
6	19,18	142071	0,524	MC
7	19,89	16017075	59,072	MC
8	20,19	10542464	38,881	MC
9	20,98	60440	0,223	MC
10	21,95	56304	0,208	MC
11	22,25	18535	0,068	MC
		27114674	100,000	

Peak rejection level: 0

97,95

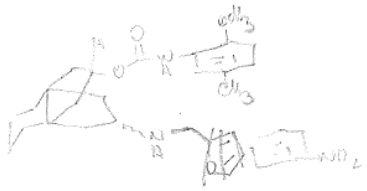

HPLC trace of compound 8-*anti*-**4i**

HPLC

Analyzed: 23.10.13 23:41

Reported: 24.10.13 10:19
Processed: 24.10.13 10:19

Data Path: D:\WIN32APP\HSM\Kirstin\DATA\0191\
Application: Kirstin
Sample Name: H100
Injection from this vial: 1 of 1

Series:0191
Vial Number: 10
Vial Type: UNK
Volume: 5,0 ul

Chrom Type: HPLC Channel : 1

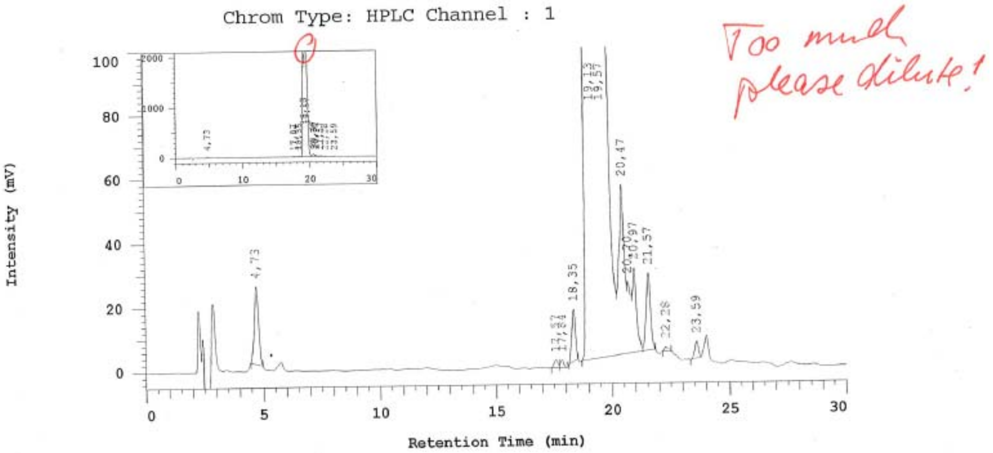

Acquisition Method: Chromni
Blank Subtr Sample Name: ACN
Column Type: 010
Solvent A: Wasser + 0,05%TFA

Developed by: Jens
Solvent B: ACN + 0,05%TFA

No.	RT	Area	Conc 1	BC
1	4,73	303242	0,316	BB
2	17,57	25144	0,026	BB
3	17,84	24191	0,025	BB
4	18,35	170387	0,177	MC
5	19,13	40504000	42,154	MC
6	19,57	53234374	55,403	MC
7	20,47	887475	0,924	MC
8	20,70	248082	0,258	MC
9	20,97	320438	0,333	MC
10	21,57	300676	0,313	MC
11	22,28	14774	0,015	BB
12	23,59	52828	0,055	MC
		96085611	100,000	

Peak rejection level: 0

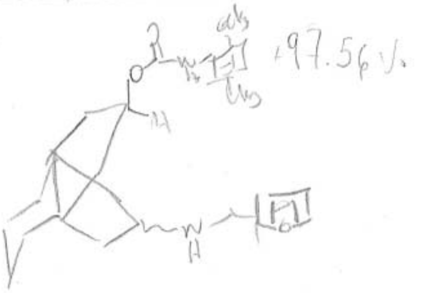

HPLC trace of compound 8-*anti*-**4k**

HPLC

Analyzed: 17.10.13 07:38

Reported: 18.10.13 10:29
Processed: 18.10.13 10:29

Data Path: D:\WIN32APP\HSM\Kirstin\DATA\0150\
Application: Kirstin
Sample Name: H101
Injection from this vial: 1 of 1

Series:0150
Vial Number: 19
Vial Type: UNK
Volume: 5,0 ul

Chrom Type: HPLC Channel : 1

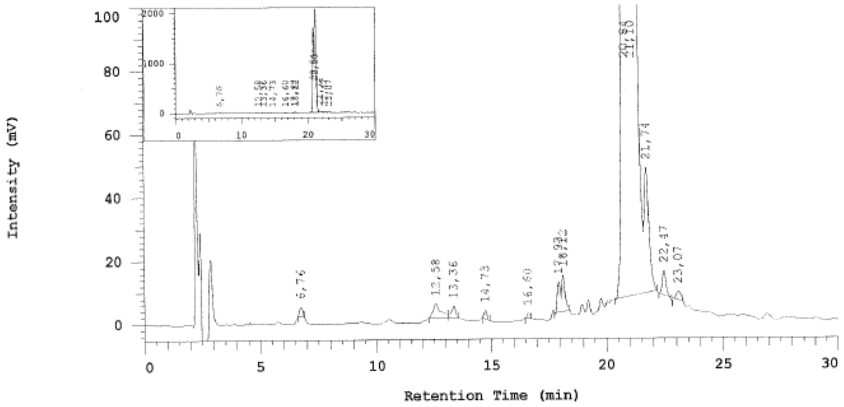

Acquisition Method: Chromni
Blank Subtr Sample Name: ACN
Column Type: 010
Developed by: Jens
Solvent A: Wasser + 0,05%TFA
Solvent B: ACN + 0,05%TFA

No.	RT	Area	Conc 1	BC
1	6,76	25202	0,051	BB
2	12,58	95955	0,195	MC
3	13,36	45187	0,092	MC
4	14,73	24192	0,049	BB
5	16,60	11643	0,024	BB
6	17,93	80493	0,164	MC
7	18,12	119871	0,244	MC
8	20,84	18454134	37,574	MC
9	21,10	29586668	60,241	MC
10	21,74	567181	1,155	MC
11	22,47	74143	0,151	MC
12	23,07	29453	0,060	MC
		49114122	100,000	

Peak rejection level: 0

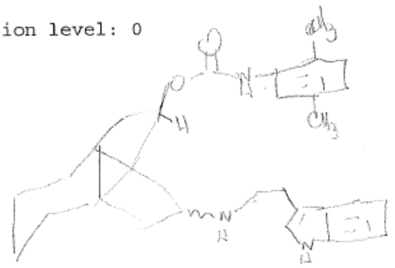

HPLC trace of compound 8-*anti*-**4m**

HPLC

Analyzed: 06.12.12 05:26

Reported: 06.12.12 09:30
Processed: 06.12.12 09:30

Data Path: D:\WIN32APP\HSM\Chromni\DATA\5639\
Application: Chromni
Sample Name: H078
Injection from this vial: 1 of 1

Series:5639
Vial Number: 15
Vial Type: UNK
Volume: 5,0 ul

Chrom Type: HPLC Channel : 1

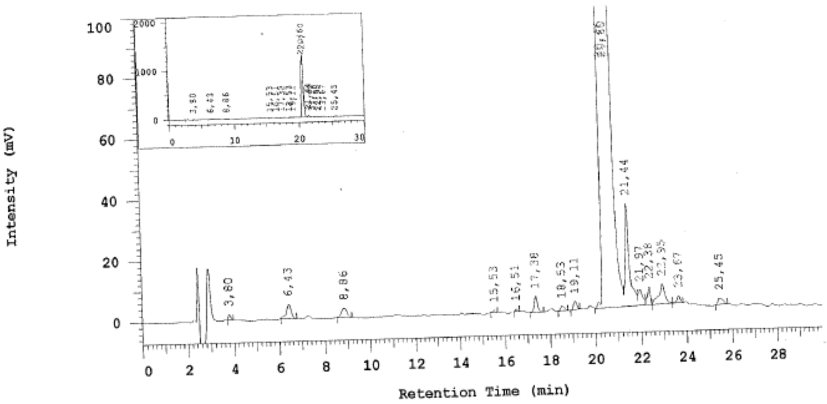

Acquisition Method: Chromni
Blank Subtr Sample Name: ACN
Column Type: 010
Solvent A: Wasser + 0,05%TFA

Developed by: Jens
Solvent B: ACN + 0,05%TFA

No.	RT	Area	Conc 1	BC
1	3,80	11406	0,042	MC
2	6,43	66165	0,246	MC
3	8,86	50747	0,189	MC
4	15,53	6796	0,025	BB
5	16,51	5637	0,021	BB
6	17,38	53090	0,198	BB
7	18,53	18974	0,071	MC
8	19,11	25775	0,096	BB
9	20,46	8725114	32,487	BV
10	20,60	17157504	63,884	VV
11	21,44	436720	1,626	MC
12	21,97	69968	0,261	MC
13	22,38	54590	0,203	MC
14	22,95	121222	0,451	BB
15	23,67	22101	0,082	BB
16	25,45	31662	0,118	MC
		26857471	100,000	

Peak rejection level: 0

HPLC trace of compound 8-*anti*-**4q**

HPLC

Analyzed: 06.12.12 06:49

Reported: 06.12.12 09:17
Processed: 06.12.12 09:17

Data Path: D:\WIN32APP\HSM\Chromni\DATA\5641\
Application: Chromni
Sample Name: H080
Injection from this vial: 1 of 1

Series:5641
Vial Number: 17
Vial Type: UNK
Volume: 5,0 ul

Chrom Type: HPLC Channel : 1

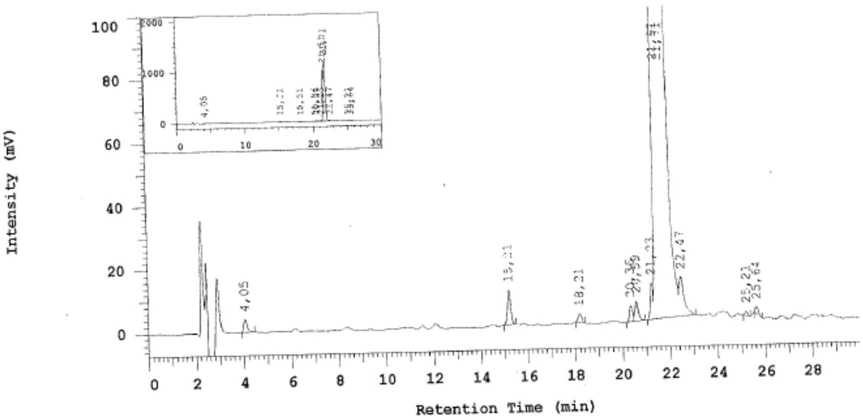

Acquisition Method: Chromni
Blank Subtr Sample Name: ACN
Column Type: 010
Developed by: Jens
Solvent A: Wasser + 0,05%TFA
Solvent B: ACN + 0,05%TFA

No.	RT	Area	Conc 1	BC
1	4,05	37783	0,137	MC
2	15,21	106818	0,387	BB
3	18,21	26273	0,095	BB
4	20,36	41397	0,150	BV
5	20,59	60919	0,221	VB
6	21,23	84258	0,305	MC
7	21,51	9791134	35,467	MC
8	21,71	17237033	62,439	MC
9	22,47	189073	0,685	MC
10	25,21	11595	0,042	BB
11	25,64	19986	0,072	BB
		27606269	100,000	

Peak rejection level: 0

HPLC trace of compound 11-*syn*-**13**

HPLC

Analyzed: 26.07.13 01:53

Reported: 26.07.13 09:32
Processed: 26.07.13 09:31

Data Path: D:\WIN32APP\HSM\Jens\DATA\2346\
Application: Jens
Sample Name: M001
Injection from this vial: 1 of 1

Series:2346
Vial Number: 12
Vial Type: UNK
Volume: 5,0 ul

Chrom Type: HPLC Channel : 1

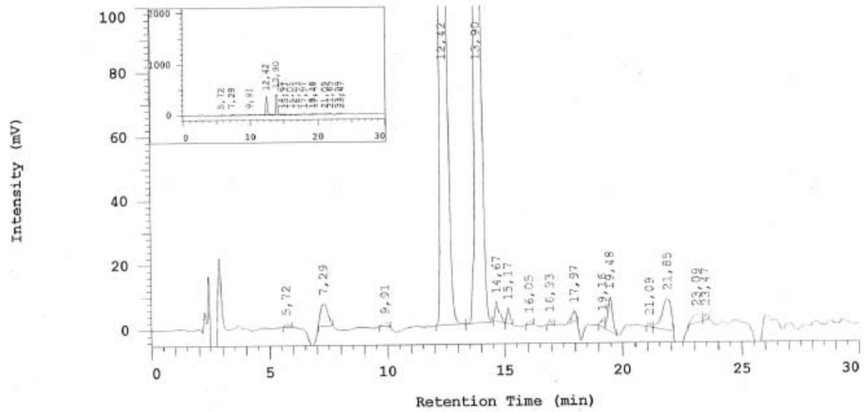

Acquisition Method: Chromni
Blank Subtr Sample Name: ACN
Column Type: 010
Solvent A: Wasser + 0,05%TFA

Developed by: Jens
Solvent B: ACN + 0,05%TFA

No.	RT	Area	Conc 1	BC
1	5,72	10845	0,089	BB
2	7,29	147149	1,206	MC
3	9,91	21413	0,175	BB
4	12,42	5586298	45,784	MC
5	13,90	5771912	47,305	MC
6	14,67	69230	0,567	MC
7	15,17	35812	0,294	MC
8	16,05	10100	0,083	BB
9	16,93	13708	0,112	BB
10	17,97	29050	0,238	MC
11	19,16	27861	0,228	MC
12	19,48	140728	1,153	MC
13	21,09	9830	0,081	MC
14	21,85	247715	2,030	MC
15	23,09	62696	0,514	MC
16	23,47	17063	0,140	MC
		12201410	100,000	

Peak rejection level: 0

93.09

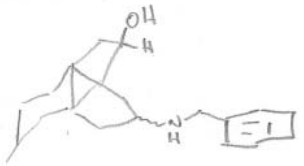

HPLC trace of compound *anti*-**14**

HPLC

Analyzed: 18.07.13 01:11

Reported: 18.07.13 09:43
Processed: 18.07.13 09:43

Data Path: D:\WIN32APP\HSM\Jens\DATA\2303\
Application: Jens
Sample Name: KBn2
Injection from this vial: 1 of 1

Series:2303
Vial Number: 11
Vial Type: UNK
Volume: 5,0 ul

Chrom Type: HPLC Channel : 1

not enough

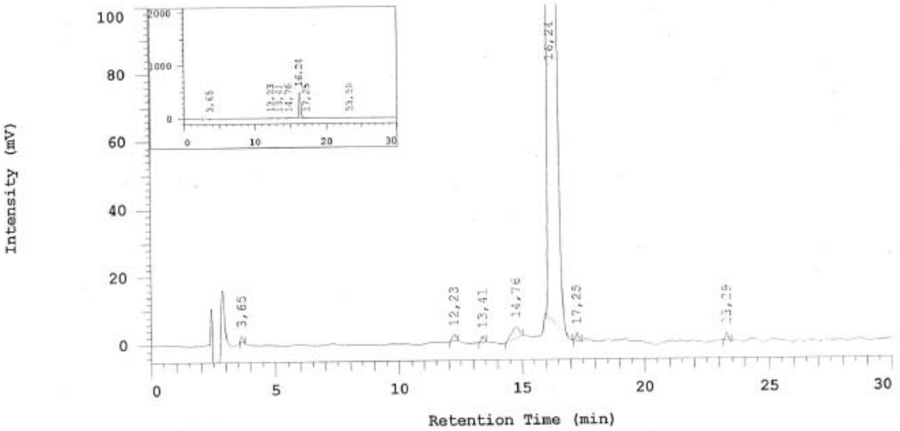

Acquisition Method: Chromni
Blank Subtr Sample Name: ACN
Column Type: 010
Solvent A: Wasser + 0,05%TFA

Developed by: Jens
Solvent B: ACN + 0,05%TFA

No.	RT	Area	Conc 1	BC
1	3,65	12763	0,146	MC
2	12,23	19312	0,221	MC
3	13,41	13426	0,154	BB
4	14,76	74151	0,848	BB
5	16,24	8590286	98,236	MC
6	17,25	18678	0,214	MC
7	23,29	15913	0,182	BB
		8744529	100,000	

Peak rejection level: 0

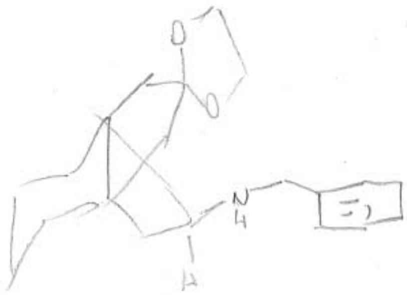

HPLC trace of compound **15**

HPLC

Analyzed: 08.08.13 20:54

Reported: 09.08.13 08:59
Processed: 09.08.13 08:59

Data Path: D:\WIN32APP\HSM\Jens\DATA\2488\
Application: Jens
Sample Name: PA11
Injection from this vial: 1 of 1

Series:2488
Vial Number: 11
Vial Type: UNK
Volume: 5,0 ul

Chrom Type: HPLC Channel : 1

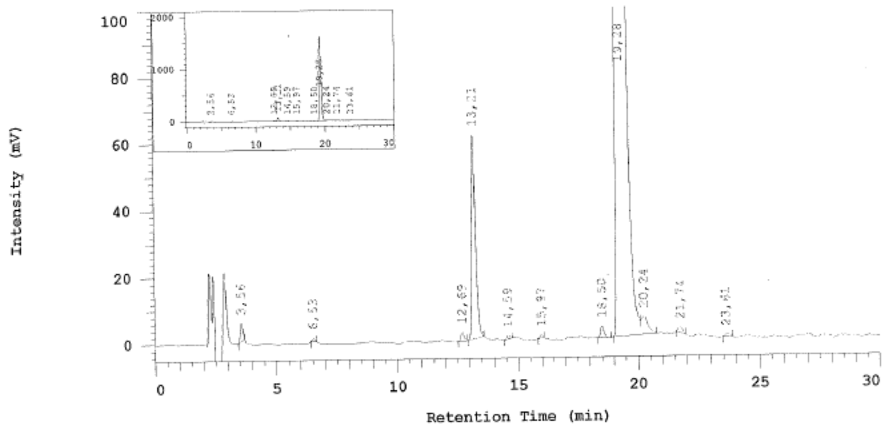

Acquisition Method: Chromni
Blank Subtr Sample Name: ACN
Column Type: 010
Developed by: Jens
Solvent A: Wasser + 0,05%TFA
Solvent B: ACN + 0,05%TFA

No.	RT	Area	Conc 1	BC
1	3,56	37016	0,134	BB
2	6,53	8309	0,030	BB
3	12,69	24205	0,088	BB
4	13,21	636001	2,301	BB
5	14,59	8386	0,030	BB
6	15,97	6930	0,025	BB
7	18,50	34141	0,124	BB
8	19,28	26754505	96,792	MC
9	20,24	102526	0,371	MC
10	21,74	18825	0,068	BB
11	23,61	10324	0,037	BB
		27641168	100,000	

Peak rejection level: 0

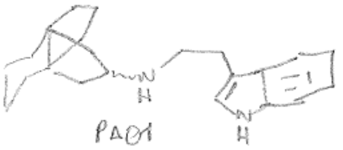

PA01

HPLC trace of compound **17**

HPLC

Analyzed: 08.08.13 18:08

Reported: 09.08.13 08:54
Processed: 09.08.13 08:54

Data Path: D:\WIN32APP\HSM\Jens\DATA\2484\
Application: Jens
Sample Name: PA02
Injection from this vial: 1 of 1

Series:2484
Vial Number: 8
Vial Type: UNK
Volume: 5,0 ul

Chrom Type: HPLC Channel : 1

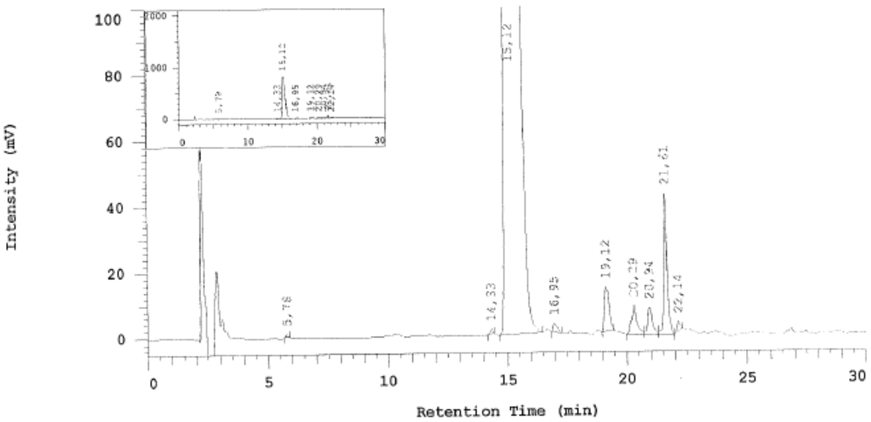

Acquisition Method: Chromni
Blank Subtr Sample Name: ACN
Column Type: 010
Solvent A: Wasser + 0,05%TFA

Developed by: Jens
Solvent B: ACN + 0,05%TFA

No.	RT	Area	Conc 1	BC
1	5,78	4678	0,020	BB
2	14,33	10069	0,043	BB
3	15,12	22739411	96,145	BB
4	16,95	32452	0,137	MC
5	19,12	185275	0,783	MC
6	20,29	132846	0,562	BB
7	20,94	110080	0,465	BB
8	21,61	410747	1,737	BB
9	22,14	25659	0,108	BB
		23651217	100,000	

Peak rejection level: 0

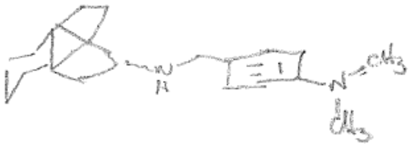

HPLC trace of compound **18**

[illegible]
Sample Name: PA06-Hector

```
=====================================================================
Acq. Operator   : SYSTEM                        Seq. Line :   5
Acq. Instrument : LCMS                           Location : P1-B-04
Injection Date  : 25-Oct-13 14:54:38                  Inj :   1
                                               Inj Volume : 1.000 µl
Acq. Method     : C:\CHEM32\1\DATA\SEQUENZEN\2013_10_25 2013-10-25 14-02-52\ROUTINE_12MIN_POSITIV.M
Last changed    : 25-Oct-13 14:02:52 by SYSTEM
Analysis Method : C:\CHEM32\1\METHODS\ROUTINE_12MIN_POSITIV.M
Last changed    : 25-Oct-13 15:32:26 by SYSTEM
Method Info     : Gradient und Fragmentor 100

Additional Info : Peak(s) manually integrated
```

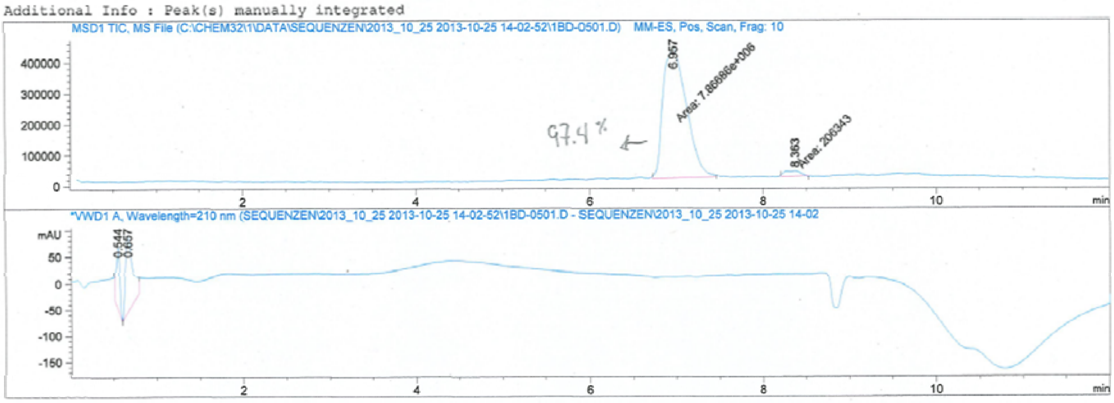

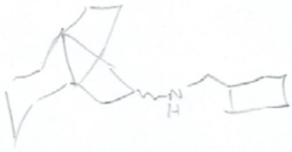

LCMS 25-Oct-13 15:49:43 SYSTEM   Page 1 of 2

HPLC trace of compound *anti-anti*-**22**

HPLC

Analyzed: 27.06.12 21:53

Reported: 28.06.12 11:05
Processed: 28.06.12 11:05

Data Path: D:\WIN32APP\HSM\Chromni\DATA\4938\
Application: Chromni
Sample Name: H064
Injection from this vial: 1 of 1

Series:4938
Vial Number: 6
Vial Type: UNK
Volume: 5,0 ul

Chrom Type: HPLC Channel : 1

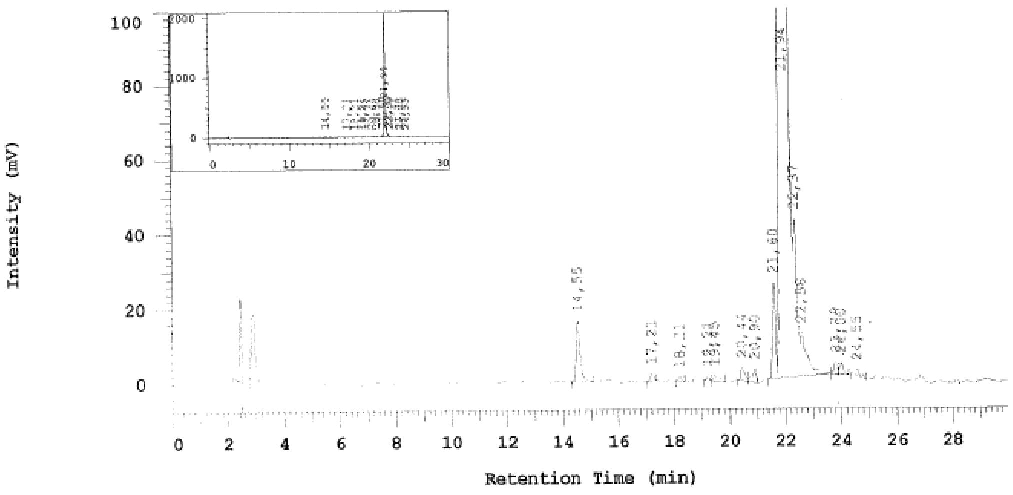

Acquisition Method: Chromni
Blank Subtr Sample Name: ACN
Column Type: 010
Developed by: Jens
Solvent A: Wasser + 0,05%TFA
Solvent B: ACN + 0,05%TFA

No.	RT	Area	Conc 1	BC
1	14,55	165909	0,656	MC
2	17,21	15061	0,060	BB
3	18,21	8192	0,032	BB
4	19,21	8637	0,034	BB
5	19,45	26641	0,105	MC
6	20,44	39225	0,155	BV
7	20,90	38025	0,150	VB
8	21,60	191308	0,756	MC
9	21,94	24174628	95,527	MC
10	22,37	349719	1,382	MC
11	22,58	211391	0,835	MC
12	23,79	24873	0,098	BV
13	24,00	29060	0,115	VB
14	24,55	23875	0,094	BB
		25306544	100,000	

Peak rejection level: 0

HPLC trace of compound **23a**

HPLC

Analyzed: 31.10.13 03:46

Data Path: D:\WIN32APP\HSM\Kirstin\DATA\0216\
Application: Kirstin
Sample Name: T12N
Injection from this vial: 1 of 1

Reported: 31.10.13 14:17
Processed: 31.10.13 14:17

Series:0216
Vial Number: 15
Vial Type: UNK
Volume: 5,0 ul

Chrom Type: HPLC Channel : 1

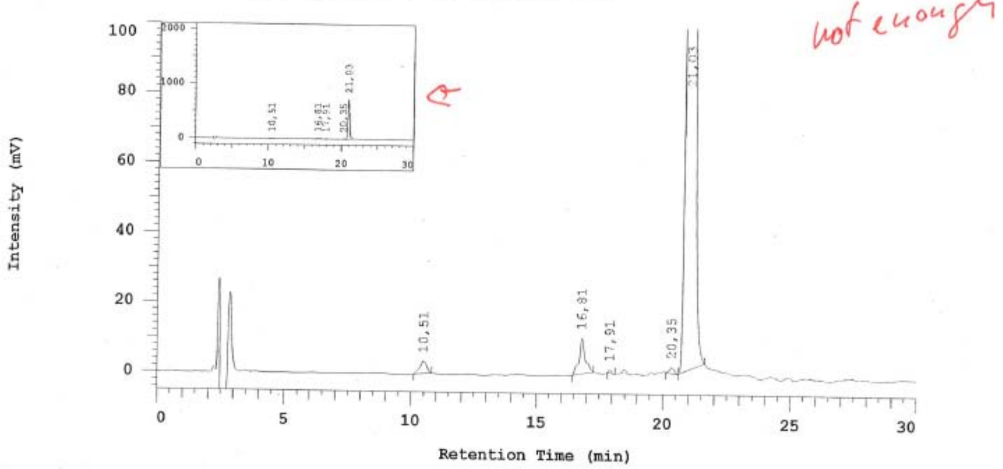

Acquisition Method: Chromni
Blank Subtr Sample Name: ACN
Column Type: 010
Solvent A: Wasser + 0,05%TFA
Developed by: Jens
Solvent B: ACN + 0,05%TFA

No.	RT	Area	Conc 1	BC
1	10,51	64315	0,643	MC
2	16,81	167459	1,675	MC
3	17,91	6878	0,069	BB
4	20,35	20935	0,209	MC
5	21,03	9739804	97,404	BB
		9999391	100,000	

Peak rejection level: 0

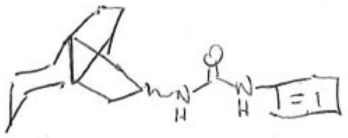

repurified

HPLC trace of compound **23d**

HPLC

Analyzed: 08.08.13 07:01

Reported: 08.08.13 10:37
Processed: 08.08.13 10:37

Data Path: D:\WIN32APP\HSM\Jens\DATA\2470\
Application: Jens
Sample Name: T015
Injection from this vial: 1 of 1

Series:2470
Vial Number: 20
Vial Type: UNK
Volume: 5,0 ul

Chrom Type: HPLC Channel : 1

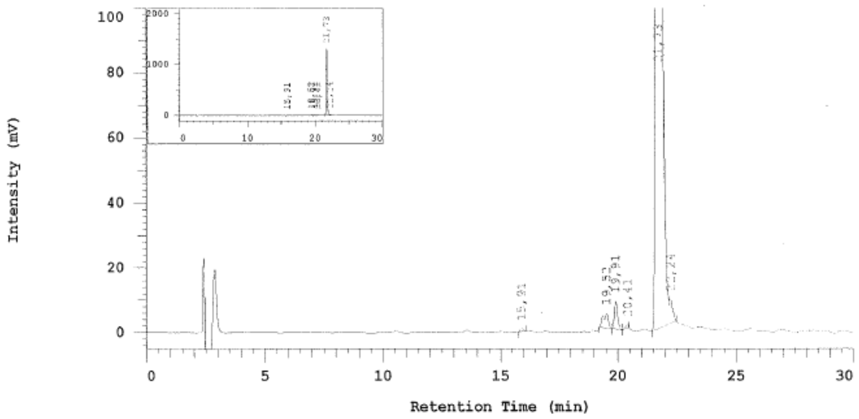

Acquisition Method: Chromni
Blank Subtr Sample Name: ACN
Column Type: 010
Solvent A: Wasser + 0,05%TFA

Developed by: Jens
Solvent B: ACN + 0,05%TFA

No.	RT	Area	Conc 1	BC
1	15,91	7978	0,060	BB
2	19,52	68577	0,514	MC
3	19,91	73792	0,553	MC
4	20,41	7376	0,055	BB
5	21,73	13110874	98,245	BV
6	22,24	76482	0,573	MC
		13345079	100,000	

Peak rejection level: 0

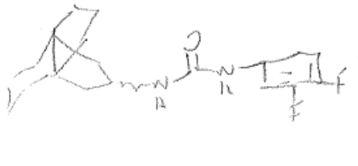

HPLC trace of compound *anti*-**25**

HPLC

Analyzed: 12.09.13 04:11

Reported: 12.09.13 11:19
Processed: 12.09.13 11:19

Data Path: D:\WIN32APP\HSM\Jens\DATA\2747\
Application: Jens
Sample Name: FOO2
Injection from this vial: 1 of 1

Series:2747
Vial Number: 14
Vial Type: UNK
Volume: 5,0 ul

Chrom Type: HPLC Channel : 1

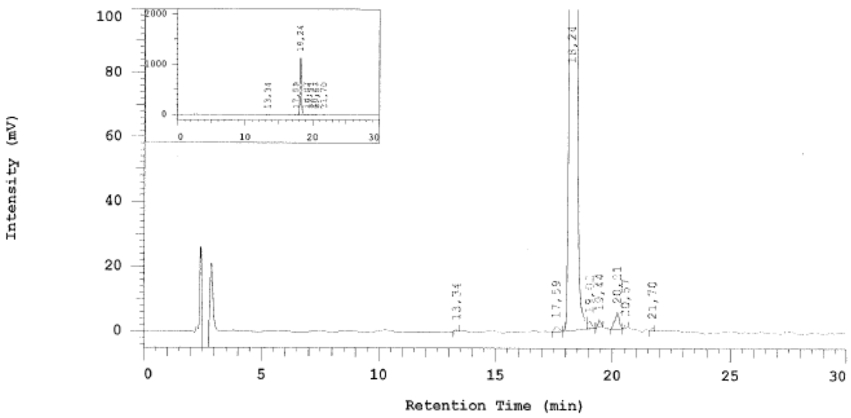

Acquisition Method: Chromni
Blank Subtr Sample Name: ACN
Column Type: 010
Developed by: Jens
Solvent A: Wasser + 0,05%TFA
Solvent B: ACN + 0,05%TFA

No.	RT	Area	Conc 1	BC
1	13,34	5990	0,050	BB
2	17,59	19864	0,165	BB
3	18,24	11921338	98,743	BV
4	19,02	27056	0,224	MC
5	19,44	20297	0,168	BB
6	20,21	65800	0,545	BB
7	20,57	5769	0,048	BB
8	21,70	7024	0,058	BB
		12073138	100,000	

Peak rejection level: 0

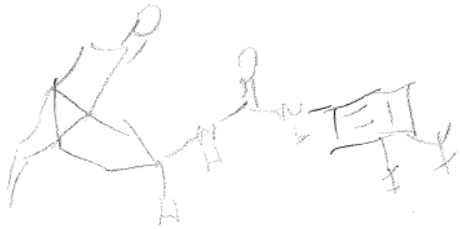

Supplement: Supplementary file 1 [file ijms-22-05685-s001.zip › ijms-1200597-supplementary.pdf]
